# AIE‐Active Difluoroboron Complexes with N,O‐Bidentate Ligands: Rapid Construction by Copper‐Catalyzed C−H Activation

**DOI:** 10.1002/advs.202101814

**Published:** 2021-07-26

**Authors:** Guangying Tan, Iván Maisuls, Felix Strieth‐Kalthoff, Xiaolong Zhang, Constantin Daniliuc, Cristian A. Strassert, Frank Glorius

**Affiliations:** ^1^ Organisch‐Chemisches Institut Westfälische Wilhelms‐Universität Münster Corrensstraße 40 Münster 48149 Germany; ^2^ Institut für Anorganische und Analytische Chemie CeNTech CiMIC SoN Westfälische Wilhelms‐Universität Münster Heisenbergstraße 11 Münster 48149 Germany

**Keywords:** aggregation‐induced emission, BF_2_ complexes with N,O‐bidentate ligands, C—H activation, copper catalysis, solid‐state fluorescence

## Abstract

The development of organic materials with high solid‐state luminescence efficiency is highly desirable because of their fundamental importance and applicability in optoelectronics. Herein, a rapid construction of novel BF_2_ complexes with N,O‐bidentate ligands by using Cu(BF_4_)_2_•6H_2_O as a catalyst and BF_2_ source is disclosed, which avoids the need for pre‐composing the N,O‐bidentate ligands and features a broad substrate scope and a high tolerance level for sensitive functional groups. Moreover, molecular oxygen is employed as the terminal oxidant in this transformation. A library of 36 compounds as a new class of BF_2_ complexes with remarkable photophysical properties is delivered in good to excellent yields, showing a substituent‐dependency on the photophysical properties, derived from the *π*–π* character of the photoexcited state. In addition, aggregation‐induced emission (AIE) is observed and quantified for the brightest exemplars. The excited state properties are fully investigated in solids and in THF/H_2_O mixtures. Hence, a new series of photofunctional materials with variable photophysical properties is reported, with potential applications for sensing, bioimaging, and optoelectronics.

## Graphical abstract

A novel strategy is developed for the rapid construction of aggregation‐induced emission (AIE) active BF_2_ complexes with N,O‐bidentate ligands through a copper‐catalyzed cascade C—H activation/acyloxylation and difluoroboronation, which avoids the need for pre‐composing the N,O‐bidentate ligands. A library of 36 compounds as a new class of AIE‐active BF_2_ complexes with remarkable photophysical properties is delivered in good to excellent yields.

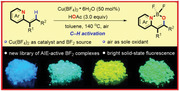

## 1 Introduction

Since the seminal discovery of organic difluoroboron (BF_2_) complexes by Treibs and Kreuzer in 1968,^[^ ^1^ ^]^ their analogues have attracted a continuing interest of organic chemists and materials scientists, owing to their outstanding photophysical properties such as high fluorescence brightness and quantum yields, tunable emission, as well as high photo‐ and chemical‐stability.^[^ ^2^ ^]^ Despite significant developments, the majority of organic BF_2_ complexes exhibit strong luminescence in dilute solutions. Their emission in aggregates or in the solid state are usually spoiled by self‐absorption and dissipative intermolecular interactions, referred to as aggregation‐caused quenching (ACQ) effects.^[^ ^3^ ^]^ As a result, the application of organic BF_2_ complexes has been confined to the fields of fluorescence sensing and bioimaging for a long time.^[^ ^2^, ^4^ ^]^ Due to the high demand for organic luminogens with strong solid‐state emission as organic optoelectronic materials for applications including organic lasers, organic light‐emitting diodes (OLEDs), and organic light‐emitting transistors (OLETs),^[^ ^5^ ^]^ the development of organic BF_2_ complexes with high solid‐state luminescence efficiency is highly desirable.

**Scheme 1:**
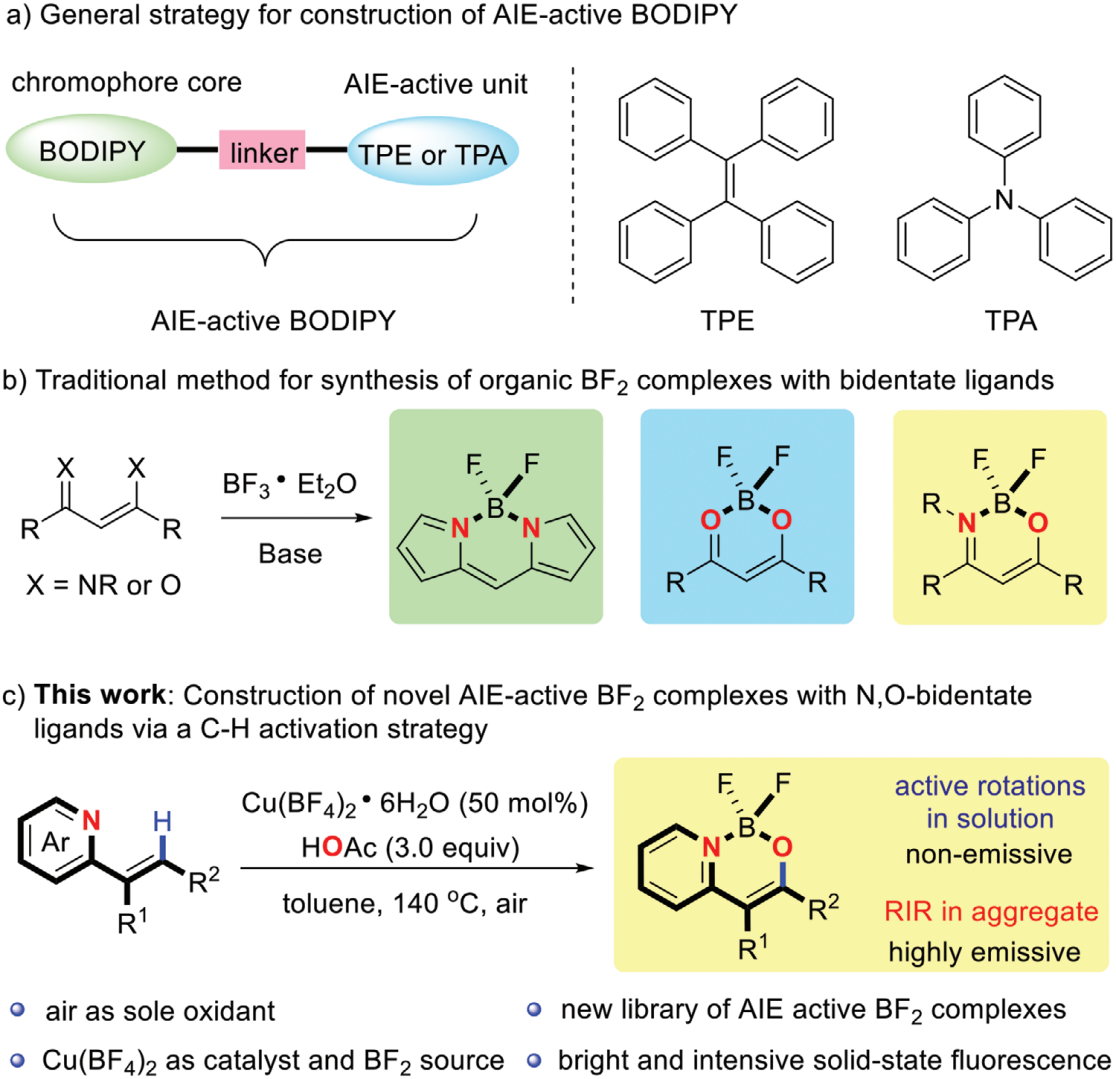
Synthesis of organic difluroboron (BF_2_) complexes. RIR: restriction of intramolecular rotation.

Aggregation‐induced emission (AIE) describes the behavior of organic molecules that exhibit dim or no emission in dilute solutions but significantly enhanced luminescence in aggregates or in the solid state, which has been widely accepted to be an efficient strategy exploiting the reduction of rotovibrational relaxation pathways.^[^ ^6^ ^]^ In recent years, a number of AIE‐active organic BF_2_ complexes have been achieved,^[^ ^7^, ^8^, ^9^, ^10^, ^11^ ^]^ and their applications as aggregate‐state emitters for OLEDs, stimuli‐response switches, and in other fields have also been demonstrated.^[^ ^12^ ^]^ Despite tremendous efforts over the last decade, the design and application of the AIE‐active BF_2_ complexes is still facing significant challenges. Most notably, established methods of achieving AIE activity of classical BF_2_ complexes (BODIPY) mainly focus on linking AIE‐active units such as tetraphenylethylene (TPE) and triphenylamine (TPA) to the chromophoric core (**Scheme** **1**a).^[^ ^7^, ^8^, ^9^ ^]^ Although ACQ effects of organic BF_2_ complexes could be suppressed to some degree by the introduction of bulky moieties, the aggregate‐state quantum yields for most AIE‐active BF_2_ complexes still remained low, thus limiting their further applications. Therefore, developing a new strategy or a proper platform to build up a library of AIE‐active BF_2_ complexes is still an enormous challenge.

Generally, organic BF_2_ complexes are prepared by the reaction of a prefabricated organic bidentate ligand precursors with boron trifluoride–diethyl etherate in the presence of a base (Scheme 1b).^[^ ^2a^ ^]^ Thus, the development of organic BF_2_ complexes is mainly dependent on the design and synthesis of such organic ligands. However, the structural diversity of organic BF_2_ complexes is usually limited by the lack of suitable synthetic methods and the low tolerance of functional groups. Over the past two decades, transition‐metal catalyzed C—H activation has developed rapidly and has been proven to be a very straightforward approach for the construction of functional materials.^[^ ^13^, ^14^ ^]^ Compared to the traditional synthesis of organic BF_2_ complexes, transition‐metal catalyzed C—H activations might provide a unique opportunity to access new skeletons, and understand and manipulate the specific properties of molecular structures. Due to the highly efficient and modular features of these C—H activation‐based methods, it is usually possible to assemble a versatile molecular library in a single step. However, the synthesis of BF_2_ complexes via C—H activation remains extremely rare to date. In 2020, we reported our preliminary study of C—H activation‐based copper‐catalyzed one‐shot synthesis of ACQ‐type organic BF_2_ complexes from 2‐phenylpyridine derivatives.^[^ ^15^ ^]^ Costly and air‐sensitive AgBF_4_ was needed stoichiometrically as the oxidant and BF_2_ source for the transformation. As such, that protocol showed low reactivity to the C(alkenyl)—H bond and poor tolerance of sensitive functional groups such as acyl, cyano, methylthio, and alkenyl. To the best of our knowledge, the transition‐metal catalyzed C(alkenyl)—H bond activation/acyloxylation and difluoroboronation remain an unknown transformation.

Herein, we describe a full study of the construction of solid‐state emissive luminogens via copper‐catalyzed C—H activation without using AgBF_4_, which provides a rapid and efficient method to access a new library of organic BF_2_ complexes with N,O‐bidentate ligands (Scheme 1c). Noteworthy features of this study include: 1) the use of the accessible and inexpensive Cu(BF_4_)_2_•6H_2_O (50 mol%) as a catalyst and BF_2_ source to transform a broad range of 2‐vinylpyridine derivatives into the corresponding BF_2_ complexes with N,O‐bidentate ligands in excellent yields; 2) molecular oxygen (precisely: ^3^O_2_) is employed as the terminal oxidant (electron acceptor) in this transformation, which eliminates by‐product formation of stoichiometric silver salts and thereby enhances the practicality of this protocol; 3) most notably, the photophysical properties of these obtained BF_2_ complexes were fully investigated and quantified, both at room temperature and 77K. In addition, the AIE nature of selected BF_2_ complexes was studied and rationalized through the character of the excited states. Finally, these remarkable results were further evaluated and interpreted by a combination of experimental and theoretical calculations.

## 2 Results and Discussion

### 2.1 Evaluation of the Reaction Parameters

**Scheme 2:**
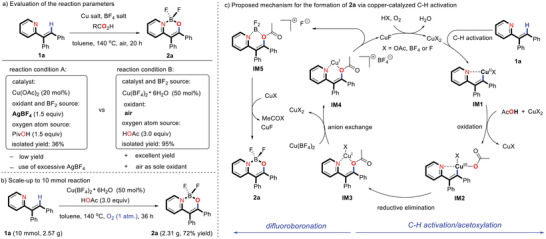
a) Evaluation of the reaction parameters. b) Scale‐up to 10 mmol reaction. c) Proposed mechanism for the formation of **2a** via copper‐catalyzed C—H activation

Our investigation was initiated by applying (*E*)‐2‐(1,2‐diphenylvinyl)pyridine (**1a**) as a model substrate under a previously established reaction system for 2‐phenylpyridine derivatives, which was composed of Cu(OAc)_2_ (20 mol%), AgBF_4_ (1.5 equiv.), and PivOH (1.5 equiv.) in toluene (0.2 m) under air atmosphere at 140 °C for 20 h (**Scheme** **2**a, reaction condition A). Indeed, the desired product **2a** could be obtained in 35% yield. However, the use of excessive AgBF_4_ as oxidant and BF_2_ source increased the economic and environmental costs and thereby hindered the sustainability of large‐scale applications. From the perspective of sustainable chemistry and its applications, we turned to the development of a “greener” reaction system. To our delight, after an extensive screening of reaction parameters, we identified the combination of Cu(BF_4_)_2_•6H_2_O (50 mol%) and AcOH (3.0 equiv.) in toluene under air atmosphere at 140 °C for 20 h as the optimum system to deliver the desired BF_2_ complex **2a** in 95% isolated yield (Scheme 2a, reaction condition B). In this reaction system, Cu(BF_4_)_2_•6H_2_O services as a catalyst and BF_2_ source, and the O_2_ in air is employed as the terminal oxidant for this transformation. A summary of the optimization results is shown in Table S1, Supporting Information. Several optimization results are worth noting: (1) The yield of **2a** was reduced to 34% when running the reaction under argon atmosphere, suggesting that the O_2_ in air plays the role of the oxidant (Table S1, entry 4, Supporting Information); (2) by removal of AcOH, the reaction was shut down completely, implying that AcOH could be the source of oxygen atoms for the transformation (Table S1, entry 3, Supporting Information);^[^ ^16^ ^]^ (3) compound **2a** could be obtained in 83% yield when reducing the loading of Cu(BF_4_)_2_•6H_2_O to 20 mol% and adding 1.0 equiv. of additional NaBF_4_ as BF_2_ source (Table S1, entry 5, Supporting Information). In addition, the yield of **2a** was further reduced to 72% when replacing Cu(BF_4_)_2_•6H_2_O (20 mol%) by Cu(OAc)_2_ (20 mol%) as catalyst (Table S1, entry 7, Supporting Information); (4) the reaction temperature significantly affected the transformation, and only trace amounts of **2a** were detected when the reaction was performed at 100 °C (Table S1, entry 8, Supporting Information). Furthermore, sensitivity screening revealed that this reaction was relatively robust, only suffering from slightly diminshed yields at lower temperatures or larger scales (see Table S2 and Figure S1, Supporting Information, for the details).^[^ ^17^ ^]^ Finally, to highlight the practicality of this protocol, we further illustrated the scalability of the reaction. When the reaction was scaled up to 10 mmol with a gram scale, the desired **2a** was obtained in 72% yield by using O_2_ (1 atm.) as the oxidant (Scheme 2b).

### 2.2 Proposed Mechanism

Based on the above observations and our previous work,^[^ ^15^ ^]^ we surmised that the formation of **2a** was unlocked through the elaborate copper‐catalyzed pathway illustrated in Scheme 2c, which involves C—H activation/acyloxylation in tandem with difluoroboronation. In the C—H acyloxylation process, (*E*)‐2‐(1,2‐diphenylvinyl)pyridine (**1a**) undergoes C—H activation to form intermediate **IM1**, oxidation to form Cu(III) intermediate **IM2**, and reductive elimination to form intermediate **IM3**, successively. Then, intermediate **IM3** can react with Cu(BF_4_)_2_ through anion exchange to afford **IM4**, which decomposes to give intermediate **IM5** along with CuF due to its instability at high temperature. Subsequently, intermediate **IM5** can deliver the desired product **2a** by releasing a molecule of MeCOF, which is presumably quenched by copper salts in the reaction system. Finally, the generated Cu(I) species is re‐oxidized to the Cu(II) species by O_2_ to close the catalytic cycle.

### 2.3 Scope

With the optimized reaction conditions in hand, the scope of this reaction was examined. As summarized in **Table** **1**, AIE‐active BF_2_ complexes with N,O‐bidentate ligands were obtained as a library of 36 compounds via copper‐catalyzed C—H activation, which features a broad substrate scope and a high tolerance level for sensitive functional groups. (*E*)‐2‐(1,2‐Diarylvinyl)pyridines (**1**) bearing electron donating or electron withdrawing substituents on the ortho‐, meta‐, or para‐position of the phenyl rings smoothly underwent this transformation, producing the corresponding products in good to excellent yields (Table 1, **2a**–**2w**). Satisfactorily, a variation of the substitution pattern on the pyridine moiety was also tolerated in this reaction (Table 1, **2** **r**–**2** **w**). Moreover, the pyridyl unit could be replaced with isoquinolinyl unit as chelating group without any decrease in reactivity (Table 1, **2** **x**). Other *N*‐heterocycle units such as pyrimidinyl, pyrazolyl, imidazolyl, thiazolyl, and oxazolyl could not be applicable in this protocol. Furthermore, various (hetero)aryls could be installed on the 4‐position of the pyridine ring, delivering the corresponding BF_2_ complexes in excellent yields (Table 1, **2y–2ai**). A variety of functional groups, especially sensitive functional groups such as TMS, MeO, MeS, CHO, Ac, CO_2_Me, NO_2_, and even CN were tolerated well.

**Table 1:** Scope of copper‐catalyzed synthesis of AIE‐active BF_2_ complexes with N,O‐bidentate ligands and photophysical data of the resulting products^[Link]^, ^[Link]^

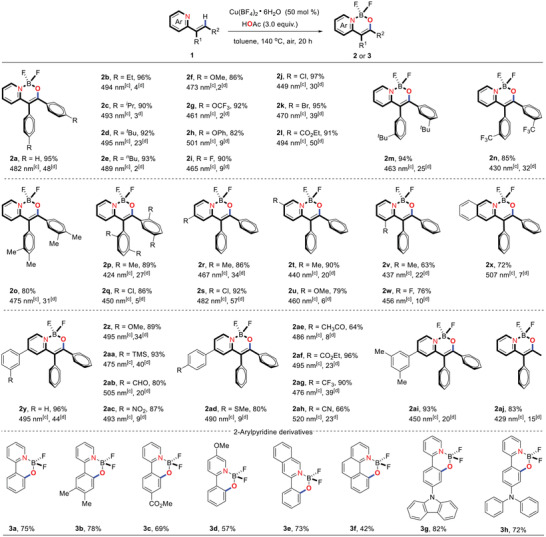

^a)^ Standard conditions were used: substrates 1 (0.1 mmol), Cu(BF_4_)•6H_2_O (0.05 mmol, 50 mol%), and AcOH (0.3 mmol, 3.0 equiv.) in toluene (0.5 mL, 0.2 m) at 140 °C under air atmosphere for 20 h;

^b)^ Isolated yields are indicated as percentages;

^c)^ Photoluminescence maxima in the solid state;

^d)^Fluorescence quantum yield [%] of the solid powder (±2%, RT).

To further demonstrate the efficiency of this protocol, a series of 2‐arylpyridine derivatives were employed as reactants in this transformation, delivering the corresponding products in good yields (**3a**–**3h**). Due to its nonplanar characteristics, excellent electron‐donating properties, and hole‐transporting capabilities, the triphenylamine (TPA) unit and its derivatuves have been widely used in organic optoelectronic materials. To our delight, the TPA‐containing pyridines could smoothly undergo this protocol to form the desired BF_2_ complex **3h** in 72% yield. It needs to be noted that the triphenylamine unit could not be tolerated in our previous method.^[^ ^15^ ^]^ Therefore, compared with our previous method, the current protocol exhibited broader substrate applicability and could be used as a general method for the synthesis of organic difluoroboron complexes with N,O‐bidentate ligands.

### 2.4 Photophysical Properties

As documented in the bibliographic literature,^[^ ^2^ ^]^ BF_2_ derivatives can exhibit efficient photoluminescence. In this frame, different photophysical studies were carried out to understand the photophysical properties of these obtained compounds. Hence, the photoluminescence quantum yields (*Φ*_F_) and emission maxima of all the obtained BF_2_ complexes were measured in the solid state, and the results are summarized in Table 1. Interestingly, a strong substituent‐dependency is observable, as the emission maxima shift from 430 to 520 nm and the *Φ*_F_ can reach up to 50%. These variations depend on the *π*‐donating ability of the substituents, which most likely destabilize the highest occupied molecular orbital (HOMO) while diminishing the HOMO–LUMO gap and the excited state energy (vide infra).

**Figure 1:**
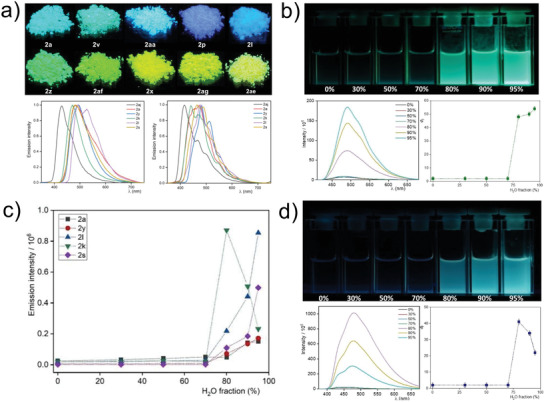
Photophysical properties of selected BF_2_ complexes. a) Top: fluorescence photographs of solid powder of representative BF_2_ complexes, taken under the illumination of a UV lamp; bottom left: emission spectra of selected compounds in the solid state at RT; bottom right: emission spectra of selected compounds in frozen 2‐Me‐THF glassy matrices at 77 K. b) Top: photographic evidence of the AIE nature of **2y** in THF/water mixtures with increasing H_2_O content upon excitation with a UV‐lamp (*λ* = 365 ± 20 nm); bottom left: photoluminescence spectra of **2y** for increasing water content; bottom right: corresponding *Φ*_F_ values (*λ*_exc_ = 340 nm). c) Emission intensity of selected compounds for increasing water content. d) Top: photographic evidence of the AIE nature of **2k** in THF/water mixtures with increasing H_2_O content upon excitation with a UV‐lamp (*λ* = 365 ± 20 nm); bottom left: photoluminescence spectra of **2k** for increasing water content; bottom right: corresponding *Φ*_F_ values (*λ*_exc_ = 340 nm).

In this study, six representative exemplars were selected for in‐depth photophysical studies, namely **2a**, **2k**, **2l**, **2s**, **2y**, and **2aj**, as they display the highest *Φ*_F_ of each family of compounds. For these complexes, time‐resolved photoluminescence decays and the resulting excited state lifetimes (*τ*) at room temperature (RT) were measured. Moreover, *Φ*_F_ and *τ* were also evaluated in diluted frozen 2‐Me‐THF glassy matrices at 77 K (**Table** **2**). The emission spectra at RT and at 77 K are depicted in **Figure** **1**a.

**Table 2:** Photophysical properties of selected compounds including steady‐state photoluminescence quantum yields in frozen glassy matrices at 77 K and as solids at room temperature, as well as amplitude‐weighted average lifetimes at RT (solids) and at 77 K (frozen glassy matrices)

Compound	*Φ*_F(RT)_ ± 2 / [%]^a)^	*Φ*_F(77K)_ ± 3 / [%]^b)^	*τ*_av_amp (RT)_ / [ns]^a)^	*τ*_av_amp (77K)_ / [ns]^b)^
**2a**	48	91	3.53 ± 0.04	4.33 ± 0.03
**2k**	39	90	1.29 ± 0.05	3.92 ± 0.04
**2l**	50	94	2.80 ± 0.02	4.11 ± 0.02
**2s**	57	79	3.14 ± 0.04	4.51 ± 0.03
**2y**	44	75	3.42 ± 0.06	6.11 ± 0.07
**2aj**	15	90	2.54 ± 0.06	4.11 ± 0.04

^a)^ Solid state at RT;

^b)^ Frozen glassy matrix (2‐MeTHF) at 77K. The time‐resolved photoluminescence decay profiles are shown in Figures S3–S14, Supporting Information, including the individual components and their relative pre‐exponential amplitudes.

In glassy matrices at 77 K, the excited state lifetimes are longer than at RT and the *Φ*_F_ reach nearly 100%, as non‐radiative deactivation pathways caused by rotovibrational modes and interaction with the solvent are suppressed while the charge transfer states are destabilized. Therefore, the emission is originated by singlet excited states with higher *π*–π* character, as opposed to RT where the *n*–*π* ^∗ ^character is more pronounced with concomitantly lower oscillator strengths and broader emission bands. As shown in Figure 1a, significant red‐shifts are observed in the emission spectra (both at RT and 77 K), depending on the *π*‐electron‐donating ability of the substituents. Notably, these BF_2_ complexes display bright and intensive solid‐state fluorescence, varying from blue to yellow (Figure 1a).

However, when dissolved in THF (or in any fluid organic solvent), only very weak fluorescence intensities can be traced, whereas an intense solid‐state photoluminescence can be detected (Table 1, Figure 1a). This result encouraged us to investigate their AIE ability in THF/mixtures with increasing water content to promote aggregation. The resulting fluorescence spectra can be seen in Figures S15–S19, Supporting Information. In addition, *Φ*_F_ and *τ  *were also determined (see Table S3 and Figures S20–S34, Supporting Information, for the details). To highlight the emissive properties of these AIE chromophores, photoluminescence spectra and photographic documentation of the fluorescence evolution, are shown exemplarily for **2y** in Figure 1b, along with the enhancement of *Φ*_F_ for increasing H_2_O contents and progressive aggregation. As the solubility of the BF_2_ complexes is reduced, the formation of luminescent aggregates is promoted. Up to H_2_O fractions between 70 and 80%, the compounds showed little or no fluorescence at all (evidenced by the relatively weak intensity and low *Φ*_F_, see Figure 1b and Table S3, Supporting Information). When increasing the H_2_O fraction, the compounds start to aggregate, resulting in a drastic jump of *Φ*_F_ and photoluminescence intensity, as shown in Figure 1b and Figures S15–S19, Supporting Information. This trend was confirmed for all the evaluated compounds (Figure 1c), confirming that this new set of compounds represent a remarkable class of AIE chromophores. Interestingly, compound **2k** represents an exception from the typical AIE behavior, exhibiting a drop of efficiency above 80% of H_2_O, as evidenced by the reduction of the emission intensity and *Φ*_F_ (Figure 1c,d; Table S3, Supporting Information). This result can be explained by the structure of the molecule (Table 1). In this case, the two peripheral Br atoms can enhance the intersystem crossing rate, due to the heavy atom effect. It is clear from Figure 1d that this becomes more significant upon aggregation at higher H_2_O fractions, which forces the formation of increasingly compact aggregates; hence, on the one hand, the rotovibrational degrees of freedom are suppressed and the fluorescence becomes more competitive, while on the other side, the contact between the chromophoric centers and the heavy halogen atoms is enhanced to promote growingly fast intersystem crossing that effectively competes with the radiative decay. Thus, the emission intensity and the *Φ*_F_ reach a maximum and then suddenly drop.

In general, the enhancement of the photoluminescence *Φ*_F_ is accompanied by prolonged excited state lifetimes, pointing to reduced rotovibrational rate constants upon rigidification in frozen glassy matrices, solids, and aggregates.

### 2.5 X‐Ray Diffractometric Analysis of Single Crystals

**Figure 2:**
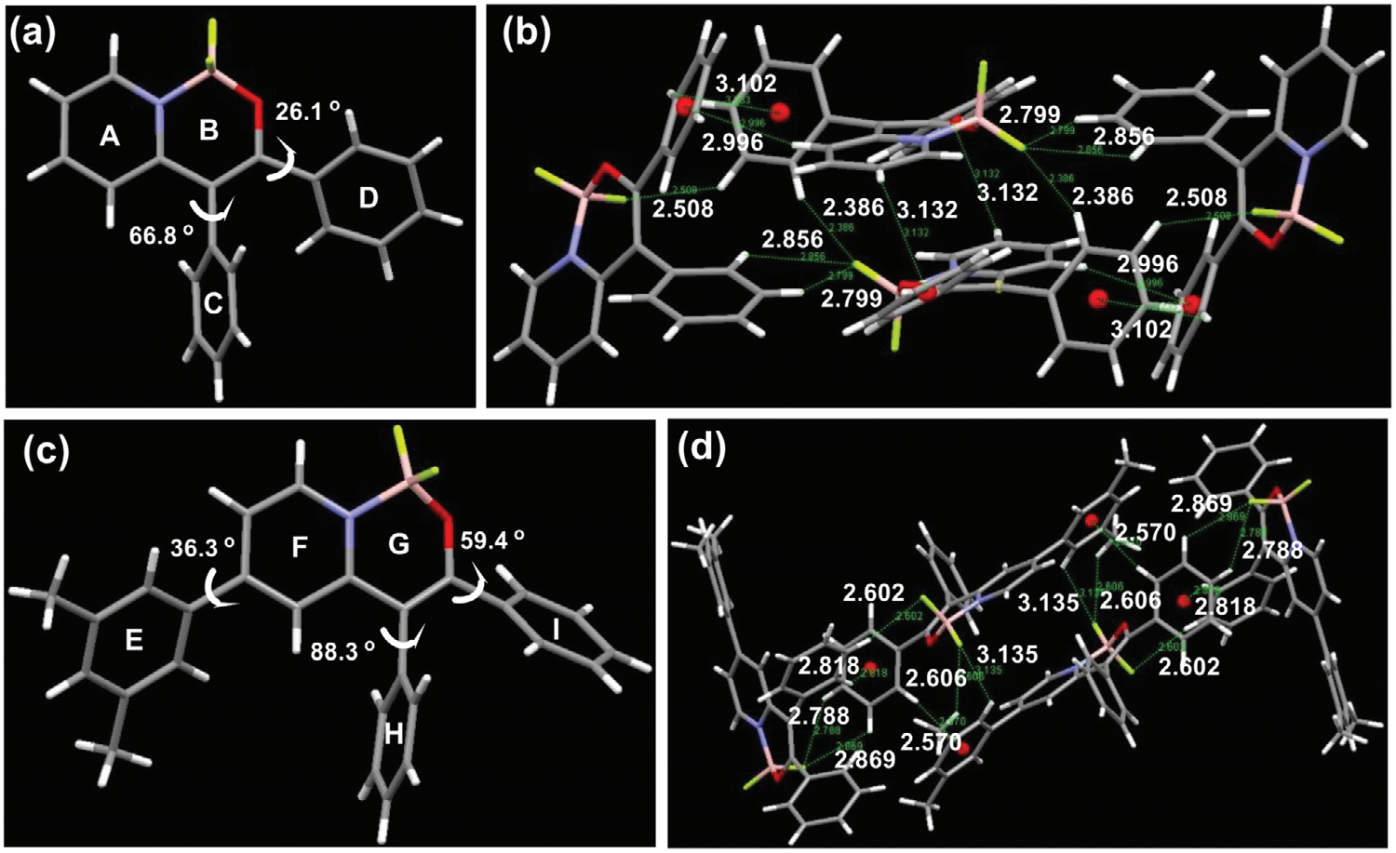
a) Molecular structure of **2a** in the crystal. b) Packing structure and multiple C—H⋅⋅⋅*π* and C—H⋅⋅⋅F interactions of **2a** in the crystalline phase. c) Molecular structure of **2ai** in the crystal. d) Packing structure and multiple C—H⋅⋅⋅*π* and C—H⋅⋅⋅F interactions of **2ai** in the crystalline phase.

To better understand the solid‐state emission properties of these BF_2_ complexes, X‐ray diffractometric analysis of single crystals from two selected compounds, namely **2a** and **2ai**, was performed.^[^ ^18^ ^]^ The conformation and packing patterns of **2a** and **2ai** were determined from crystalline phases, which were grown by using ethyl acetate as a solvent through slow evaporation at room temperature. As shown in **Figure** **2**a–c, compounds **2a** and **2ai** display twisted conformations and the phenyl rings (C and D, H and I) are not coplanar. The dihedral angles between rings B and C, rings B and D, rings E and F, rings G and H, and rings G and I are 66.8°, 26.1°, 36.3°, 88.3°, and 59.4° respectively. This twist enables the inhibition of intermolecular *π*–*π* stacking and thereby reduces the exciton quenching in the solid state, which would otherwise induce aggregation‐caused quenching (ACQ). As anticipated, no face‐to‐face intermolecular *π*–*π* stacking was found but multiple intermolecular interactions including C—H⋯*π* and C—H⋯F interactions were observed within the packing structures of **2a** and **2ai** (Figure 2b–d; see also Supporting Information for further details about the crystallographic analysis).^[^ ^19^ ^]^ These weak intermolecular interactions could rigidify their highly twisted conformation and impede intramolecular rotation, thus inhibiting their nonradiative relaxation. The above results can serve as a rationale for the intense fluorescence in the solid‐state.

### 2.6 DFT Computations

To further investigate the transitions involved in the absorption and emission of these AIE‐active BF_2_ complexes, density functional theory (DFT) computations were performed for a series of representative compounds (see Supporting information for the details). Geometry optimizations revealed that – in excellent agreement with the X‐ray crystallographic analysis – both phenyl rings are not coplanar with the central *π* system, but adopt a highly twisted conformation. As a result of this minimal conjugation, frontier molecular orbitals are mainly located on the central bicyclic core. Both the *π*‐type HOMO and the π*‐type LUMO are delocalized over this heterocyclic core, with only minor contributions from the aryl substituents. Time‐dependent DFT computations revealed that the experimentally observed absorption and emission bands can be assigned to spin‐allowed transitions between the ground state and the lowest excited singlet, where the latter can be described as a local *π*→π* monoelectronic excited configuration accessible *via* photoexcitation. This assignment applied to all investigated examples with different electronic properties, and the obtained trends in TD‐DFT excitation energies nicely correlate with the experimentally observed emission wavelengths.

## 3 Conclusion

In summary, we have demonstrated a novel strategy for the rapid construction of aggregation‐induced emission (AIE) active BF_2_ complexes with N,O‐bidentate ligands through a copper‐catalyzed cascade C—H activation/acetoxylation and difluoroboronation, which avoids the need for pre‐composing the N,O‐bidentate ligands and features a broad substrate scope and a high tolerance level for sensitive functional groups. This straightforward synthetic strategy enabled us to obtain a library of 36 compounds as a new class of BF_2_ complexes with N,O‐bidentate ligands in good to excellent yields. The photophysical properties of these compounds were studied in different conditions, such as solid state, frozen glassy matrices, in dilute solution, and under conditions that induce aggregation with concomitant AIE properties. We observed that depending on the substituent pattern, and consequently the *π*‐electron‐donating ability of the substituents, different emission maxima and quantum yields are obtained, depending principally on the *π*–π* character of the excited state configuration. X‐ray diffractometry and crystallographic analysis of selected BF_2_ complexes demonstrated that the weak intermolecular C—H⋯*π* and C—H⋯F interactions, which could rigidify their highly twisted conformation and impede intramolecular rotation, are responsible for intense luminescence in the solid state. Overall, a new series of photofunctional materials with variable photophysical properties is reported, with potential applications for sensing, bioimaging, and optoelectronics.

## Conflict of Interest

The authors declare no conflict of interest.

## Supporting information

Supporting Information

## Data Availability

The data that supports the findings of this study are available in the supplementary material of this article.
