# Supplementary material for: AIE‐Active Difluoroboron Complexes with N,O‐Bidentate Ligands: Rapid Construction by Copper‐Catalyzed C−H Activation
Source: Adv Sci (Weinh). 2021 Jul 26;8(18):2101814. doi: 10.1002/advs.202101814 (PMC8456238; doi:10.1002/advs.202101814)
Supplement: Supplementary file 1 — Supporting Information [file ADVS-8-2101814-s001.pdf]

© 2021 Wiley-VCH GmbH

Supporting Information

for *Adv. Sci.*, DOI: 10.1002/advs.202101814

AIE-Active Difluoroboron Complexes with N,O-Bidentate Ligands: Rapid Construction by Copper-Catalyzed C−H Activation

Guangying Tan[‡], Iván Maisuls[‡], Felix Strieth-Kalthoff, Xiaolong Zhang, Constantin Daniliuc, Cristian A. Strassert, and Frank Glorius**

Supporting Information

AIE-Active Difluoroboron Complexes with N,O-Bidentate Ligands: Rapid Construction by Copper-Catalyzed C−H Activation

Guangying Tan‡, Iván Maisuls‡, Felix Strieth-Kalthoff, Xiaolong Zhang, Constantin Daniliuc, Cristian A. Strassert, and Frank Glorius**

Table of Contents

I. General remarks..S2

II. Optimization of the reaction conditions……………………………..……..…S3

III. Sensitivity assessment…..…………………………………………..……..….S4

IV. General procedure for the synthesis of AIE-active BF_2 complexes…………......S5

V. Proposed mechanism……..……………………………………..……..…S5

VI. Scale-up to 10 mmol reaction of **1a**…………………………..…...……..…S6

VII. Experimental data for the described substances...S7

VIII. Photophysical properties of selected products..S31

IX. X-ray analysis………………………...S48

X. TDDFT calculation……………………..S53

XI. References...S69

XII. Copies of ^{1}H and ^{13}C NMR spectra...S70

I. General remarks

NMR spectra were obtained on a Bruker AV-400 MHz. The residual solvent signals were used as references for ^{1}H and ^{13}C NMR spectra ($CDCl_3$: δH = 7.26 ppm, δC = 77.16 ppm; 77.16 ppm; DMSO-d_6: δH = 2.50 ppm, δC = 39.52 ppm). High-resolution mass spectra (HRMS) were obtained with a Bruker Daltonics MicroTof spectrometer (ESI). Photoluminescence spectra spectra were recorded on a FluoTime300 spectrometer from PicoQuant equipped with a 300 W ozone-free lamp (290-900 nm). Photoexcited state lifetimes were recorded using the FluoTime 300 spectrometer with a diode laser (pulse width < 80 ps) (376,6 nm) operated by a computer-controlled laser driver PDL-820. Steady state and fluorescence lifetimes were recorded in TSCPC mode by a Picoharp 300 (minimum base resolution 4 ps). Solid state samples were measured using a sample holder equipped with an adjustable front-face sample holder. Photoluminescence quantum yields were measured with a Hamamatsu Photonics absolute PL quantum yield measurement system (C9920-02) and with an integrating sphere integrated in the Fluorotime 300 (Picoquant), where the results were identical within the experimental uncertainty. Lifetime analysis was performed using the commercial FluoFit software. The samples in fluid solutions were measured in quartz cuvettes whereas the frozen glassy matrices were analyzed employing a custom-made quartz Dewar filled with liquid nitrogen while being able to accommodate the custom-made Suprasil® quartz tubes containing the samples.

Commercially available chemicals were obtained from Acros Organics, Aldrich Chemical Co., Alfa Aesar, ABCR, and TCI Europe and used as received unless otherwise stated. Analytical thin layer chromatography (TLC) was performed on silica gel 60 F254 aluminum plates (Merck). TLC plates were visualized by exposure to short wave ultraviolet light (254 nm, 366 nm) and/or $KMnO_4$. Flash chromatography was performed on Merck silica gel (40-63 mesh) by standard techniques. Various (*E*)-2-(1,2-diarylvinyl)pyridines were prepared according to the literature procedures.[1] Toluene was purified and dried according to standard methods prior to use.

II. Optimization of the reaction conditions

A 10 mL Schlenk tube with a magnetic stir bar was charged with $Cu(BF_4)_2{\cdot}6H_2O$ (17.3 mg, 50 mol %), AcOH (18.0 mg, 0.3 mmol, 3.0 equiv), and (*E*)-2-(1,2-diphenylvinyl)pyridine **1a** (25.7 mg, 0.1 mmol, 1.0 equiv) in toluene (0.5 mL) under air atmosphere. The resulting mixture was stirred at 140 °C for 20 h and then diluted with 3 mL of dichloromethane. The mixture was filtered through a celite pad and washed with 10-20 mL of dichloromethane. The filtrate was concentrated, and the residue was purified by column chromatography on silica gel (pentane/EtOAc = 8/1, v/v) to provide **2a**.

Table S1: Optimization of the reaction.[a]

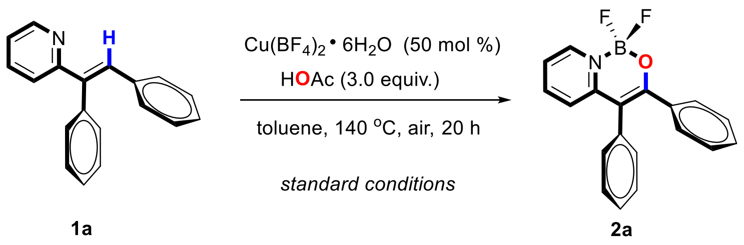

Entry	Variation	Yield [%][b]
1	None	95
2	Without $Cu(BF_4)_2{\cdot}6H_2O$	N.R.
3	Without HOAc	N.R.
4	Under argon atmosphere	34
5	Using 20 mol% of $Cu(BF_4)_2{\cdot}6H_2O$ as catalyst and 1.0 equiv of $NaBF_4$ as additive	83
6	Using 20 mol% of $Cu(OAc)_2$ as catalyst, 1.5 equiv of $AgSbF_6$ as oxidant and BF_2 source, and 1.5 equiv PivOH as oxygen atom source	36
7	Using 20 mol% of $Cu(OAc)_2$ as catalyst and 1.0 equiv of $NaBF_4$ as additive	72
8	100 °C	trace

[a] Standard conditions: (*E*)-2-(1,2-diphenylvinyl)pyridine **1a** (0.1 mmol), $Cu(BF_4)_2{\cdot}6H_2O$ (0.05 mmol, 50 mol%), and AcOH (0.3 mmol, 3.0 equiv) in toluene (0.5 mL, 0.2 M) at 140 °C under air atmosphere for 20 h. [b] Isolated yields.

III. Sensitivity assessment[2]

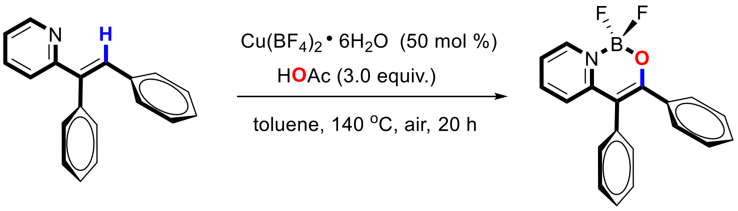

A 10 mL Schlenk tube with a magnetic stir bar was charged with $Cu(BF_4)_2{\cdot}6H_2O$ (17.3 mg, 50 mol %), AcOH (18.0 mg, 0.3 mmol, 3.0 equiv), and (*E*)-2-(1,2-diphenylvinyl)pyridine **1a** (25.7 mg, 0.1 mmol, 1.0 equiv) in toluene (0.5 mL) under air atmosphere. The resulting mixture was stirred at 140 °C for 20 h and then cooled to room temperature and diluted with $CDCl_3$. (Trifluoromethyl)benzene (14.6 mg, 0.1 mmol) was added as an internal standard and the mixture was filtered through a Celite plug and analyzed by ^{19}F NMR.

Big scale reaction:

A 200 mL Schlenk tube with a magnetic stir bar was charged with $Cu(BF_4)_2{\cdot}6H_2O$ (346.0 mg, 50 mol %), AcOH (360.0 mg, 6.0 mmol, 3.0 equiv), and (*E*)-2-(1,2-diphenylvinyl)pyridine **1a** (514.0 mg, 2.0 mmol, 1.0 equiv) in toluene (10.0 mL) under air atmosphere. The resulting mixture was stirred at 140 °C for 24 h. The reaction mixture was cooled to room temperature and diluted with $CDCl_3$. The yield of **2a** was determined by ^{19}F NMR analysis of the crude product using (trifluoromethyl)benzene (292.0 mg, 2.0 mmol) as internal standard.

Table S2. Result of sensitivity assessment

Entry	Experiment	Preparation	Yield (%)	Deviation (%)
1	high c	std. cond., V(toluene) = 0.45 mL	97	0
2	low c	std. cond., V(toluene) = 0.55 mL	97	0
3	high H_2O	std. cond., + 5 μL H_2O	94	–3
4	low O_2	std. cond., under argon atmosphere	36	–61
5	high O_2	std. cond., under O_2 atmosphere	98	1

6	high T	std. cond., T = 150 ℃	98	1
7	low T	std. cond., T = 130 ℃	67	–30
8	control	std. cond.	97	–
9	big scale	cond. of ‘big scale’	84	–13

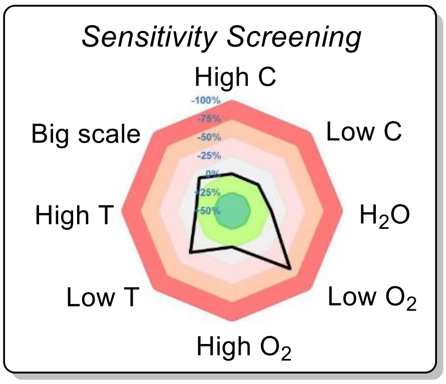

Figure S1. Result of sensitivity assessment.

IV. General procedure for the synthesis of AIE-active BF_2 complexes

A 10 mL Schlenk tube with a magnetic stir bar was charged with $Cu(BF_4)_2{\cdot}6H_2O$ (17.3 mg, 50 mol %), AcOH (18.0 mg, 0.3 mmol, 3.0 equiv), and (*E*)-2-(1,2-diarylvinyl)pyridines **1** (0.1 mmol, 1.0 equiv) in toluene (0.5 mL) under air atmosphere. The resulting mixture was stirred at 140 ^{o}C for 20 h and then diluted with 3 mL of dichloromethane. The mixture was filtered through a celite pad and washed with 10-20 mL of dichloromethane. The filtrate was concentrated, and the residue was purified by column chromatography on silica gel to provide the desired products **2**.

V. Proposed mechanism

We hypothesized that the formation of **2a** is unlocked through the copper-catalyzed pathway illustrated in Figure S2, which involves C−H activation/acyloxylation in tandem with difluoroboronation. In the C−H acyloxylation process, (*E*)-2-(1,2-diphenylvinyl)pyridine (**1a**) undergoes C−H activation to form intermediate **IM1**, oxidation to form Cu(III) intermediate **IM2**, and reductive elimination to form intermediate **IM3**, successively. Then, intermediate **IM3** can react

with $Cu(BF_4)_2$ through anion exchange to afford **IM4**, which decomposes to give intermediate **IM5** along with CuF due to its instability at high temperature. Subsequently, intermediate **IM5** can deliver the desired product **2a** by releasing a molecule of MeCOF, which is presumably quenched by copper and/or silver salts in the reaction system. Finally, the generated Cu(I) species is re-oxidized to the Cu(II) species by O_2 to close the catalytic cycle.

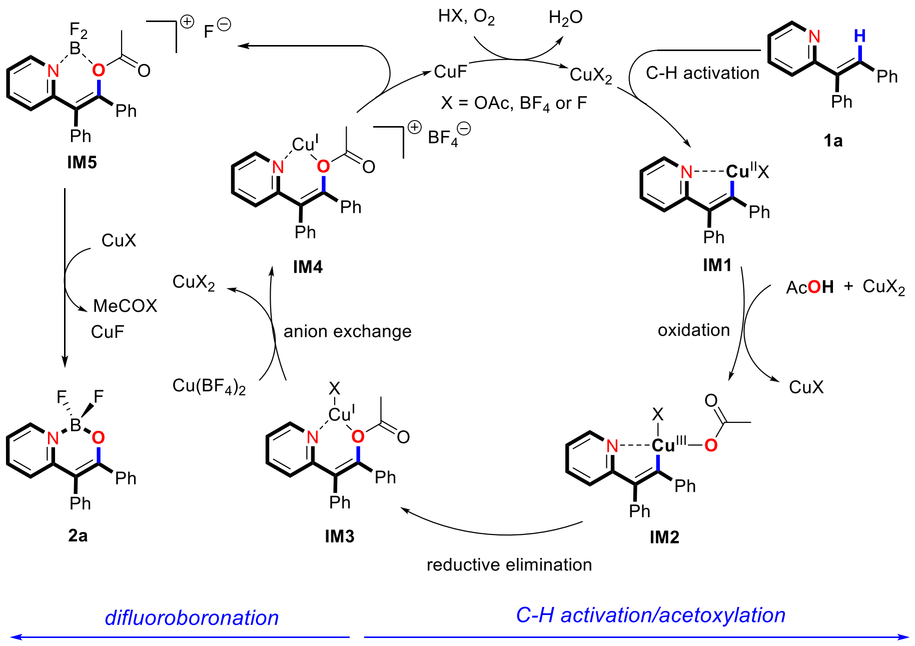

Figure S2. Proposed mechanism for the formation of **2a** via copper-catalyzed C–H activation.

VI. Scale-up to 10 mmol reaction of 1a

A 200 mL Schlenk tube with a magnetic stir bar was charged with $Cu(BF_4)_2{\cdot}6H_2O$ (1.73 g, 5.0 mmol, 50 mol %), AcOH (1.8 g, 30.0 mmol, 3.0 equiv), and (*E*)-2-(1,2-diphenylvinyl)pyridine **1a** (2.57 g, 2.0 mmol, 1.0 equiv) in toluene (20 mL) under O_2 (1 atm.) atmosphere. The resulting mixture was stirred at 140 ^{o}C for 36 h. The reaction mixture was cooled to room temperature and and then diluted with 30 mL of dichloromethane. The mixture was filtered through a celite pad and washed with 50-60 mL of dichloromethane. The filtrate was concentrated and the residue was purified by column chromatography on silica gel to provide the desired product **2a** in 72% yield (2.31 g).

VII. Experimental data for the described substances

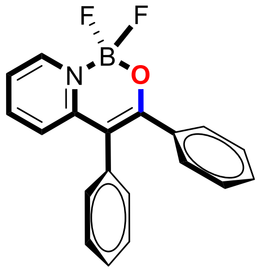

1,1-Difluoro-3,4-diphenyl-1*H*-1λ^4,9λ^4-pyrido[1,2-*c*][1,3,2]oxazaborinine (2a)

Following the general procedure. (*E*)-2-(1,2-Diphenylvinyl)pyridine **1a** (25.7 mg, 0.1 mmol) was used. Purification via column chromatography on silica gel (pentane/EtOAc = 8/1, v/v) afforded **2a** as a yellow solid (30 mg, 95% yield). ^{1}H NMR (600 MHz, $CDCl_3$): δ = 7.08-7.24 (m, 6H), 7.33-7.41 (m, 6H), 7.80-7.85 (m, 1H), 8.55 (d, J = 6.4 Hz, 1H) ppm. ^{13}C NMR (151 MHz, $CDCl_3$): δ = 109.2, 120.6, 122.1, 127.6, 128.1, 129.38, 129.44, 129.5, 132.4, 135.2, 135.9, 140.0, 141.3, 152.9, 160.9 ppm. HRMS (ESI): calcd for $C_{19}H_{14}BF_2NNaO$ $[M+Na]^+$ 344.1034, found 344.1034.

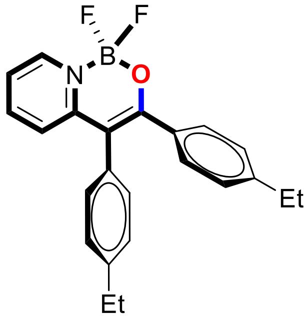

3,4-Bis(4-ethylphenyl)-1,1-difluoro-1*H*-1λ^4,9λ^4-pyrido[1,2-*c*][1,3,2]oxazaborinine (2b)

Following the general procedure. (*E*)-2-(1,2-Bis(4-ethylphenyl)vinyl)pyridine **1b** (31.3 mg, 0.1 mmol) was used. Purification via column chromatography on silica gel (pentane/EtOAc = 8/1, v/v) afforded **2b** as a yellow solid (36 mg, 96% yield). ^{1}H NMR (400 MHz, $CDCl_3$): δ = 1.16 (t, J = 7.6 Hz, 3H), 1.28 (t, J = 7.6 Hz, 3H), 2.56 (q, J = 7.6 Hz, 2H), 2.70 (q, J = 7.6 Hz, 2H), 6.96-6.99 (m, 2H), 7.07-7.14 (m, 3H), 7.21-7.23 (m, 2H), 7.27-7.32 (m, 3H), 7.77-7.81 (m, 1H), 8.52 (d, J = 6.0 Hz, 1H) ppm. ^{13}C NMR (101 MHz, $CDCl_3$): δ = 15.2, 15.6, 28.7, 108.6, 120.2, 122.1, 127.1,

129.0, 129.6, 132.2, 132.5, 133.3, 139.9, 141.1, 144.2, 145.8, 153.3, 160.7 ppm. HRMS (ESI): calcd for $C_{23}H_{23}BF_2NO$ $[M+H]^+$ 378.1841, found 378.1839.

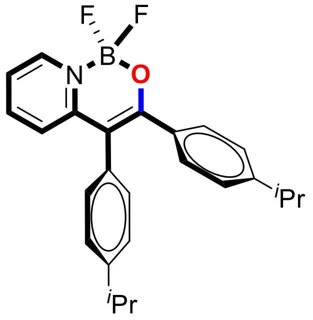

1,1-Difluoro-3,4-bis(4-isopropylphenyl)-1*H*-1λ^4,9λ^4-pyrido[1,2-*c*][1,3,2]oxazaborinine (2c)

Following the general procedure. (*E*)-2-(1,2-Bis(4-isopropylphenyl)vinyl)pyridine **1c** (34.1 mg, 0.1 mmol) was used. Purification via column chromatography on silica gel (pentane/EtOAc = 8/1, v/v) afforded **2c** as a yellow solid (36 mg, 90% yield). ^{1}H NMR (400 MHz, $CDCl_3$): δ = 1.19 (d, J = 7.2 Hz, 6H), 1.31 (d, J = 6.8 Hz, 6H), 2.78-2.88 (m, 1H), 2.92-3.05 (m, 1H), 6.99-7.02 (m, 2H), 7.10-7.16 (m, 3H), 7.24-7.36 (m, 5H), 7.79-7.83 (m, 1H), 8.54 (d, J = 6.0 Hz, 1H) ppm. ^{13}C NMR (101 MHz, $CDCl_3$): δ = 23.8, 24.1, 34.0, 108.7, 120.2, 122.2, 125.7, 127.5, 129.6, 132.2, 132.6, 133.4, 139.8, 141.0, 148.9, 150.4, 153.3, 160.6 ppm. HRMS (ESI): calcd for $C_{23}H_{23}BF_2NO$ $[M+H]^+$ 406.2154, found 406.2151.

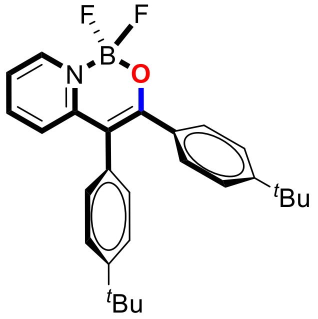

3,4-Bis(4-(*tert*-butyl)phenyl)-1,1-difluoro-1*H*-1λ^4,9λ^4-pyrido[1,2-*c*][1,3,2]oxazaborinine (2d)

Following the general procedure. (*E*)-2-(1,2-Bis(4-(*tert*-butyl)phenyl)vinyl)pyridine **1d** (36.9 mg, 0.1 mmol) was used. Purification via column chromatography on silica gel (pentane/EtOAc = 8/1, v/v) afforded **2d** as a yellow solid (40 mg, 92% yield). ^{1}H NMR (400 MHz, $CDCl_3$): δ = 1.24 (s, 9H), 1.36 (s, 9H), 7.08-7.16 (m, 5H), 7.27-7.32

(m, 3H), 7.40-7.43 (m, 2H), 7.77-7.82 (m, 1H), 8.52 (d, *J* = 6.0 Hz) ppm. ^{13}C NMR (101 MHz, $CDCl_3$): δ = 31.2, 31.5, 34.77, 34.79, 108.6, 120.2, 122.2, 124.5, 126.4, 129.4, 131.9, 132.3, 133.0, 139.8, 141.0, 151.2, 152.6, 153.2, 160.5 ppm. HRMS (ESI): calcd for $C_{27}H_{31}BF_2NO$ $[M+H]^+$ 434.2467, found 434.2466.

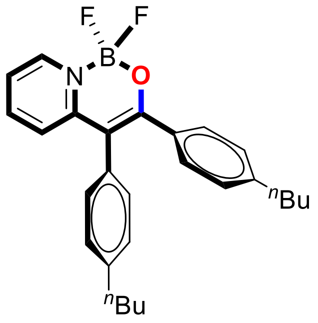

3,4-Bis(4-butylphenyl)-1,1-difluoro-1*H*-1λ^4,9λ^4-pyrido[1,2-*c*][1,3,2]oxazaborinine (2e)

Following the general procedure. (*E*)-2-(1,2-Bis(4-butylphenyl)vinyl)pyridine **1e** (36.9 mg, 0.1 mmol) was used. Purification via column chromatography on silica gel (pentane/EtOAc = 8/1, v/v) afforded **2e** as a yellow solid (41 mg, 93% yield). ^{1}H NMR (400 MHz, $CDCl_3$): δ = 0.88 (t, *J* = 7.6 Hz, 3H), 0.96 (t, *J* = 7.6 Hz, 3H), 1.24-1.42 (m, 4H), 1.48-1.56 (m, 2H), 1.60-1.68 (m, 2H), 2.52 (t, *J* = 8.0 Hz, 2H), 2.66 (t, *J* = 8.0 Hz, 2H), 6.93-96.95 (m, 2H), 7.09-7.12 (m, 3H), 7.19-7.21 (m, 2H), 7.25-7.32 (m, 3H), 7.77-7.81 (m, 1H), 8.52 (d, *J* = 6.0 Hz, 1H) ppm. ^{13}C NMR (101 MHz, $CDCl_3$): δ = 14.0, 14.1, 22.36, 22.40, 33.3, 33.6, 35.49, 35.52, 108.6, 120.2, 122.1, 127.6, 129.5, 129.6, 132.2, 132.5, 133.2, 139.9, 141.1, 142.8, 144.5, 153.2, 160.8 ppm. HRMS (ESI): calcd for $C_{27}H_{31}BF_2NO$ $[M+H]^+$ 434.2467, found 434.2460.

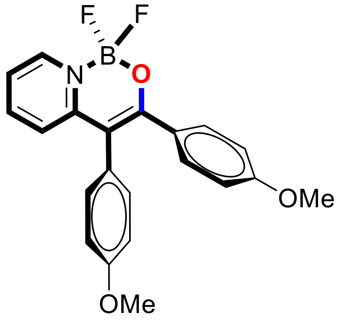

1,1-Difluoro-3,4-bis(4-methoxyphenyl)-1*H*-1λ^4,9λ^4-pyrido[1,2-*c*][1,3,2]oxazaborinine (2f)

Following the general procedure. (*E*)-2-(1,2-Bis(4-methoxyphenyl)vinyl)pyridine **1f** (31.7 mg, 0.1 mmol) was used. Purification via column chromatography on silica gel (pentane/EtOAc = 8/1, v/v) afforded **2f** as a yellow solid (33 mg, 86% yield). ^{1}H NMR (400 MHz, $CDCl_3$): δ = 3.76 (s, 3H), 3.85 (s, 3H), 6.66-6.70 (m, 2H), 6.89-6.96 (m, 2H), 7.06-7.15 (m, 3H), 7.27-7.38 (m, 3H), 7.76-7.80 (m, 1H), 8.50 (d, *J* = 6.0 Hz, 1H) ppm. ^{13}C NMR (101 MHz, $CDCl_3$): δ = 55.3, 55.4, 107.6, 113.1, 115.0, 120.0, 121.9, 127.6, 128.3, 131.4, 133.5, 139.8, 141.0, 153.4, 159.4, 160.5, 160.6 ppm. HRMS (ESI): calcd for $C_{21}H_{19}BF_2NO_3$ $[M+H]^+$ 382.1426, found 382.1424.

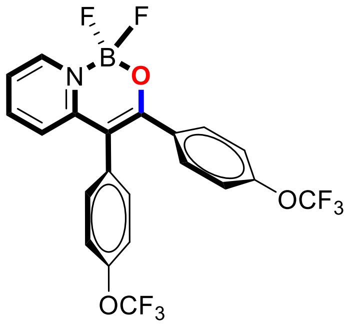

1,1-Difluoro-3,4-bis(4-(trifluoromethoxy)phenyl)-1*H*-1λ^4,9λ^4-pyrido[1,2-*c*][1,3,2] oxazaborinine (2g)

Following the general procedure. (*E*)-2-(1,2-Bis(4-(trifluoromethoxy)phenyl)vinyl)pyridine **1g** (42.5 mg, 0.1 mmol) was used. Purification via column chromatography on silica gel (pentane/EtOAc = 8/1, v/v) afforded **2g** as a yellow solid (45 mg, 92% yield). ^{1}H NMR (400 MHz, $CDCl_3$): δ = 6.99-7.02 (m, 2H), 7.08 (d, *J* = 8.4 Hz, 1H), 7.23-7.28 (m, 4H), 7.31-7.37 (m, 2H), 7.41-7.45 (m, 1H), 7.88-7.92 (m, 1H), 8.58 (d, *J* = 6.0 Hz, 1H) ppm. ^{13}C NMR (101 MHz, $CDCl_3$): δ = 108.2, 116.6 (d, *J* = 13.0 Hz), 119.2 (d, *J* = 13.0 Hz), 119.9 (d, *J* = 1.0 Hz), 121.3, 121.7 (d, *J* = 13.0 Hz), 121.9, 122.0, 124.3 (d, *J* = 12.0 Hz), 131.2, 133.5, 133.9, 134.1, 140.4, 141.8, 149.3 (q, *J* = 2.0 Hz), 149.8 (q, *J* = 2.0 Hz), 152.2, 159.8 ppm. HRMS (ESI): calcd for $C_{21}H_{13}BF_8NO_3$ $[M+H]^+$ 490.0861, found 490.0859.

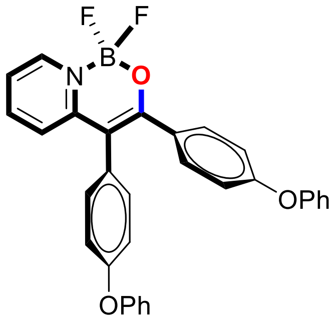

1,1-Difluoro-3,4-bis(4-phenoxyphenyl)-1*H*-1λ[4],9λ[4]-pyrido[1,2-*c*][1,3,2]oxazaborinine (2h)

Following the general procedure. (*E*)-2-(1,2-Bis(4-phenoxyphenyl)vinyl)pyridine **1h** (44.1 mg, 0.1 mmol) was used. Purification via column chromatography on silica gel (pentane/EtOAc = 8/1, v/v) afforded **2h** as a yellow solid (41 mg, 82% yield). ^{1}H NMR (400 MHz, $CDCl_3$): δ = 6.78-6.83 (m, 2H), 6.99-7.08 (m, 6H), 7.10-7.21 (m, 5H), 7.32-7.42 (m, 7H), 7.78-7.87 (m, 1H), 8.54-8.55 (m, 1H) ppm. ^{13}C NMR (101 MHz, $CDCl_3$): δ = 107.9, 117.2, 119.3, 119.6, 119.8, 120.4, 121.9, 123.9, 124.1, 129.9, 129.99, 130.01, 131.0, 131.4, 133.8, 140.0, 141.3, 153.0, 156.2, 156.7, 157.4, 158.6, 160.4 ppm. HRMS (ESI): calcd for $C_{31}H_{23}BF_2NO_3$ $[M+H]^+$ 506.1739, found 506.1739.

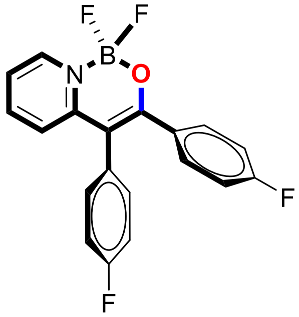

1,1-Difluoro-3,4-bis(4-fluorophenyl)-1*H*-1λ[4],9λ[4]-pyrido[1,2-*c*][1,3,2]oxazaborinine (2i)

Following the general procedure. (*E*)-2-(1,2-Bis(4-fluorophenyl)vinyl)pyridine **1i** (29.3 mg, 0.1 mmol) was used. Purification via column chromatography on silica gel (pentane/EtOAc = 8/1, v/v) afforded **2i** as a yellow solid (32 mg, 90% yield). ^{1}H NMR (400 MHz, $CDCl_3$): δ = 6.83-6.89 (m, 2H), 7.06-7.13 (m, 3H), 7.15-7.21 (m, 2H), 7.30-7.35 (m, 2H), 7.37-7.40 (m, 1H), 7.84-7.89 (m, 1H), 8.56 (d, J = 6.4 Hz, 1H) ppm. ^{13}C NMR (101 MHz, $CDCl_3$): δ = 107.9, 114.8 (d, J = 22.0 Hz) 116.7 (d, J =

22.0 Hz), 120.9, 121.8, 131.0 (d, *J* = 4.0 Hz), 131.6 (d, *J* = 8.0 Hz), 131.8, 134.1 (d, *J* = 8.0 Hz), 140.2, 141.6, 152.6, 160.2, 161.6 (d, *J* = 56.0 Hz), 164.1 (d, *J* = 58.0 Hz) ppm. HRMS (ESI): calcd for $C_{19}H_{12}BF_4NNaO$ $[M+Na]^+$ 380.0846, found 380.0841.

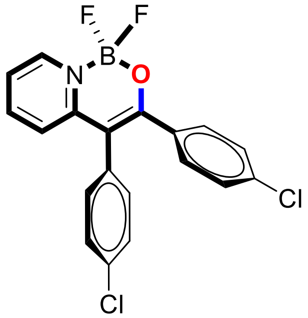

3,4-Bis(4-chlorophenyl)-1,1-difluoro-1*H*-1λ^4,9λ^4-pyrido[1,2-*c*][1,3,2]oxazaborinine (2j)

Following the general procedure. (*E*)-2-(1,2-Bis(4-chlorophenyl)vinyl)pyridine **1j** (32.5 mg, 0.1 mmol) was used. Purification via column chromatography on silica gel (pentane/EtOAc = 8/1, v/v) afforded **2j** as a yellow solid (38 mg, 97% yield). ^1H NMR (400 MHz, $CDCl_3$): δ = 7.06 (d, *J* = 8.4 Hz, 1H), 7.13-7.16 (m, 4H), 7.25-7.28 (m, 2H), 7.37-7.42 (m, 3H), 7.85-7.90 (m, 1H), 8.56 (d, *J* = 6.0 Hz, 1H) ppm. ^{13}C NMR (101 MHz, $CDCl_3$): δ = 108.2, 121.1, 121.9, 128.1, 130.0, 130.8, 133.4, 133.7, 134.1, 134.5, 135.7, 140.3, 141.7, 152.2, 159.9 ppm. HRMS (ESI): calcd for $C_{19}H_{12}BCl_2F_2NNaO$ $[M+Na]^+$ 412.0255, found 412.0249.

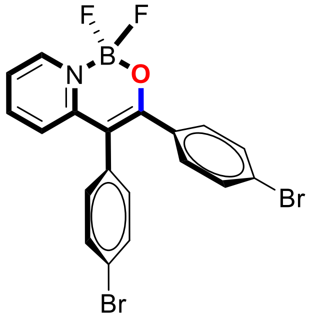

3,4-Bis(4-bromophenyl)-1,1-difluoro-1*H*-1λ^4,9λ^4-pyrido[1,2-*c*][1,3,2]oxazaborinine (2k)

Following the general procedure. (*E*)-2-(1,2-Bis(4-bromophenyl)vinyl)pyridine **1k** (41.3 mg, 0.1 mmol) was used. Purification via column chromatography on silica gel (pentane/EtOAc = 8/1, v/v) afforded **2k** as a yellow solid (45 mg, 95% yield). ^1H NMR (400 MHz, $CDCl_3$): δ = 7.05-7.10 (m, 3H), 7.18-7.21 (m, 2H), 7.30-7.34 (m,

2H), 7.39-7.42 (m, 1H), 7.52-7.56 (m, 2H), 7.85-7.90 (m, 1H), 8.56 (d, J = 6.0 Hz, 1H) ppm. ^{13}C NMR (101 MHz, $CDCl_3$): δ = 108.3, 121.2, 121.9, 122.7, 124.2, 131.0, 131.1, 132.9, 133.9, 134.0, 134.5, 140.3, 141.7, 152.2, 160.0 ppm. HRMS (ESI): calcd for $C_{19}H_{12}BBr_2F_2NNaO$ $[M+Na]^+$ 499.9244, found 499.9239.

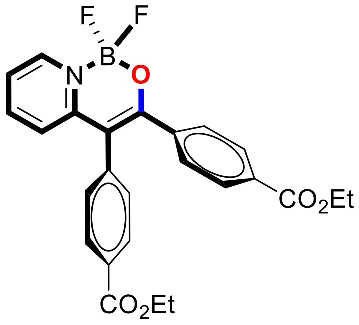

Diethyl 4,4'-(1,1-difluoro-1*H*-1λ^4,9λ^4-pyrido[1,2-*c*][1,3,2]oxazaborinine-3,4-diyl)dibenzoate (2l)

Following the general procedure. Diethyl 4,4'-(1-(Pyridin-2-yl)ethene-1,2-diyl)(*E*)-dibenzoate **1l** (40.1 mg, 0.1 mmol) was used. Purification via column chromatography on silica gel (pentane/EtOAc = 8/1, v/v) afforded **2l** as a yellow solid (42 mg, 91% yield). ^{1}H NMR (400 MHz, $CDCl_3$): δ = 1.35 (td, J = 7.2 Hz, 1.2 Hz, 3H), 1.40 (td, J = 7.2 Hz, 0.8 Hz, 3H), 4.29-4.35 (m, 2H), 4.37-4.42 (m, 2H), 7.06 (d, J = 8.8 Hz, 1H), 7.27-7.30 (m, 2H), 7.35-7.38 (m, 2H), 7.42-7.45 (m, 1H), 7.80-7.83 (m, 2H), 7.86-7.91 (m, 1H), 8.04-8.06 (m, 2H), 8.60 (d, J = 6.0 Hz, 1H) ppm. ^{13}C NMR (101 MHz, $CDCl_3$): δ = 14.37, 14.43, 61.2, 61.4, 109.3, 121.4, 121.9, 128.9, 129.4, 130.5, 130.7, 131.1, 132.4, 139.6, 139.8, 140.4, 141.8, 151.9, 160.2, 166.0, 166.1 ppm. HRMS (ESI): calcd for $C_{25}H_{22}BF_2NNaO_5$ $[M+Na]^+$ 488.1457, found 488.1451.

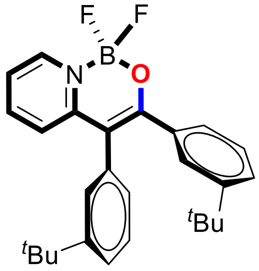

3,4-Bis(3-(*tert*-butyl)phenyl)-1,1-difluoro-1*H*-1λ^4,9λ^4-pyrido[1,2-*c*][1,3,2]oxazaborinine (2m)

Following the general procedure. (*E*)-2-(1,2-Bis(3-(*tert*-butyl)phenyl)vinyl)pyridine **1m** (36.9 mg, 0.1 mmol) was used. Purification via column chromatography on silica gel (pentane/EtOAc = 8/1, v/v) afforded **2m** as a yellow solid (41 mg, 94% yield). ^{1}H NMR (400 MHz, $CDCl_3$): δ = 1.03 (s, 9H), 1.21 (s, 9H), 7.03-7.05 (m, 1H), 7.10 (t, *J* = 2.0 Hz, 1H), 7.15-7.24 (m, 4H), 7.29-7.36 (m, 3H), 7.43-7.45 (m, 1H), 7.80-7.84 (m, 1H), 8.54-8.56 (m, 1H) ppm. ^{13}C NMR (101 MHz, $CDCl_3$): δ = 31.2, 31.4, 34.5, 34.7, 109.5, 120.4, 122.0, 124.7, 126.2, 126.3, 127.2, 127.6, 129.0, 129.1, 129.8, 135.1, 135.6, 139.9, 141.2, 149.8, 152.5, 153.0, 161.8 ppm. HRMS (ESI): calcd for $C_{27}H_{31}BF_2NO$ $[M+H]^+$ 434.2467, found 434.2466.

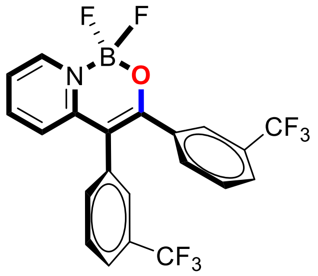

1,1-Difluoro-3,4-bis(3-(trifluoromethyl)phenyl)-1*H*-1λ^4,9λ^4-pyrido[1,2-*c*][1,3,2]oxazaborinine (2n)

Following the general procedure. (*E*)-2-(1,2-Bis(3-(trifluoromethyl)phenyl)vinyl)pyridine **1n** (39.3 mg, 0.1 mmol) was used. Purification via column chromatography on silica gel (pentane/EtOAc = 8/1, v/v) afforded **2n** as a yellow solid (39 mg, 85% yield). ^{1}H NMR (400 MHz, $CDCl_3$): δ = 7.08 (d, *J* = 8.4 Hz, 1H), 7.32 (t, *J* = 8.0 Hz, 1H), 7.40-7.57 (m, 7H), 7.65-7.68 (m, 1H), 7.92-7.97 (m, 1H), 8.62 (d, *J* = 6.0 Hz, 1H) ppm. ^{13}C NMR (101 MHz, $CDCl_3$): δ = 108.8, 121.6, 121.9, 123.7 (q, *J* = 271.0 Hz), 123.8 (q, *J* = 270.0 Hz), 125.4 (q, *J* = 4.0 Hz), 126.2 (q, *J* = 4.0 Hz), 126.4 (q, *J* = 4.0 Hz), 128.5, 129.1 (q, *J* = 4.0 Hz), 130.2 (q, *J* = 32.0 Hz), 130.3, 132.2 (q, *J* = 32.0 Hz), 132.5 (q, *J* = 1.0 Hz), 135.6, 135.7 (q, *J* = 1.0 Hz), 136.1, 140.6, 142.1, 151.8, 159.9 ppm. HRMS (ESI): calcd for $C_{21}H_{12}BF_8NNaO$ $[M+Na]^+$ 480.0782, found 480.0777.

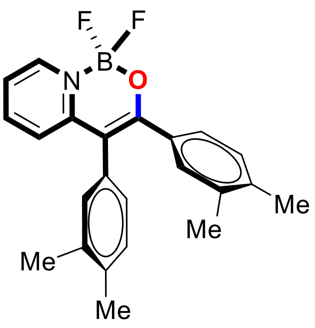

3,4-Bis(3,4-dimethylphenyl)-1,1-difluoro-1*H*-1λ^4,9λ^4-pyrido[1,2-*c*][1,3,2]oxazaborinine (2o)

Following the general procedure. (*E*)-2-(1,2-Bis(3,4-dimethylphenyl)vinyl)pyridine **1o** (31.8 mg, 0.1 mmol) was used. Purification via column chromatography on silica gel (pentane/EtOAc = 8/1, v/v) afforded **2o** as a yellow solid (30 mg, 80% yield). ^{1}H NMR (400 MHz, $CDCl_3$): δ = 2.13 (s, 3H), 2.17 (s, 3H), 2.24 (s, 3H), 2.30 (s, 3H), 6.84 (d. J = 8.0 Hz, 1H), 6.91-6.98 (m, 2H), 7.00 (d, J = 1.6 Hz, 1H), 7.07 (d, J = 8.8 Hz, 1H), 7.14 (d, J = 7.6 Hz, 1H), 7.27-7.31 (m, 1H), 7.33 (d, J = 2.0 Hz, 1H), 7.75-7.79 (m, 1H), 8.51 (d, J = 6.0 Hz, 1H) ppm. ^{13}C NMR (101 MHz, $CDCl_3$): δ = 19.7, 19.8, 19.9, 108.7, 120.1, 122.2, 127.3, 128.7, 129.7, 130.7, 132.8, 133.3, 135.9, 136.4, 137.7, 138.3, 139.7, 140.9, 153.4, 160.6 ppm. HRMS (ESI): calcd for $C_{23}H_{23}BF_2NO$ $[M+H]^+$ 378.1841, found 378.1839.

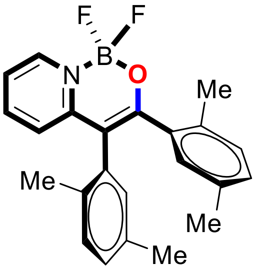

3,4-Bis(2,5-dimethylphenyl)-1,1-difluoro-1*H*-1λ^4,9λ^4-pyrido[1,2-*c*][1,3,2]oxazaborinine (2p)

Following the general procedure. (*E*)-2-(1,2-Bis(2,5-dimethylphenyl)vinyl)pyridine **1p** (31.8 mg, 0.2 mmol) was used. Purification via column chromatography on silica gel (pentane/EtOAc = 8/1, v/v) afforded **2p** as a yellow solid (29 mg, 79% yield). ^{1}H NMR (400 MHz, $CDCl_3$): δ = 2.05 (s, 3H), 2.09 (s, 3H), 2.21 (s, 3H), 2.38 (s, 3H), 6.84-6.91 (m, 4H), 6.95-6.98 (m, 2H), 7.02 (d, J = 8.0 Hz, 1H), 7.34-7.38 (m, 1H), 7.80-7.85 (m, 1H), 8.56 (d, J = 6.0 Hz, 1H) ppm. ^{13}C NMR (101 MHz, $CDCl_3$): δ =

19.3, 19.4, 20.8, 20.9, 109.3, 120.7, 121.8, 129.0, 129.5, 129.6, 130.1, 130.3, 133.2, 133.4, 133.5, 133.9, 135.1, 135.4, 135.5, 140.1, 141.5, 152.3, 163.8 ppm. HRMS (ESI): calcd for $C_{23}H_{22}BF_2NNaO$ $[M+Na]^+$ 400.1660, found 400.1652.

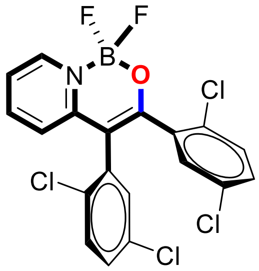

3,4-Bis(2,5-dichlorophenyl)-1,1-difluoro-1*H*-1λ^4,9λ^4-pyrido[1,2-*c*][1,3,2]oxazaborinine (2q)

Following the general procedure. (*E*)-2-(1,2-Bis(2,5-dichlorophenyl)vinyl)pyridine **1q** (39.2 mg, 0.1 mmol) was used. Purification via column chromatography on silica gel (pentane/EtOAc = 8/1, v/v) afforded **2q** as a yellow solid (39 mg, 86% yield). ^{1}H NMR (400 MHz, $CDCl_3$): δ = 6.88 (d, J = 8.4 Hz, 1H), 7.17-7.20 (m, 1H), 7.23-7.30 (m, 4H), 7.36-7.38 (m, 1H), 7.49-7.53 (m, 1H), 7.95-8.00 (m, 1H), 8.65 (d, J = 6.0 Hz, 1H) ppm. ^{13}C NMR (101 MHz, $CDCl_3$): δ = 108.0, 121.6, 122.0, 129.4, 130.5, 130.7, 130.9, 131.0, 131.1, 132.3, 133.2, 134.1, 134.4, 136.2, 140.9, 142.3, 150.3, 160.3 ppm. HRMS (ESI): calcd for $C_{19}H_{10}BCl_4F_2NNaO$ $[M+Na]^+$ 481.9446, found 481.9440.

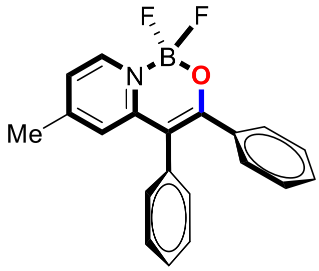

1,1-Difluoro-6-methyl-3,4-diphenyl-1*H*-1λ^4,9λ^4-pyrido[1,2-*c*][1,3,2]oxazaborinine (2r)

Following the general procedure. (*E*)-2-(1,2-Diphenylvinyl)-4-methylpyridine **1r** (27.1 mg, 0.1 mmol) was used. Purification via column chromatography on silica gel (pentane/EtOAc = 8/1, v/v) afforded **2r** as a yellow solid (29 mg, 86% yield). ^{1}H NMR (400 MHz, $CDCl_3$): δ = 2.35 (s, 3H), 6.85 (s, 1H), 7.11-7.23 (m, 6H), 7.30-7.41 (m, 5H), 8.41 (d, J = 6.4 Hz, 1H) ppm. ^{13}C NMR (101 MHz, $CDCl_3$): δ = 22.1, 108.9,

121.8, 120.0, 127.6, 128.0, 129.2, 129.4, 129.5, 132.4, 135.4, 136.1, 139.5, 154.1, 160.6 ppm. HRMS (ESI): calcd for $C_{20}H_{16}BF_2NNaO$ $[M+Na]^+$ 358.1191, found 358.1185.

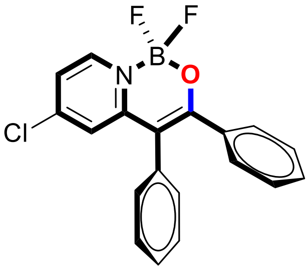

6-Chloro-1,1-difluoro-3,4-diphenyl-1*H*-1λ^4,9λ^4-pyrido[1,2-*c*][1,3,2]oxazaborinine (2s)

Following the general procedure. (*E*)-4-Chloro-2-(1,2-diphenylvinyl)pyridine **1s** (29.1 mg, 0.1 mmol) was used. Purification via column chromatography on silica gel (pentane/EtOAc = 8/1, v/v) afforded **2s** as a yellow solid (32 mg, 92% yield). ^{1}H NMR (400 MHz, $CDCl_3$): δ = 7.04 (d, J = 2.0 Hz, 1H), 7.13-7.17 (m, 2H), 7.18-7.25 (m, 3H), 7.30-7.36 (m, 3H), 7.38-7.43 (m, 3H), 8.44 (d, J = 6.4 Hz, 1H) ppm. ^{13}C NMR (101 MHz, $CDCl_3$): δ = 108.6, 121.2, 121.5, 127.7, 128.5, 129.5, 129.6, 129.7, 132.3, 134.5, 135.6, 141.1, 149.8, 153.9, 162.6 ppm. HRMS (ESI): calcd for $C_{19}H_{13}BClF_2NNaO$ $[M+Na]^+$ 378.0644 found 378.0640.

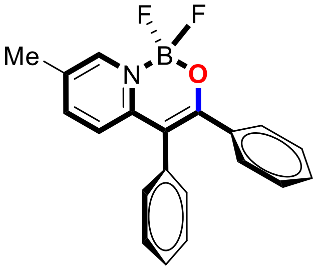

1,1-Difluoro-7-methyl-3,4-diphenyl-1*H*-1λ^4,9λ^4-pyrido[1,2-*c*][1,3,2]oxazaborinine (2t)

Following the general procedure. (*E*)-2-(1,2-Diphenylvinyl)-5-methylpyridine **1t** (27.1 mg, 0.1 mmol) was used. Purification via column chromatography on silica gel (pentane/EtOAc = 8/1, v/v) afforded **2t** as a yellow solid (30 mg, 90% yield). ^{1}H NMR (400 MHz, $CDCl_3$): δ = 2.42 (s, 3H), 7.00 (d, J = 8.4 Hz, 1H), 7.11-7.16 (m, 2H), 7.18-7.22 (m, 3H), 7.32-7.40 (m, 5H), 7.63-7.66 (m, 1H), 8.39 (s, 1H) ppm. ^{13}C

NMR (101 MHz, $CDCl_3$): δ = 18.2, 109.0, 121.8, 127.6, 128.0, 129.2, 129.4, 129.5, 131.2, 132.4, 135.4, 136.0, 139.2, 142.6, 150.5, 159.6 ppm. HRMS (ESI): calcd for $C_{20}H_{16}BF_2NNaO$ $[M+Na]^+$ 358.1191, found 358.1185.

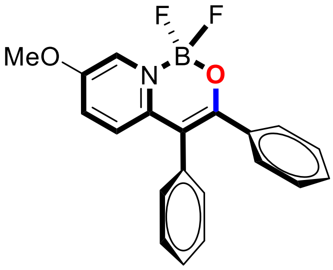

1,1-Difluoro-7-methoxyl-3,4-diphenyl-1*H*-1λ^4,9λ^4-pyrido[1,2-*c*][1,3,2]oxazaborinine (2u)

Following the general procedure. (*E*)-2-(1,2-Diphenylvinyl)-5-methoxylpyridine **1u** (28.7 mg, 0.1 mmol) was used. Purification via column chromatography on silica gel (pentane/EtOAc = 8/1, v/v) afforded **2u** as a yellow solid (28 mg, 79% yield). ^{1}H NMR (400 MHz, $CDCl_3$): δ = 3.94 (s, 3H), 7.03 (d, *J* = 9.2 Hz, 1H), 7.11-7.22 (m, 5H), 7.32-7.40 (m, 5H), 7.44 (dd, *J* = 9.2 Hz, 3.2 Hz, 1H), 8.16 (d, *J* = 2.8 Hz, 1H) ppm. ^{13}C NMR (101 MHz, $CDCl_3$): δ = 56.6, 109.0, 123.3, 123.9, 127.6, 128.1, 129.0, 129.4, 130.4, 132.3, 135.5, 136.0, 147.1, 154.2, 158.2 ppm. HRMS (ESI): calcd for $C_{20}H_{16}BF_2NNaO_2$ $[M+Na]^+$ 374.1140 found 374.1134.

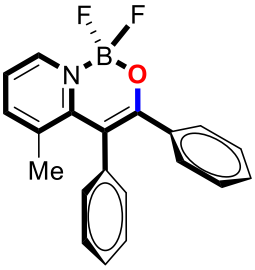

1,1-Difluoro-5-methyl-3,4-diphenyl-1*H*-1λ^4,9λ^4-pyrido[1,2-*c*][1,3,2]oxazaborinine (2v)

Following the general procedure. (*E*)-2-(1,2-Diphenylvinyl)-3-methylpyridine **1v** (27.1 mg, 0.1 mmol) was used. Purification via column chromatography on silica gel (pentane/EtOAc = 8/1, v/v) afforded **2v** as a yellow solid (21 mg, 63% yield). ^{1}H NMR (400 MHz, $CDCl_3$): δ = 1.66 (s, 3H), 7.10-7.28 (m, 10H), 7.32-7.35 (m, 1H), 7.68-7.70 (m, 1H), 8.53 (d, *J* = 6.0 Hz, 1H) ppm. ^{13}C NMR (101 MHz, $CDCl_3$): δ =

22.6, 111.6, 121.1, 127.6, 127.7, 128.5. 128.6, 129.0, 132.6, 133.3, 136.8, 137.1, 138.4, 145.4, 150.6, 162.5 ppm. HRMS (ESI): calcd for $C_{20}H_{16}BF_2NNaO$ $[M+Na]^+$ 358.1191, found 358.1185.

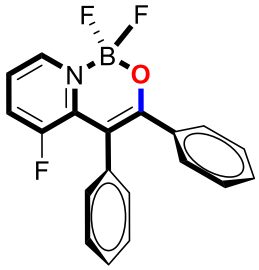

1,1,5-Trifluoro-3,4-diphenyl-1*H*-1λ^4,9λ^4-pyrido[1,2-*c*][1,3,2]oxazaborinine (2w)

Following the general procedure. (*E*)-2-(1,2-Diphenylvinyl)-3-fluoropyridine **1w** (27.5 mg, 0.1 mmol) was used. Purification via column chromatography on silica gel (pentane/EtOAc = 8/1, v/v) afforded **2w** as a yellow solid (26 mg, 76% yield). ^{1}H NMR (400 MHz, $CDCl_3$): δ = 7.12-7.17 (m, 2H), 7.19-7.24 (m, 3H), 7.25-7.32 (m, 5H), 7.36-7.41 (m, 1H), 7.57-7.62 (m, 1H), 8.46 (d, *J* = 5.6 Hz, 1H) ppm. ^{13}C NMR (101 MHz, $CDCl_3$): δ = 106.9, 121.3 (d, *J* = 6.0 Hz), 127.6, 127.7, 128.3, 128.5 (d, *J* = 20.0 Hz), 129.3, 129.4, 131.8 (d, *J* = 4.0 Hz), 135.6 (d, *J* = 4.0 Hz), 135.9, 136.6, 143.1 (d, *J* = 13.0 Hz), 155.4 (d, *J* = 259.0 Hz), 163.4 ppm. HRMS (ESI): calcd for $C_{19}H_{13}BF_3NNaO$ $[M+Na]^+$ 362.0940 found 362.0934.

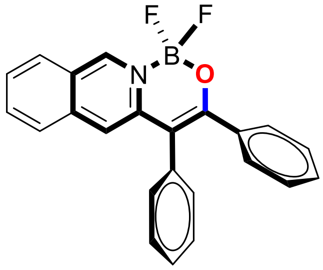

1,1-Difluoro-3,4-diphenyl-1*H*-1λ^4,11λ^4-[1,3,2]oxazaborinino[3,4-*b*]isoquinoline (2x)

Following the general procedure. (*E*)-3-(1,2-Diphenylvinyl)isoquinoline **1x** (30.7 mg, 0.1 mmol) was used. Purification via column chromatography on silica gel (pentane/EtOAc = 8/1, v/v) afforded **2x** as a yellow solid (27 mg, 72% yield). ^{1}H NMR (400 MHz, $CDCl_3$): δ = 7.12-7.22 (m, 3H), 7.28-7.32 (m, 2H), 7.36-7.39 (m, 3H), 7.40-7.45 (m, 3H), 7.61-7.65 (m, 1H), 7.68-7.70 (m, 1H), 7.77-7.81 (m, 1H),

8.09 (d, J = 8.4 Hz, 1.2 Hz, 1H), 9.40 (s, 1H) ppm. ^{13}C NMR (101 MHz, $CDCl_3$): δ = 109.6, 118.3, 125.6, 127.0, 127.6, 128.0, 128.4, 128.8, 129.4, 129.5, 132.4, 134.6, 136.0, 136.4, 138.7, 145.0, 145.7, 157.1 ppm. HRMS (ESI): calcd for $C_{23}H_{16}BF_2NNaO$ $[M+Na]^+$ 394.1191 found 394.1186.

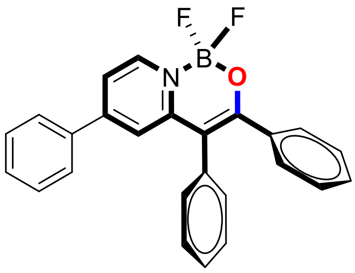

1,1-Difluoro-3,4,6-triphenyl-1*H*-1λ^4,9λ^4-pyrido[1,2-*c*][1,3,2]oxazaborinine (2y)

Following the general procedure. (*E*)-2-(1,2-Diphenylvinyl)-4-phenylpyridine **1y** (33.3 mg, 0.1 mmol) was used. Purification via column chromatography on silica gel (pentane/EtOAc = 8/1, v/v) afforded **2y** as a yellow solid (38 mg, 96% yield). ^{1}H NMR (400 MHz, $CDCl_3$): δ = 7.12-7.16 (m, 2H), 7.17-7.27 (m, 4H), 7.34-7.41 (m, 5H), 7.42-7.50 (m, 5H), 7.54 (dd, J = 6.4 Hz, 2.0 Hz, 1H), 8.57 (d, J = 6.4 Hz, 1H) ppm. ^{13}C NMR (101 MHz, $CDCl_3$): δ = 109.4, 118.8, 119.2, 127.3, 127.6, 128.2, 129.3, 129.5, 129.6, 130.8, 132.4, 135.3, 136.0, 136.3, 140.3, 152.9, 153.6, 161.0 ppm. HRMS (ESI): calcd for $C_{25}H_{18}BF_2NNaO$ $[M+Na]^+$ 420.1347 found 420.1341.

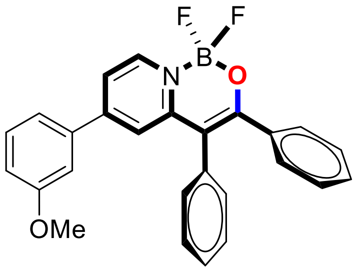

1,1-Difluoro-6-(3-methoxyphenyl)-3,4-diphenyl-1*H*-1λ^4,9λ^4-pyrido[1,2-*c*][1,3,2]oxazaborinine (2z)

Following the general procedure. (*E*)-2-(1,2-Diphenylvinyl)-4-(3-methoxyphenyl)pyridine **1z** (36.3 mg, 0.1 mmol) was used. Purification via column chromatography on silica gel (pentane/EtOAc = 8/1, v/v) afforded **2z** as a yellow solid (38 mg, 89% yield). ^{1}H NMR (400 MHz, $CDCl_3$): δ = 3.82 (s, 3H), 6.98-7.07 (m, 3H), 7.13-7.18 (m, 2H), 7.20-7.27 (m, 4H), 7.35-7.42 (m,

6H), 7.52-7.54 (m, 1H), 8.57 (d, J = 6.4 Hz, 1H) ppm. ^{13}C NMR (101 MHz, $CDCl_3$): δ = 55.5, 109.4, 113.3, 115.8, 118.9, 119.3, 119.7, 127.6, 128.2, 129.3, 129.4, 129.6, 130.7, 132.4, 135.3, 136.0, 137.8, 140.2, 152.9, 153.5, 160.3, 161.0 ppm. HRMS (ESI): calcd for $C_{26}H_{20}BF_2NNaO_2$ $[M+Na]^+$ 450.1453 found 450.1450.

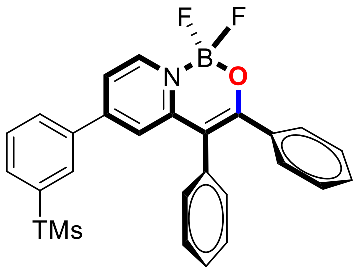

1,1-Difluoro-3,4-diphenyl-6-(3-(trimethylsilyl)phenyl)-1*H*-1λ^4,9λ^4-pyrido[1,2-*c*][1,3,2]oxazaborinine (2aa)

Following the general procedure. (*E*)-2-(1,2-Diphenylvinyl)-4-(3-(trimethylsilyl)phenyl)pyridine **1aa** (40.5 mg, 0.1 mmol) was used. Purification via column chromatography on silica gel (pentane/EtOAc = 8/1, v/v) afforded **2aa** as a yellow solid (44 mg, 93% yield). ^{1}H NMR (400 MHz, $CDCl_3$): δ = 0.24-0.25 (m, 9H), 7.11-7.15 (m, 2H), 7.18-7.25 (m, 4H), 7.31-7.46 (m, 7H), 7.52-7.62 (m, 1H), 8.56 (dd, J = 6.4 Hz, 2.0 Hz, 1H) ppm. ^{13}C NMR (101 MHz, $CDCl_3$): δ = 1.20, 109.4, 118.8, 119.5, 127.6, 127.7, 128.1, 128.9, 129.3, 129.4, 129.6, 132.2, 132.4, 135.4, 135.6, 135.7, 136.0, 140.2, 142.5, 152.9, 153.9, 160.8 ppm. HRMS (ESI): calcd for $C_{28}H_{26}BF_2NNaOSi$ $[M+Na]^+$ 492.1742 found 492.1739.

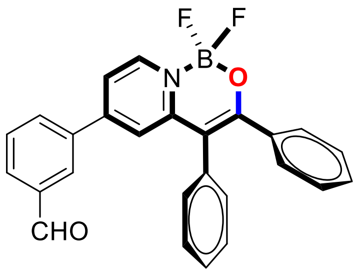

3-(1,1-Difluoro-3,4-diphenyl-1*H*-1λ^4,9λ^4-pyrido[1,2-*c*][1,3,2]oxazaborinin-6-yl)benzaldehyde (2ab)

Following the general procedure. (*E*)-3-(2-(1,2-Diphenylvinyl)pyridin-4-yl)benzaldehyde **1ab** (36.1 mg, 0.1 mmol) was

used. Purification via column chromatography on silica gel (pentane/EtOAc = 8/1, v/v) afforded **2ab** as a yellow solid (34 mg, 80% yield). ^{1}H NMR (400 MHz, $CDCl_3$): δ = 7.13-7.17 (m, 2H), 7.20-7.28 (m, 4H), 7.34-7.43 (m, 5H), 7.59 (dd, J = 6.4 Hz, 2.0 Hz, 1H), 7.65 (t, J = 7.6 Hz, 1H), 7.70-7.73 (m, 1H), 7.97-8.00 (m, 2H), 8.62 (d, J = 6.4 Hz, 1H), 10.05 (s, 1H) ppm. ^{13}C NMR (101 MHz, $CDCl_3$): δ = 109.3, 118.8, 119.4, 127.6, 128.0, 128.3, 129.5, 129.56, 129.57, 130.4, 131.8, 132.4, 132.9, 135.1, 135.9, 137.3, 137.5, 140.6, 152.2, 153.2, 161.7, 191.3 ppm. HRMS (ESI): calcd for $C_{26}H_{18}BF_2NNaO_2$ $[M+Na]^+$ 448.1296 found 448.1292.

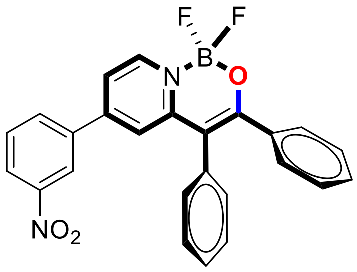

1,1-Difluoro-6-(3-nitrophenyl)-3,4-diphenyl-1*H*-1λ^4,9λ^4-pyrido[1,2-*c*][1,3,2]oxaza borinine (2ac)

Following the general procedure. (*E*)-2-(1,2-Diphenylvinyl)-4-(3-nitrophenyl)pyridine **1ac** (37.8 mg, 0.1 mmol) was used. Purification via column chromatography on silica gel (pentane/EtOAc = 8/1, v/v) afforded **2ac** as a yellow solid (38 mg, 87% yield). ^{1}H NMR (400 MHz, $CDCl_3$): δ = 7.14 (t, J = 7.6 Hz, 2H), 7.20-7.30 (m, 4H), 7.32-7.36 (m, 2H), 7.37-7.44 (m, 3H), 7.56-7.58 (m, 1H), 7.66 (t, J = 8.0 Hz, 1H), 7.76-7.79 (m, 1H), 8.30-8.34 (m, 2H), 8.62 (d, J = 6.4 Hz, 1H) ppm. ^{13}C NMR (101 MHz, $CDCl_3$): δ = 109.3, 118.6, 119.5, 122.3, 125.1, 127.7, 128.4, 129.55, 129.59, 129.64, 130.8, 132.3, 133.1, 134.9, 135.8, 138.1, 140.8, 148.9, 151.0, 153.4, 162.0 ppm. HRMS (ESI): calcd for $C_{25}H_{17}BF_2N_2NaO_3$ $[M+Na]^+$ 465.1198 found 465.1197.

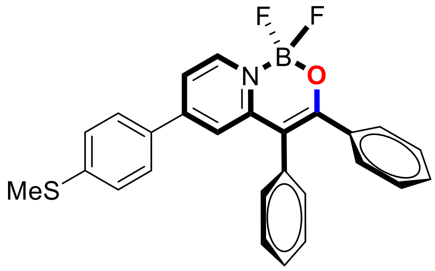

1,1-Difluoro-6-(4-(methylthio)phenyl)-3,4-diphenyl-1*H*-1λ^4,9λ^4-pyrido[1,2-*c*][1,3,

2]oxazaborinine (2ad)

Following the general procedure. (*E*)-2-(1,2-Diphenylvinyl)-4-(4-(methylthio)phenyl)pyridine **1ad** (37.9 mg, 0.1 mmol) was used. Purification via column chromatography on silica gel (pentane/EtOAc = 8/1, v/v) afforded **2ad** as a yellow solid (35 mg, 80% yield). ^{1}H NMR (400 MHz, $CDCl_3$): δ = 2.49 (s, 3H), 7.12-7.17 (m, 2H), 7.19-7.29 (m, 6H), 7.34-7.42 (m, 7H), 7.51 (dd, J = 4.4 Hz, 1H), 8.54 (d, J = 6.4 Hz, 1H) ppm. ^{13}C NMR (101 MHz, $CDCl_3$): δ = 15.2, 109.3, 118.2, 118.5, 126.5, 127.5, 127.6, 128.2, 129.3, 129.4, 129.6, 132.2, 132.4, 135.3, 136.1, 140.2, 143.2, 152.8, 152.9, 161.0 ppm. HRMS (ESI): calcd for $C_{26}H_{20}BF_2NNaOS$ $[M+Na]^+$ 466.1224 found 466.1219.

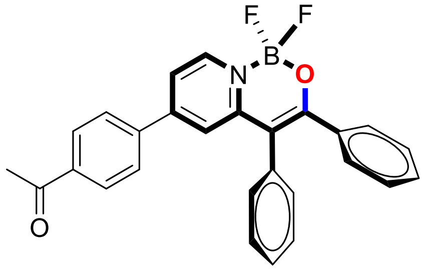

1-(4-(1,1-Difluoro-3,4-diphenyl-1*H*-1λ^4,9λ^4-pyrido[1,2-*c*][1,3,2]oxazaborinin-6-yl) phenyl)ethan-1-one (2ae)

Following the general procedure. (*E*)-1-(4-(2-(1,2-Diphenylvinyl)pyridin-4-yl)phenyl)ethan-1-one **1ae** (37.5 mg, 0.1 mmol) was used. Purification via column chromatography on silica gel (pentane/EtOAc = 8/1, v/v) afforded **2ae** as a yellow solid (28 mg, 64% yield). ^{1}H NMR (400 MHz, $CDCl_3$): δ = 2.62 (s, 3H), 7.14-7.18 (m, 2H), 7.21-7.27 (m, 4H), 7.35-7.43 (m, 5H), 7.55-7.59 (m, 3H), 8.02-8.05 (m, 2H), 8.61 (d, J = 6.4 Hz, 1H) ppm. ^{13}C NMR (101 MHz, $CDCl_3$): δ = 26.9, 109.3, 118.7, 119.6, 127.68, 127.69, 128.3, 129.4, 129.5, 129.57, 129.60, 132.4, 135.2, 135.7, 138.5, 140.5, 140.6, 152.3, 153.2, 161.7, 197.2 ppm. HRMS (ESI): calcd for $C_{27}H_{20}BF_2NNaO_2$ $[M+Na]^+$ 462.1453 found 462.1449.

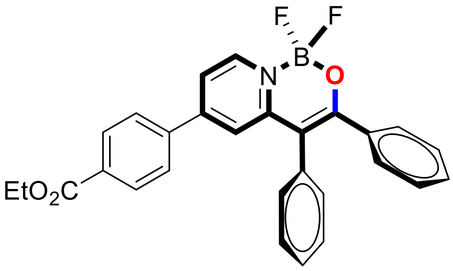

Ethyl 4-(1,1-difluoro-3,4-diphenyl-1*H*-1λ^4,9λ^4-pyrido[1,2-*c*][1,3,2]oxazaborinin-6-yl)benzoate (2af)

Following the general procedure. Ethyl (*E*)-4-(2-(1,2-diphenylvinyl)pyridin-4-yl)benzoate **1af** (40.5 mg, 0.1 mmol) was used. Purification via column chromatography on silica gel (pentane/EtOAc = 8/1, v/v) afforded **2af** as a yellow solid (45 mg, 96% yield). ^{1}H NMR (400 MHz, $CDCl_3$): δ = 1.40 (t, J = 7.2 Hz, 3H), 4.37-4.42 (m, 2H), 7.13-7.18 (m, 2H), 7.20-7.28 (m, 4H), 7.35-7.43 (m, 5H), 7.52-7.57 (m, 3H), 8.11-8.14 (m, 2H), 8.61 (d, J = 6.4 Hz, 1H) ppm. ^{13}C NMR (101 MHz, $CDCl_3$): δ = 14.4, 61.6, 109.4, 118.8, 119.6, 127.4, 127.6, 128.3, 129.5, 129.55, 129.59, 130.7, 132.38, 132.40, 135.1, 135.9, 140.4, 140.5, 152.5, 153.2, 161.5, 165.8 ppm. HRMS (ESI): calcd for $C_{28}H_{22}BF_2NNaO_3$ $[M+Na]^+$ 492.1559 found 492.1555.

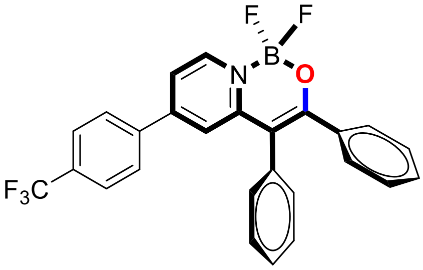

1,1-Difluoro-3,4-diphenyl-6-(4-(trifluoromethyl)phenyl)-1*H*-1l4,9l4-pyrido[1,2-*c*][1,3,2]oxazaborinine (2ag)

Following the general procedure. (*E*)-2-(1,2-Diphenylvinyl)-4-(4-(trifluoromethyl)phenyl)pyridine **1ag** (40.1 mg, 0.1 mmol) was used. Purification via column chromatography on silica gel (pentane/EtOAc = 8/1, v/v) afforded **2ag** as a yellow solid (42 mg, 90% yield). ^{1}H NMR (400 MHz, $CDCl_3$): δ = 7.12-7.16 (m, 2H), 7.19-7.26 (m, 4H), 7.33-7.43 (m,

5H), 7.53-7.49 (m, 3H), 7.71 (d, J = 8.0 Hz, 2H), 8.61 (d, J = 6.4 Hz, 1H) ppm. ^{13}C NMR (101 MHz, $CDCl_3$): δ = 109.3, 118.8, 119.7, 122.4, 125.1, 126.5 (q, J = 4.0 Hz), 127.7, 127.8, 128.3, 129.5 (q, J = 3.0 Hz), 132.3, 132.5 (q, J = 33.0 Hz), 135.1, 135.8, 139.9, 140.6 ppm. HRMS (ESI): calcd for $C_{26}H_{17}BF_5NNaO$ [M+Na]$^+$ 488.1221 found 488.1220.

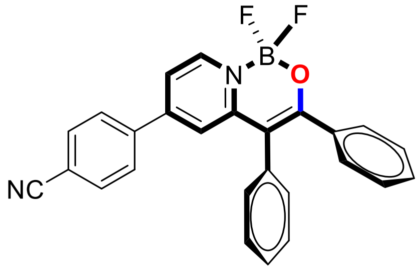

4-(1,1-Difluoro-3,4-diphenyl-1*H*-1λ^4,9λ^4-pyrido[1,2-*c*][1,3,2]oxazaborinin-6-yl)benzonitrile (2ah)

Following the general procedure. (*E*)-4-(2-(1,2-Diphenylvinyl)pyridin-4-yl)benzonitrile **1ah** (35.8 mg, 0.1 mmol) was used. Purification via column chromatography on silica gel (pentane/EtOAc = 8/1, v/v) afforded **2ah** as a yellow solid (28 mg, 66% yield). ^{1}H NMR (400 MHz, $CDCl_3$): δ = 7.14-7.18 (m, 2H), 7.21-7.26 (m, 4H), 7.34-7.37 (m, 2H), 7.38-7.43 (m, 3H), 7.52 (dd, J = 6.4 Hz, 2.0 Hz, 1H), 7.56-7.58 (m, 2H), 7.75-7.77 (m, 2H), 8.62 (d, J = 6.0 Hz, 1H) ppm. ^{13}C NMR (101 MHz, $CDCl_3$): δ = 109.3, 114.4, 118.0, 118.6, 119.7, 127.7, 128.1, 128.4, 129.59, 129.64, 132.3, 133.3, 135.0, 135.8, 140.7, 140.8, 151.5, 153.4, 161.7, 167.7 ppm. HRMS (ESI): calcd for $C_{26}H_{17}BF_2N_2NaO$ [M+Na]$^+$ 445.1300 found 445.1298.

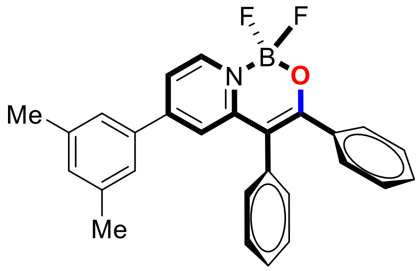

6-(3,5-Dimethylphenyl)-1,1-difluoro-3,4-diphenyl-1*H*-1λ^4,9λ^4-pyrido[1,2-*c*][1,3,2] oxazaborinine (2ai)

Following the general procedure. (*E*)-4-(3,5-Dimethylphenyl)-2-(1,2-diphenylvinyl)pyridine **1ai** (36.1 mg, 0.2 mmol) was used. Purification via column chromatography on silica gel (pentane/EtOAc = 8/1, v/v) afforded **2ai** as a yellow solid (39 mg, 93% yield). ^{1}H NMR (400 MHz, $CDCl_3$): δ = 2.33 (s, 6H), 7.06 (s, 2H), 7.10-7.16 (m, 3H), 7.18-7.27 (m, 4H), 7.33-7.41 (m, 5H), 7.50-7.53 (m, 1H), 8.54 (d, *J* = 6.4 Hz, 1H) ppm. ^{13}C NMR (101 MHz, $CDCl_3$): δ = 21.4, 109.4, 119.0, 119.2, 125.2, 127.6, 128.1, 129.3, 129.4, 129.6, 132.37, 132.43, 135.3, 136.1, 136.4, 139.3, 140.1, 152.8, 154.1, 160.9 ppm. HRMS (ESI): calcd for $C_{27}H_{22}BF_2NNaO$ $[M+Na]^+$ 448.1660 found 448.1659.

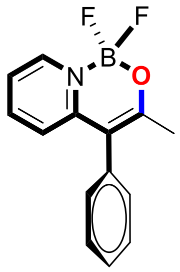

1,1-Difluoro-3-methyl-4-phenyl-1*H*-1λ^4,9λ^4-pyrido[1,2-*c*][1,3,2]oxazaborinine (2aj)

Following the general procedure. (*E*)-2-(1-Phenylprop-1-en-1-yl)pyridine **1aj** (19.5 mg, 0.1 mmol) was used. Purification via column chromatography on silica gel (pentane/EtOAc = 8/1, v/v) afforded **2aj** as a yellow solid (21 mg, 83% yield). ^{1}H NMR (400 MHz, $CDCl_3$): δ = 1.97 (s, 3H), 6.82 (d, *J* = 8.4 Hz, 1H), 7.21-7.29 (m, 3H), 7.39-7.49 (m, 3H), 7.39-7.78 (m, 1H), 8.44 (d, *J* = 6.4 Hz, 1H) ppm. ^{13}C NMR (101 MHz, $CDCl_3$): δ = 21.3, 108.6, 119.9, 121.2, 128.2, 129.5, 131.6, 135.4, 139.8, 141.3, 152.3, 164.1 ppm. HRMS (ESI): calcd for $C_{14}H_{12}BF_2NNaO$ $[M+Na]^+$ 282.0878 found 282.0874.

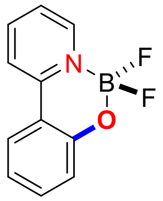

6,6-Difluoro-6*H*-6λ^4,7λ^4-benzo[*e*]pyrido[1,2-*c*][1,3,2]oxazaborinine (3a)

Following the general procedure. 2-Phenylpyridine **1ak** (15.5 mg, 0.1 mmol) was used. Purification via column chromatography on silica gel (pentane/EtOAc = 3/1, v/v)

afforded **3a** as a white solid (16 mg, 75% yield). ^{1}H NMR (400 MHz, $CDCl_3$): δ = 7.03-7.07 (m, 1H), 7.19 (dd, J = 8.4 Hz, 1.2 Hz, 1H), 7.48-7.52 (m, 1H), 7.57-7.60 (m, 1H), 7.83 (dd, J = 8.0 Hz, 1.6 Hz, 1H), 8.11-8.13 (m, 1H), 8.17-8.21 (m, 1H), 8.70 (d, J = 5.6 Hz, 1H) ppm. ^{13}C NMR (101 MHz, $CDCl_3$): δ = 120.6, 120.8, 120.9, 121.0, 123.0, 125.4, 134.9, 141.3, 142.4, 150.5, 156.0 ppm. HRMS (ESI): calcd for $C_{11}H_8BF_2NNaO$ $[M+Na]^+$ 242.0565 found 242.0560.

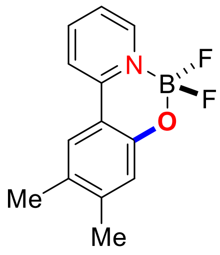

6,6-Difluoro-2,3-dimethyl-6*H*-6λ^4,7λ^4-benzo[*e*]pyrido[1,2-*c*][1,3,2]oxazaborinine (3b)

Following the general procedure. 2-(3,4-Dimethylphenyl)pyridine **1al** (18.3 mg, 0.1 mmol) was used. Purification via column chromatography on silica gel (pentane/EtOAc = 3/1, v/v) afforded **3b** as a yellow solid (19 mg, 78% yield). ^{1}H NMR (400 MHz, DMSO-d_6): δ = 2.25 (s, 3H), 2.26 (s, 3H), 6.89 (s, 1H), 7.76-7.79 (m, 1H), 7.95 (s, 1H), 8.40-8.44 (m, 1H), 8.50-8.52 (m, 1H), 8.69 (d, J = 5.6 Hz, 1H) ppm. ^{13}C NMR (101 MHz, DMSO-d_6): δ = 18.6, 19.7, 113.4, 120.1, 120.9, 123.8, 126.4, 128.8, 140.8, 143.6, 144.2, 149.0, 153.0 ppm. HRMS (ESI): calcd for $C_{13}H_{12}BF_2NNaO$ $[M+Na]^+$ 270.0878 found 270.0875.

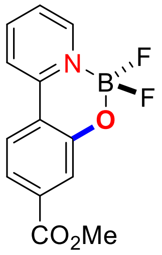

Methyl 6,6-difluoro-6*H*-6λ^4,7λ^4-benzo[*e*]pyrido[1,2-*c*][1,3,2]oxazaborinine-3-carboxylate (3c)

Following the general procedure. Methyl 4-(pyridin-2-yl)benzoate **1am** (21.3 mg, 0.1 mmol) was used. Purification via column chromatography on silica gel (pentane/EtOAc = 3/1, v/v) afforded **3c** as a yellow solid (19 mg, 69% yield). ^{1}H NMR (400 MHz, DMSO-d_6): δ = 3.89 (s, 3H), 7.56 (s, 1H), 7.63 (d, J = 6.4 Hz, 1H), 7.95 (t, J = 5.2 Hz, 1H), 8.36 (d, J = 6.8 Hz, 1H), 8.54-8.57 (m, 1H), 8.67-8.69 (m, 1H), 8.83 (d, J = 4.8 Hz, 1H) ppm. ^{13}C NMR (101 MHz, DMSO-d_6): δ = 52.5, 119.9, 120.0, 120.7, 122.4, 125.7, 127.1, 134.4, 141.5, 144.4, 147.4, 154.6, 165.3 ppm. HRMS (ESI): calcd for $C_{13}H_{10}BF_2NNaO_3$ [M+Na]$^+$ 300.0619 found 300.0620.

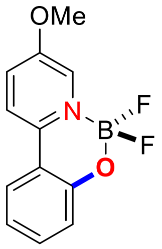

6,6-Difluoro-9-methoxy-6*H*-6λ^4,7λ^4-benzo[*e*]pyrido[1,2-*c*][1,3,2]oxazaborinine (3d)

Following the general procedure. 5-Methoxy-2-phenylpyridine **1an** (18.5 mg, 0.1 mmol) was used. Purification via column chromatography on silica gel (pentane/EtOAc = 3/1, v/v) afforded **3d** as a yellow solid (14 mg, 69% yield). ^{1}H NMR (400 MHz, DMSO-d_6): δ = 2.59 (s, 3H), 7.05-7.12 (m, 2H), 7.50-7.54 (m, 1H), 7.66-7.68 (m, 1H), 8.18-8.20 (m, 1H), 8.46 (s, 1H), 8.59 (d, J = 6.4 Hz, 1H) ppm. ^{13}C NMR (101 MHz, DMSO-d_6): δ = 56.4, 115.9, 118.9, 120.2, 122.4, 125.4, 125.8, 130.3, 132.8, 141.7, 153.5, 155.6 ppm. HRMS (ESI): calcd for $C_{12}H_{10}BF_2NNaO_2$ [M+Na]$^+$ 272.0670 found 272.0668.

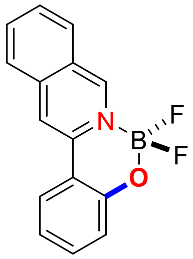

6,6-Difluoro-6*H*-6λ^4,7λ^4-benzo[5,6][1,3,2]oxazaborinino[3,4-*b*]isoquinoline (3e)

Following the general procedure. 5-Methoxy-2-phenylpyridine **1ao** (20.5 mg, 0.1 mmol) was used. Purification via column chromatography on silica gel (pentane/EtOAc = 3/1, v/v) afforded **3e** as a yellow solid (20 mg, 73% yield). ^{1}H NMR (400 MHz, DMSO-d_6): δ = 7.08-7.16 (m, 2H), 7.46-7.50 (m, 1H), 7.87-7.91 (m, 1H), 8.09-8.13 (m, 1H), 8.22-8.26 (m, 2H), 8.55-8.58 (m, 1H), 9.06 (s, 1H), 9.84 (s, 1H) ppm. ^{13}C NMR (101 MHz, DMSO-d_6): δ = 117.2, 118.4, 119.7, 120.8, 125.7, 126.3, 127.1, 129.5, 130.1, 132.9, 135.5, 138.1, 140.7, 146.4, 154.1 ppm. HRMS (ESI): calcd for $C_{15}H_{10}BF_2NNaO$ [M+Na]$^+$ 292.0721 found 292.0720.

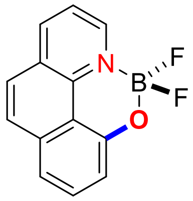

4,4-Difluoro-4*H*-5-oxa-3aλ^4-aza-4λ^4-borapyrene (3f)

Following the general procedure. Benzo[*h*]quinoline **1ap** (17.9 mg, 0.1 mmol) was used. Purification via column chromatography on silica gel (pentane/EtOAc = 3/1, v/v) afforded **3f** as a yellow solid (10 mg, 42% yield). ^{1}H NMR (400 MHz, DMSO-d_6): δ = 7.32-7.35 (m, 1H), 7.69-7.72 (m, 1H), 7.88 (t, J = 8.0 Hz, 1H), 8.06-8.09 (m, 1H), 8.16-8.20 (m, 2H), 9.07-9.09 (m, 1H), 9.16 (dd, J = 6.0 Hz, 0.8 Hz, 1H) ppm. ^{13}C NMR (101 MHz, DMSO-d_6): δ = 112.2, 115.3, 119.1, 123.2, 124.7, 126.4, 130.0, 132.8, 134.3, 137.8, 141.6, 142.8, 153.2 ppm. HRMS (ESI): calcd for $C_{13}H_8BF_2NNaO$ [M+Na]$^+$ 266.0565 found 266.0566.

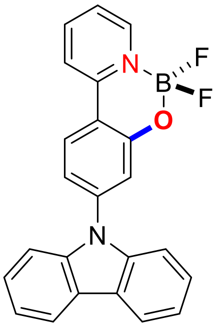

3-(9*H*-Carbazol-9-yl)-6,6-difluoro-6*H*-6λ^4,7λ^4-benzo[*e*]pyrido[1,2-*c*][1,3,2]oxazab orinine (3g)

Following the general procedure. 9-(4-(Pyridin-2-yl)phenyl)-9*H*-carbazole **1aq** (32.0 mg, 0.1 mmol) was used. Purification via column chromatography on silica gel

(pentane/EtOAc = 3/1, v/v) afforded **3g** as a yellow solid (31 mg, 82% yield). ^{1}H NMR (400 MHz, DMSO-d_6): δ = 7.31-7.35 (m, 2H), 7.38-7.43 (m, 2H), 7.46-7.50 (m, 2H), 7.54-7.57 (m, 2H), 7.89-7.93 (m, 1H), 8.25-8.28 (m, 2H), 8.50-8.58 (m, 2H), 8.69-8.71 (m, 1H), 8.82 (dd, J = 6.0 Hz, 1.2 Hz, 1H) ppm. ^{13}C NMR (101 MHz, DMSO-d_6): δ = 110.0, 115.0, 116.6, 118.5, 120.6, 121.7, 123.2, 124.8, 126.4, 126.5, 128.4, 139.5, 141.3, 142.2, 144.2, 148.1, 156.2 ppm. HRMS (ESI): calcd for $C_{23}H_{15}BF_2N_2NaO$ [M+Na]$^+$ 407.1143 found 407.1145.

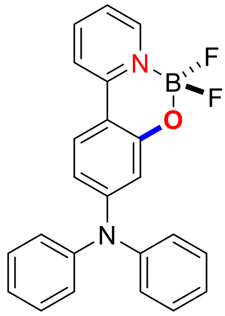

6,6-Difluoro-*N*,*N*-diphenyl-6*H*-6λ^4,7λ^4-benzo[*e*]pyrido[1,2-*c*][1,3,2]oxazaborinin-3-amine (3h)

Following the general procedure. *N*,*N*-Diphenyl-4-(pyridin-2-yl)aniline **1ar** (32.2 mg, 0.1 mmol) was used. Purification via column chromatography on silica gel (pentane/EtOAc = 3/1, v/v) afforded **3h** as a yellow solid (28 mg, 72% yield). ^{1}H NMR (400 MHz, $CDCl_3$): δ = 6.61-6.66 (m, 2H), 7.13-7.20 (m, 6H), 7.30-7.40 (m, 5H), 7.58 (d, J = 8.8 Hz, 1H), 7.90 (d, J = 8.4 Hz, 1H), 8.01-8.05 (m, 1H), 8.54 (dd, J = 6.0 Hz, 1.6 Hz, 1H) ppm. ^{13}C NMR (101 MHz, $CDCl_3$): δ = 108.6, 109.6, 113.1, 119.5, 121.0, 125.2, 126.2, 126.6, 129.8, 140.8, 141.7, 146.2, 150.4, 154.0, 157.3 ppm. HRMS (ESI): calcd for $C_{23}H_{17}BF_2N_2NaO$ [M+Na]$^+$ 409.1300 found 409.1308.

VIII. Photophysical properties of selected products

Complete description of the equipment: Steady-state excitation and emission spectra were recorded on a FluoTime300 spectrometer from PicoQuant equipped with a 300 W ozone-free Xe lamp (250-900 nm), a 10 W Xe flash-lamp (250-900 nm, pulse width < 10 µs) with repetition rates of 0.1 – 300 Hz, two excitation monochromators (Czerny-Turner 2.7 nm/mm dispersion, 1200 grooves/mm, blazed at 350 nm and 600 g/mm, blazed at 1250 nm), diode lasers (pulse width < 80 ps) operated by a computer-controlled laser driver PDL-820 (repetition rate up to 80 MHz, burst mode for slow and weak decays), two emission monochromators (Czerny-Turner, selectable gratings blazed at 500 nm with 2.7 nm/mm dispersion and 1200 grooves/mm, or blazed at 1250 nm with 5.4 nm/mm dispersion and 600 grooves/mm) with adjustable slit width between 0 mm and 10 mm, Glan-Thompson polarizers for excitation (Xe-lamps) and emission, a Peltier-thermostatized sample holder (-40 ℃ – 105 ℃), and two detectors, namely a PMA Hybrid 40 (transit time spread FWHM < 120 ps, 200 – 900 nm) and a R5509-42 NIR-photomultiplier tube (transit time spread FWHM 1.5 ns, 300-1400 nm) from Hamamatsu. Signal-to-noise ratio (optical noise) typically better than 29000:1, as measured with double monochromators in the excitation and emission light path. Steady-state and fluorescence lifetimes were recorded in TCSPC mode by a PicoHarp 300 (minimum base resolution 4 ps) or MSC mode by a Timeharp 300, where up to several ms can be detected. Emission and excitation spectra were corrected for source intensity (lamp and grating) by standard correction curves. For samples with lifetime in ns order instrument response function calibration (IRF) was performed using a diluted Ludox® solution. Lifetime analysis was performed using the commercial FluoFit software. The quality of the fit was assessed by minimizing the reduced chi squared function ($\chi 2$) and visual inspection of the weighted residuals and their autocorrelation.

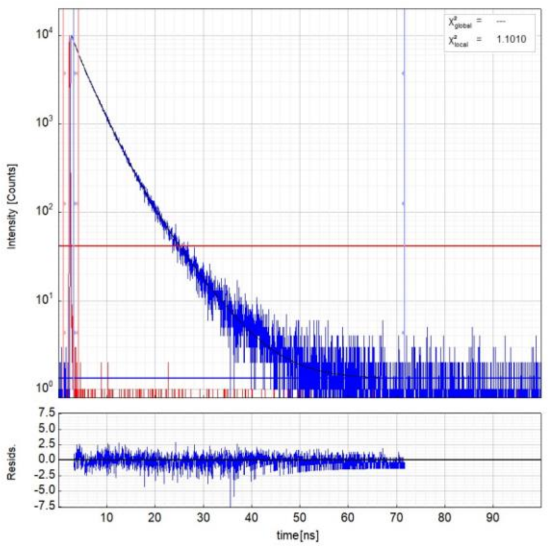

Parameter	Value	Error
A_1[kCnts/Chnl]	1.56	± 0.46
τ_1[ns]	6.13	± 0.46
I_1[kCnts]	300	± 70
$A_{Rel\,1}$[%]	13.1	± 3.8
$I_{Rel\,1}$[%]	22.7	± 5.2
A_2[kCnts/Chnl]	10.40	± 0.61
τ_2[ns]	3.13	± 0.12
I_2[kCnts]	1019	± 90
$A_{Rel\,2}$[%]	86.9	± 3.8
$I_{Rel\,2}$[%]	77.3	± 5.2
$Bkgr_{Dec}$[kCnts]	0.0013	± 0.0002
$Bkgr_{IRF}$[Cnts/Chnl]	41.5	± 9.7
$Shift_{IRF}$[ps]	-244	± 112
$\tau_{Av\,Int}$[ns]	3.814	± 0.028
$\tau_{Av\,Amp}$[ns]	3.525	± 0.041

Figure S3. Left: Time-resolved photoluminescence decay of **2a** (blue) at 298K in the solid state, including the residuals and the Instrument Response Function (red) (λ_{ex} = 376.7 nm, λ_{em} = 482 nm). Right: Fitting parameters including pre-exponential factors and confidence limits.

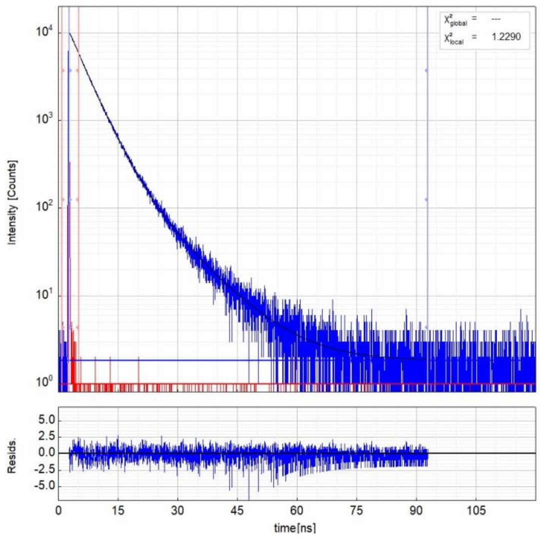

A_1[kCnts/Chnl]	0.728	± 0.086
τ_1[ns]	9.53	± 0.35
I_1[kCnts]	217	± 19
$A_{Rel\,1}$[%]	6.79	± 0.80
$I_{Rel\,1}$[%]	15.0	± 1.4
A_2[kCnts/Chnl]	10.00	± 0.16
τ_2[ns]	3.948	± 0.047
I_2[kCnts]	1233	± 33
$A_{Rel\,2}$[%]	93.21	± 0.80
$I_{Rel\,2}$[%]	85.0	± 1.4
$Bkgr_{Dec}$[kCnts]	0.0018	± 0.0002
$Bkgr_{IRF}$[Cnts/Chnl]	1.0	± 4.4
$Shift_{IRF}$[ps]	166	± 48
$\tau_{Av\,Int}$[ns]	4.783	± 0.020
$\tau_{Av\,Amp}$[ns]	4.327	± 0.025

Figure S4. Left: Time-resolved photoluminescence decay of **2a** (blue) in a 2-Me-THF glassy matrix at 77 K, including the residuals and the Instrument Response Function (red) (λ_{ex} = 376.7 nm, λ_{em} = 470 nm). Right: Fitting parameters including pre-exponential factors and confidence limits.

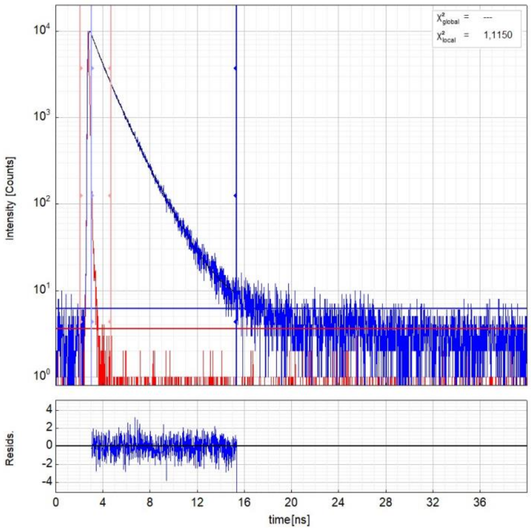

A_1[kCnts/Chnl]	4,87	± 0,62
τ_1[ns]	1,711	± 0,082
I_1[kCnts]	520	± 38
$A_{Rel\,1}$[%]	43,2	± 7,7
$I_{Rel\,1}$[%]	57,4	± 9,1
A_2[kCnts/Chnl]	6,4	± 1,2
τ_2[ns]	0,97	± 0,13
I_2[kCnts]	386	± 108
$A_{Rel\,2}$[%]	56,8	± 7,7
$I_{Rel\,2}$[%]	42,6	± 9,1
$Bkgr_{Dec}$[kCnts]	0,0061	± 0,0013
$Bkgr_{IRF}$[Cnts/Chnl]	4	± 13
$Shift_{IRF}$[ps]	-65	± 38
A_{Scatt}[kCnts]	120	± 116
$Period_{Rep}$[ns]	6	± 223
$\tau_{Av\,Int}$[ns]	1,394	± 0,031
$\tau_{Av\,Amp}$[ns]	1,289	± 0,052

Figure S5. Left: Time-resolved photoluminescence decay of **2k** (blue) in the solid state at 298 K, including the residuals and the Instrument Response Function (red) (λ_{ex} = 376.7 nm, λ_{em} = 470 nm). Right: Fitting parameters including pre-exponential factors and confidence limits.

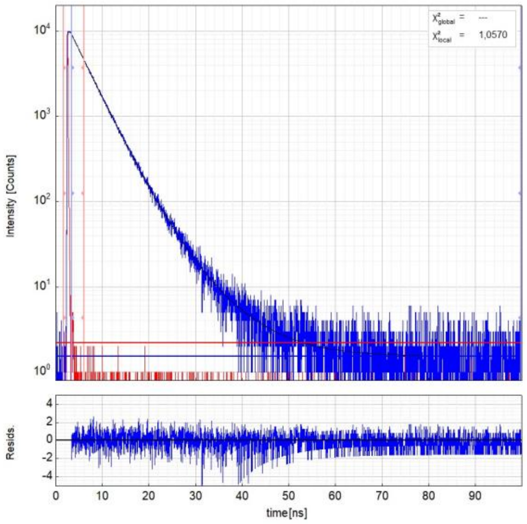

Parameter	Value	Error
A_1[kCnts/Chnl]	0,30	± 0,31
τ_1[ns]	8,3	± 1,4
I_1[kCnts]	78	± 57
$A_{Rel\,1}$[%]	2,6	± 2,7
$I_{Rel\,1}$[%]	5,6	± 4,1
A_2[kCnts/Chnl]	11,2	± 1,4
τ_2[ns]	3,798	± 0,092
I_2[kCnts]	1334	± 180
$A_{Rel\,2}$[%]	97,4	± 2,7
$I_{Rel\,2}$[%]	94,4	± 4,1
$Bkgr_{Dec}$[kCnts]	0,0015	± 0,0002
$Bkgr_{IRF}$[Cnts/Chnl]	2,2	± 4,6
$Shift_{IRF}$[ps]	60	± 475
$\tau_{Av\,Int}$[ns]	4,046	± 0,033
$\tau_{Av\,Amp}$[ns]	3,915	± 0,041

Figure S6. Left: Time-resolved photoluminescence decay of **2k** (blue) in a 2-Me-THF glassy matrix at 77 K, including the residuals and the Instrument Response Function (red) (λ_{ex} = 376.7 nm, λ_{em} = 440 nm). Right: Fitting parameters including pre-exponential factors and confidence limits.

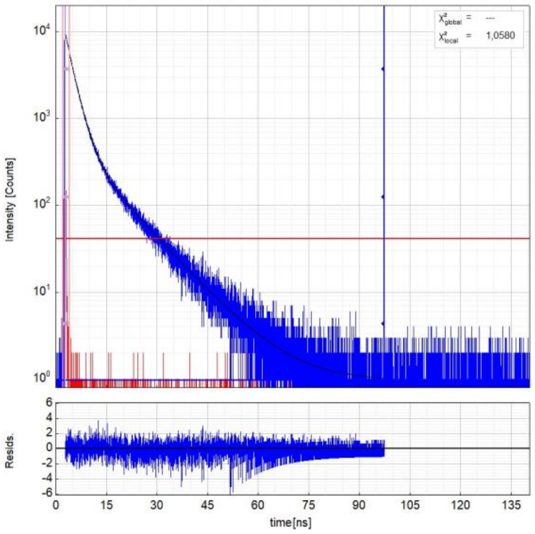

Parameter	Value	Error
A_1[kCnts/Chnl]	0,645	± 0,017
τ_1[ns]	10,32	± 0,12
I_1[kCnts]	415,9	± 7,4
$A_{Rel\,1}$[%]	6,07	± 0,20
$I_{Rel\,1}$[%]	22,41	± 0,53
A_2[kCnts/Chnl]	9,98	± 0,39
τ_2[ns]	2,309	± 0,019
I_2[kCnts]	1440	± 57
$A_{Rel\,2}$[%]	93,93	± 0,20
$I_{Rel\,2}$[%]	77,59	± 0,53
$Bkgr_{Dec}$[kCnts]	0,0010	± 0,0001
$Bkgr_{IRF}$[Cnts/Chnl]	41,2	± 8,2
$Shift_{IRF}$[ps]	181	± 84
$\tau_{Av\,Int}$[ns]	4,103	± 0,043
$\tau_{Av\,Amp}$[ns]	2,796	± 0,022

Figure S7. Left: Time-resolved photoluminescence decay of **2l** in the solid state at 298 K (blue), including the residuals and the Instrument Response Function (red) (λ_{ex} = 376.7 nm, λ_{em} = 490 nm). Right: Fitting parameters including pre-exponential factors and confidence limits.

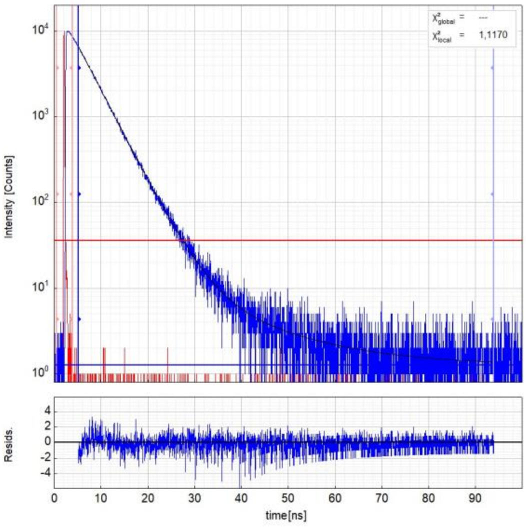

A_1[kCnts/Chnl]	0,037	± 0,011
τ_1[ns]	16,0	± 2,3
I_1[kCnts]	18,7	± 3,0
$A_{Rel\,1}$[%]	0,239	± 0,065
$I_{Rel\,1}$[%]	0,93	± 0,15
A_2[kCnts/Chnl]	15,56	± 0,16
τ_2[ns]	4,084	± 0,016
I_2[kCnts]	1985	± 21
$A_{Rel\,2}$[%]	99,761	± 0,065
$I_{Rel\,2}$[%]	99,07	± 0,15
$Bkgr_{Dec}$[kCnts]	0,0013	± 0,0002
$Bkgr_{IRF}$[Cnts/Chnl]	36,0	± 5,4
$Shift_{IRF}$[ps]	-351	± 57
$\tau_{Av\,Int}$[ns]	4,195	± 0,018
$\tau_{Av\,Amp}$[ns]	4,112	± 0,014

Figure S8. Left: Time-resolved photoluminescence decay of **2l** in a 2-Me-THF glassy matrix at 77 K (blue), including the residuals and the Instrument Response Function (red) (λ_{ex} = 376.7 nm, λ_{em} = 475 nm). Right: Fitting parameters including pre-exponential factors and confidence limits.

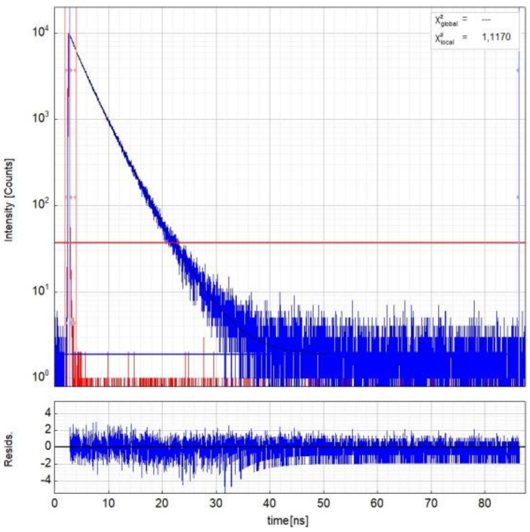

A_1[kCnts/Chnl]	7,4	± 1,4
τ_1[ns]	2,64	± 0,25
I_1[kCnts]	1228	± 322
$A_{Rel\,1}$[%]	70	± 14
$I_{Rel\,1}$[%]	59	± 16
A_2[kCnts/Chnl]	3,2	± 1,5
τ_2[ns]	4,31	± 0,30
I_2[kCnts]	854	± 311
$A_{Rel\,2}$[%]	30	± 14
$I_{Rel\,2}$[%]	41	± 16
$Bkgr_{Dec}$[kCnts]	0,0019	± 0,0001
$Bkgr_{IRF}$[Cnts/Chnl]	37	± 15
$Shift_{IRF}$[ps]	47	± 162
A_{Scatt}[kCnts]	-59	± 298
$\tau_{Av\,Int}$[ns]	3,328	± 0,022
$\tau_{Av\,Amp}$[ns]	3,142	± 0,043

Figure S9. Left: Time-resolved photoluminescence decay of **2s** in the solid state at 298 K (blue), including the residuals and the Instrument Response Function (red) (λ_{ex} = 376.7 nm, λ_{em} = 480 nm). Right: Fitting parameters including pre-exponential factors and confidence limits.

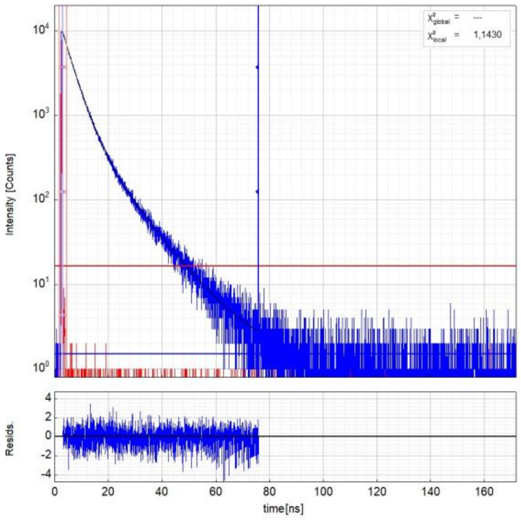

A_1[kCnts/Chnl]	1,061	± 0,059
τ_1[ns]	11,02	± 0,24
I_1[kCnts]	365	± 14
$A_{Rel\,1}$[%]	9,35	± 0,56
$I_{Rel\,1}$[%]	22,85	± 0,97
A_2[kCnts/Chnl]	10,29	± 0,51
τ_2[ns]	3,837	± 0,039
I_2[kCnts]	1234	± 61
$A_{Rel\,2}$[%]	90,65	± 0,56
$I_{Rel\,2}$[%]	77,15	± 0,97
$Bkgr_{Dec}$[kCnts]	0,0015	± 0,0003
$Bkgr_{IRF}$[Cnts/Chnl]	16,5	± 5,3
$Shift_{IRF}$[ps]	150	± 175
$\tau_{Av\,Int}$[ns]	5,477	± 0,048
$\tau_{Av\,Amp}$[ns]	4,508	± 0,031

Figure S10. Left: Time-resolved photoluminescence decay of **2s** in a 2-Me-THF glassy matrix at 77 K (blue), including the residuals and the Instrument Response Function (red) (λ_{ex} = 376.7 nm, λ_{em} = 465 nm). Right: Fitting parameters including pre-exponential factors and confidence limits.

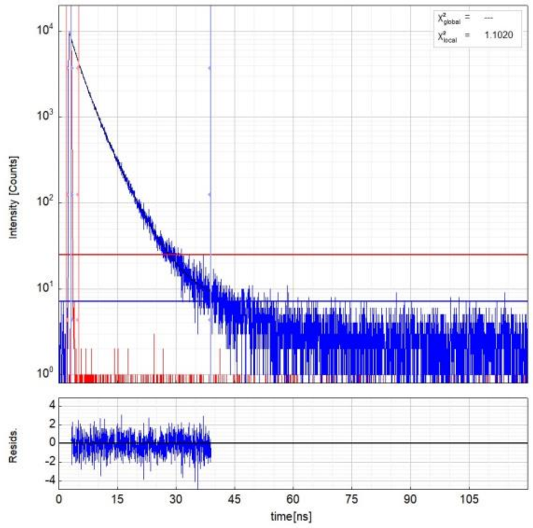

A_1[kCnts/Chnl]	2.48	± 0.75
τ_1[ns]	5.13	± 0.40
I_1[kCnts]	397	± 90
$A_{Rel\,1}$[%]	30.4	± 8.6
$I_{Rel\,1}$[%]	46	± 11
A_2[kCnts/Chnl]	5.67	± 0.77
τ_2[ns]	2.68	± 0.23
I_2[kCnts]	474	± 102
$A_{Rel\,2}$[%]	69.6	± 8.6
$I_{Rel\,2}$[%]	54	± 11
$Bkgr_{Dec}$[kCnts]	0.0072	± 0.0012
$Bkgr_{IRF}$[Cnts/Chnl]	25	± 13
$Shift_{IRF}$[ps]	669	± 126
A_{Scatt}[kCnts]	12.9	± 6.4
$\tau_{Av\,Int}$[ns]	3.794	± 0.046
$\tau_{Av\,Amp}$[ns]	3.422	± 0.064

Figure S11. Left: Time-resolved photoluminescence decay of **2y** in the solid state at 298 K (blue), including the residuals and the Instrument Response Function (red) (λ_{ex} = 376.7 nm, λ_{em} = 493 nm). Right: Fitting parameters including pre-exponential factors and confidence limits.

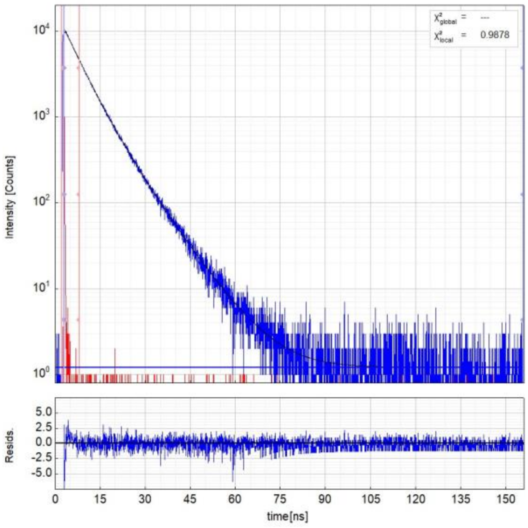

A_1[kCnts/Chnl]	2.97	± 0.52
τ_1[ns]	9.04	± 0.33
I_1[kCnts]	420	± 58
$A_{Rel\,1}$[%]	27.2	± 4.8
$I_{Rel\,1}$[%]	40.3	± 6.0
A_2[kCnts/Chnl]	7.94	± 0.54
τ_2[ns]	5.02	± 0.23
I_2[kCnts]	623	± 68
$A_{Rel\,2}$[%]	72.8	± 4.8
$I_{Rel\,2}$[%]	59.7	± 6.0
$Bkgr_{Dec}$[kCnts]	0.0012	± 0.0001
$Bkgr_{IRF}$[Cnts/Chnl]	-4.6	± 4.1
$Shift_{IRF}$[ps]	526	± 110
$\tau_{Av\,Int}$[ns]	6.638	± 0.034
$\tau_{Av\,Amp}$[ns]	6.116	± 0.068

Figure S12. Left: Time-resolved photoluminescence decay of **2y** in a 2-Me-THF glassy matrix at 77 K (blue), including the residuals and the Instrument Response Function (red) (λ_{ex} = 376.7 nm, λ_{em} = 480 nm). Right: Fitting parameters including pre-exponential factors and confidence limits.

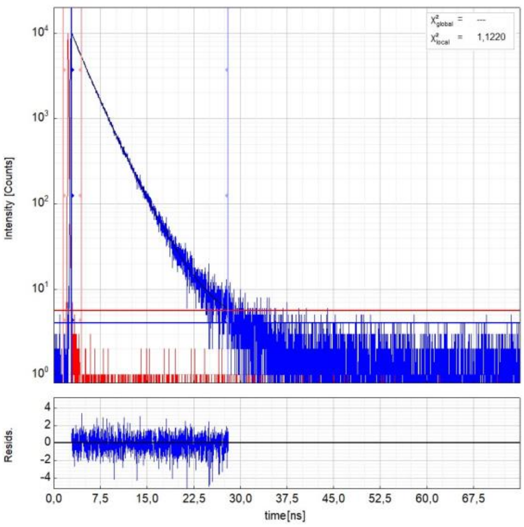

A_1[kCnts/Chnl]	4,2	± 3,0
τ_1[ns]	3,35	± 0,34
I_1[kCnts]	879	± 471
$A_{Rel\,1}$[%]	33	± 23
$I_{Rel\,1}$[%]	43	± 25
A_2[kCnts/Chnl]	8,6	± 3,0
τ_2[ns]	2,14	± 0,34
I_2[kCnts]	1156	± 514
$A_{Rel\,2}$[%]	67	± 23
$I_{Rel\,2}$[%]	57	± 25
$Bkgr_{Dec}$[kCnts]	0,0040	± 0,0011
$Bkgr_{IRF}$[Cnts/Chnl]	6	± 13
$Shift_{IRF}$[ps]	-363	± 90
$\tau_{Av\,Int}$[ns]	2,664	± 0,031
$\tau_{Av\,Amp}$[ns]	2,538	± 0,063

Figure S13. Left: Time-resolved photoluminescence decay of **2aj** in the solid state at 298 K (blue), including the residuals and the Instrument Response Function (red) (λ_{ex} = 376.7 nm, λ_{em} = 430 nm). Right: Fitting parameters including pre-exponential factors and confidence limits.

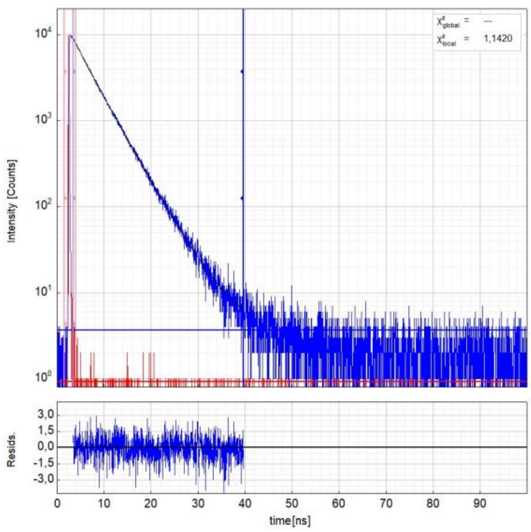

A_1[kCnts/Chnl]	9,0	± 4,7
τ_1[ns]	4,59	± 0,60
I_1[kCnts]	1289	± 580
$A_{Rel\,1}$[%]	77	± 39
$I_{Rel\,1}$[%]	86	± 39
A_2[kCnts/Chnl]	2,7	± 4,5
τ_2[ns]	2,6	± 1,1
I_2[kCnts]	219	± 583
$A_{Rel\,2}$[%]	23	± 39
$I_{Rel\,2}$[%]	14	± 39
$Bkgr_{Dec}$[kCnts]	0,0037	± 0,0015
$Bkgr_{IRF}$[Cnts/Chnl]	0,9	± 7,7
$Shift_{IRF}$[ps]	-54	± 45
A_{Scatt}[kCnts]	-2989	± 4658
$\tau_{Av\,Int}$[ns]	4,293	± 0,035
$\tau_{Av\,Amp}$[ns]	4,113	± 0,091

Figure S14. Left: Time-resolved photoluminescence decay of **2aj** in a 2-Me-THF glassy matrix at 77 K (blue), including the residuals and the Instrument Response Function (red) (λ_{ex} = 376.7 nm, λ_{em} = 418 nm). Right: Fitting parameters including pre-exponential factors and confidence limits.

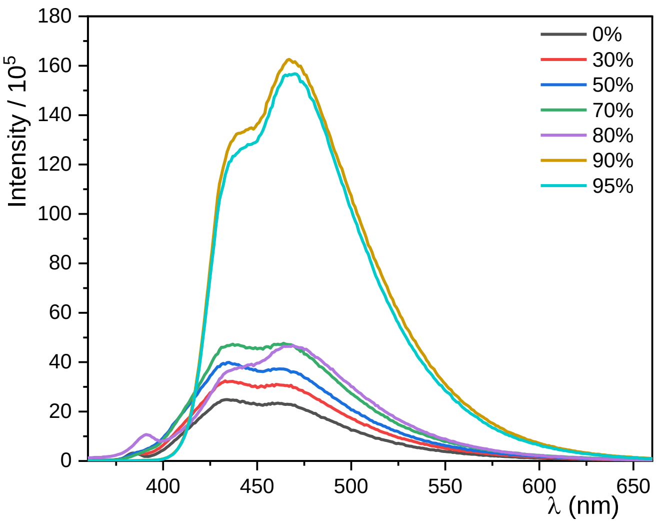

Figure S15. Photoluminescence spectra of **2a** in different THF/Water mixtures (λ_{ex} = 330 nm)

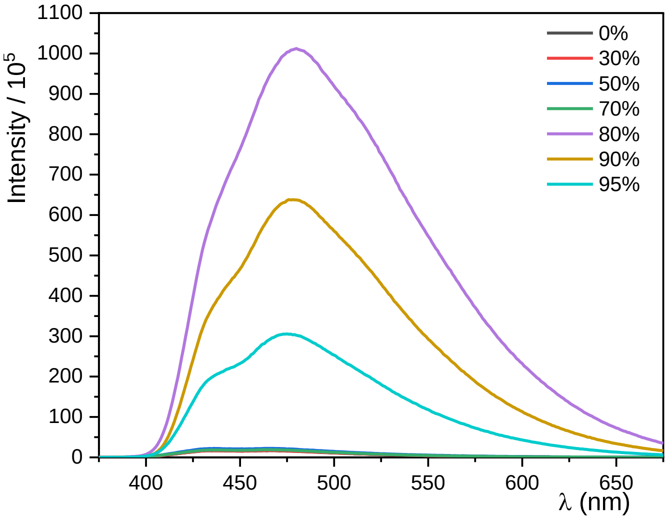

Figure S16. Photoluminescence spectra of **2k** in different THF/Water mixtures (λ_{ex} = 330 nm)

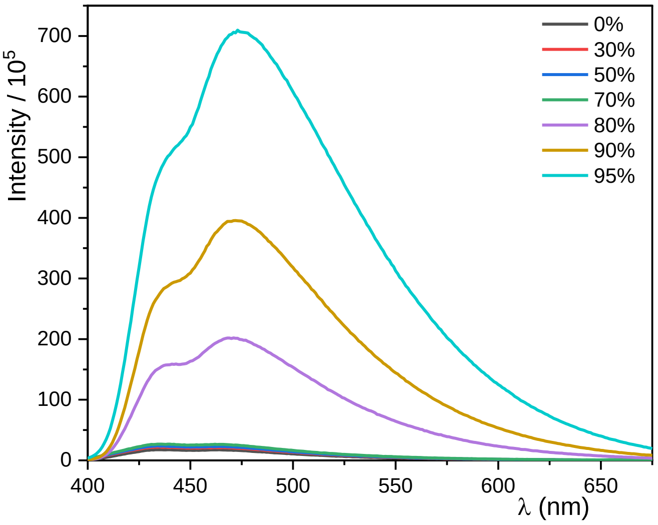

Figure S17. Photoluminescence spectra of **2l** in different THF/Water mixtures (λ_{ex} = 330 nm)

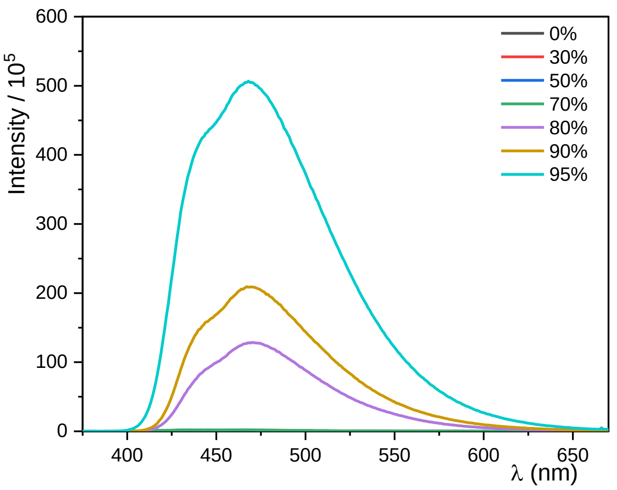

Figure S18. Photoluminescence spectra of **2s** in different THF/Water mixtures (λ_{ex} = 330 nm).

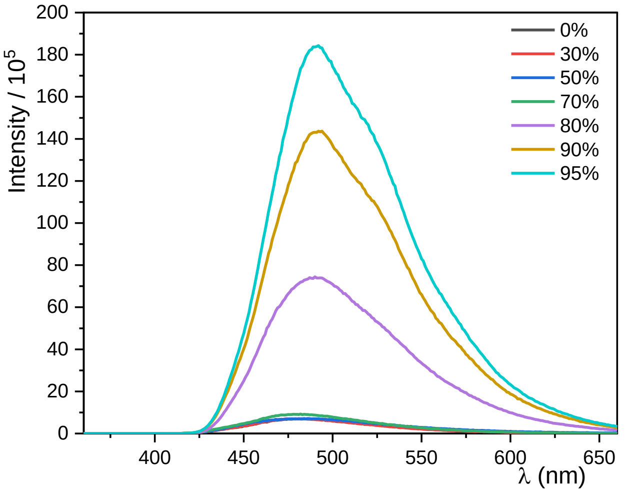

Figure S19. Photoluminescence spectra of **2y** in different THF/Water mixtures (λ_{ex} = 330 nm).

Table S3: Emission maxima, Φ_F and τ for the higher fractions of H_2O. τ_{av_amp}: average lifetimes (amplitude weighted). Less than 70% water always gave Φ_F < 2%.

H_2O content (%)	**2a**	**2k**	**2l**	**2s**	**2y**
	λ_{max} (nm)	λ_{max} (nm)	λ_{max} (nm)	λ_{max} (nm)	λ_{max} (nm)
	$\Phi_F \pm 2$ / %	$\Phi_F \pm 2$ / %	$\Phi_F \pm 2$ / %	$\Phi_F \pm 2$ / %	$\Phi_F \pm 2$ / %
	τ_{av_amp} (ns)	τ_{av_amp} (ns)	τ_{av_amp} (ns)	τ_{av_amp} (ns)	τ_{av_amp} (ns)
80	468	480	470	488	488
	< 2	41	3	50	50
	0.13 ±0.03	0.085 ±0.002	0.072 ±0.004	2.61 ±0.01	0.097 ±0.004
90	468	478	472	491	491
	46	34	8	57	57
	3.51 ±0.08	0.115 ±0.002	0.122 ±0.006	2.55 ±0.01	1.42 ±0.03

95	468	475	475	490	490
	58	22	14	60	61
	3.57 ±0.14	0.207 ±0.009	0.23 ±0.04	2.28 ±0.06	2.06 ±016

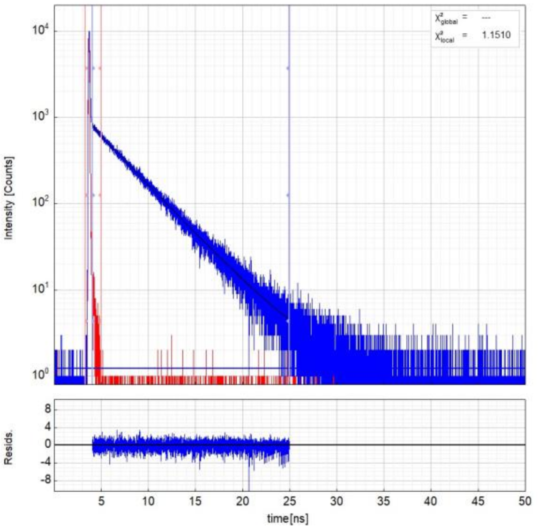

A_1[kCnts/Chnl]	0.8439	± 0.0081
τ_1[ns]	3.8322	± 0.0046
I_1[kCnts]	808.5	± 6.8
$A_{Rel\,1}$[%]	2.01	± 0.57
$I_{Rel\,1}$[%]	59.2	± 4.0
A_2[kCnts/Chnl]	41	± 13
τ_2[ns]	0.0543	± 0.0057
I_2[kCnts]	557	± 92
$A_{Rel\,2}$[%]	97.99	± 0.57
$I_{Rel\,2}$[%]	40.8	± 4.0
$Bkgr_{Dec}$[kCnts]	0.0012	± 0.0001
$Bkgr_{IRF}$[Cnts/Chnl]	-5.3	± 2.6
$Shift_{IRF}$[ps]	-19	± 26
$\tau_{Av\,Int}$[ns]	2.29	± 0.15
$\tau_{Av\,Amp}$[ns]	0.130	± 0.028

Figure S20. Left: Time-resolved photoluminescence decay of **2a** (blue) in a THF/water mixture (80/20) fraction at 298 K, including the residuals and the Instrument Response Function (red) (λ_{ex} = 376.7 nm, λ_{em} = 460 nm).

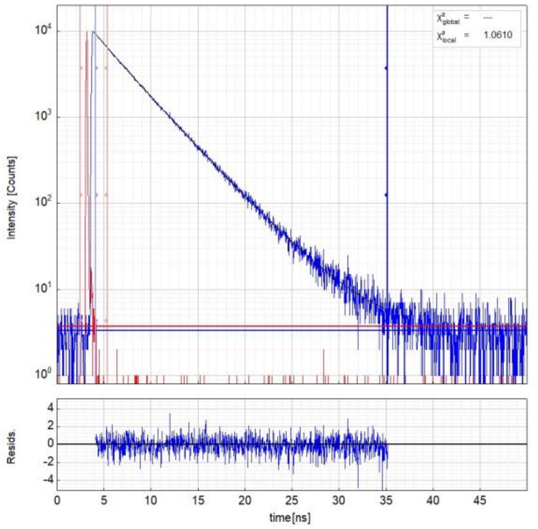

A_1[kCnts/Chnl]	6.9	± 2.8
τ_1[ns]	4.03	± 0.27
I_1[kCnts]	869	± 269
$A_{Rel\,1}$[%]	56	± 27
$I_{Rel\,1}$[%]	64	± 27
A_2[kCnts/Chnl]	5.5	± 3.4
τ_2[ns]	2.87	± 0.94
I_2[kCnts]	491	± 371
$A_{Rel\,2}$[%]	44	± 27
$I_{Rel\,2}$[%]	36	± 27
$Bkgr_{Dec}$[kCnts]	0.0033	± 0.0009
$Bkgr_{IRF}$[Cnts/Chnl]	4	± 13
$Shift_{IRF}$[ps]	-544	± 236
$\tau_{Av\,Int}$[ns]	3.609	± 0.027
$\tau_{Av\,Amp}$[ns]	3.515	± 0.076

Figure S21. Left: Time-resolved photoluminescence decay of **2a** (blue) in a THF/water mixture (90/10) fraction at 298 K, including the residuals and the Instrument Response Function (red) (λ_{ex} = 376.7 nm, λ_{em} = 460 nm).

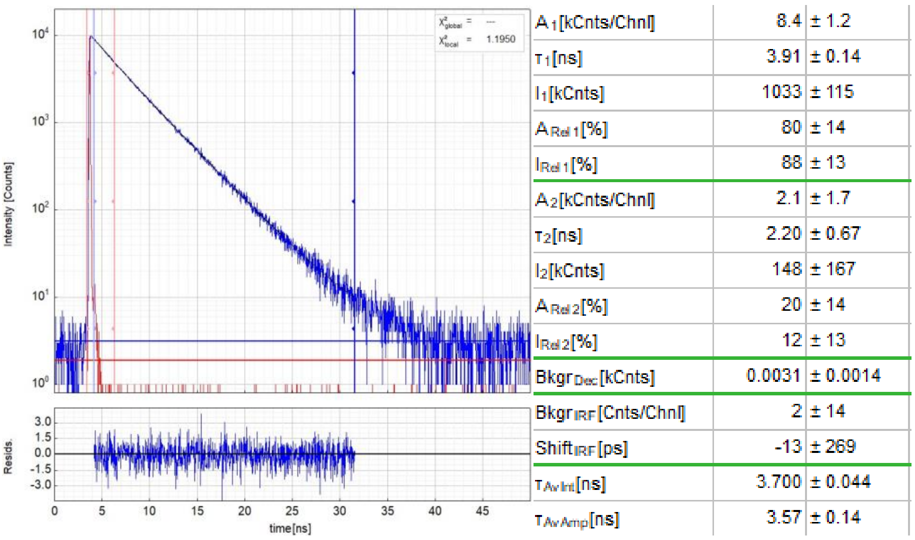

A_1[kCnts/Chnl]	8.4	± 1.2
τ_1[ns]	3.91	± 0.14
I_1[kCnts]	1033	± 115
$A_{Rel\,1}$[%]	80	± 14
$I_{Rel\,1}$[%]	88	± 13
A_2[kCnts/Chnl]	2.1	± 1.7
τ_2[ns]	2.20	± 0.67
I_2[kCnts]	148	± 167
$A_{Rel\,2}$[%]	20	± 14
$I_{Rel\,2}$[%]	12	± 13
$Bkgr_{Dec}$[kCnts]	0.0031	± 0.0014
$Bkgr_{IRF}$[Cnts/Chnl]	2	± 14
$Shift_{IRF}$[ps]	-13	± 269
$\tau_{Av\,Int}$[ns]	3.700	± 0.044
$\tau_{Av\,Amp}$[ns]	3.57	± 0.14

Figure S22. Left: Time-resolved photoluminescence decay of **2a** (blue) in a THF/water mixture (95/5) fraction at 298 K, including the residuals and the Instrument Response Function (red) (λ_{ex} = 376.7 nm, λ_{em} = 460 nm).

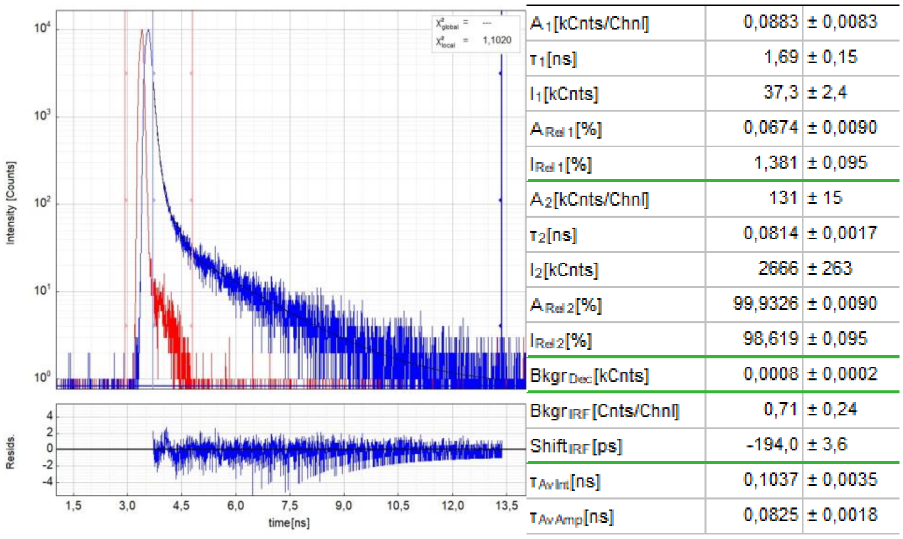

A_1[kCnts/Chnl]	0,0883	± 0,0083
τ_1[ns]	1,69	± 0,15
I_1[kCnts]	37,3	± 2,4
$A_{Rel\,1}$[%]	0,0674	± 0,0090
$I_{Rel\,1}$[%]	1,381	± 0,095
A_2[kCnts/Chnl]	131	± 15
τ_2[ns]	0,0814	± 0,0017
I_2[kCnts]	2666	± 263
$A_{Rel\,2}$[%]	99,9326	± 0,0090
$I_{Rel\,2}$[%]	98,619	± 0,095
$Bkgr_{Dec}$[kCnts]	0,0008	± 0,0002
$Bkgr_{IRF}$[Cnts/Chnl]	0,71	± 0,24
$Shift_{IRF}$[ps]	-194,0	± 3,6
$\tau_{Av\,Int}$[ns]	0,1037	± 0,0035
$\tau_{Av\,Amp}$[ns]	0,0825	± 0,0018

Figure S23. Left: Time-resolved photoluminescence decay of **2k** (blue) in a THF/water mixture (80/20) fraction at 298 K, including the residuals and the Instrument Response Function (red) (λ_{ex} = 376.7 nm, λ_{em} = 460 nm).

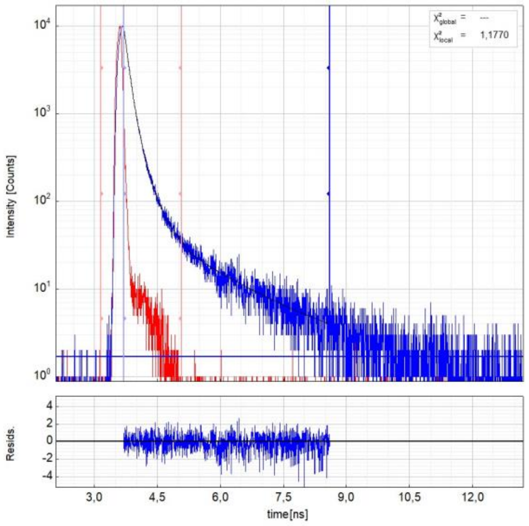

A_1[kCnts/Chnl]	0,0697	± 0,0083
τ_1[ns]	1,47	± 0,16
I_1[kCnts]	25,56	± 0,76
$A_{Rel\,1}$[%]	0,277	± 0,036
$I_{Rel\,1}$[%]	3,55	± 0,16
A_2[kCnts/Chnl]	3,75	± 0,55
τ_2[ns]	0,2136	± 0,0091
I_2[kCnts]	200	± 21
$A_{Rel\,2}$[%]	14,9	± 1,9
$I_{Rel\,2}$[%]	27,8	± 2,7
A_3[kCnts/Chnl]	21,31	± 0,93
τ_3[ns]	0,0929	± 0,0030
I_3[kCnts]	495	± 27
$A_{Rel\,3}$[%]	84,8	± 2,0
$I_{Rel\,3}$[%]	68,7	± 2,7
$Bkgr_{Dec}$[kCnts]	0,0017	± 0,0008
$Bkgr_{IRF}$[Cnts/Chnl]	-1,70	± 0,77
$Shift_{IRF}$[ps]	-56,8	± 3,8
A_{Scatt}[kCnts]	-100	± 52
$\tau_{Av\,Int}$[ns]	0,1752	± 0,0063
$\tau_{Av\,Amp}$[ns]	0,1147	± 0,0019

Figure S24. Left: Time-resolved photoluminescence decay of **2k** (blue) in a THF/water mixture (90/10) fraction at 298 K, including the residuals and the Instrument Response Function (red) (λ_{ex} = 376.7 nm, λ_{em} = 460 nm).

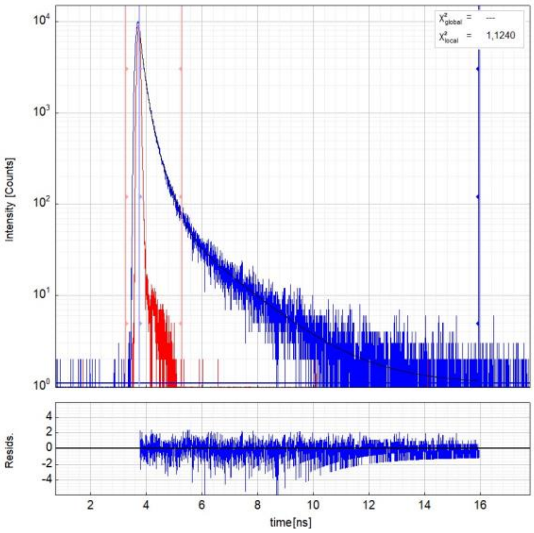

A_1[kCnts/Chnl]	0.109	± 0.011
τ_1[ns]	1.681	± 0.073
I_1[kCnts]	45.7	± 2.5
$A_{Rel\,1}$[%]	1.216	± 0.098
$I_{Rel\,1}$[%]	9.87	± 0.52
A_2[kCnts/Chnl]	6.29	± 0.46
τ_2[ns]	0.131	± 0.018
I_2[kCnts]	206	± 34
$A_{Rel\,2}$[%]	70.5	± 7.3
$I_{Rel\,2}$[%]	44.6	± 7.5
A_3[kCnts/Chnl]	2.53	± 0.99
τ_3[ns]	0.333	± 0.037
I_3[kCnts]	211	± 50
$A_{Rel\,3}$[%]	28.3	± 7.3
$I_{Rel\,3}$[%]	45.6	± 7.5
$Bkgr_{Dec}$[kCnts]	0.0011	± 0.0001
$Bkgr_{IRF}$[Cnts/Chnl]	-3.8	± 4.1
$Shift_{IRF}$[ps]	63.0	± 5.0
A_{Scatt}[kCnts]	84.1	± 4.8
$\tau_{Av\,Int}$[ns]	0.376	± 0.016
$\tau_{Av\,Amp}$[ns]	0.2071	± 0.0090

Figure S25. Left: Time-resolved photoluminescence decay of **2k** (blue) in a THF/water mixture (95/5) fraction at 298 K, including the residuals and the Instrument Response Function (red) (λ_{ex} = 376.7 nm, λ_{em} = 460 nm).

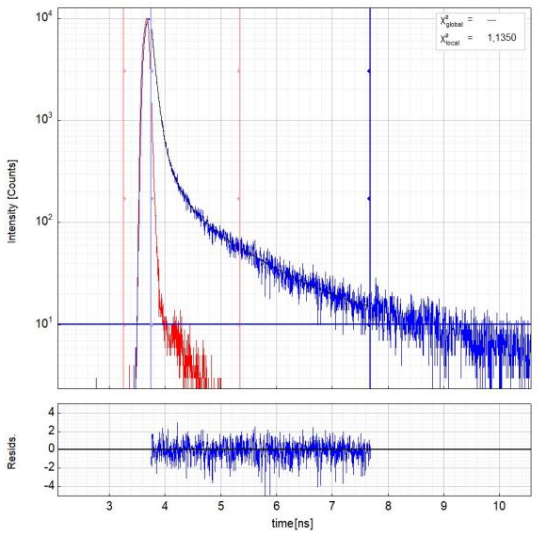

A_1[kCnts/Chnl]	0,233	± 0,045
τ_1[ns]	1,06	± 0,14
I_1[kCnts]	61,6	± 4,7
$A_{Rel\,1}$[%]	0,72	± 0,20
$I_{Rel\,1}$[%]	10,6	± 2,3
A_2[kCnts/Chnl]	2,6	± 1,2
τ_2[ns]	0,165	± 0,032
I_2[kCnts]	106	± 26
$A_{Rel\,2}$[%]	8,0	± 3,7
$I_{Rel\,2}$[%]	18,2	± 6,0
A_3[kCnts/Chnl]	29,4	± 6,2
τ_3[ns]	0,0561	± 0,0062
I_3[kCnts]	413	± 130
$A_{Rel\,3}$[%]	91,3	± 3,9
$I_{Rel\,3}$[%]	71,2	± 8,0
$Bkgr_{Dec}$[kCnts]	0,0101	± 0,0022
$Bkgr_{IRF}$[Cnts/Chnl]	1,4	± 2,3
$Shift_{IRF}$[ps]	-31	± 19
$\tau_{Av\,Int}$[ns]	0,182	± 0,018
$\tau_{Av\,Amp}$[ns]	0,0720	± 0,0036

Figure S26. Left: Time-resolved photoluminescence decay of **2l** (blue) in a THF/water mixture (80/20) fraction at 298 K, including the residuals and the Instrument Response Function (red) (λ_{ex} = 376.7 nm, λ_{em} = 460 nm).

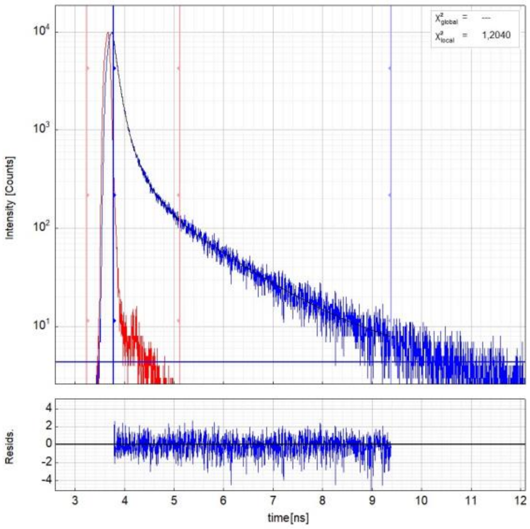

A_1[kCnts/Chnl]	0,329	± 0,040
τ_1[ns]	1,264	± 0,084
I_1[kCnts]	104,1	± 6,1
$A_{Rel\,1}$[%]	1,21	± 0,31
$I_{Rel\,1}$[%]	12,5	± 2,7
A_2[kCnts/Chnl]	3,0	± 1,1
τ_2[ns]	0,246	± 0,038
I_2[kCnts]	185	± 41
$A_{Rel\,2}$[%]	11,0	± 4,2
$I_{Rel\,2}$[%]	22,2	± 5,9
A_3[kCnts/Chnl]	24,0	± 7,0
τ_3[ns]	0,0911	± 0,0072
I_3[kCnts]	546	± 183
$A_{Rel\,3}$[%]	87,8	± 4,5
$I_{Rel\,3}$[%]	65,4	± 8,1
$Bkgr_{Dec}$[kCnts]	0,0044	± 0,0012
$Bkgr_{IRF}$[Cnts/Chnl]	-1,2	± 3,0
$Shift_{IRF}$[ps]	-56	± 33
$\tau_{Av\,Int}$[ns]	0,272	± 0,035
$\tau_{Av\,Amp}$[ns]	0,1223	± 0,0061

Figure S27. Left: Time-resolved photoluminescence decay of **2l** (blue) in a THF/water mixture (90/10) fraction at 298 K, including the residuals and the Instrument Response Function (red) (λ_{ex} = 376.7 nm, λ_{em} = 460 nm).

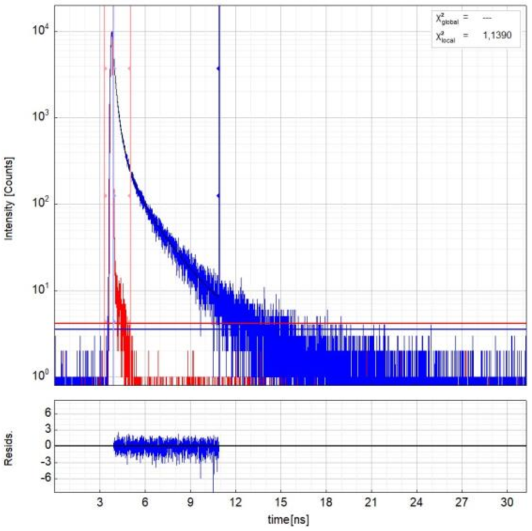

A_1[kCnts/Chnl]	0,319	± 0,083
τ_1[ns]	1,70	± 0,33
I_1[kCnts]	135	± 17
$A_{Rel\,1}$[%]	2,88	± 0,64
$I_{Rel\,1}$[%]	21,1	± 2,5
A_2[kCnts/Chnl]	8,96	± 0,44
τ_2[ns]	0,142	± 0,049
I_2[kCnts]	318	± 111
$A_{Rel\,2}$[%]	81	± 16
$I_{Rel\,2}$[%]	50	± 18
A_3[kCnts/Chnl]	1,8	± 2,6
τ_3[ns]	0,42	± 0,15
I_3[kCnts]	187	± 106
$A_{Rel\,3}$[%]	16	± 16
$I_{Rel\,3}$[%]	29	± 16
$Bkgr_{Dec}$[kCnts]	0,0035	± 0,0028
$Bkgr_{IRF}$[Cnts/Chnl]	4	± 17
$Shift_{IRF}$[ps]	22,4	± 7,8
A_{Scatt}[kCnts]	550	± 610
$\tau_{Av\,Int}$[ns]	0,550	± 0,054
$\tau_{Av\,Amp}$[ns]	0,231	± 0,041

Figure S28. Left: Time-resolved photoluminescence decay of **2l** (blue) in a THF/water mixture (95/5) fraction at 298 K, including the residuals and the Instrument Response Function (red) (λ_{ex} = 376.7 nm, λ_{em} = 460 nm).

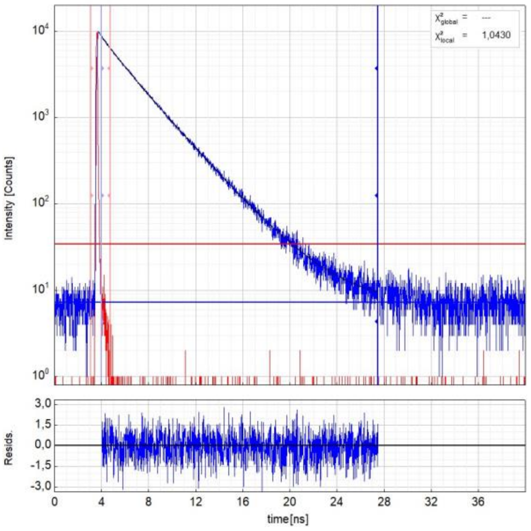

A_1[kCnts/Chnl]	6,68	± 0,33
τ_1[ns]	3,027	± 0,034
I_1[kCnts]	1264	± 49
$A_{Rel\,1}$[%]	63,2	± 3,9
$I_{Rel\,1}$[%]	73,3	± 3,7
A_2[kCnts/Chnl]	3,89	± 0,45
τ_2[ns]	1,898	± 0,082
I_2[kCnts]	461	± 71
$A_{Rel\,2}$[%]	36,8	± 3,9
$I_{Rel\,2}$[%]	26,7	± 3,7
$Bkgr_{Dec}$[kCnts]	0,0073	± 0,0004
$Bkgr_{IRF}$[Cnts/Chnl]	34,1	± 9,2
$Shift_{IRF}$[ps]	-37	± 31
A_{Scatt}[kCnts]	33	± 103
$\tau_{Av\,Int}$[ns]	2,7249	± 0,0055
$\tau_{Av\,Amp}$[ns]	2,611	± 0,014

Figure S29. Left: Time-resolved photoluminescence decay of **2s** (blue) in a THF/water mixture (80/20) fraction at 298 K, including the residuals and the Instrument Response Function (red) (λ_{ex} = 376.7 nm, λ_{em} = 460 nm).

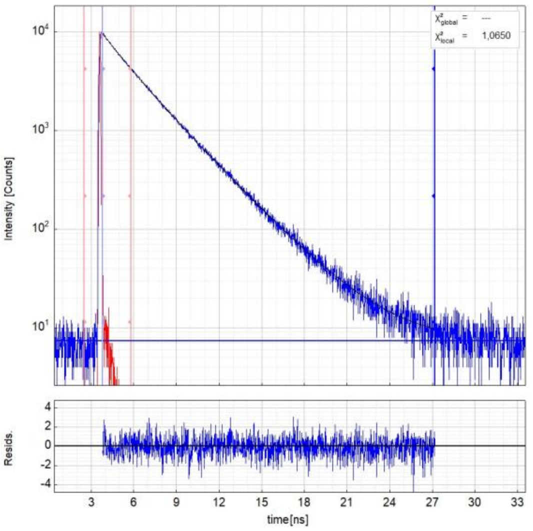

A_1[kCnts/Chnl]	7,27	± 0,44
τ_1[ns]	2,953	± 0,043
I_1[kCnts]	1342	± 61
$A_{Rel\,1}$[%]	69,4	± 4,0
$I_{Rel\,1}$[%]	80,4	± 4,0
A_2[kCnts/Chnl]	3,21	± 0,42
τ_2[ns]	1,63	± 0,13
I_2[kCnts]	327	± 70
$A_{Rel\,2}$[%]	30,6	± 4,0
$I_{Rel\,2}$[%]	19,6	± 4,0
$Bkgr_{Dec}$[kCnts]	0,0073	± 0,0004
$Bkgr_{IRF}$[Cnts/Chnl]	2,5	± 1,6
$Shift_{IRF}$[ps]	-28,6	± 5,7
A_{Scatt}[kCnts]	725	± 176
$\tau_{Av\,Int}$[ns]	2,6939	± 0,0054
$\tau_{Av\,Amp}$[ns]	2,549	± 0,014

Figure S30. Left: Time-resolved photoluminescence decay of **2s** (blue) in a THF/water mixture (90/10) fraction at 298 K, including the residuals and the Instrument Response Function (red) (λ_{ex} = 376.7 nm, λ_{em} = 460 nm).

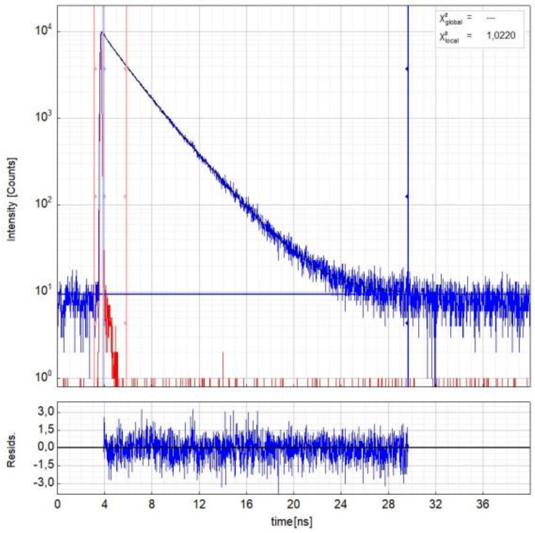

A_1[kCnts/Chnl]	7,41	± 0,42
τ_1[ns]	2,744	± 0,029
I_1[kCnts]	1272	± 85
$A_{Rel\,1}$[%]	70,5	± 2,0
$I_{Rel\,1}$[%]	84,5	± 3,2
A_2[kCnts/Chnl]	3,10	± 0,39
τ_2[ns]	1,20	± 0,23
I_2[kCnts]	233	± 78
$A_{Rel\,2}$[%]	29,5	± 2,0
$I_{Rel\,2}$[%]	15,5	± 3,2
$Bkgr_{Dec}$[kCnts]	0,0093	± 0,0003
$Bkgr_{IRF}$[Cnts/Chnl]	-13	± 13
$Shift_{IRF}$[ps]	25	± 181
$\tau_{Av\,Int}$[ns]	2,505	± 0,014
$\tau_{Av\,Amp}$[ns]	2,288	± 0,066

Figure S31. Left: Time-resolved photoluminescence decay of **2s** (blue) in a THF/water mixture (95/5) fraction at 298 K, including the residuals and the Instrument Response Function (red) (λ_{ex} = 376.7 nm, λ_{em} = 460 nm).

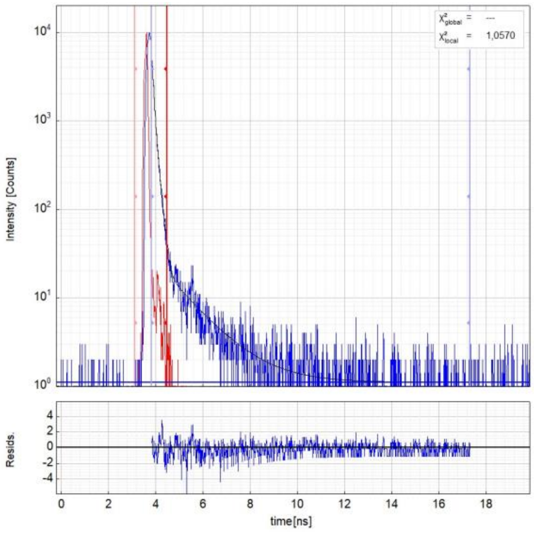

A_1[kCnts/Chnl]	63	± 43
τ_1[ns]	0,0966	± 0,0035
I_1[kCnts]	379	± 242
$A_{Rel\,1}$[%]	99,947	± 0,023
$I_{Rel\,1}$[%]	99,26	± 0,28
A_2[kCnts/Chnl]	0,0333	± 0,0064
τ_2[ns]	1,36	± 0,15
I_2[kCnts]	2,83	± 0,29
$A_{Rel\,2}$[%]	0,053	± 0,023
$I_{Rel\,2}$[%]	0,74	± 0,28
$Bkgr_{Dec}$[kCnts]	0,0011	± 0,0001
$Bkgr_{IRF}$[Cnts/Chnl]	-1,0	± 2,0
$Shift_{IRF}$[ps]	-75	± 43
$\tau_{Av\,Int}$[ns]	0,1060	± 0,0070
$\tau_{Av\,Amp}$[ns]	0,0973	± 0,0037

Figure S32. Left: Time-resolved photoluminescence decay of **2y** (blue) in a THF/water mixture (80/20) fraction at 298 K, including the residuals and the Instrument Response Function (red) (λ_{ex} = 376.7 nm, λ_{em} = 460 nm).

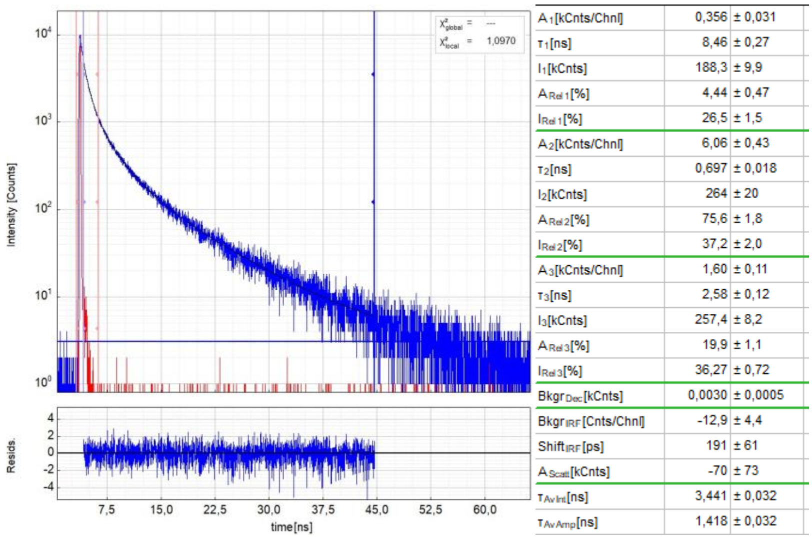

A_1[kCnts/Chnl]	0,356	± 0,031
τ_1[ns]	8,46	± 0,27
I_1[kCnts]	188,3	± 9,9
$A_{Rel\,1}$[%]	4,44	± 0,47
$I_{Rel\,1}$[%]	26,5	± 1,5
A_2[kCnts/Chnl]	6,06	± 0,43
τ_2[ns]	0,697	± 0,018
I_2[kCnts]	264	± 20
$A_{Rel\,2}$[%]	75,6	± 1,8
$I_{Rel\,2}$[%]	37,2	± 2,0
A_3[kCnts/Chnl]	1,60	± 0,11
τ_3[ns]	2,58	± 0,12
I_3[kCnts]	257,4	± 8,2
$A_{Rel\,3}$[%]	19,9	± 1,1
$I_{Rel\,3}$[%]	36,27	± 0,72
$Bkgr_{Dec}$[kCnts]	0,0030	± 0,0005
$Bkgr_{IRF}$[Cnts/Chnl]	-12,9	± 4,4
$Shift_{IRF}$[ps]	191	± 61
A_{Scat}[kCnts]	-70	± 73
$\tau_{Av\,Int}$[ns]	3,441	± 0,032
$\tau_{Av\,Amp}$[ns]	1,418	± 0,032

Figure S33. Left: Time-resolved photoluminescence decay of **2y** (blue) in a THF/water mixture (90/10) fraction at 298 K, including the residuals and the Instrument Response Function (red) (λ_{ex} = 376.7 nm, λ_{em} = 460 nm).

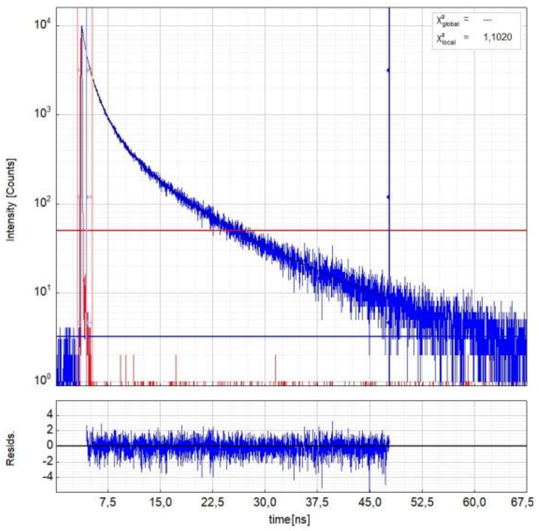

A_1[kCnts/Chnl]	0.41	± 0.12
τ_1[ns]	10.4	± 1.4
I_1[kCnts]	268	± 34
$A_{Rel\,1}$[%]	5.1	± 1.2
$I_{Rel\,1}$[%]	25.7	± 4.6
A_2[kCnts/Chnl]	6.20	± 0.70
τ_2[ns]	1.17	± 0.21
I_2[kCnts]	455	± 131
$A_{Rel\,2}$[%]	76.3	± 8.4
$I_{Rel\,2}$[%]	43.5	± 9.5
A_3[kCnts/Chnl]	1.51	± 0.68
τ_3[ns]	3.42	± 0.84
I_3[kCnts]	323	± 31
$A_{Rel\,3}$[%]	18.6	± 7.2
$I_{Rel\,3}$[%]	30.9	± 4.9
$Bkgr_{Dec}$[kCnts]	0.0032	± 0.0019
$Bkgr_{IRF}$[Cnts/Chnl]	50	± 25
$Shift_{IRF}$[ps]	125	± 82
A_{Scatt}[kCnts]	33	± 53
$\tau_{Av\,Int}$[ns]	4.23	± 0.23
$\tau_{Av\,Amp}$[ns]	2.06	± 0.16

Figure S34. Left: Time-resolved photoluminescence decay of **2y** (blue) in a THF/water mixture (95/5) fraction at 298 K, including the residuals and the Instrument Response Function (red) (λ_{ex} = 376.7 nm, λ_{em} = 460 nm).

IX. X-ray analysis

X-Ray diffraction: Data sets for compounds **2a** and **2ai** were collected with a Bruker D8 Venture PHOTON III diffractometer. Programs used: data collection: APEX3 V2016.1-0[3] (Bruker AXS Inc., **2016**); cell refinement: SAINT V8.37A (Bruker AXS Inc., **2015**); data reduction: SAINT V8.37A (Bruker AXS Inc., **2015**); absorption correction, SADABS V2014/7 (Bruker AXS Inc., **2014**); structure solution *SHELXT-2015*[4] (Sheldrick, G. M. *Acta Cryst.*, **2015**, *A71*, 3-8); structure refinement *SHELXL-2015*[5] (Sheldrick, G. M. *Acta Cryst.*, **2015**, *C71* (1), 3-8) and graphics, *XP*[6] (Version 5.1, Bruker AXS Inc., Madison, Wisconsin, USA, **1998**). *R*-values are given for observed reflections, and wR^2 values are given for all reflections.

X-ray crystal structure analysis of 2a: A colorless prism-like specimen of $C_{19}H_{14}BF_2NO$, approximate dimensions 0.128 mm x 0.223 mm x 0.261 mm, was used for the X-ray crystallographic analysis. The X-ray intensity data were measured on a Bruker D8 Venture PHOTON III Diffractometer system equipped with a micro focus tube Mo Ims (MoK_{α}, λ = 0.71073 Å) and a MX mirror monochromator. A total of 980 frames were collected. The total exposure time was 4.08 hours. The frames were integrated with the Bruker SAINT software package using a narrow-frame algorithm. The integration of the data using a monoclinic unit cell yielded a total of 30583 reflections to a maximum θ angle of 27.49 ° (0.77 Å resolution), of which 3455 were independent (average redundancy 8.852, completeness = 99.2%, R_{int} = 4.71%, R_{sig} = 2.31%) and 3197 (92.53%) were greater than $2\sigma(F^2)$. The final cell constants of a = 10.2026(2) Å, b = 12.0776(2) Å, c = 12.3125(2) Å, β = 92.8040(10) °, volume = 1515.37(5) $Å^3$, are based upon the refinement of the XYZ-centroids of 9943 reflections above 20 σ(I) with 4.727 ° < 2θ < 54.94 °. Data were corrected for absorption effects using the Multi-Scan method (SADABS). The ratio of minimum to maximum apparent transmission was 0.964. The calculated minimum and maximum transmission coefficients (based on crystal size) are 0.9740 and 0.9870. The structure was solved and refined using the Bruker SHELXTL Software Package, using the space group $P2_1/n$, with Z = 4 for the formula unit, $C_{19}H_{14}BF_2NO$. The final anisotropic full-matrix least-squares refinement on F^2 with 217 variables converged at R1 = 3.47%, for the

observed data and wR2 = 8.97% for all data. The goodness-of-fit was 1.033. The largest peak in the final difference electron density synthesis was 0.340 $e^{-}/Å^{3}$ and the largest hole was -0.191 $e^{-}/Å^{3}$ with an RMS deviation of 0.041 $e^{-}/Å^{3}$. On the basis of the final model, the calculated density was 1.408 g/cm^{3} and F(000), 664 e^{-}. CCDC Nr.: 2035686.

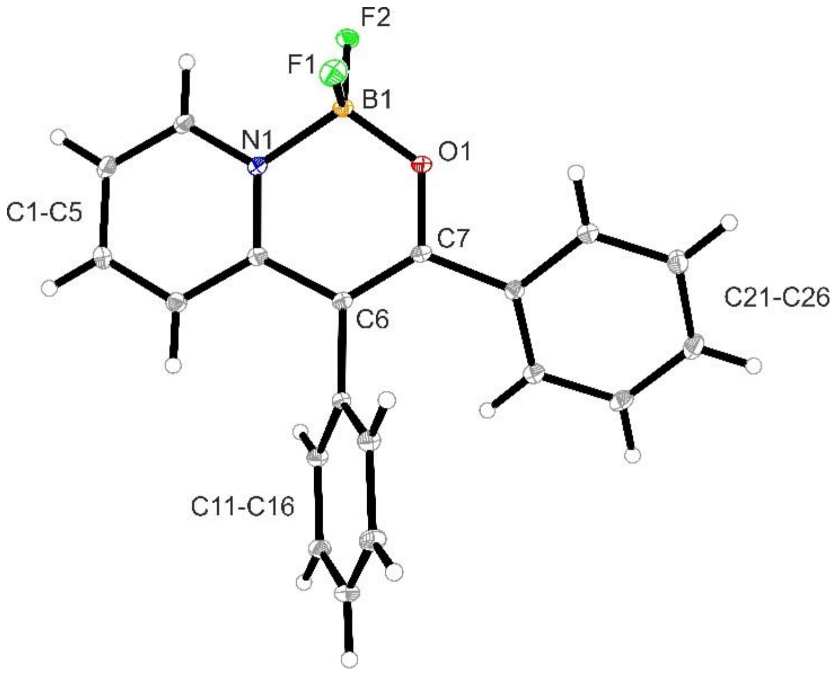

Figure S35a: Crystal structure of compound **2a**. Thermal ellipsoids are shown at 30% probability.

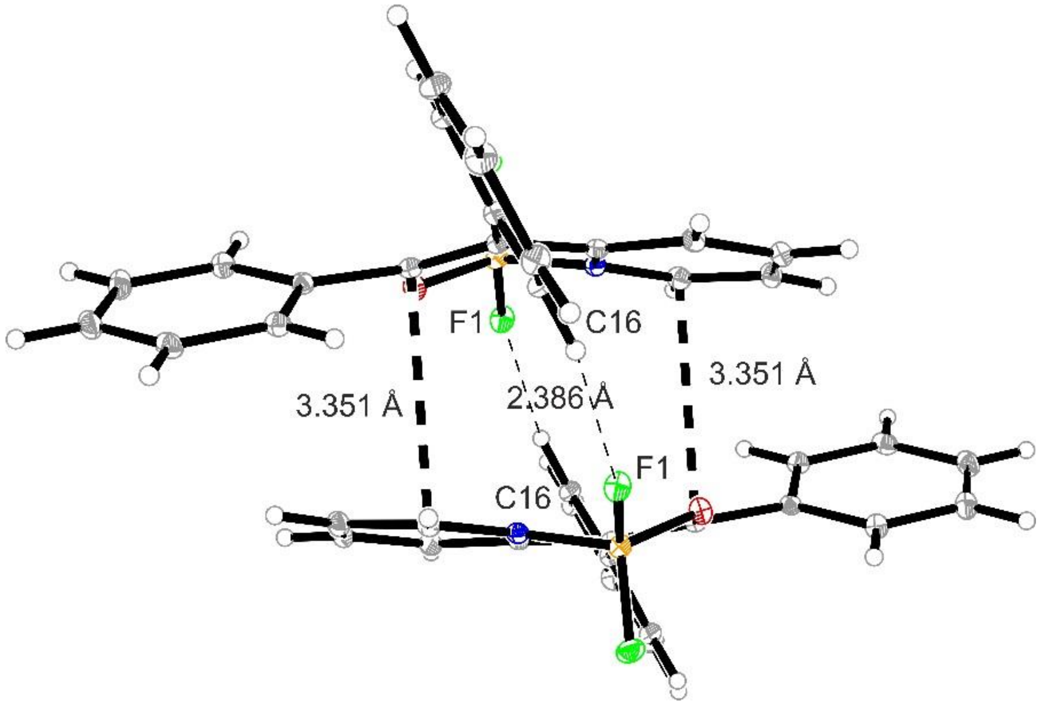

Figure S35b: Dimer formation involving partial parallel offset intermolecular π–π stacking and CH···F interactions.

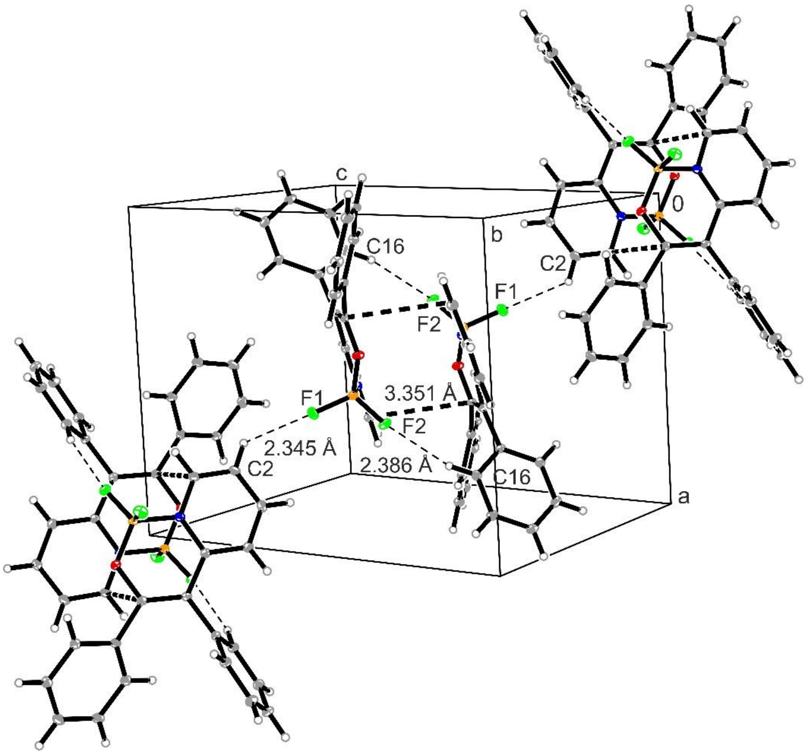

Figure S35c: View perpendicular to *bc*-diagonal presenting the formation of a linear chain between dimers orientated orthogonal.

Table S4. Non-covalent intermolecular interactions in compound **2a** (Å and deg)[a]

D-H···*A*	*d*(*D*-H)	*d*(H···*A*)	*d*(*D*···*A*)	∠(*D*H*A*)
C2-H2···F1$^{\#1}$	0.95	2.345	3.016	127.2
*C*g1···*C*g1$^{\#1, a}$			3.428	
C1···C7$^{\#1, a}$			3.351	
C23-H23···F2$^{\#2}$	0.95	2.619	3.559	170.5
C16-H16···F2$^{\#3}$	0.95	2.386	3.281	156.9
C15-H15···F1$^{\#4}$	0.95	2.508	3.260	136.1
C13-H13···O1$^{\#5}$	0.95	2.582	3.531	176.3

Symmetry transformations used to generate equivalent atoms: $^{\#1}$ -x+0.5, y+0.5, -z+0.5; $^{\#2}$ x+1, y, z; $^{\#3}$ -x+1, -y+1, -z+1; $^{\#4}$ -x+1.5, y+0.5, -z+0.5; $^{\#5}$ x+0.5, -y+0.5, z-0.5; [a] *C*g1 is the centroid involving atoms: C1/N1/C7; The shortest distance between these aromatic units is: C1···C7 3.351 Å.

X-ray crystal structure analysis of 2ai: A colorless plate-like specimen of $C_{27}H_{22}BF_2NO$, approximate dimensions 0.058 mm x 0.125 mm x 0.226 mm, was used for the X-ray crystallographic analysis. The X-ray intensity data were measured on a Bruker D8 Venture PHOTON III Diffractometer system equipped with a micro focus

tube Cu Ims (CuK_α, λ = 1.54178 Å) and a MX mirror monochromator. A total of 1492 frames were collected. The total exposure time was 19.95 hours. The frames were integrated with the Bruker SAINT software package using a wide-frame algorithm. The integration of the data using a monoclinic unit cell yielded a total of 37067 reflections to a maximum θ angle of 68.28° (0.83 Å resolution), of which 3933 were independent (average redundancy 9.425, completeness = 99.5%, R_{int} = 3.67%, R_{sig} = 1.97%) and 3542 (90.06%) were greater than $2\sigma(F^2)$. The final cell constants of a = 10.3655(2) Å, b = 12.2199(2) Å, c = 17.2044(3) Å, β = 98.1420(10)°, volume = 2157.24(7) $Å^3$, are based upon the refinement of the XYZ-centroids of 9827 reflections above 20 σ(I) with 9.411° < 2θ < 136.4°. Data were corrected for absorption effects using the Multi-Scan method (SADABS). The ratio of minimum to maximum apparent transmission was 0.887. The calculated minimum and maximum transmission coefficients (based on crystal size) are 0.8520 and 0.9590. The structure was solved and refined using the Bruker SHELXTL Software Package, using the space group $P2_1/n$, with Z = 4 for the formula unit, $C_{27}H_{22}BF_2NO$. The final anisotropic full-matrix least-squares refinement on F^2 with 291 variables converged at R1 = 3.30%, for the observed data and wR2 = 8.49% for all data. The goodness-of-fit was 1.035. The largest peak in the final difference electron density synthesis was 0.243 $e^-/Å^3$ and the largest hole was -0.225 $e^-/Å^3$ with an RMS deviation of 0.036 $e^-/Å^3$. On the basis of the final model, the calculated density was 1.309 g/cm^3 and F(000), 888 e^-. CCDC Nr.: 2035688.

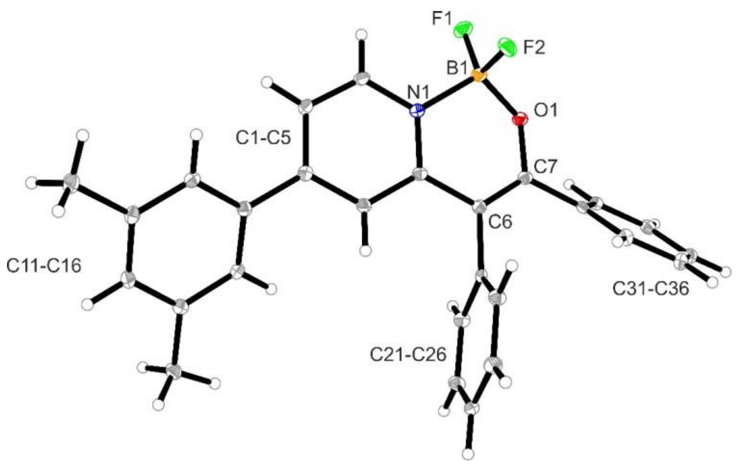

Figure S36a: Crystal structure of compound **2ai**. Thermal ellipsoids are shown at 30% probability.

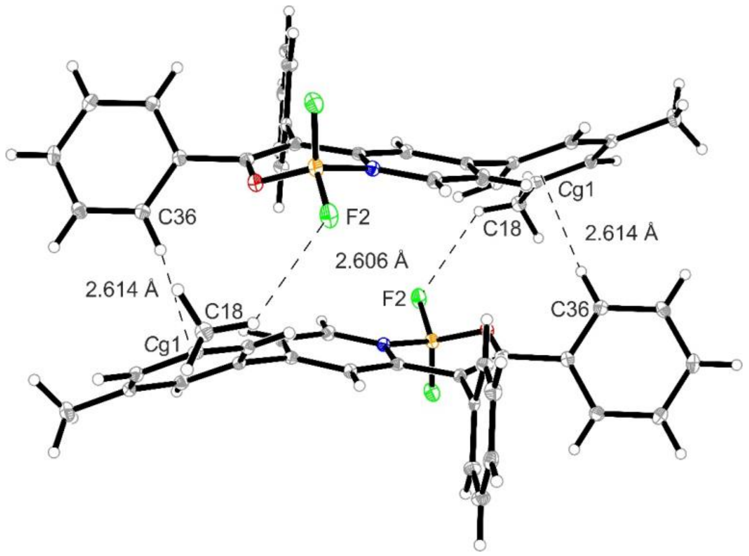

Figure S36b: Dimer formation involving CH$^{\cdots}\pi$ and CH$^{\cdots}$F interactions.

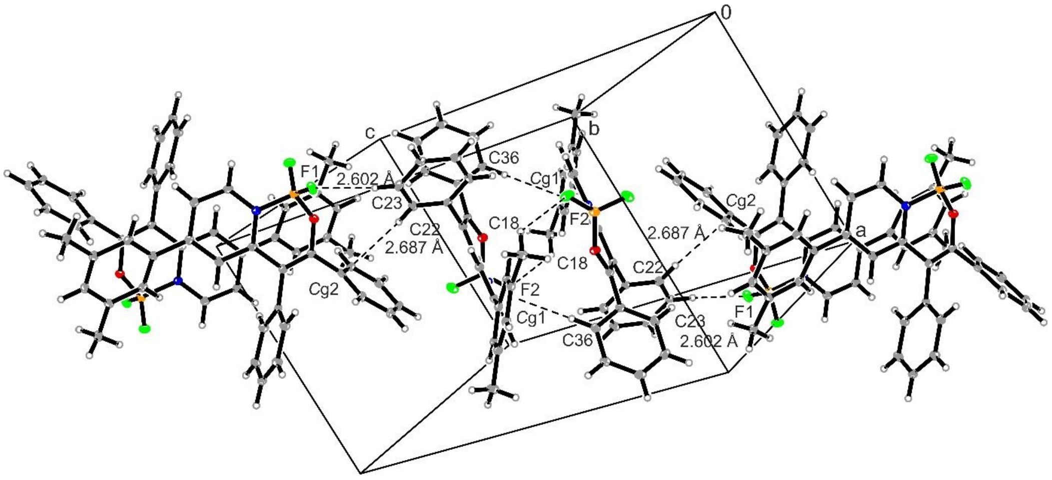

Figure S36c: View perpendicular to *bc*-diagonal presenting the formation of a linear chain between dimers involving additional CH$^{\cdots}\pi$ and CH$^{\cdots}$F interactions.

Table S5. Non-covalent intermolecular interactions in compound **2ai** (Å and deg)[a]

D-H$^{\cdots}$*A*	*d*(*D*-H)	*d*(H$^{\cdots}$*A*)	*d*(*D*$^{\cdots}$*A*)	∠(*D*H*A*)
C36-H36$^{\cdots}$*C*g1[#1, a]	0.95	2.614	3.551	169.0
C18-H18$^{\cdots}$F2[#1]	0.98	2.606	3.351	133.9
*C*g2$^{\cdots}$*C*g2[#2, b]			3.359	
C2-H2$^{\cdots}$*C*g4[#2]	0.95	2.794	3.693	158.2
C22-H22$^{\cdots}$*C*g3[#3]	0.95	2.687	3.626	169.7
C23-H23$^{\cdots}$F1[#3]	0.95	2.602	3.535	167.5
C12-H12$^{\cdots}$F1[#4]	0.95	2.643	3.493	149.2
C17-H17A$^{\cdots}$F1[#4]	0.98	2.588	3.507	156.1

Symmetry transformations used to generate equivalent atoms: #1 -x+1, -y+1, -z+1; #2 -x, -y+1, -x+1; #3 -x+0.5, y+0.5, -z+1.5; #4 -x+1.5, y-0.5, -z+1.5; [a] *C*g1 is the centroid involving atoms: C14/C15/C16; [b] *C*g2 is the centroid involving atoms: C23/C24/C25; [c] *C*g3 is the centroid involving atoms: C31/C36/C35.

X. TDDFT calculation

All computations were carried out using the ORCA 4.1.1 software suite, as published by Neese and co-workers.[7,8] All structures and orbitals were visualized using *IBOView*.[9]

Ground state geometries of compounds **2a**, **2k**, **2l**, **2s**, **2y** and **2aj** were optimized using the range-separated CAM-B3LYP hybrid functional[10] in the def2-TZVPP basis set by Ahlrichs and co-workers,[11] applying Grimme's D3 dispersion correction.[12,13] The obtained structures were confirmed to be local minima by the absence of negative eigenvalues of the Hessian, as obtained from a harmonic frequency calculation. Geometries are given as xyz coordinates of all atoms.

On the optimized geometries, time-dependent density functional theory (TD-DFT) computations were carried out at the same level of theory (CAM-B3LYP D3 / def2-TZVPP), taking into account the first 25 excitations. In all cases, the lowest accessible excitation (by electronic transition dipole moment) is characterized below.

Control computations using different methodologies (M06-2X/def2-TZVPP, PBE0/def2-TZVPP)[14,15] on substrate **2a** have shown very similar HOMO-LUMO energy differences, as well as TDDFT excitation energies.

<u>Substrate **2a**</u>

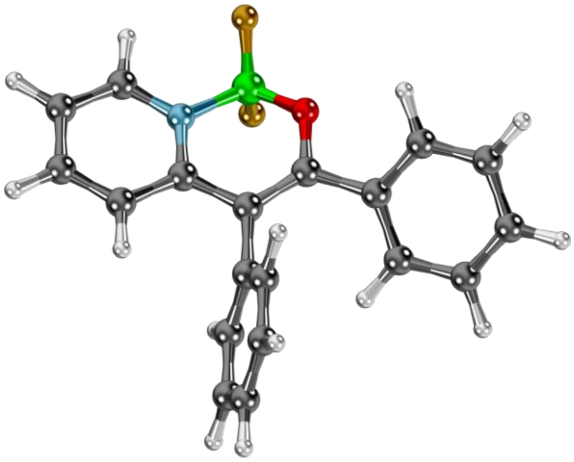

C	0.793566	0.331061	0.731302
C	0.843776	-1.042906	0.958910
C	1.879559	0.920927	0.150949
N	2.968407	0.219776	-0.198636
C	3.057074	-1.110745	0.014641
C	1.959101	-1.755566	0.607798
H	0.006137	-1.549205	1.418676
H	-0.068300	0.922346	0.997478
H	1.930922	1.978019	-0.060513
H	2.015551	-2.815455	0.791770
C	4.280662	-1.802624	-0.315761
C	4.303246	-3.281430	-0.142717
C	3.643389	-4.107744	-1.047525
C	4.953573	-3.867091	0.937149
C	3.644759	-5.482476	-0.885300
C	4.306110	-6.054133	0.189472
C	4.959814	-5.242144	1.100624
H	5.467344	-3.236612	1.649969
H	3.133035	-3.664943	-1.892989
H	3.132464	-6.108870	-1.602748
H	4.311045	-7.127915	0.316117
H	5.477080	-5.679725	1.943431
C	5.391442	-1.072886	-0.636097
C	6.752475	-1.620295	-0.847656
O	5.362279	0.239537	-0.721082
B	4.138545	0.983157	-0.964695
F	4.236344	2.251632	-0.450577
F	3.807080	0.982909	-2.303188
C	7.835366	-0.890298	-0.363747
C	6.992073	-2.795329	-1.550925
C	8.287446	-3.238129	-1.751514
C	9.126850	-1.345785	-0.547884
C	9.357078	-2.521916	-1.242821
H	6.168867	-3.359355	-1.960148
H	8.459712	-4.146538	-2.311890
H	7.653485	0.040649	0.152389
H	9.957343	-0.774781	-0.156425
H	10.368102	-2.873619	-1.395986

Table S6: Electronic Excitation Spectrum of Compound **2a**, as obtained from TDDFT computations (oscillator strengths are computed building on electronic transition dipole moments).

**State Number**	**Wavenumber [cm^{-1}]**	**Wavelength [nm]**	**Oscillator Strength**
1	22096.5	452.6	0.000000000
2	28733.7	348.0	0.000000000
**3**	**32055.1**	**312.0**	**0.567546750**
4	32112.5	311.4	0.000000000
5	32879.2	304.1	0.000000000
6	36071.3	277.2	0.000000000
7	37476.6	266.8	0.000000000
8	37629.8	265.7	0.319373455
9	37974.7	263.3	0.000000001
10	38296.4	261.1	0.000000000
11	38459.8	260.0	0.000000001
12	39453.6	253.5	0.000000002
13	40433.3	247.3	0.000000000
14	40836.7	244.9	0.010855993
15	41025.8	243.7	0.000000000
16	42128.6	237.4	0.000000002
17	42260.5	236.6	0.040728938
18	42411.6	235.8	0.040582659
19	43349.3	230.7	0.000000001
20	43666.5	229.0	0.022246086
21	44708.5	223.7	0.000000001
22	44942.6	222.5	0.018858422
23	46310.1	215.9	0.000000045
24	46452.9	215.3	0.043105517
25	46680.8	214.2	0.000000011

State 3: MO82 → MO83 (c = 0.926054)

MO82

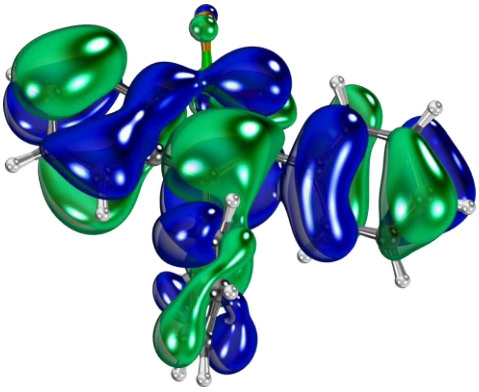

MO83

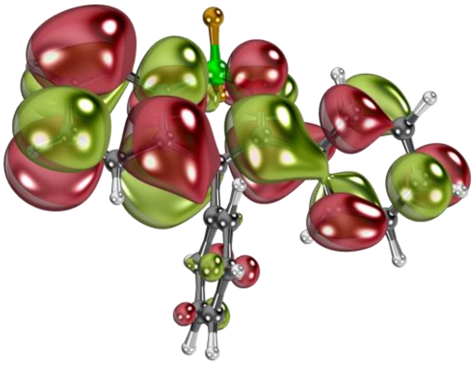

Substrate **2k**

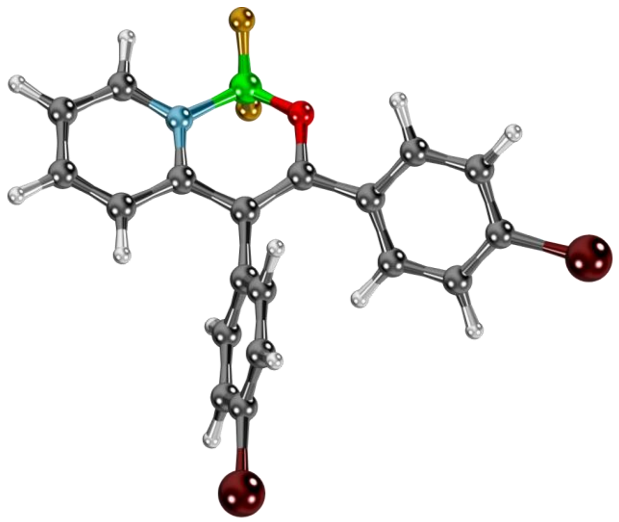

C	0.84573621892046	0.49783810491087	0.75358649960413
C	0.82663136557188	-0.87692519543111	0.97592599331214
C	1.96648725174472	1.03740503793177	0.18930978063967
N	3.02423521989754	0.28587341206913	-0.14942044433405
C	3.04534153705819	-1.04769230892031	0.05950308466477
C	1.91165695928239	-1.64134634801046	0.63566794854381
H	2.07108938000662	2.09160721883095	-0.01693973508072
H	1.91351562465435	-2.70314911934392	0.81732693611608
C	4.24083105882316	-1.79553198645815	-0.25940117067678
C	4.19147131102881	-3.27414562235318	-0.09854086266935
C	3.50343193783809	-4.06338055353730	-1.01458384431265
C	4.80298331526859	-3.90128306358528	0.98022855214352
C	3.43569757531391	-5.43796745072106	-0.87038211819582
C	4.06331471348611	-6.03528406186303	0.20756742803774
C	4.74803131029712	-5.27555375637835	1.13711045220276
H	5.33885677770788	-3.30633535455006	1.70688878367125
H	3.01988256587231	-3.59547616321629	-1.86198552001784
H	2.90383924315403	-6.03942890353217	-1.59251838285707
Br	3.97939310494673	-7.91647003888689	0.41368323806119
H	5.23310081937360	-5.75017168399394	1.97700677521321
C	5.38910548801192	-1.11790931413171	-0.55847604379862
C	6.72504414695806	-1.72762592647208	-0.75708507546772
O	5.42511885610169	0.19458774353201	-0.63199693056022
B	4.24038701122489	0.99426794870486	-0.89904986835345
F	4.38809358624276	2.25678859957866	-0.38621877494179
F	3.93183004143852	1.00064883440936	-2.24191962458865
C	7.83618699317328	-1.04728759701191	-0.26639124318090
C	6.92224006874890	-2.91247941268592	-1.45599180824343

C	8.19506785800955	-3.41895363978524	-1.64560218438469
C	9.10991990420870	-1.55462757483462	-0.43287629429760
C	9.27983465990843	-2.74150061067473	-1.12225622069763
H	6.08306671752583	-3.44265553805462	-1.87752220813768
H	8.34004926371606	-4.33341757979133	-2.20075435172064
H	7.69608849496544	-0.10713443933511	0.24582170457136
H	9.96478713736755	-1.02643895133102	-0.03814818427659
Br	11.02288759639254	-3.43957416048297	-1.36535291684090
H	0.01002518491838	1.12901316651790	1.01138551836479
H	-0.03998029915903	-1.34416371111280	1.42301111248839

Table S7: Electronic Excitation Spectrum of Compound **2k**, as obtained from TDDFT computations (oscillator strengths are computed building on electronic transition dipole moments).

State Number	Wavenumber [cm^{-1}]	Wavelength [nm]	Oscillator Strength
1	22175.7	450.9	0.000000000
2	28881.9	346.2	0.000000000
3	31117.8	321.4	0.000000000
4	31792.6	314.5	0.000000000
**5**	**31987.5**	**312.6**	**0.688074544**
6	36245.9	275.9	0.000000000
7	36417.5	274.6	0.000000000
8	37295.7	268.1	0.000000000
9	37511.2	266.6	0.333280808
10	37759.4	264.8	0.000000000
11	38041.3	262.9	0.000000000
12	38920.4	256.9	0.000000000
13	39305.8	254.4	0.000000000
14	40168.6	249.0	0.038397355
15	40876.5	244.6	0.000000000
16	41232.3	242.5	0.047685234
17	41469.1	241.1	0.000000000
18	41477.3	241.1	0.034631308
19	42571.1	234.9	0.047399360
20	42824.9	233.5	0.000000001
21	43227.6	231.3	0.000000069
22	43407.0	230.4	0.000000020
23	43443.4	230.2	0.068524118
24	43799.9	228.3	0.000000001
25	45108.2	221.7	0.000000003

State 5: MO116 → MO117 (c = 0.907936)
MO114 → MO117 (c = 0.026472)

MO116 MO117

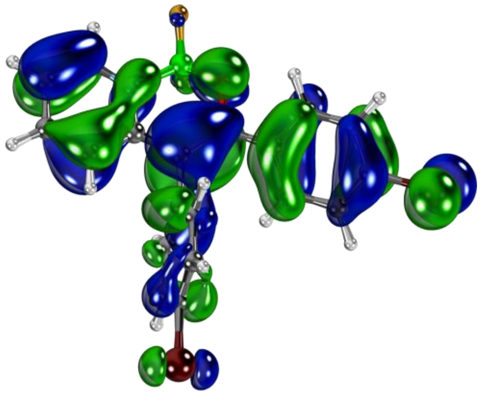

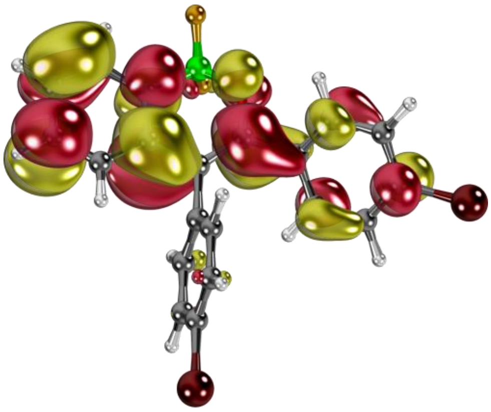

Substrate **2l**

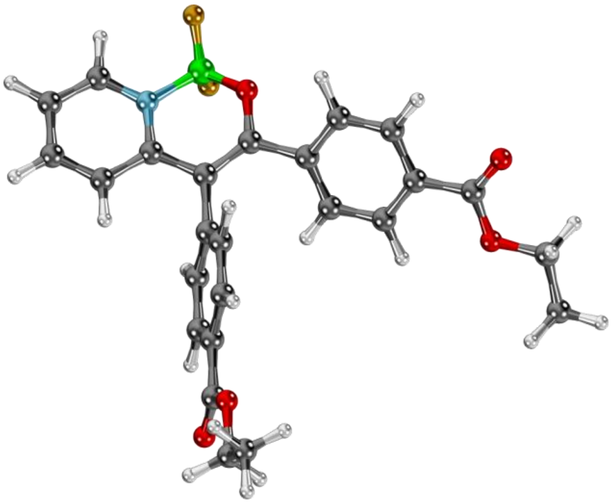

C	1.10050563360618	-0.15106658781972	0.59538643448654
C	1.14423765022833	-1.53194979439122	0.77064484171530
C	2.22136494889190	0.46787171208978	0.11950432521956
N	3.33727054801278	-0.21336810963537	-0.17873831011941
C	3.41886874724948	-1.55063254911677	-0.01346693949201
C	2.28839122803436	-2.22460250796712	0.47284994868053
H	2.27949685603553	1.53303661085681	-0.04428961041755
H	2.33856709949016	-3.29070963024405	0.61906436453236
C	4.67186676442082	-2.21996377948824	-0.28271707039270
C	4.70088749031265	-3.70371449142138	-0.17426881721523
C	4.08476391895863	-4.49471872395880	-1.14124839815451
C	5.31446454529590	-4.33120660805085	0.90398976464720
C	4.09344856637775	-5.87148609205562	-1.03993959948546

C	4.71822795273795	-6.48849883345615	0.03621966980409
C	5.32915655795821	-5.70976698985952	1.00944059631412
H	5.79202581481926	-3.72978844345510	1.66496872347519
H	3.60385753179861	-4.02058392531445	-1.98657017183412
H	3.62340285864911	-6.48903479311688	-1.79159140371563
C	4.70158401493825	-7.97479547823106	0.09724320782380
H	5.81334640169932	-6.18560533187379	1.84853458145299
C	5.79456877510381	-1.47029692842426	-0.48861000591926
C	7.16937427566507	-2.00702083572429	-0.62962158273731
O	5.76524177446454	-0.15655510925581	-0.51866398566629
B	4.55473648142769	0.58811034253820	-0.82779468459994
F	4.60026772181384	1.83565545671900	-0.26220762368194
F	4.32486648794564	0.62997002796895	-2.18558295949132
C	8.20865536735206	-1.33268399645000	0.00677368810536
C	7.46184972565928	-3.11656404814154	-1.41366769318658
C	8.76525018802013	-3.55547432410274	-1.54635206409858
C	9.50654653541134	-1.78384737674560	-0.10766551049033
C	9.79333423969076	-2.89860884037379	-0.88473555814921
H	6.67330675889110	-3.63154231955421	-1.93972603079017
H	8.98796231683068	-4.40940087689516	-2.16732817768686
H	7.98816195051005	-0.44895578538379	0.58679173768730
H	10.31775564252359	-1.27631793297690	0.39340906468079
C	11.21159489888748	-3.33773422450834	-0.98281519959855
H	0.21722233597177	0.42491128333746	0.82196393854037
H	0.27970982721321	-2.06060893753122	1.14788657609277
O	4.17565775063747	-8.67595865256566	-0.72675998574565
O	5.33641721575554	-8.45580230729449	1.17077812212557
C	5.36755213207219	-9.88553673288291	1.30243720191046
C	6.12311244094353	-10.21348556427607	2.56309170815357
H	5.84431403039213	-10.30597404732349	0.41801548003056
H	4.34329026369895	-10.25489035036970	1.33070585754545
H	7.14231852711518	-9.83311052276726	2.51707176487898
H	6.16713000171238	-11.29392079357569	2.69478454748587
H	5.63310075049904	-9.78285030505263	3.43492553216076
O	12.12339517314822	-2.80256460099140	-0.40931625106956
O	11.37101914395821	-4.39880011277764	-1.78099944924785
C	12.71435691843181	-4.88167802389440	-1.93283906645840
C	12.67423785610611	-6.06234561031997	-2.86685949879020
H	13.10156443964941	-5.14936282521962	-0.95053794828331
H	13.33308382889495	-4.07361100424831	-2.32079149411547
H	12.04876400287560	-6.85743592569489	-2.46393747856461
H	13.68047414716536	-6.45594461293426	-3.00567711998507
H	12.28215094404664	-5.77530923579808	-3.84126198836640

Table S8: Electronic Excitation Spectrum of Compound **2l**, as obtained from TDDFT computations (oscillator strengths are computed building on electronic transition dipole moments).

State Number	Wavenumber [cm^{-1}]	Wavelength [nm]	Oscillator Strength
1	22133.7	451.8	0.000000000
2	28791.4	347.3	0.000000000
3	30223.3	330.9	0.000000000
4	30513.8	327.7	0.000000000
5	32038.6	312.1	0.707542892
6	33537.7	298.2	0.000000000
7	35775.9	279.5	0.000000000
8	36669.2	272.7	0.000000000
9	37245.5	268.5	0.292319834
10	37753.5	264.9	0.000000000
11	37900.0	263.9	0.000000000
12	38191.5	261.8	0.139897717
13	38354.3	260.7	0.000000000
14	38873.0	257.2	0.000000000
15	39169.5	255.3	0.000000000
16	39783.0	251.4	0.027431181
17	39785.9	251.3	0.000000000
18	40515.9	246.8	0.000000000
19	41413.7	241.5	0.009232055
20	41689.3	239.9	0.001848191
21	41740.4	239.6	0.000000000
22	42202.9	237.0	0.000000000
23	42434.0	235.7	0.001639527
24	43147.0	231.8	0.005295586
25	43366.6	230.6	0.000000003

State 5: MO120 → MO121 (c = 0.909624)
MO120 → MO122 (c = 0.027604)

MO120

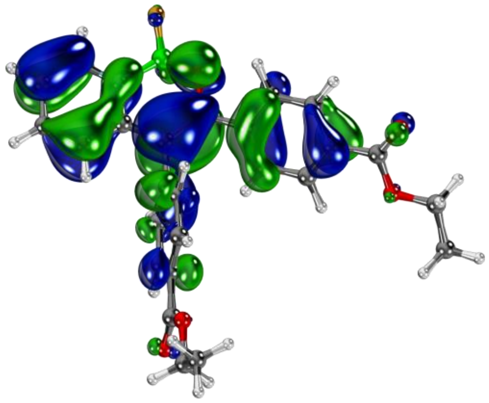

MO121

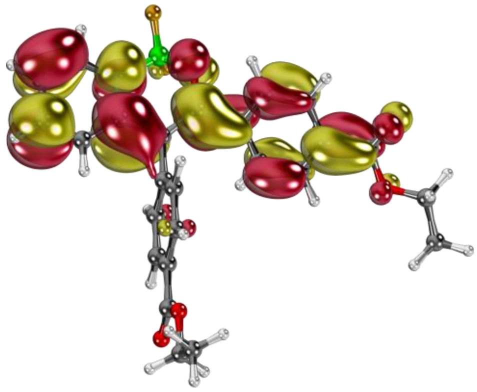

Substrate **2s**

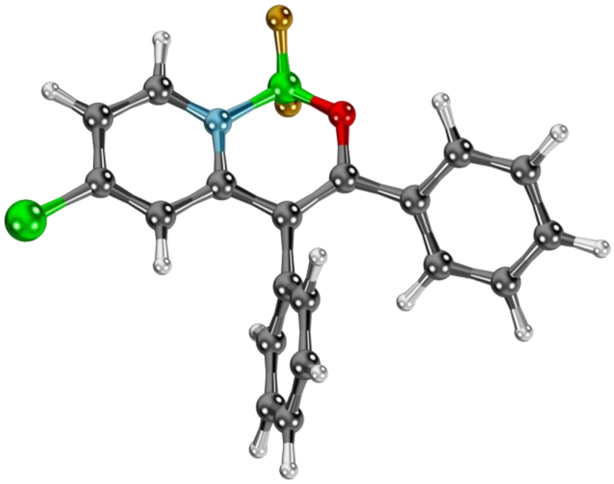

C	1.07022407399937	0.03122925848989	0.61623830357461
C	1.10191394949030	-1.35159943378326	0.78794841018202
C	2.19877037315494	0.62339449631190	0.13046872299201
N	3.30153622771121	-0.07346381411618	-0.17806862020883
C	3.36169939918387	-1.41309813924189	-0.01259721226527
C	2.22504591484743	-2.07129701053323	0.48330775948695
H	2.26838372766075	1.68805363125591	-0.03281826128482
H	2.24948255655272	-3.13721641954301	0.63220351911666
C	4.59276272632479	-2.10907526392434	-0.29229706840005
C	4.58894950879255	-3.59310050291896	-0.16871269015750
C	3.97974446534364	-4.38033511283360	-1.14124548310794
C	5.16200550838576	-4.22150625142911	0.93068192425810
C	3.95562614597967	-5.75950378817871	-1.02503547080246
C	4.54016933524465	-6.37413014190623	0.07024503975317
C	5.14265071258199	-5.60106462899134	1.04810275510962
H	5.63532426843434	-3.62139579783036	1.69581546487153
H	3.52950053307111	-3.90388199619884	-2.00243801182210
H	3.48298880198469	-6.35541434104487	-1.79373242680897
H	5.59904995894346	-6.07267743940860	1.90747979314598
C	5.72969237409106	-1.38150050697836	-0.51987709651456
C	7.09401117738428	-1.94013339661575	-0.66902325788860
O	5.72077918693431	-0.06813320226704	-0.55980090018454
B	4.52346112415744	0.70611250063303	-0.83960504041155
F	4.60527245787878	1.94725883199581	-0.26134405289815
F	4.27189706996464	0.77143569455229	-2.19288530857474
C	8.15456636220579	-1.24007869529784	-0.09887108248343
C	7.36084916312893	-3.09530718238420	-1.39513387630926
C	8.66069927392204	-3.54809116057236	-1.53444655718100
C	9.44927122062592	-1.70658189594363	-0.22200133135402
C	9.70643612977537	-2.86262267650186	-0.94079031224616

H	6.55698936625268	-3.63527573091666	-1.86987324688011
H	8.85544230400388	-4.44015596828859	-2.11332766400428
H	7.95340792932569	-0.32407870776545	0.43645018281400
H	10.26171930290148	-1.15974067764311	0.23603672423449
H	0.19348351699304	0.61104941167075	0.85325794037349
Cl	-0.29202800001316	-2.15664177687214	1.40174001870543
H	4.52450834428663	-7.45134828645613	0.16152757166055
H	10.72065350849394	-3.22256387852393	-1.04603915849025

Table S9: Electronic Excitation Spectrum of Compound **2s**, as obtained from TDDFT computations (oscillator strengths are computed building on electronic transition dipole moments).

State Number	Wavenumber [cm^{-1}]	Wavelength [nm]	Oscillator Strength
1	22096.0	452.6	0.000000000
2	28623.4	349.4	0.000000000
3	31802.1	314.4	0.593407382
4	31999.2	312.5	0.000000000
5	32905.8	303.9	0.000000000
6	35788.0	279.4	0.000000000
7	36298.4	275.5	0.000000000
8	36990.7	270.3	0.324971611
9	37557.1	266.3	0.000000000
10	38158.5	262.1	0.000000000
11	38300.2	261.1	0.000000000
12	39020.2	256.3	0.000000000
13	40518.8	246.8	0.000000000
14	40580.1	246.4	0.014134406
15	40905.9	244.5	0.000000000
16	41920.4	238.5	0.000000000
17	41933.4	238.5	0.025103078
18	42737.7	234.0	0.062674780
19	43434.3	230.2	0.000000000
20	43708.7	228.8	0.017293831
21	44286.1	225.8	0.014354074
22	44718.2	223.6	0.000000001
23	45039.1	222.0	0.000000001
24	45933.6	217.7	0.000000002
25	45966.9	217.5	0.057946818

State 3: MO90 → MO91 (c = 0.922738)

MO90 MO91

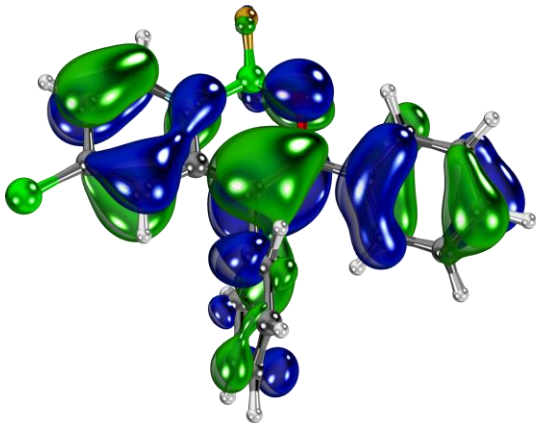

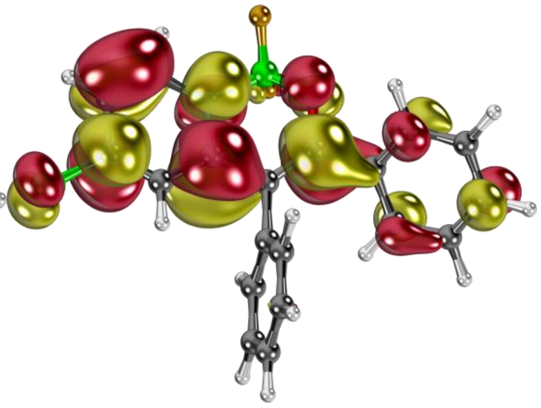

Substrate **2y**

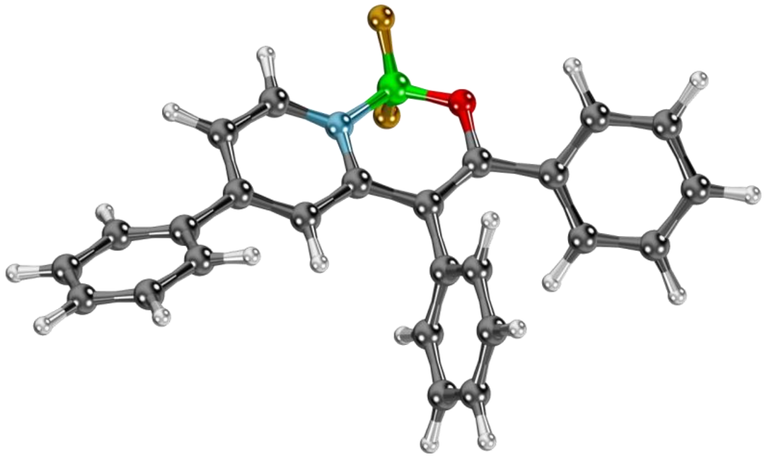

C	0.798242	0.229563	0.729837
C	0.806118	-1.164404	0.879260
C	1.904281	0.843817	0.223123
N	2.999752	0.161542	-0.140775
C	3.062548	-1.180424	-0.008481
C	1.943404	-1.847458	0.506736
C	-0.368647	-1.876467	1.424683
H	1.967862	1.913320	0.092348
H	1.983876	-2.919895	0.597230
C	4.287081	-1.867669	-0.351550
C	4.292231	-3.354110	-0.264110
C	3.644202	-4.121353	-1.227638
C	4.915474	-4.007039	0.793045
C	3.630908	-5.503022	-1.144613
C	4.265730	-6.141640	-0.091749
C	4.907418	-5.389286	0.877288

H	5.420557	-3.423083	1.550356
H	3.154094	-3.626188	-2.055817
H	3.127643	-6.082334	-1.906613
H	4.259539	-7.220947	-0.027202
H	5.404791	-5.879284	1.703117
C	5.412356	-1.134607	-0.604224
C	6.770522	-1.687555	-0.822439
O	5.403571	0.181048	-0.610155
B	4.195473	0.960238	-0.820380
F	4.300802	2.185132	-0.209030
F	3.895776	1.070422	-2.162603
C	7.852381	-1.007183	-0.269072
C	7.009688	-2.817458	-1.596125
C	8.302992	-3.265869	-1.797912
C	9.141347	-1.468973	-0.455508
C	9.370896	-2.600608	-1.220986
H	6.187936	-3.341034	-2.058655
H	8.475075	-4.138298	-2.412896
H	7.671797	-0.109152	0.302866
H	9.970739	-0.937151	-0.010170
H	10.380183	-2.956682	-1.375576
H	-0.052712	0.821256	1.027913
C	-1.658066	-1.458532	1.107570
C	-0.209766	-2.976687	2.262470
C	-1.311531	-3.640133	2.770608
C	-2.589659	-3.219211	2.443761
H	0.783824	-3.302390	2.536917
C	-2.759613	-2.127666	1.608910
H	-1.170742	-4.486476	3.428385
H	-1.801475	-0.617992	0.442836
H	-3.754142	-1.797404	1.343128
H	-3.450699	-3.739798	2.839154

Table S10: Electronic Excitation Spectrum of Compound **2y**, as obtained from TDDFT computations (oscillator strengths are computed building on electronic transition dipole moments).

State Number	Wavenumber [cm^{-1}]	Wavelength [nm]	Oscillator Strength
1	22092.9	452.6	0.000000000
2	28105.2	355.8	0.000000000
3	30745.7	325.2	0.000000000
**4**	**30984.7**	**322.7**	**0.596487154**

5	32182.2	310.7	0.000000001
6	32856.3	304.4	0.000000000
7	36167.6	276.5	0.000000000
8	36580.2	273.4	0.000000002
9	36886.0	271.1	0.000000000
10	37198.1	268.8	0.453837218
11	37382.1	267.5	0.000000003
12	38005.2	263.1	0.000000000
13	38300.9	261.1	0.000000004
14	38577.3	259.2	0.000000001
15	39751.8	251.6	0.000000015
16	40394.1	247.6	0.000000001
17	40560.0	246.5	0.000000000
18	40751.2	245.4	0.025492683
19	40912.8	244.4	0.000000019
20	41869.0	238.8	0.000000008
21	41888.7	238.7	0.345918268
22	41954.5	238.4	0.002303740
23	42120.0	237.4	0.101729286
24	42141.8	237.3	0.140899551
25	43177.3	231.6	0.000000033

State 4: MO102 → MO103 (*c* = 0.919464)

MO102

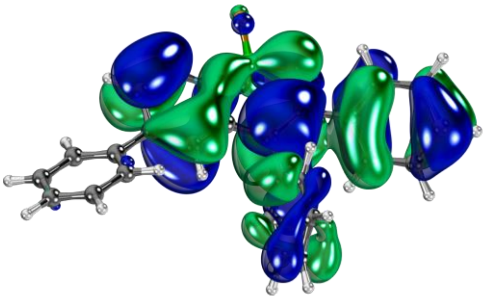

MO103

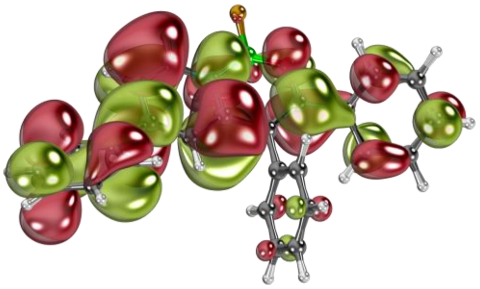

Substrate **2aj**

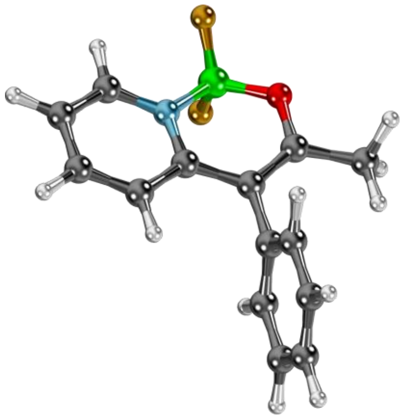

C	0.629528	0.394164	0.793002
C	0.589380	-0.996537	0.880576
C	1.794616	0.974947	0.381007
N	2.880397	0.250520	0.070453
C	2.884235	-1.096625	0.166930
C	1.701184	-1.734345	0.573152
H	-0.316799	-1.495607	1.195516
H	-0.226569	1.005185	1.031791
H	1.913659	2.042617	0.276971
H	1.690206	-2.809548	0.646804
C	4.102098	-1.814070	-0.108888
C	4.070665	-3.303948	-0.104625
C	3.745395	-4.005503	-1.260627
C	4.350381	-4.024865	1.051719
C	3.708742	-5.390107	-1.264230
C	3.994460	-6.095736	-0.107516
C	4.315748	-5.409454	1.051639
H	4.602582	-3.491410	1.958758
H	3.525810	-3.456439	-2.166608
H	3.459565	-5.918486	-2.174273
H	3.968278	-7.176673	-0.109616
H	4.541603	-5.952936	1.958917
C	5.262527	-1.122101	-0.286845
C	6.603348	-1.749135	-0.455965
H	7.275529	-1.355379	0.306433
H	7.011023	-1.453742	-1.422971
H	6.573153	-2.830670	-0.391428
O	5.313582	0.189668	-0.296908
B	4.142076	1.016145	-0.538207
F	4.261297	2.224514	0.100463
F	3.903141	1.159864	-1.889905

Table S11: Electronic Excitation Spectrum of Compound **2aj**, as obtained from TDDFT computations (oscillator strengths are computed building on electronic transition dipole moments).

State Number	Wavenumber [cm^{-1}]	Wavelength [nm]	Oscillator Strength
1	23480.9	425.9	0.000000000
2	29326.9	341.0	0.000000000
3	33451.3	298.9	0.000000000
**4**	**33659.8**	**297.1**	**0.290624561**
5	38777.1	257.9	0.000000000
6	38856.6	257.4	0.000000000

7	39046.1	256.1	0.000000000
8	40191.1	248.8	0.000000000
9	40361.9	247.8	0.341510849
10	41434.8	241.3	0.000000000
11	43248.4	231.2	0.003774348
12	43658.8	229.0	0.000000000
13	44004.3	227.3	0.003618629
14	44275.2	225.9	0.000000000
15	45700.1	218.8	0.000000000
16	45834.7	218.2	0.001982780
17	46397.5	215.5	0.000000000
18	46876.7	213.3	0.000841671
19	47777.8	209.3	0.022491823
20	48732.6	205.2	0.000000000
21	48961.8	204.2	0.000000000
22	49493.5	202.0	0.000000000
23	50472.8	198.1	0.010662791
24	50628.0	197.5	0.014809723
25	51054.2	195.9	0.000000000

State 4: MO66 → MO67 (c = 0.928106)
MO66 → MO68 (c = 0.021890)

MO66

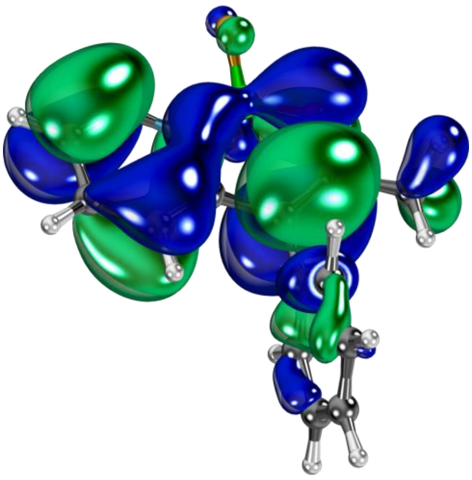

MO67

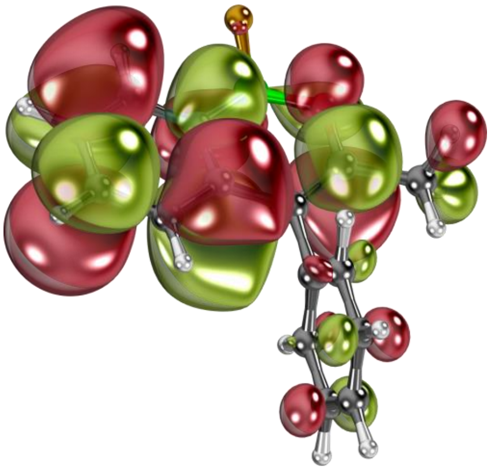

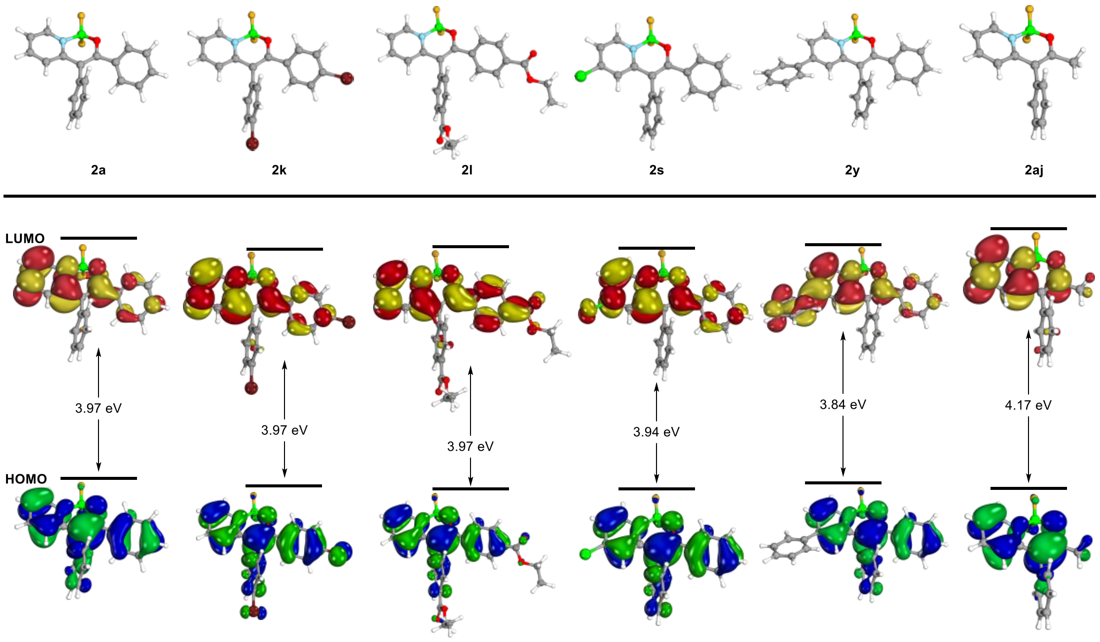

Figure S37. Computed frontier molecular orbitals and TD-DFT gaps (CAM-B3LYP D3 / def2-TZVPP) for selected N,O-bidentate BF_2 complexes with different substitution patterns.

XI. References

[1] a) H. E. Ho, N. Asao, Y. Yamamoto, T. Jin, *Org. Lett.* **2014**, *16*, 4670; b) D. Zhao, J. H. Kim, L. Stegemann, C. A. Strassert, Frank Glorius, *Angew. Chem. Int. Ed.* **2015**, *54*, 4508; *Angew. Chem.* **2015**, *127*, 4591; c) G. D. Kortman, K. L. Hull, *ACS Catal.* **2017**, *7*, 6220.

[2] L. Pitzer, F. Schäfers, F. Glorius, *Angew. Chem. Int. Ed.* **2019**, *58*, 8572; *Angew. Chem.* **2019**, *131*, 8660.

[3] *APEX3* (**2016**), *SAINT* (**2015**) and *SADABS* (**2015**), Bruker AXS Inc., Madison, Wisconsin, USA.

[4] G. M. Sheldrick, *SHELXT–Integrated space-group and crystal-structure determination, Acta Cryst.*, **2015**, *A71*, 3.

[5] Sheldrick, G.M., *Crystal structure refinement with SHELXL, Acta Cryst.*, **2015**, *C71 (1)*, 3.

[6] *XP–Interactive molecular graphics, Version 5.1*, Bruker AXS Inc., Madison, Wisconsin, USA, **1998**.

[7] F. Neese, *Wiley Interdiscip. Rev. Comput. Mol. Sci.* **2012**, *2*, 73.

[8] F. Neese, *Wiley Interdiscip. Rev. Comput. Mol. Sci.* **2018**, *8*, DOI 10.1002/wcms.1327.

[9] "IboView -- A program for chemical analysis.", http://www.iboview.org/.

[10] T. Yanai, D. P. Tew, N. C. Handy, *Chem. Phys. Lett.* **2004**, *393*, 51.

[11] F. Weigend, R. Ahlrichs, *Phys. Chem. Chem. Phys.* **2005**, *7*, 3297.

[12] S. Grimme, J. Antony, S. Ehrlich, H. Krieg, *J. Chem. Phys.* **2010**, *132*, 241722.

[13] S. Grimme, S. Ehrlich, L. Goerigk, *J. Comput. Chem.* **2011**, *32*, 1456.

[14] Y. Zhao, D. G. Truhlar, *Theor. Chem. Acc.* **2008**, *120*, 215.

[15] C. Adamo, V. Barone, *J. Chem. Phys.* **1999**, *110*, 6158.

XII. Copies of ^{1}H and ^{13}C NMR spectra

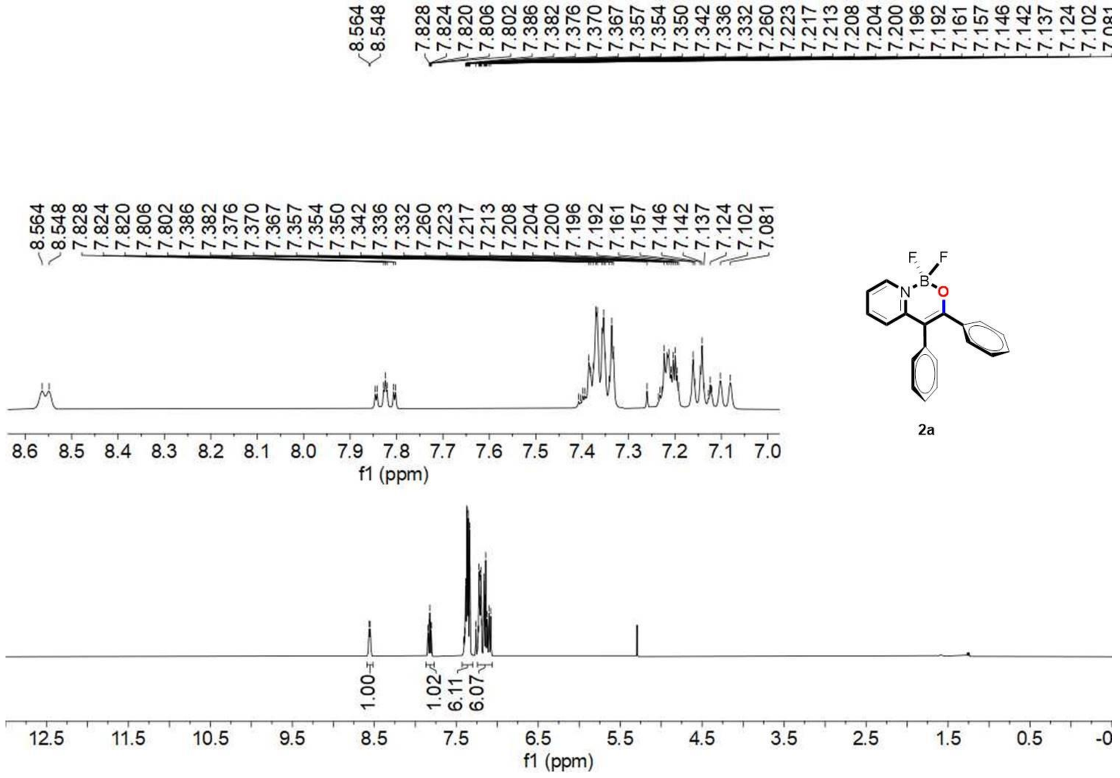

2a

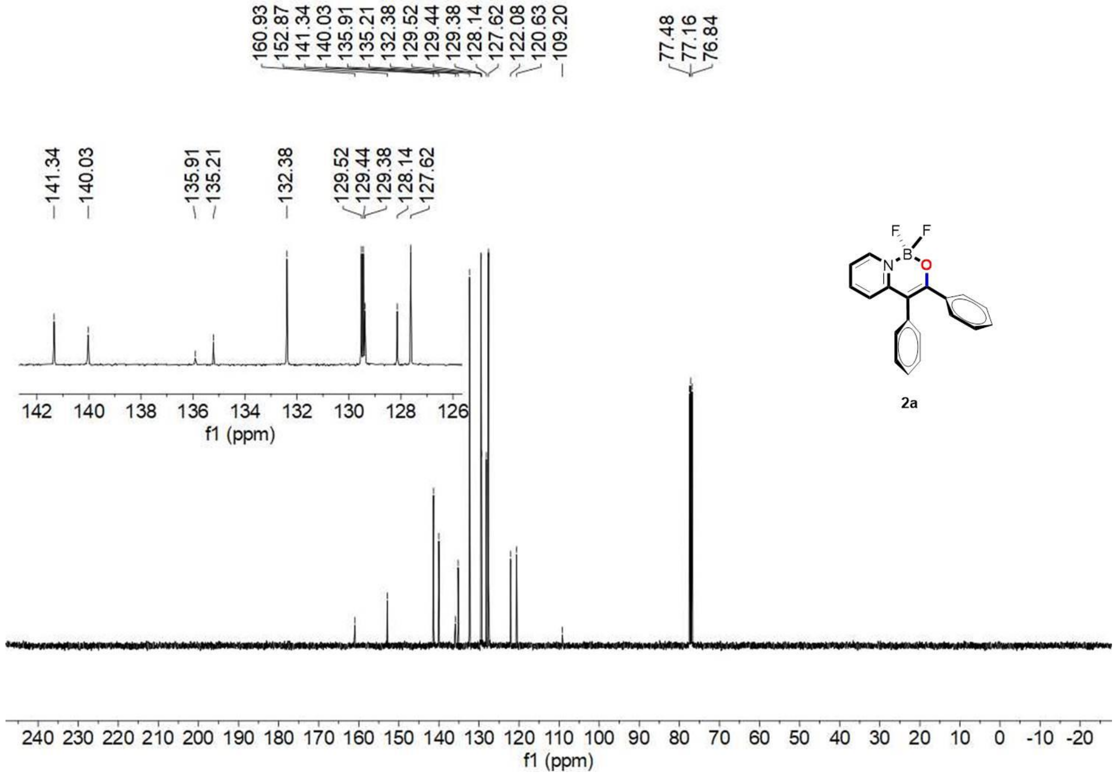

2a

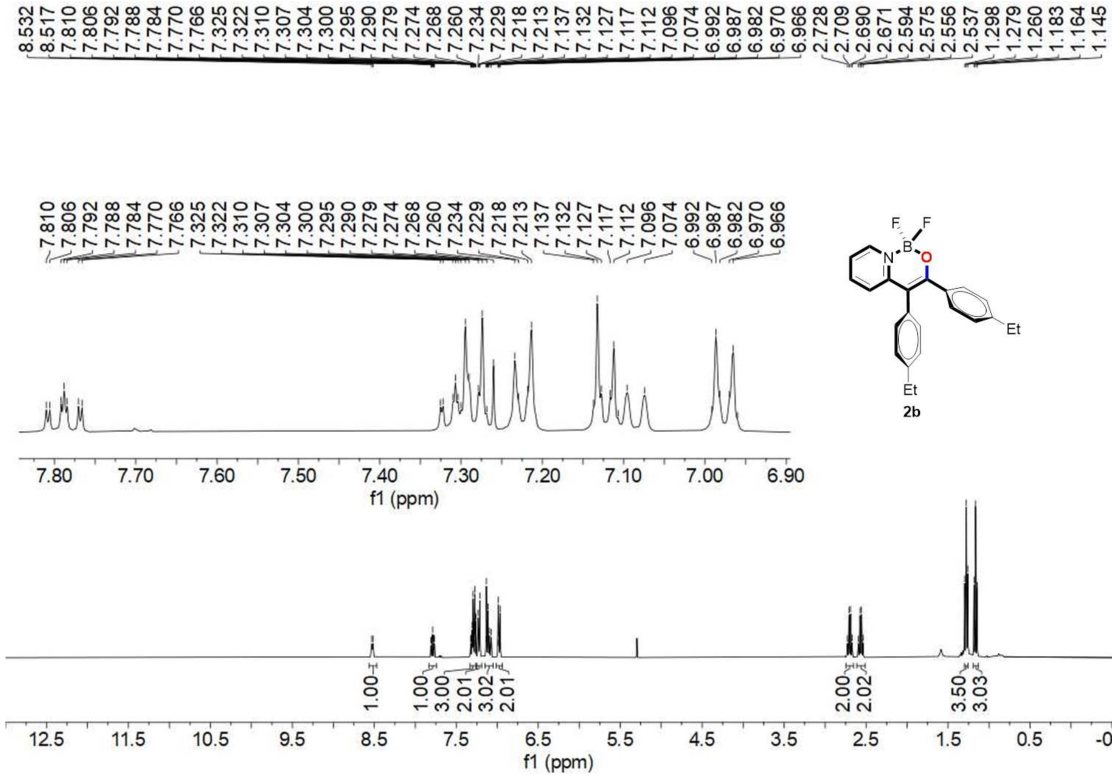
8.532
8.517
7.810
7.806
7.792
7.788
7.784
7.770
7.766
7.325
7.322
7.310
7.307
7.304
7.300
7.295
7.290
7.279
7.274
7.268
7.260
7.234
7.229
7.218
7.213
7.137
7.132
7.127
7.117
7.112
7.096
7.074
6.992
6.987
6.982
6.970
6.966
2.728
2.709
2.690
2.671
2.594
2.575
2.556
2.537
1.298
1.279
1.260
1.183
1.164
1.145
Et
2b
f1 (ppm)
1.00
1.00
3.00
2.01
3.02
2.01
2.00
2.02
3.50
3.03

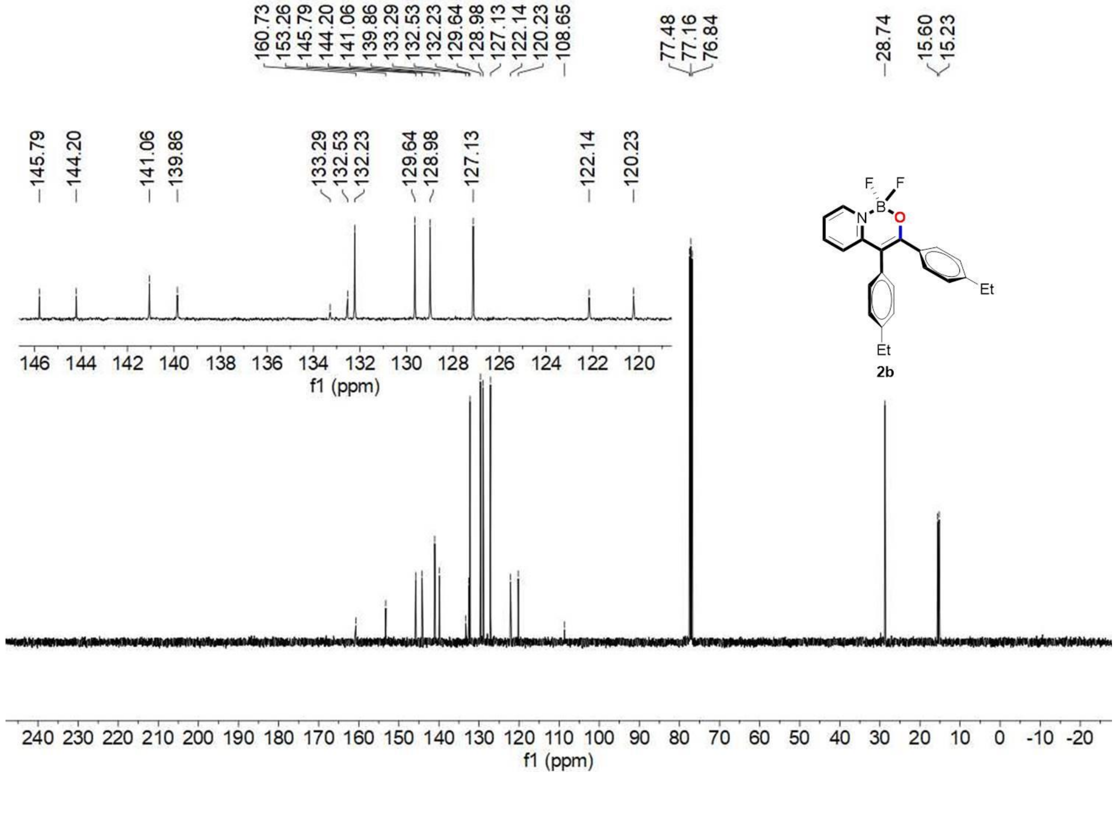
160.73
153.26
145.79
144.20
141.06
139.86
133.29
132.53
132.23
129.64
128.98
127.13
122.14
120.23
108.65
77.48
77.16
76.84
28.74
15.60
15.23
Et
2b
f1 (ppm)

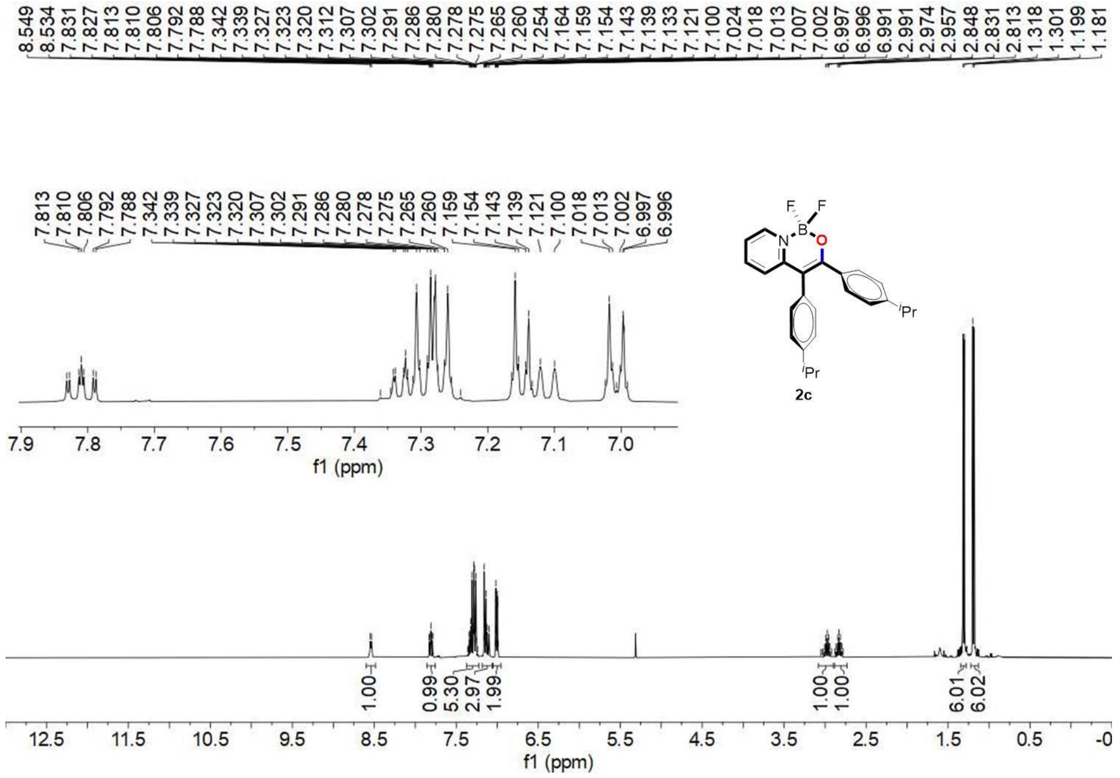

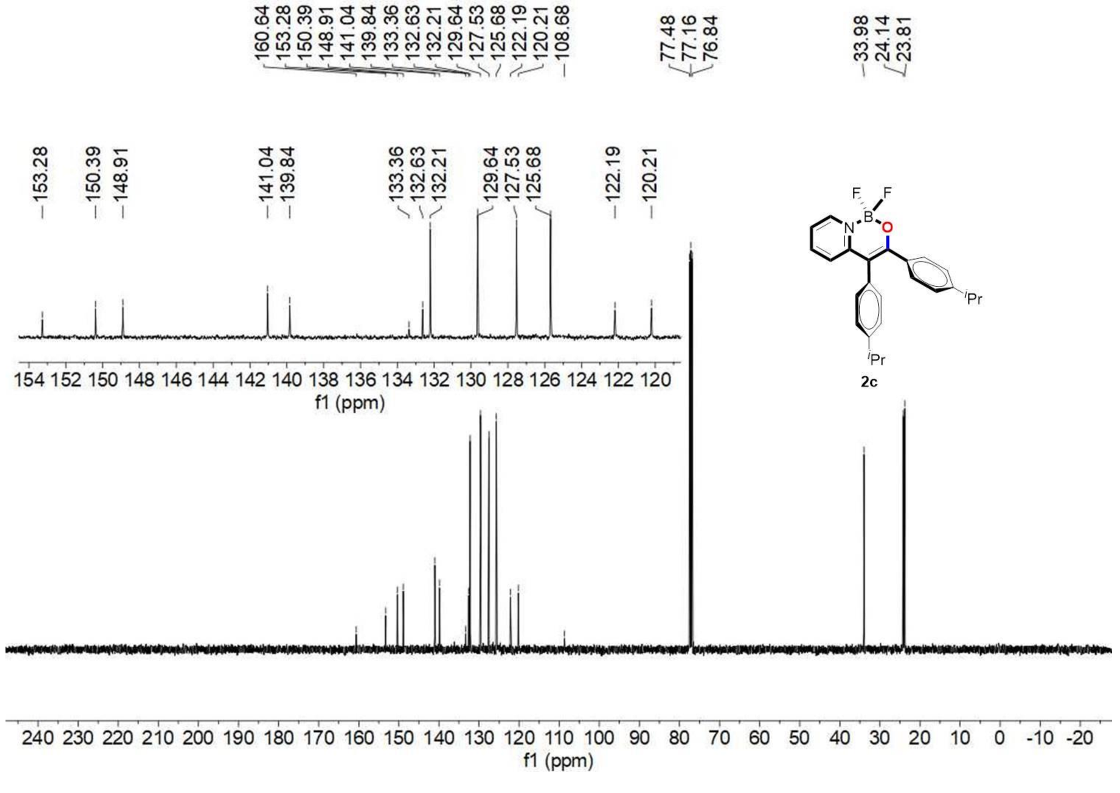

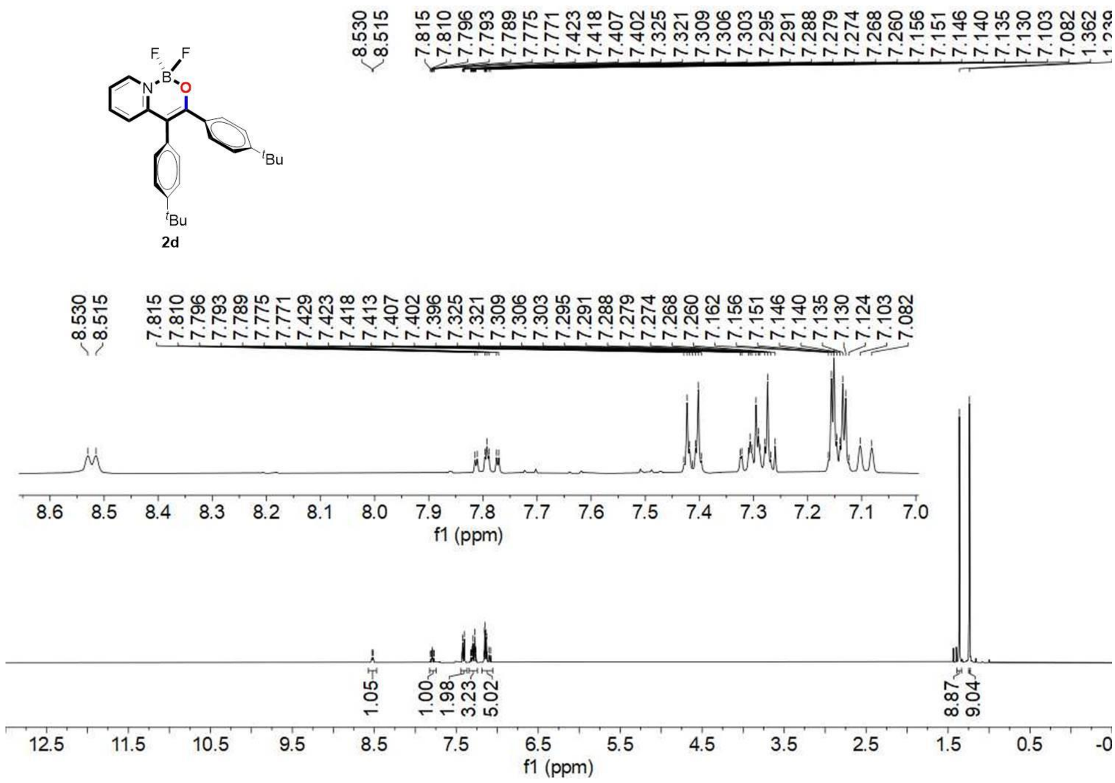

F
F
N
B
O
tBu
tBu
8.530
8.515
7.815
7.810
7.796
7.793
7.789
7.775
7.771
7.429
7.423
7.418
7.413
7.407
7.402
7.396
7.325
7.321
7.309
7.306
7.303
7.295
7.291
7.288
7.279
7.274
7.268
7.260
7.162
7.156
7.151
7.146
7.140
7.135
7.130
7.124
7.103
7.082
1.362
8.6 8.5 8.4 8.3 8.2 8.1 8.0 7.9 7.8 7.7 7.6 7.5 7.4 7.3 7.2 7.1 7.0
f1 (ppm)
1.05
1.00
1.98
3.23
5.02
8.87
9.04
12.5 11.5 10.5 9.5 8.5 7.5 6.5 5.5 4.5 3.5 2.5 1.5 0.5
f1 (ppm)

2d

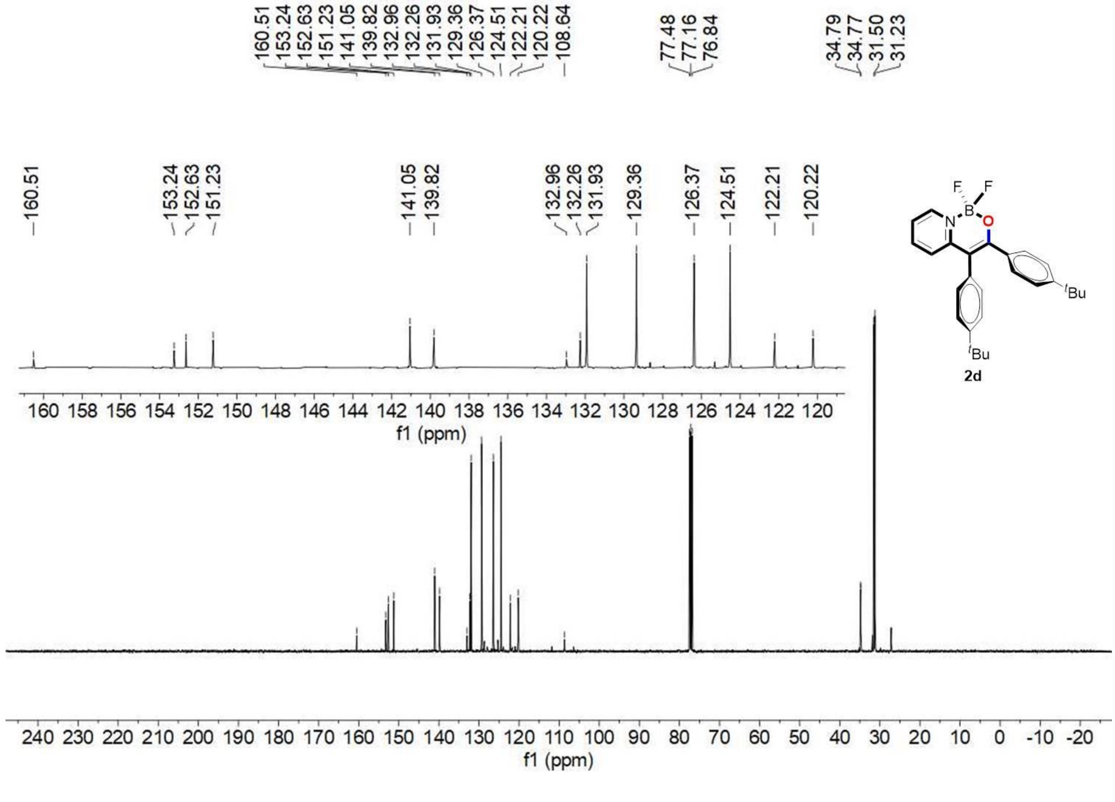

160.51
153.24
152.63
151.23
141.05
139.82
132.96
132.26
131.93
129.36
126.37
124.51
122.21
120.22
108.64
77.48
77.16
76.84
34.79
34.77
31.50
31.23
160 158 156 154 152 150 148 146 144 142 140 138 136 134 132 130 128 126 124 122 120
f1 (ppm)
F
F
N
B
O
tBu
tBu
240 230 220 210 200 190 180 170 160 150 140 130 120 110 100 90 80 70 60 50 40 30 20 10 0 -10 -20
f1 (ppm)

2d

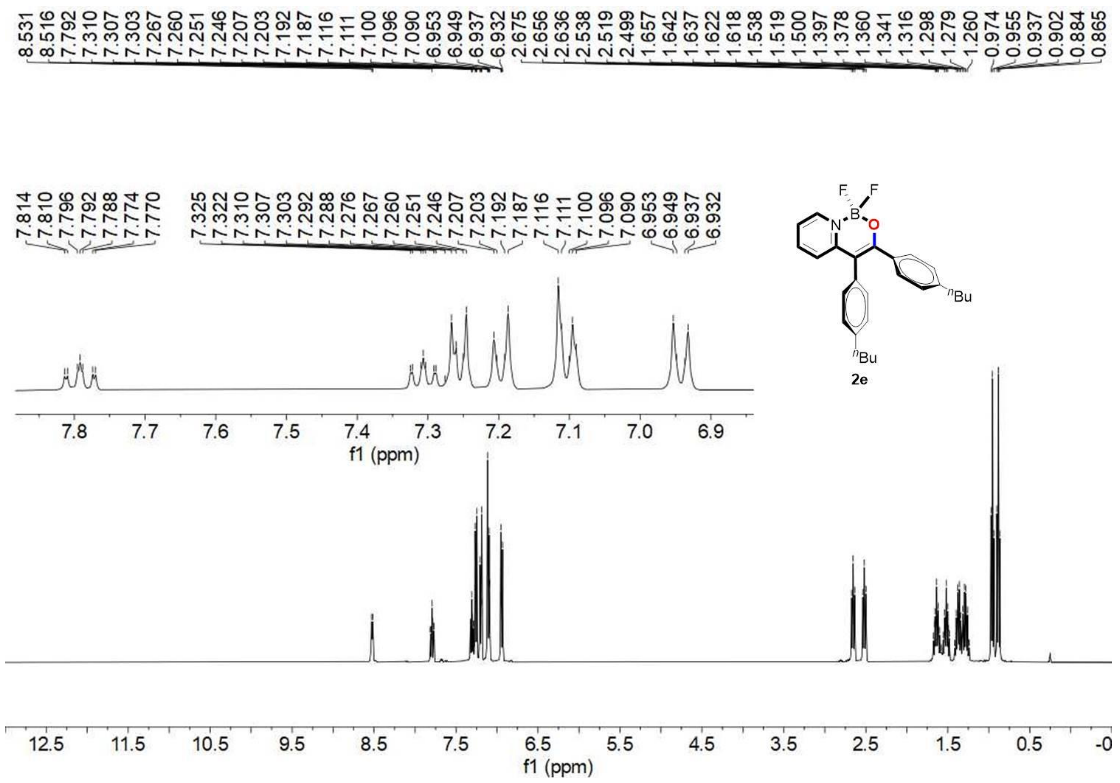
8.531 8.516 7.792 7.310 7.307 7.303 7.267 7.260 7.251 7.246 7.207 7.203 7.192 7.187 7.116 7.111 7.100 7.096 7.090 6.953 6.949 6.937 6.932 2.675 2.656 2.636 2.538 2.519 2.499 1.657 1.642 1.637 1.622 1.618 1.538 1.519 1.500 1.397 1.378 1.360 1.341 1.316 1.298 1.279 1.260 0.974 0.955 0.937 0.902 0.884 0.865
7.814 7.810 7.796 7.792 7.788 7.774 7.770 7.325 7.322 7.310 7.307 7.303 7.292 7.288 7.276 7.267 7.260 7.251 7.246 7.207 7.203 7.192 7.187 7.116 7.111 7.100 7.096 7.090 6.953 6.949 6.937 6.932
F F B N O
ⁿBu
ⁿBu
2e
f1 (ppm)
f1 (ppm)

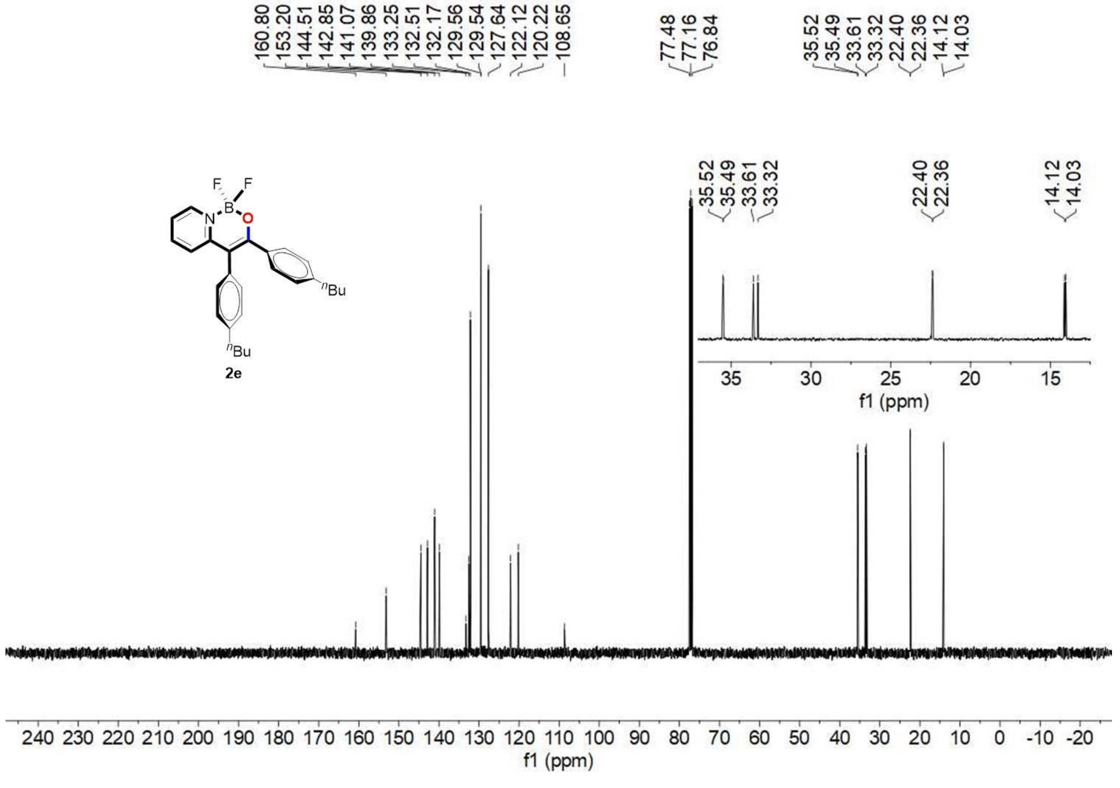
160.80 153.20 144.51 142.85 141.07 139.86 133.25 132.51 132.17 129.56 129.54 127.64 122.12 120.22 108.65
77.48 77.16 76.84
35.52 35.49 33.61 33.32 22.40 22.36 14.12 14.03
F F B N O
ⁿBu
ⁿBu
2e
f1 (ppm)
f1 (ppm)

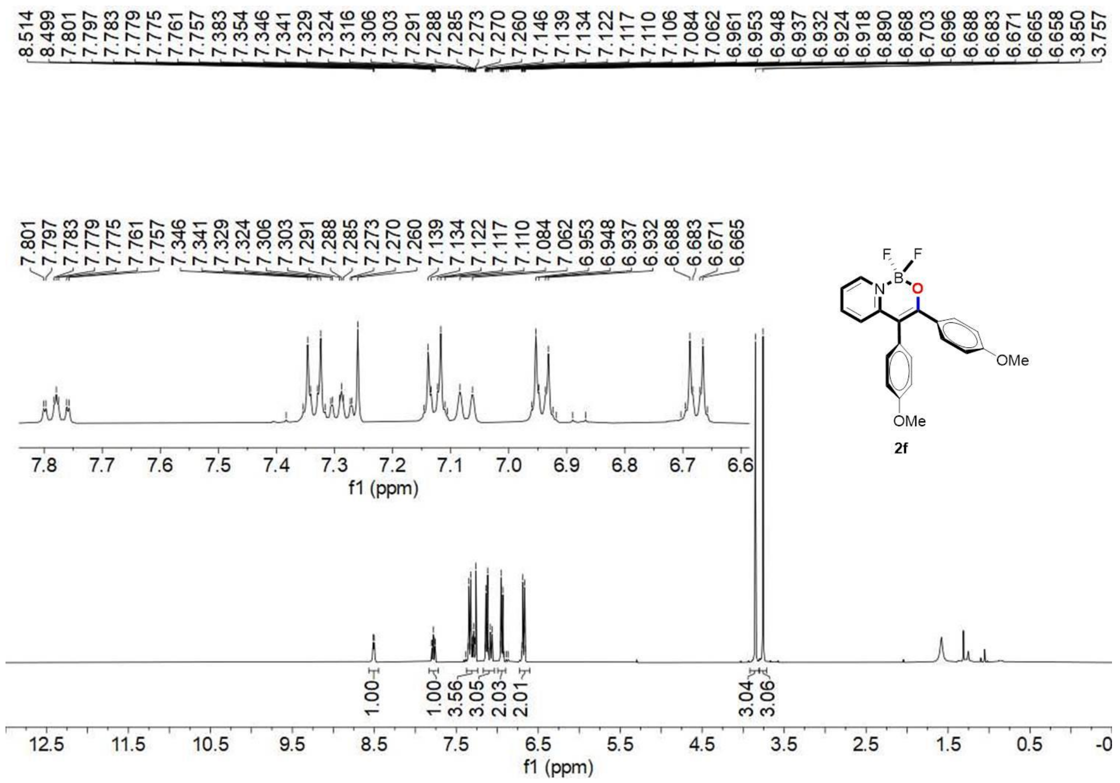
8.514
8.499
7.801
7.797
7.783
7.779
7.775
7.761
7.757
7.383
7.354
7.346
7.341
7.329
7.324
7.316
7.306
7.303
7.291
7.288
7.285
7.273
7.270
7.260
7.146
7.139
7.134
7.122
7.117
7.110
7.106
7.084
7.062
6.961
6.953
6.948
6.937
6.932
6.924
6.918
6.890
6.868
6.703
6.696
6.688
6.683
6.671
6.665
6.658
3.850
3.757
OMe
OMe
2f
1.00
1.00
3.56
3.05
2.03
2.01
3.04
3.06
f1 (ppm)
f1 (ppm)

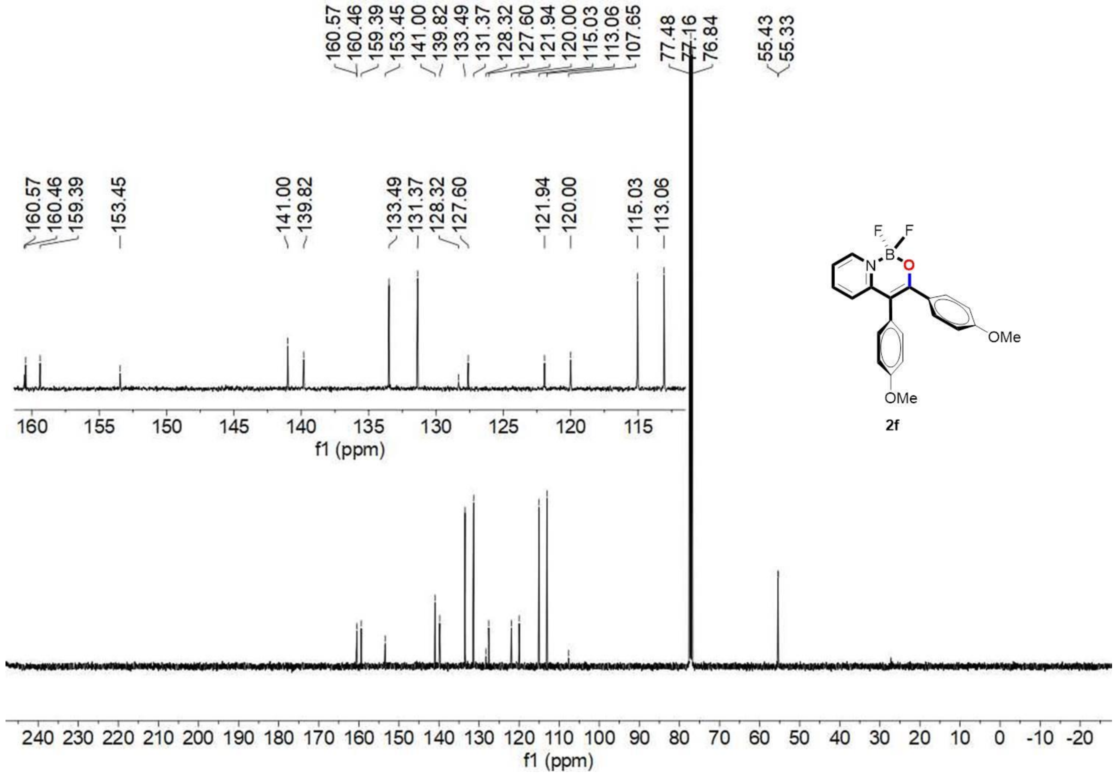
160.57
160.46
159.39
153.45
141.00
139.82
133.49
131.37
128.32
127.60
121.94
120.00
115.03
113.06
107.65
77.48
77.16
76.84
55.43
55.33
OMe
OMe
2f
f1 (ppm)
f1 (ppm)

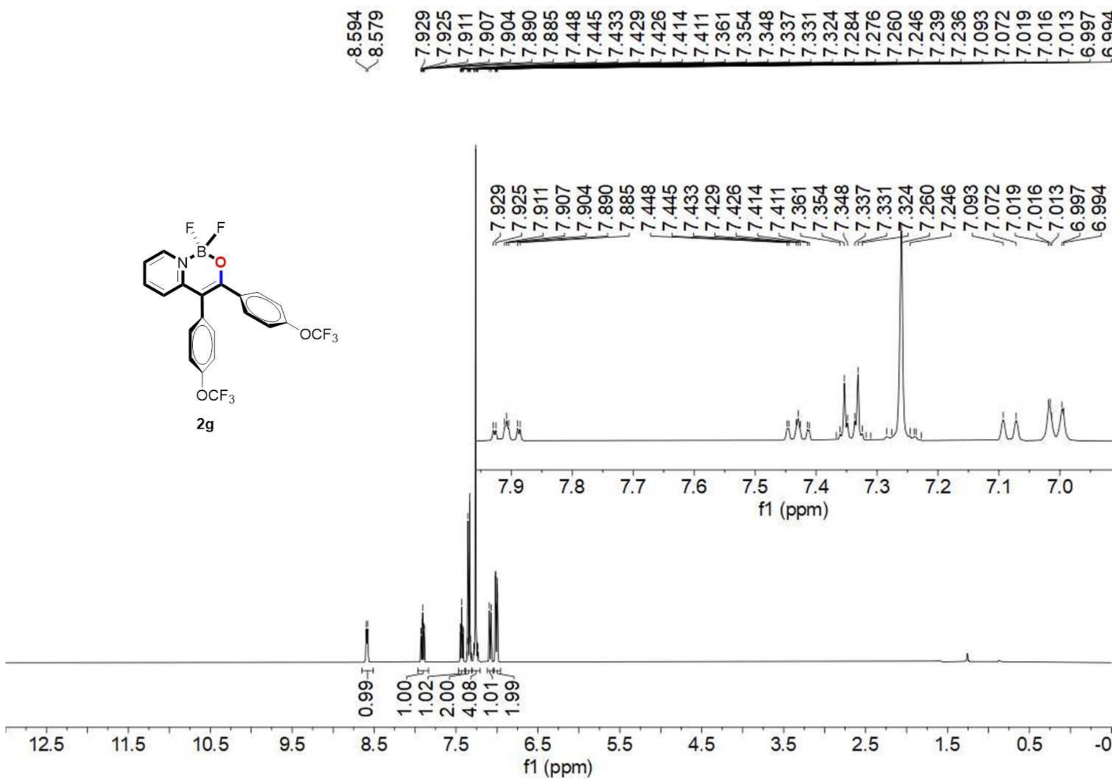
8.594
8.579
7.929 7.925 7.911 7.907 7.904 7.890 7.885 7.448 7.445 7.433 7.429 7.426 7.414 7.411 7.361 7.354 7.348 7.337 7.331 7.324 7.284 7.276 7.260 7.246 7.239 7.236 7.093 7.072 7.019 7.016 7.013 6.997
7.929 7.925 7.911 7.907 7.904 7.890 7.885 7.448 7.445 7.433 7.429 7.426 7.414 7.411 7.361 7.354 7.348 7.337 7.331 7.324 7.260 7.246 7.093 7.072 7.019 7.016 7.013 6.997 6.994
7.9 7.8 7.7 7.6 7.5 7.4 7.3 7.2 7.1 7.0
f1 (ppm)
0.99 1.00 1.02 2.00 4.08 1.01 1.99
12.5 11.5 10.5 9.5 8.5 7.5 6.5 5.5 4.5 3.5 2.5 1.5 0.5
f1 (ppm)
F F B N O
OCF3
OCF3
2g

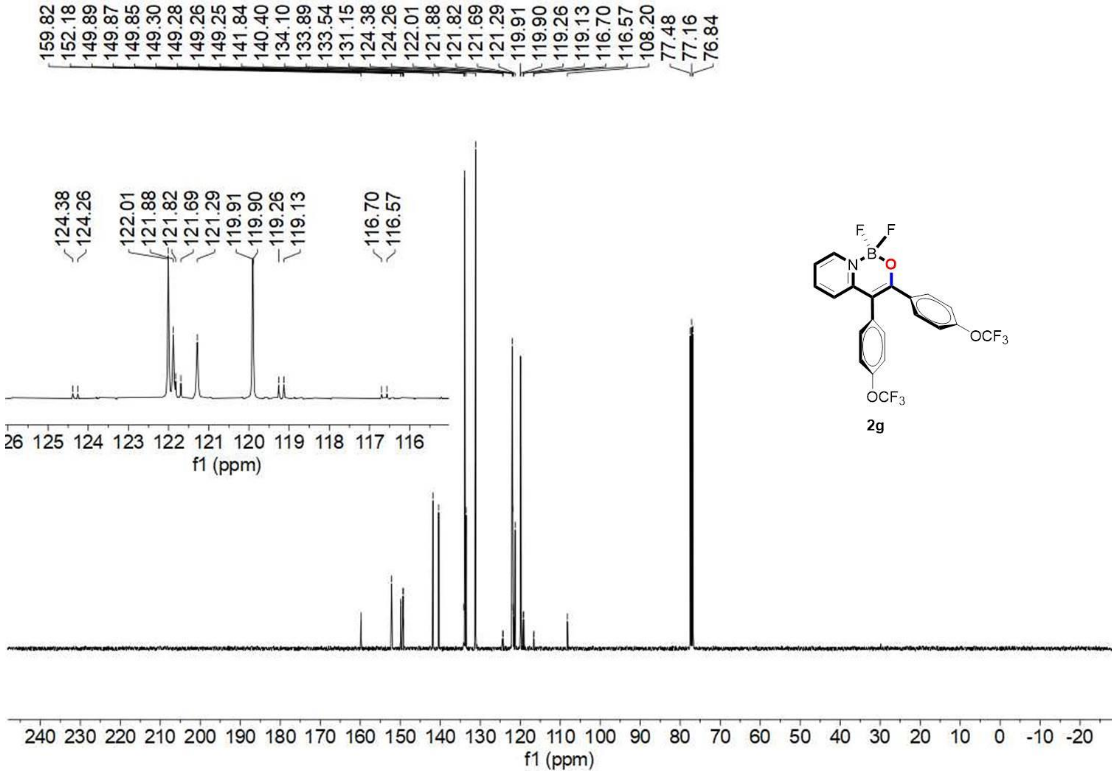
159.82 152.18 149.89 149.87 149.85 149.30 149.28 149.26 149.25 141.84 140.40 134.10 133.89 133.54 131.15 124.38 124.26 122.01 121.88 121.82 121.69 121.29 119.91 119.90 119.26 119.13 116.70 116.57 108.20
77.48 77.16 76.84
124.38 124.26 122.01 121.88 121.82 121.69 121.29 119.91 119.90 119.26 119.13 116.70 116.57
26 125 124 123 122 121 120 119 118 117 116
f1 (ppm)
F F B N O
OCF3
OCF3
2g
240 230 220 210 200 190 180 170 160 150 140 130 120 110 100 90 80 70 60 50 40 30 20 10 0 -10 -20
f1 (ppm)

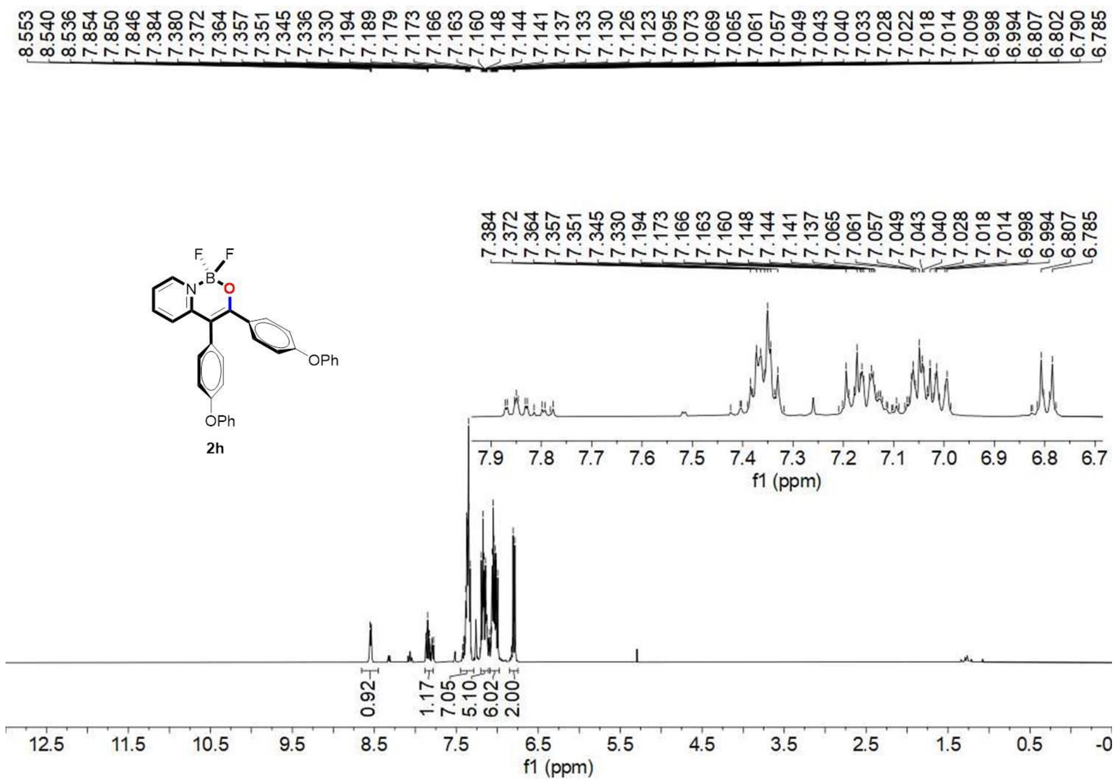
8.553 8.540 8.536 7.854 7.850 7.846 7.384 7.380 7.372 7.364 7.357 7.351 7.345 7.336 7.330 7.194 7.189 7.179 7.173 7.166 7.163 7.160 7.148 7.144 7.141 7.137 7.133 7.130 7.126 7.123 7.095 7.073 7.069 7.065 7.061 7.057 7.049 7.043 7.040 7.033 7.028 7.022 7.018 7.014 7.009 6.998 6.994 6.807 6.802 6.790 6.785
7.384 7.372 7.364 7.357 7.351 7.345 7.330 7.194 7.173 7.166 7.163 7.160 7.148 7.144 7.141 7.137 7.065 7.061 7.057 7.049 7.043 7.040 7.028 7.018 7.014 6.998 6.994 6.807 6.785
0.92 1.17 7.05 5.10 6.02 2.00
f1 (ppm)

2h

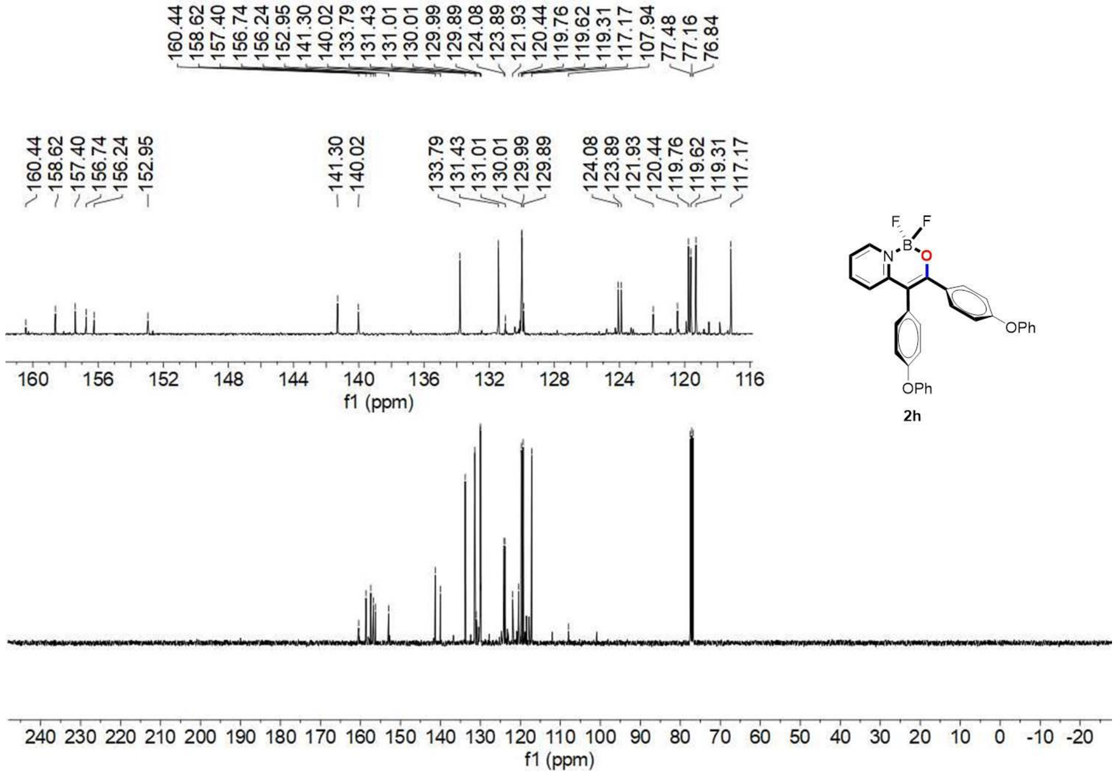
160.44 158.62 157.40 156.74 156.24 152.95 141.30 140.02 133.79 131.43 131.01 130.01 129.99 129.89 124.08 123.89 121.93 120.44 119.76 119.62 119.31 117.17 107.94 77.48 77.16 76.84
f1 (ppm)

2h

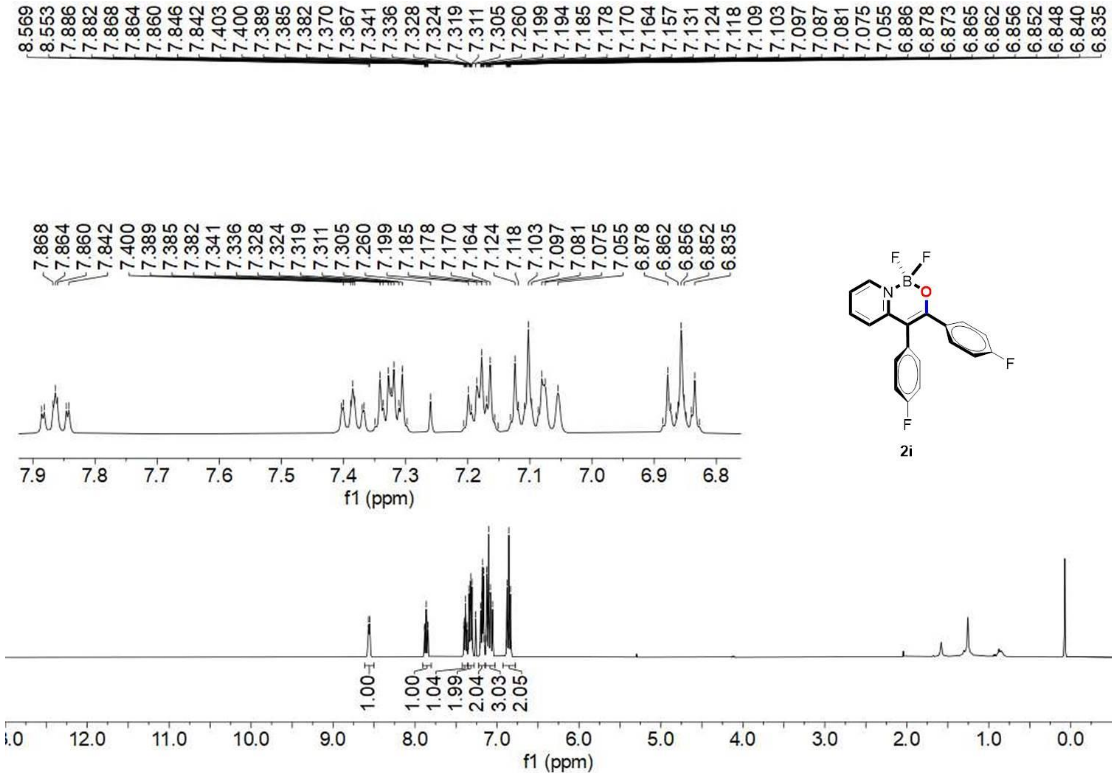
8.569 8.553 7.886 7.882 7.868 7.864 7.860 7.846 7.842 7.403 7.400 7.389 7.385 7.382 7.370 7.367 7.341 7.336 7.328 7.324 7.319 7.311 7.305 7.260 7.199 7.194 7.185 7.178 7.170 7.164 7.157 7.131 7.124 7.118 7.109 7.103 7.097 7.087 7.081 7.075 7.055 6.886 6.878 6.873 6.865 6.862 6.856 6.852 6.848 6.840 6.835
7.868 7.864 7.860 7.842 7.400 7.389 7.385 7.382 7.341 7.336 7.328 7.324 7.319 7.311 7.305 7.260 7.199 7.185 7.178 7.170 7.164 7.124 7.118 7.103 7.097 7.081 7.075 7.055 6.878 6.862 6.856 6.852 6.835
f1 (ppm)
1.00 1.00 1.04 1.99 2.04 3.03 2.05
f1 (ppm)
F F B N O F F
2i

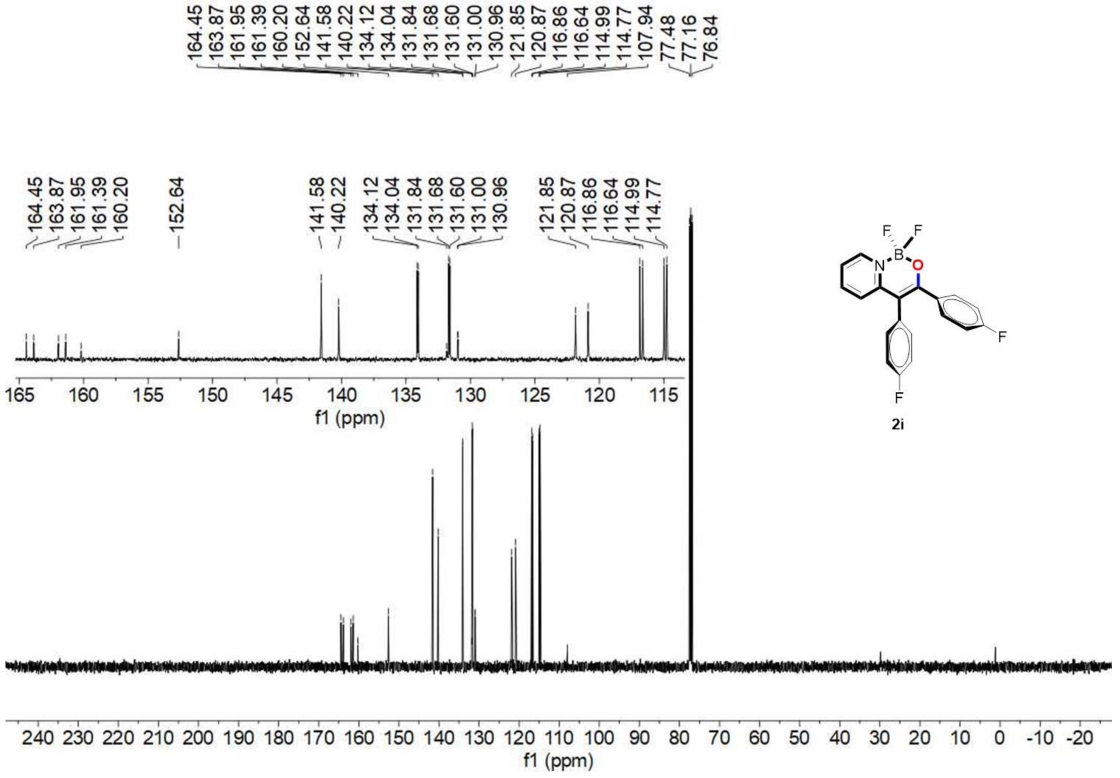
164.45 163.87 161.95 161.39 160.20 152.64 141.58 140.22 134.12 134.04 131.84 131.68 131.60 131.00 130.96 121.85 120.87 116.86 116.64 114.99 114.77 107.94 77.48 77.16 76.84
164.45 163.87 161.95 161.39 160.20 152.64 141.58 140.22 134.12 134.04 131.84 131.68 131.60 131.00 130.96 121.85 120.87 116.86 116.64 114.99 114.77
f1 (ppm)
f1 (ppm)
F F B N O F F
2i

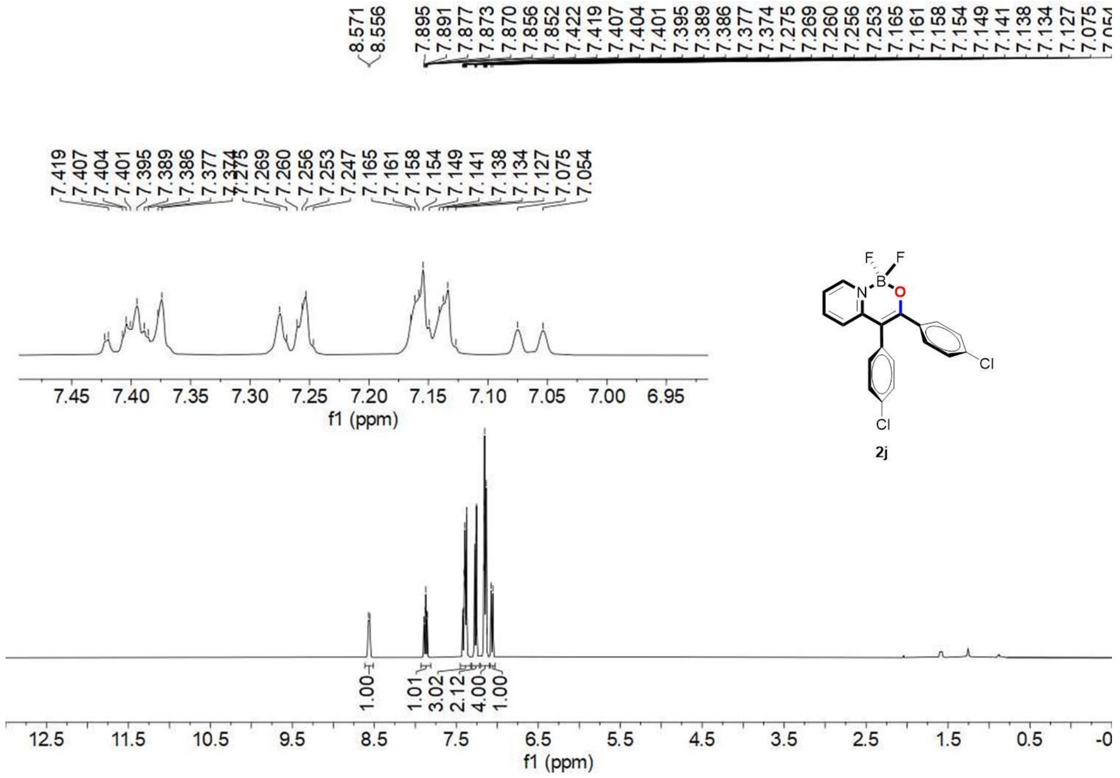

8.571
8.556
7.895
7.891
7.877
7.873
7.870
7.856
7.852
7.422
7.419
7.407
7.404
7.401
7.395
7.389
7.386
7.377
7.374
7.275
7.269
7.260
7.256
7.253
7.165
7.161
7.158
7.154
7.149
7.141
7.138
7.134
7.127
7.075
7.054
F
F
N
B
O
Cl
Cl
2j
1.00
1.01
3.02
2.12
4.00
1.00
f1 (ppm)

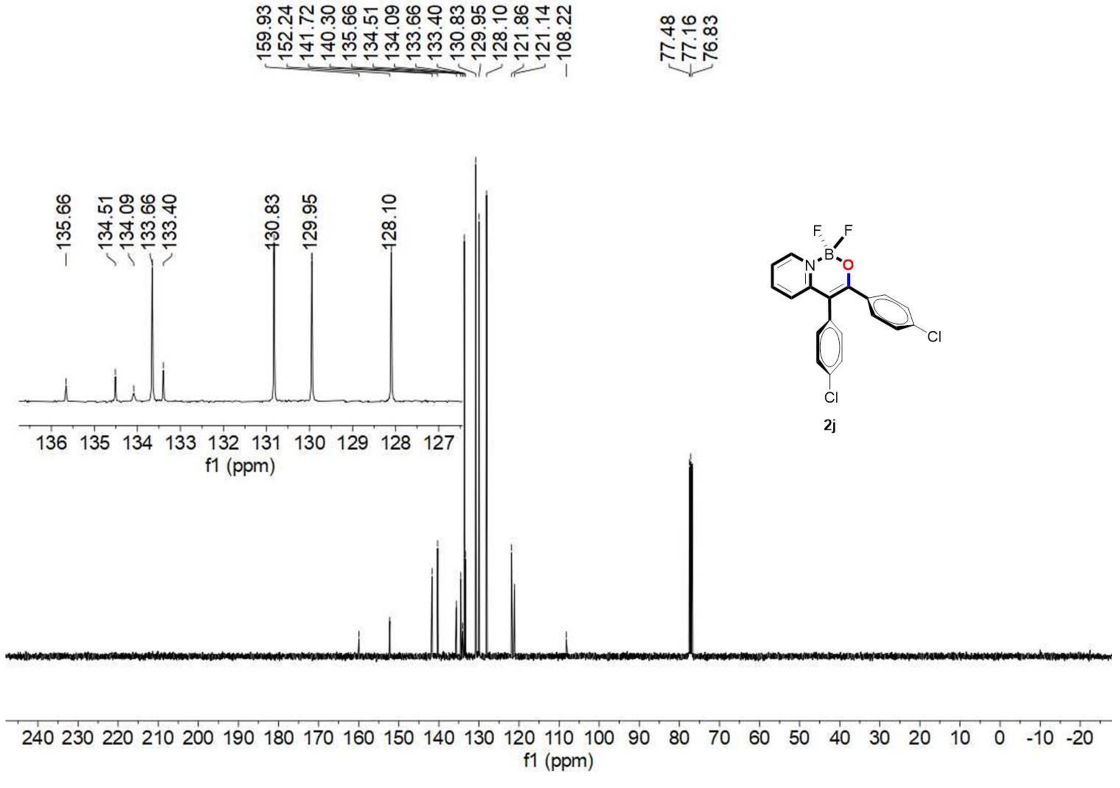

159.93
152.24
141.72
140.30
135.66
134.51
134.09
133.66
133.40
130.83
129.95
128.10
121.86
121.14
108.22
77.48
77.16
76.83
F
F
N
B
O
Cl
Cl
2j
f1 (ppm)

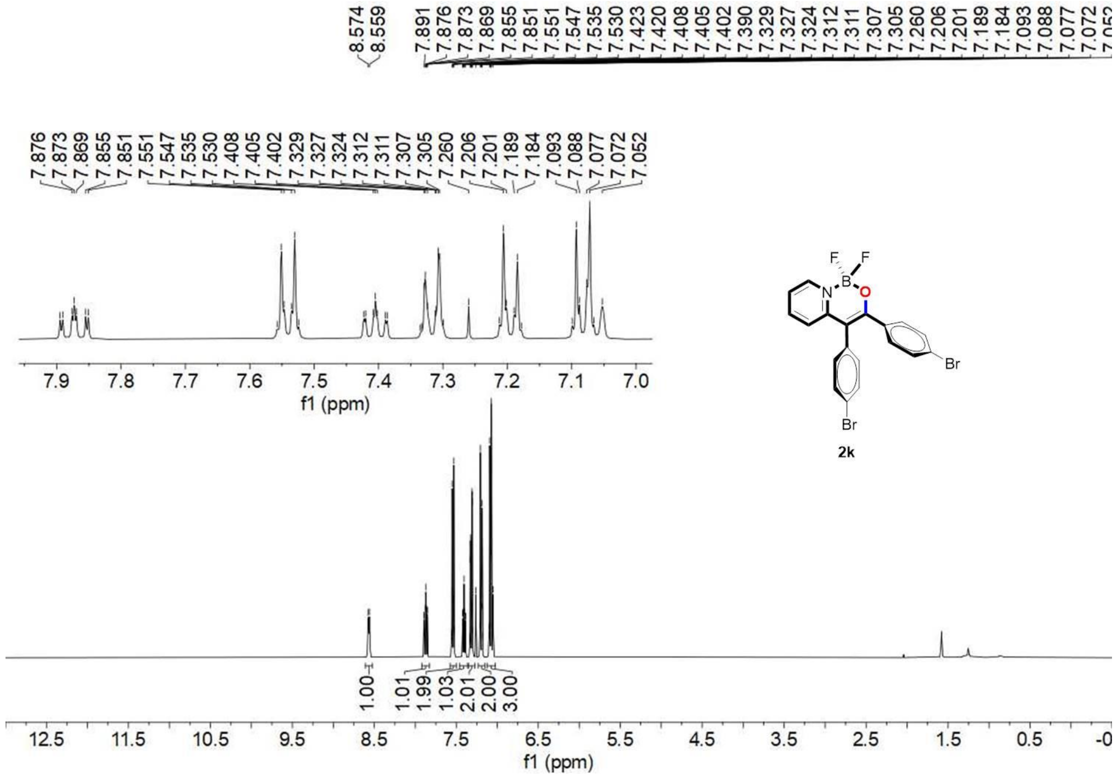
8.574
8.559
7.891
7.876
7.873
7.869
7.855
7.851
7.551
7.547
7.535
7.530
7.423
7.420
7.408
7.405
7.402
7.390
7.329
7.327
7.324
7.312
7.311
7.307
7.305
7.260
7.206
7.201
7.189
7.184
7.093
7.088
7.077
7.072
7.9
7.8
7.7
7.6
7.5
7.4
7.3
7.2
7.1
7.0
f1 (ppm)
F
F
N
B
O
Br
Br
2k
1.00
1.01
1.99
1.03
2.01
2.00
3.00
12.5
11.5
10.5
9.5
8.5
7.5
6.5
5.5
4.5
3.5
2.5
1.5
0.5
f1 (ppm)

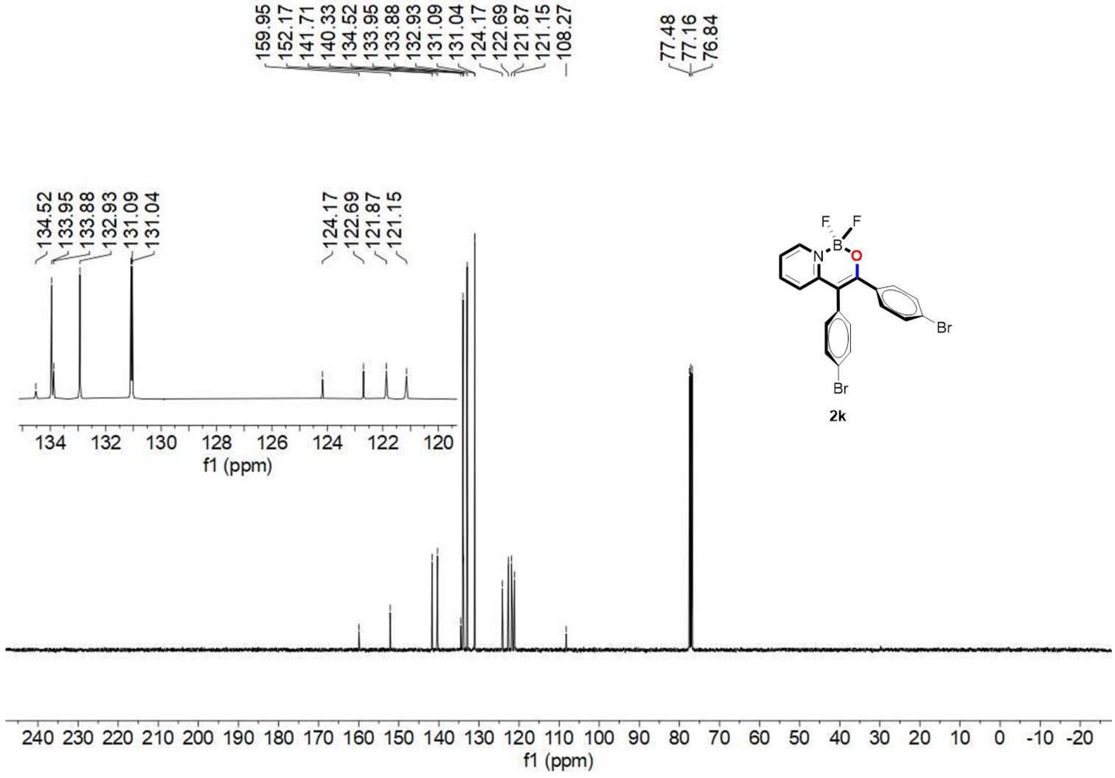
159.95
152.17
141.71
140.33
134.52
133.95
133.88
132.93
131.09
131.04
124.17
122.69
121.87
121.15
108.27
77.48
77.16
76.84
134
132
130
128
126
124
122
120
f1 (ppm)
F
F
N
B
O
Br
Br
2k
240 230 220 210 200 190 180 170 160 150 140 130 120 110 100 90 80 70 60 50 40 30 20 10 0 -10 -20
f1 (ppm)

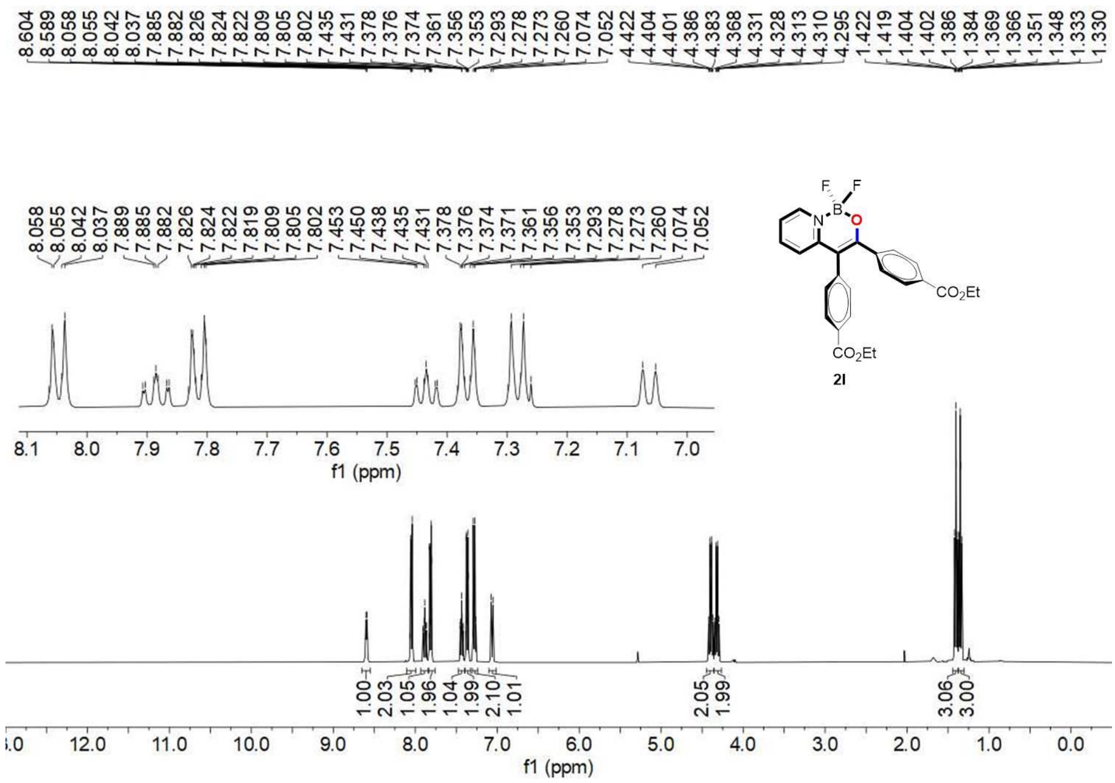

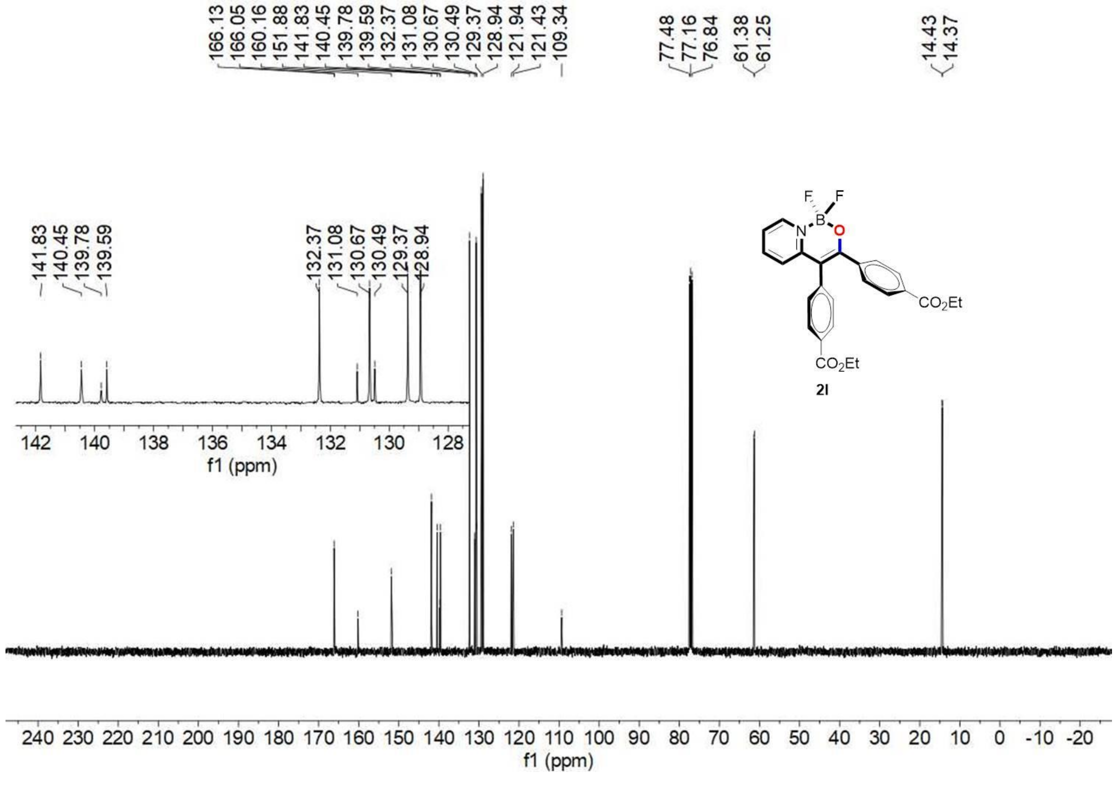

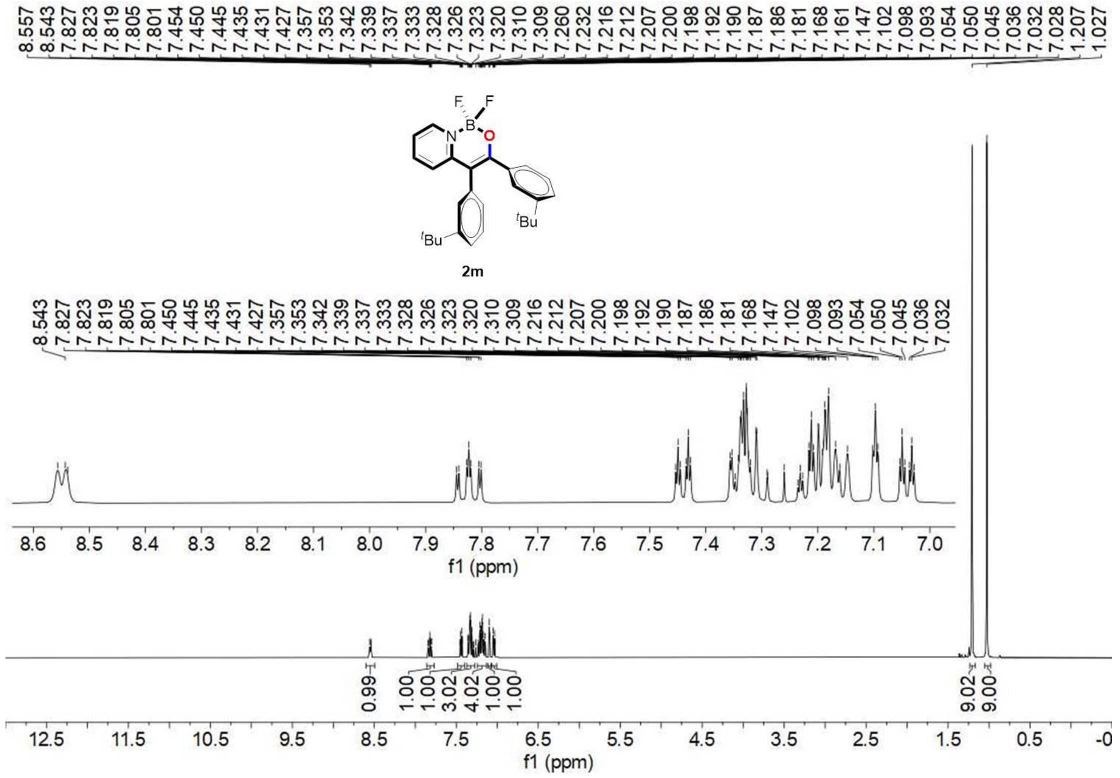
8.557 8.543 7.827 7.823 7.819 7.805 7.801 7.454 7.450 7.445 7.435 7.431 7.427 7.357 7.353 7.342 7.339 7.337 7.333 7.328 7.326 7.323 7.320 7.310 7.309 7.260 7.232 7.216 7.212 7.207 7.200 7.198 7.192 7.190 7.187 7.186 7.181 7.168 7.161 7.147 7.102 7.098 7.093 7.054 7.050 7.045 7.036 7.032 7.028 1.207 1.027
F
F
B
N
O
tBu
tBu
2m
0.99
1.00
1.00
3.02
4.02
1.00
1.00
9.02
9.00
f1 (ppm)

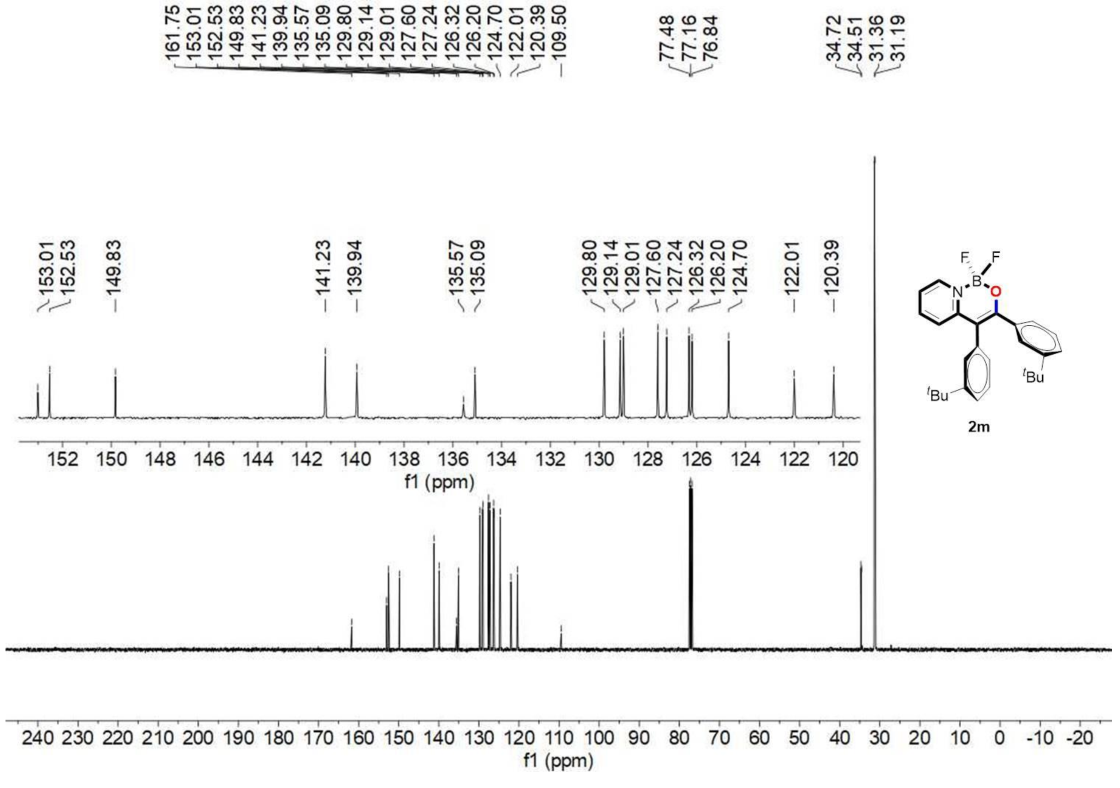
161.75 153.01 152.53 149.83 141.23 139.94 135.57 135.09 129.80 129.14 129.01 127.60 127.24 126.32 126.20 124.70 122.01 120.39 109.50
77.48 77.16 76.84
34.72 34.51 31.36 31.19
F
F
B
N
O
tBu
tBu
2m
f1 (ppm)

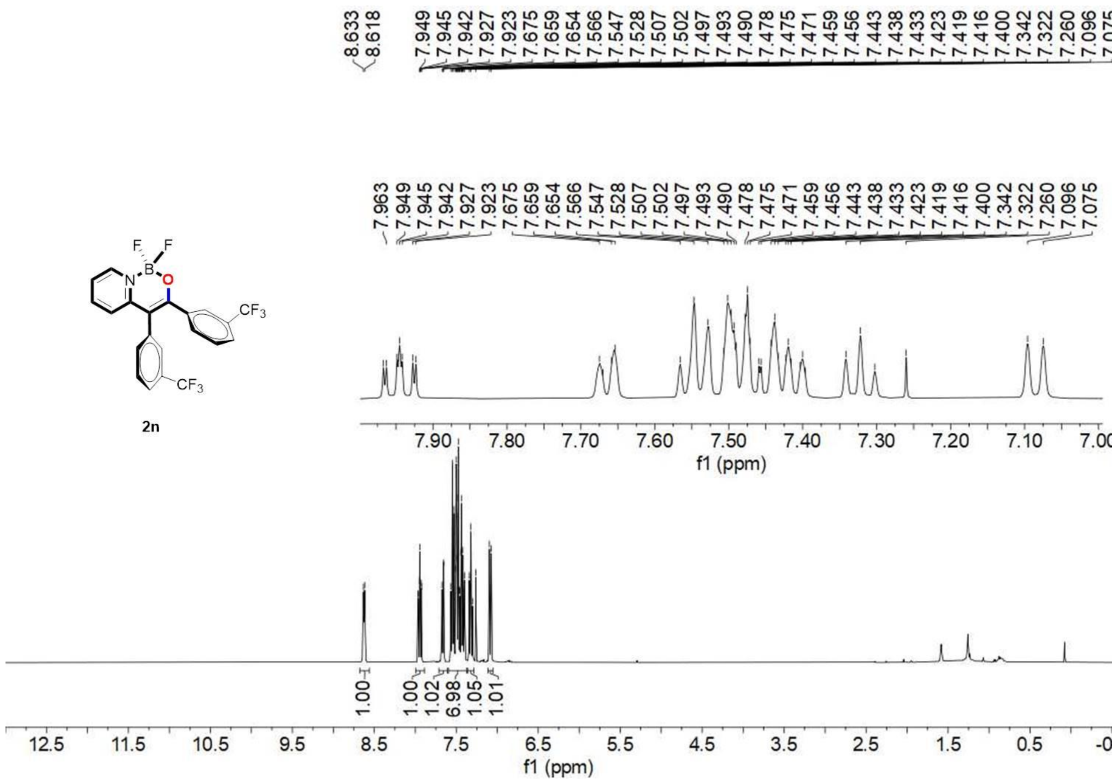

8.633
8.618
7.949
7.945
7.942
7.927
7.923
7.675
7.659
7.654
7.566
7.547
7.528
7.507
7.502
7.497
7.493
7.490
7.478
7.475
7.471
7.459
7.456
7.443
7.438
7.433
7.423
7.419
7.416
7.400
7.342
7.322
7.260
7.096
7.963
7.075
1.00
1.00
1.02
6.98
1.05
1.01
f1 (ppm)

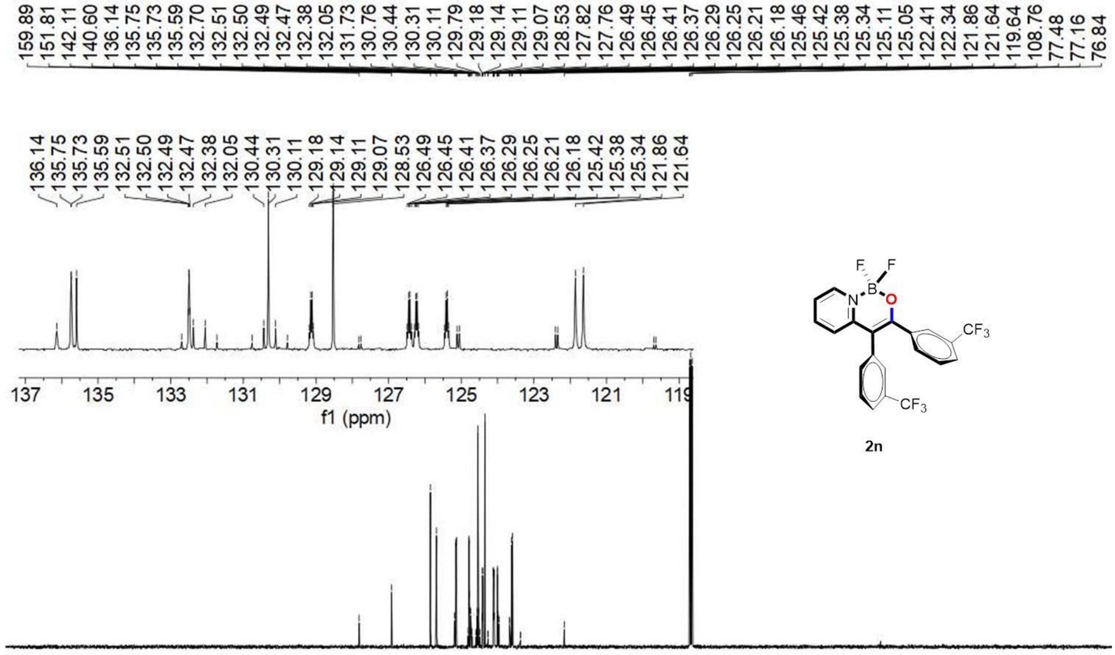

159.89
151.81
142.11
140.60
136.14
135.75
135.73
135.59
132.51
132.50
132.49
132.47
132.38
132.05
131.73
130.76
130.44
130.31
130.11
129.79
129.18
129.14
129.11
129.07
128.53
127.76
126.49
126.45
126.41
126.37
126.29
126.25
126.21
126.18
125.46
125.42
125.38
125.34
125.11
125.05
122.41
122.34
121.86
121.64
119.64
108.76
77.48
77.16
76.84
f1 (ppm)

2n

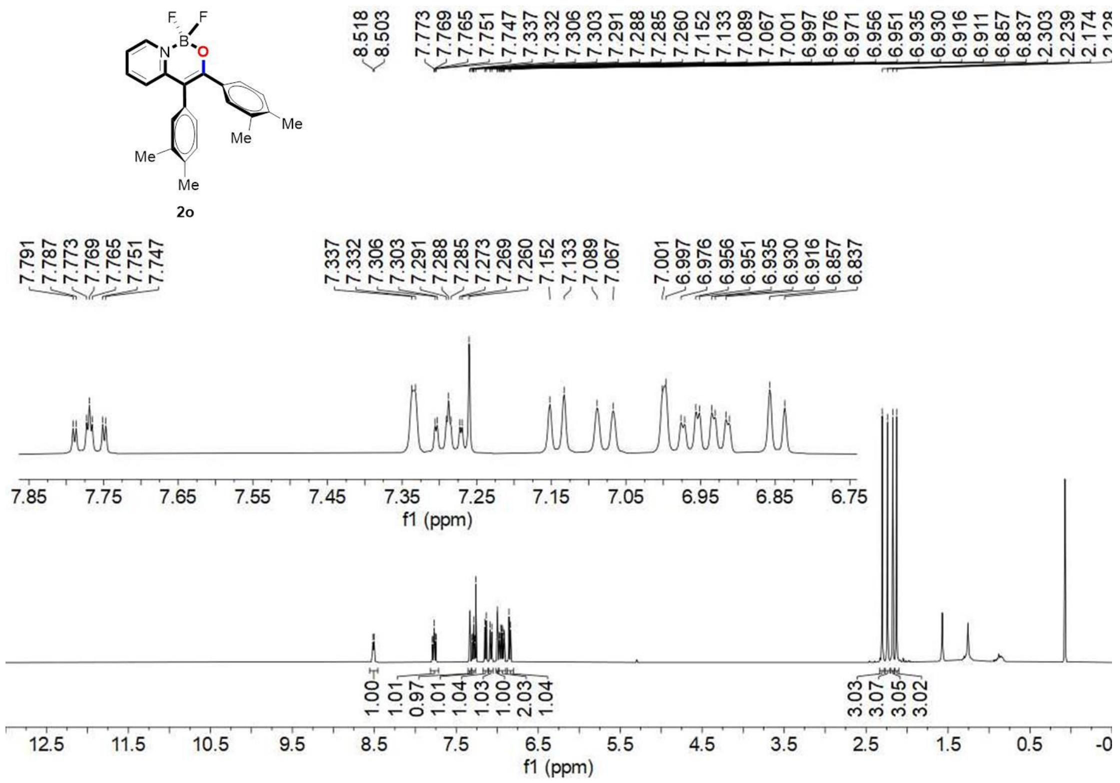

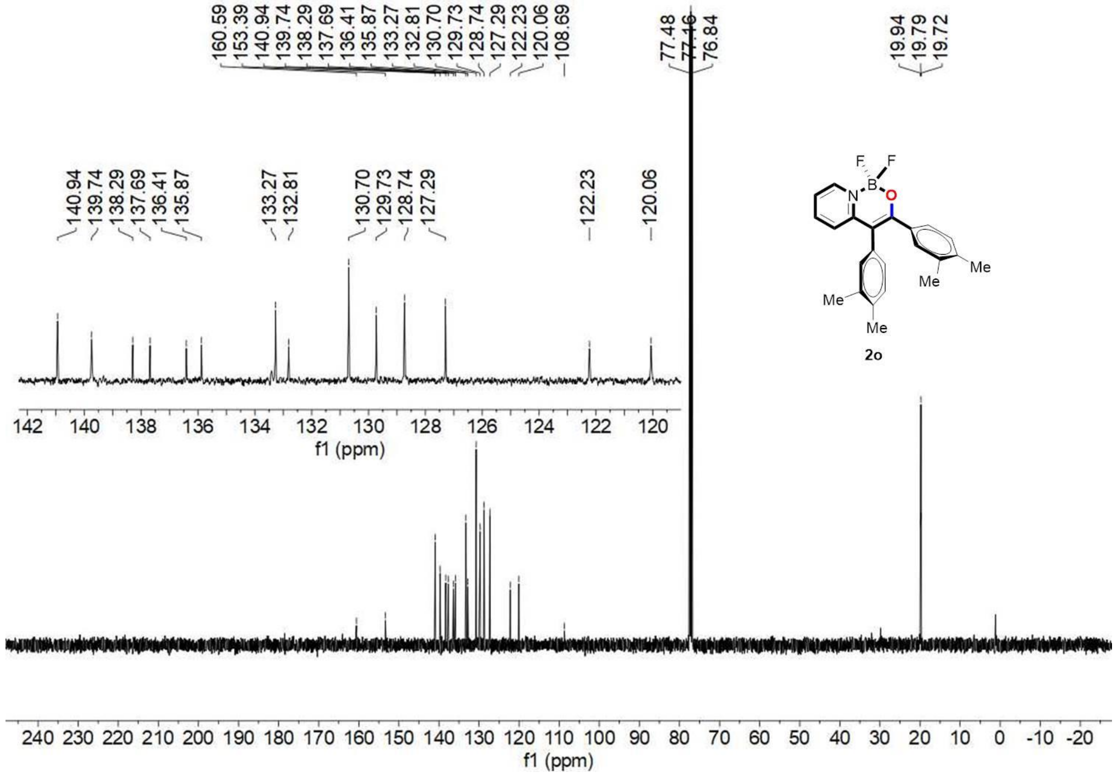

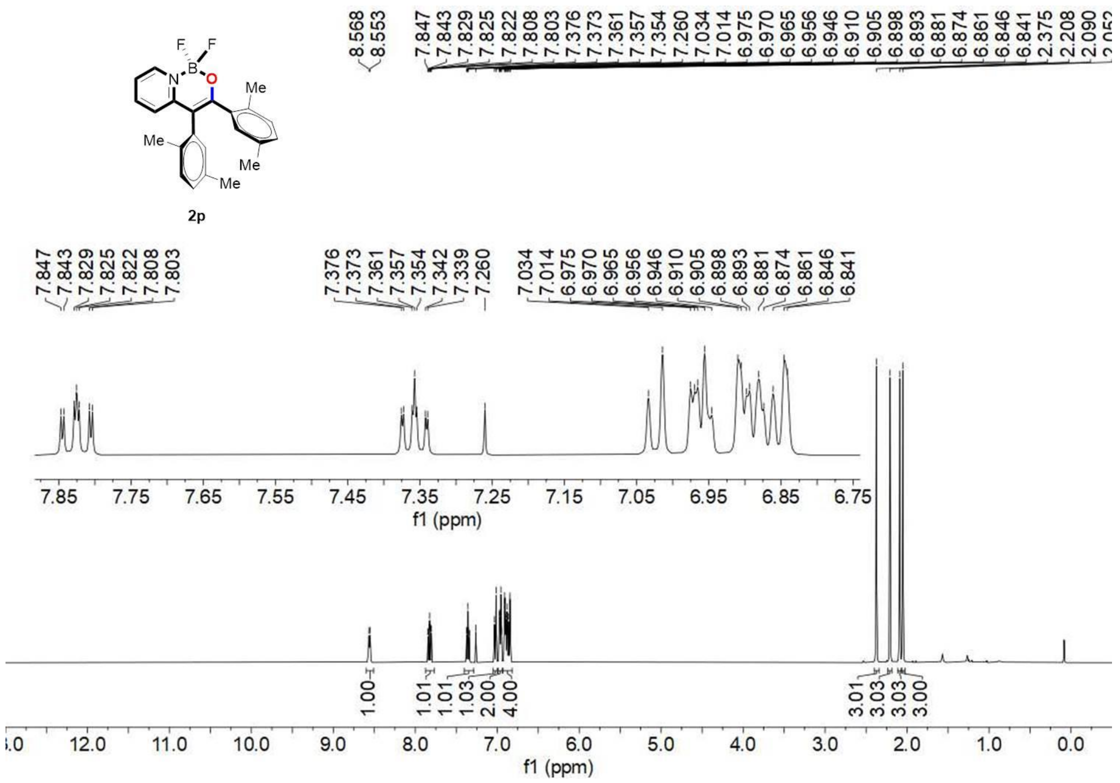

2p
8.568 8.553 7.847 7.843 7.829 7.825 7.822 7.808 7.803 7.376 7.373 7.361 7.357 7.354 7.260 7.034 7.014 6.975 6.970 6.965 6.956 6.946 6.910 6.905 6.898 6.893 6.881 6.874 6.861 6.846 6.841 2.375 2.208 2.090
7.376 7.373 7.361 7.357 7.354 7.342 7.339 7.260
1.00 1.01 1.01 1.03 2.00 4.00 3.01 3.03 3.03 3.00
f1 (ppm)
f1 (ppm)

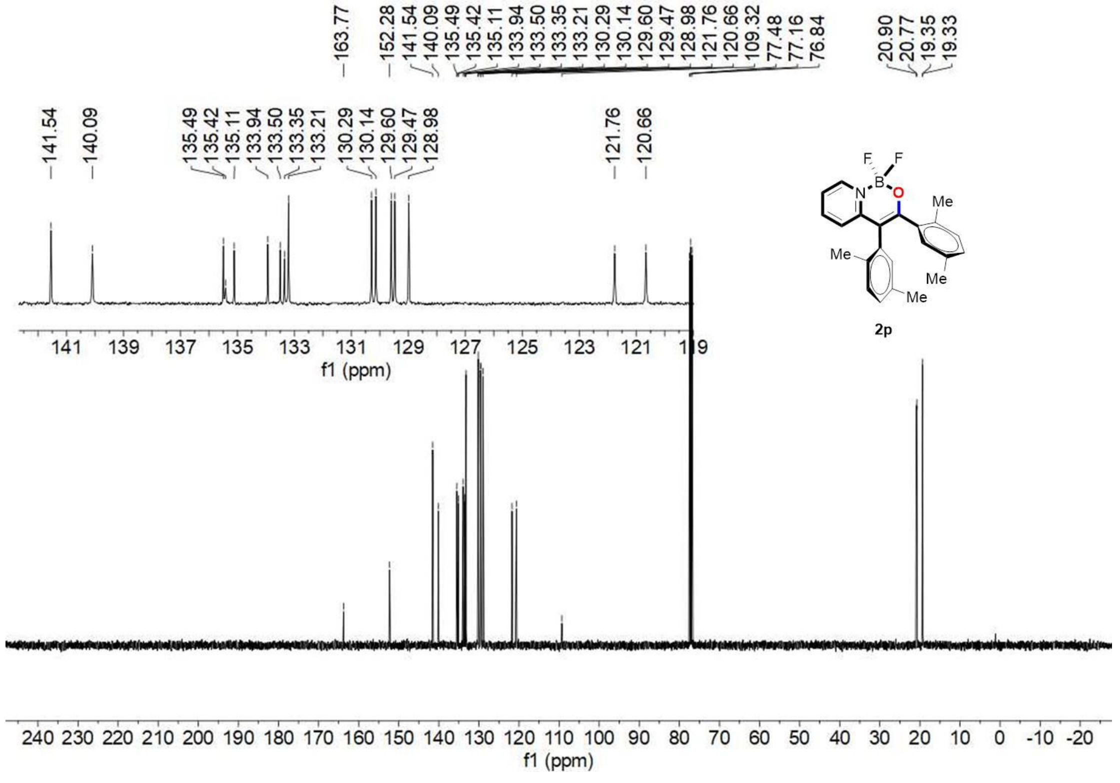

163.77 152.28 141.54 140.09 135.49 135.42 135.11 133.94 133.50 133.35 133.21 130.29 130.14 129.60 129.47 128.98 121.76 120.66 109.32 77.48 77.16 76.84
20.90 20.77 19.35 19.33
2p
f1 (ppm)
f1 (ppm)

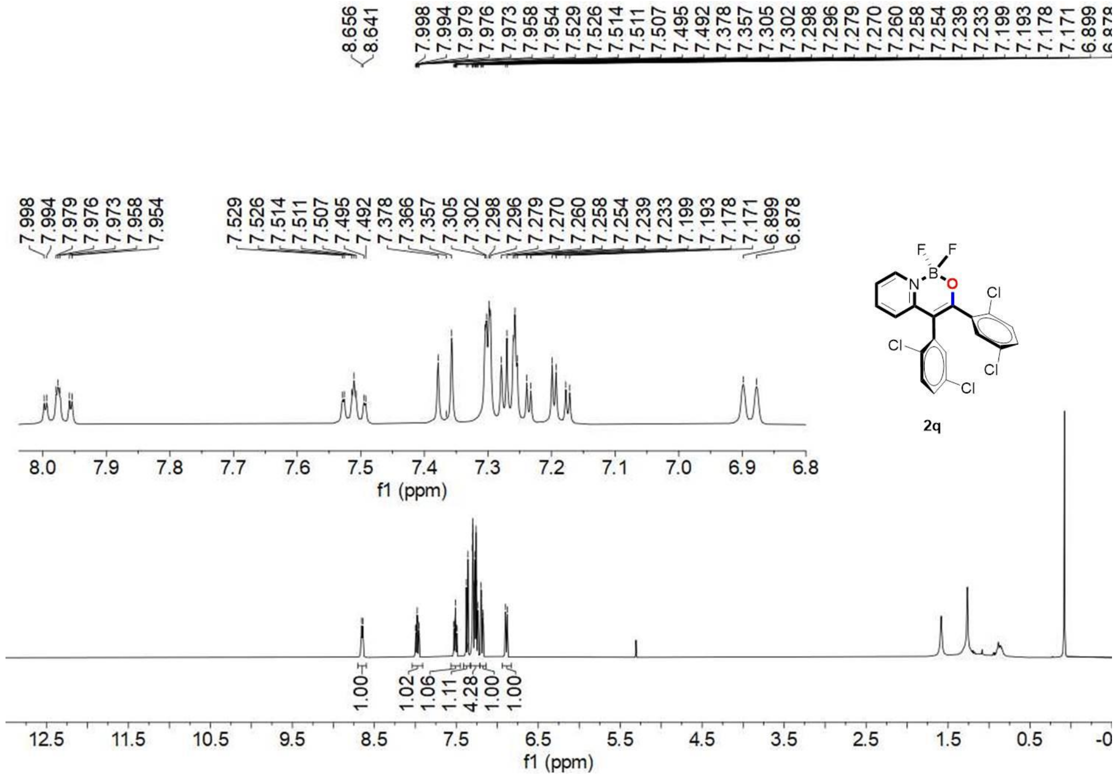
8.656
8.641
7.998
7.994
7.979
7.976
7.973
7.958
7.954
7.529
7.526
7.514
7.511
7.507
7.495
7.492
7.378
7.366
7.357
7.305
7.302
7.298
7.296
7.279
7.270
7.260
7.258
7.254
7.239
7.233
7.199
7.193
7.178
7.171
6.899
6.878
F
F
B
N
O
Cl
Cl
Cl
Cl
2q
1.00
1.02
1.06
1.11
4.28
1.00
1.00
f1 (ppm)
f1 (ppm)

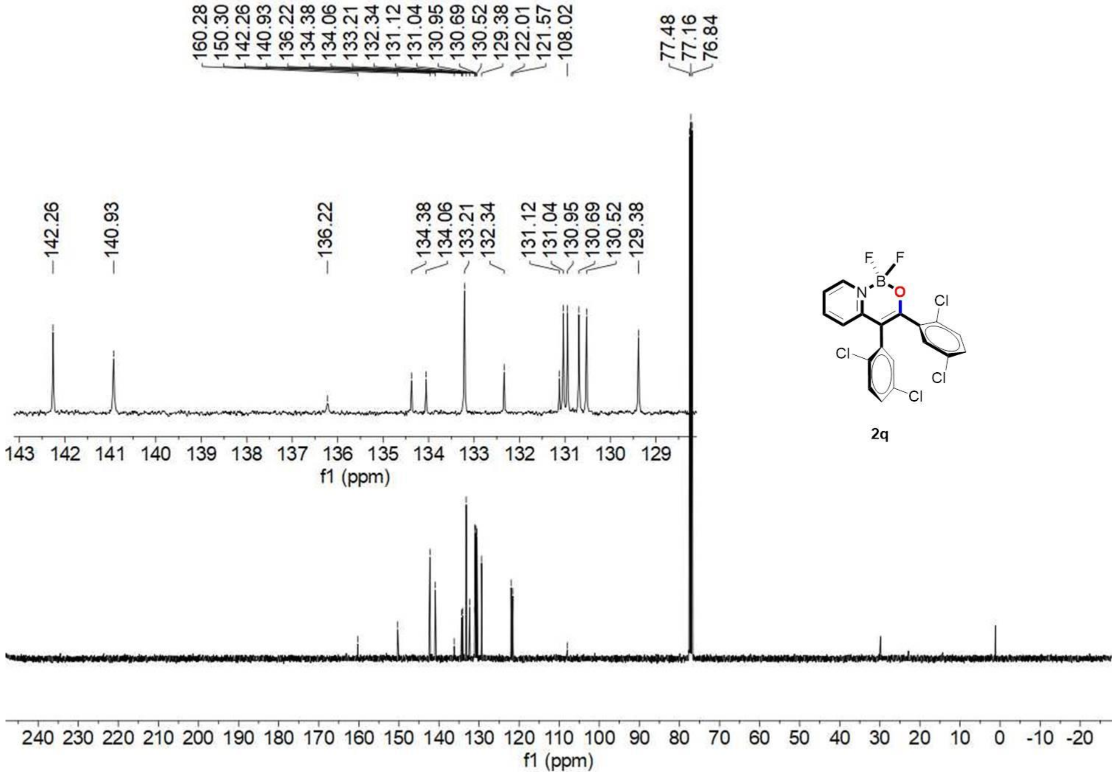
160.28
150.30
142.26
140.93
136.22
134.38
134.06
133.21
132.34
131.12
131.04
130.95
130.69
130.52
129.38
122.01
121.57
108.02
77.48
77.16
76.84
F
F
B
N
O
Cl
Cl
Cl
Cl
2q
f1 (ppm)
f1 (ppm)

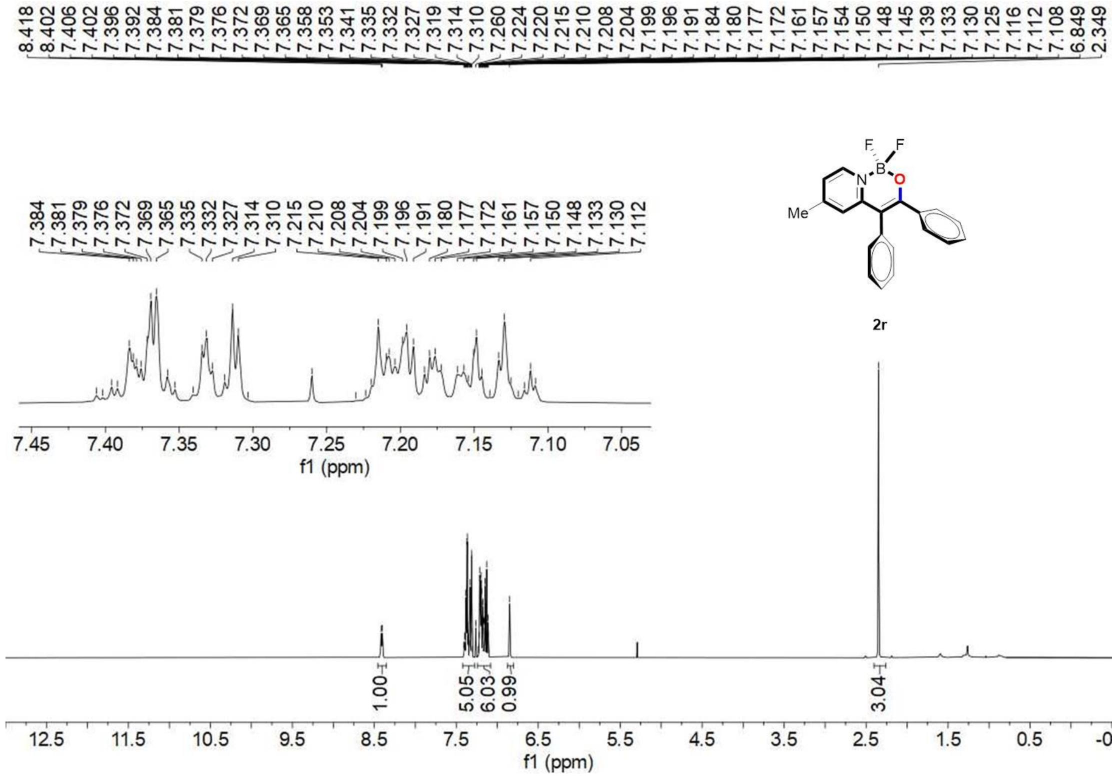
8.418 8.402 7.406 7.402 7.396 7.392 7.384 7.381 7.379 7.376 7.372 7.369 7.365 7.358 7.353 7.341 7.335 7.332 7.327 7.319 7.314 7.310 7.260 7.224 7.220 7.215 7.210 7.208 7.204 7.199 7.196 7.191 7.184 7.180 7.177 7.172 7.161 7.157 7.154 7.150 7.148 7.145 7.139 7.133 7.130 7.125 7.116 7.112 7.108 6.849 2.349
7.384 7.381 7.379 7.376 7.372 7.369 7.365 7.335 7.332 7.327 7.314 7.310 7.215 7.210 7.208 7.204 7.199 7.196 7.191 7.180 7.177 7.172 7.161 7.157 7.150 7.148 7.133 7.130 7.112
7.45 7.40 7.35 7.30 7.25 7.20 7.15 7.10 7.05
f1 (ppm)
F
F
B
N
O
Me
1.00 5.05 6.03 0.99 3.04
12.5 11.5 10.5 9.5 8.5 7.5 6.5 5.5 4.5 3.5 2.5 1.5 0.5 -0
f1 (ppm)

2r

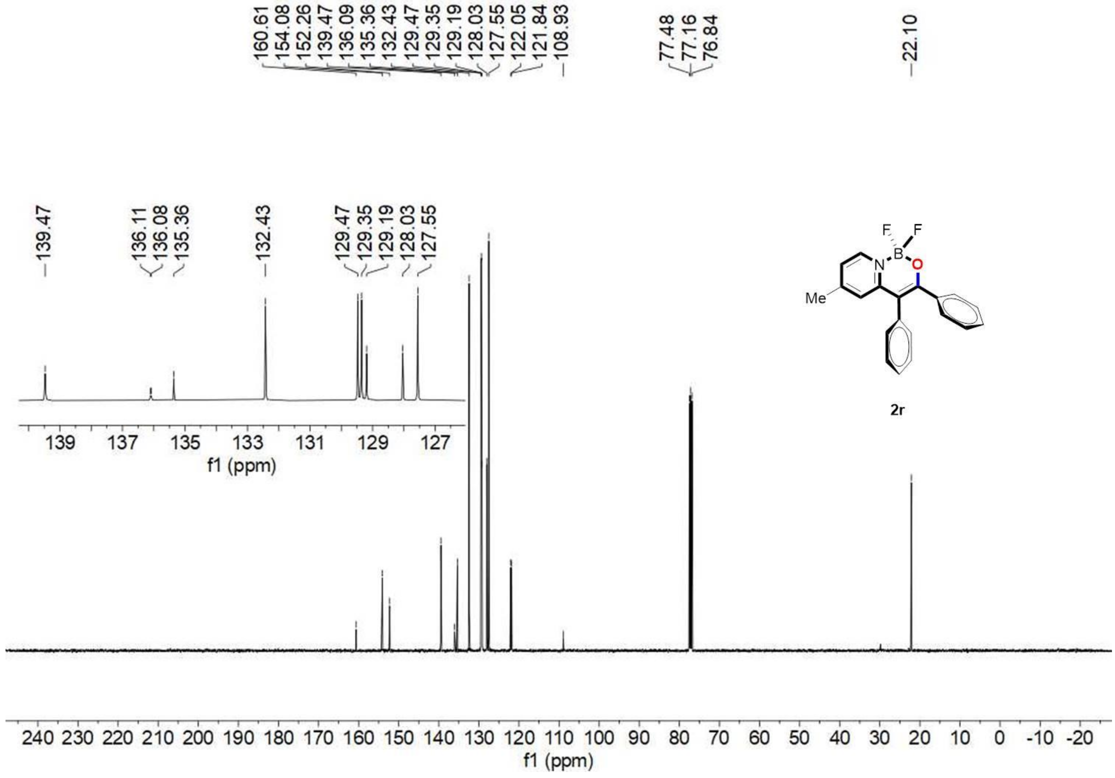
160.61 154.08 152.26 139.47 136.09 135.36 132.43 129.47 129.35 129.19 128.03 127.55 122.05 121.84 108.93
77.48 77.16 76.84
22.10
139.47 136.11 136.08 135.36 132.43 129.47 129.35 129.19 128.03 127.55
139 137 135 133 131 129 127
f1 (ppm)
F
F
B
N
O
Me
240 230 220 210 200 190 180 170 160 150 140 130 120 110 100 90 80 70 60 50 40 30 20 10 0 -10 -20
f1 (ppm)

2r

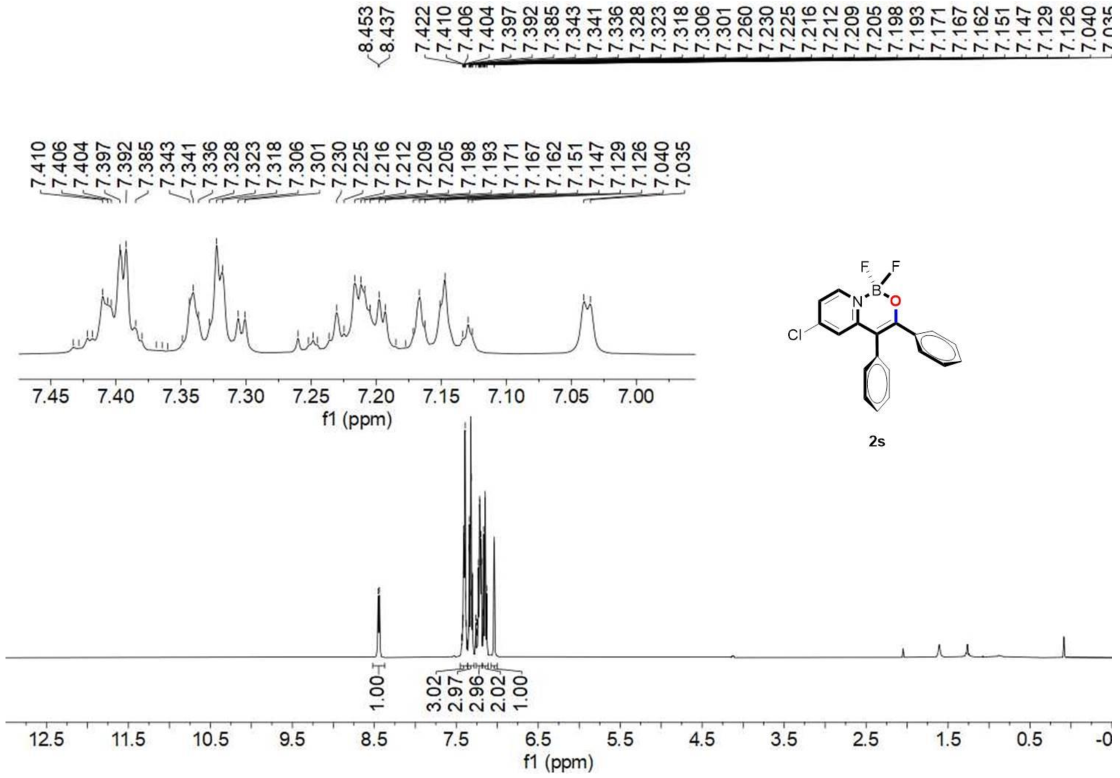

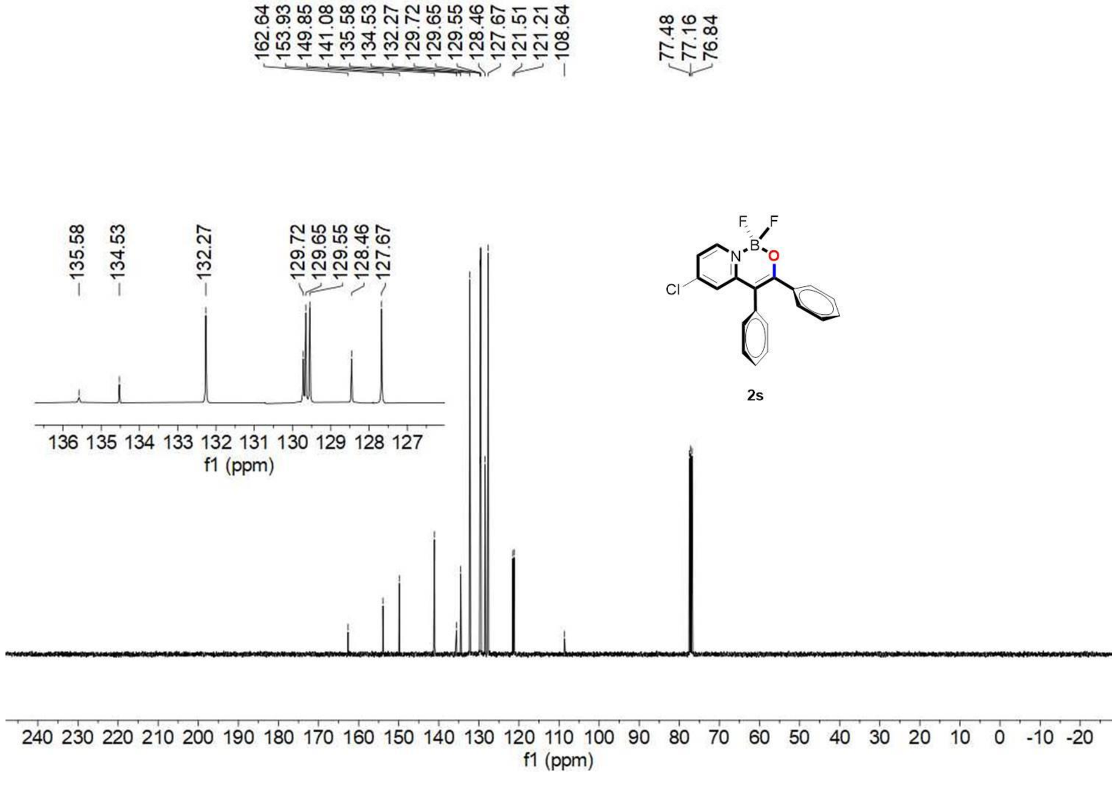

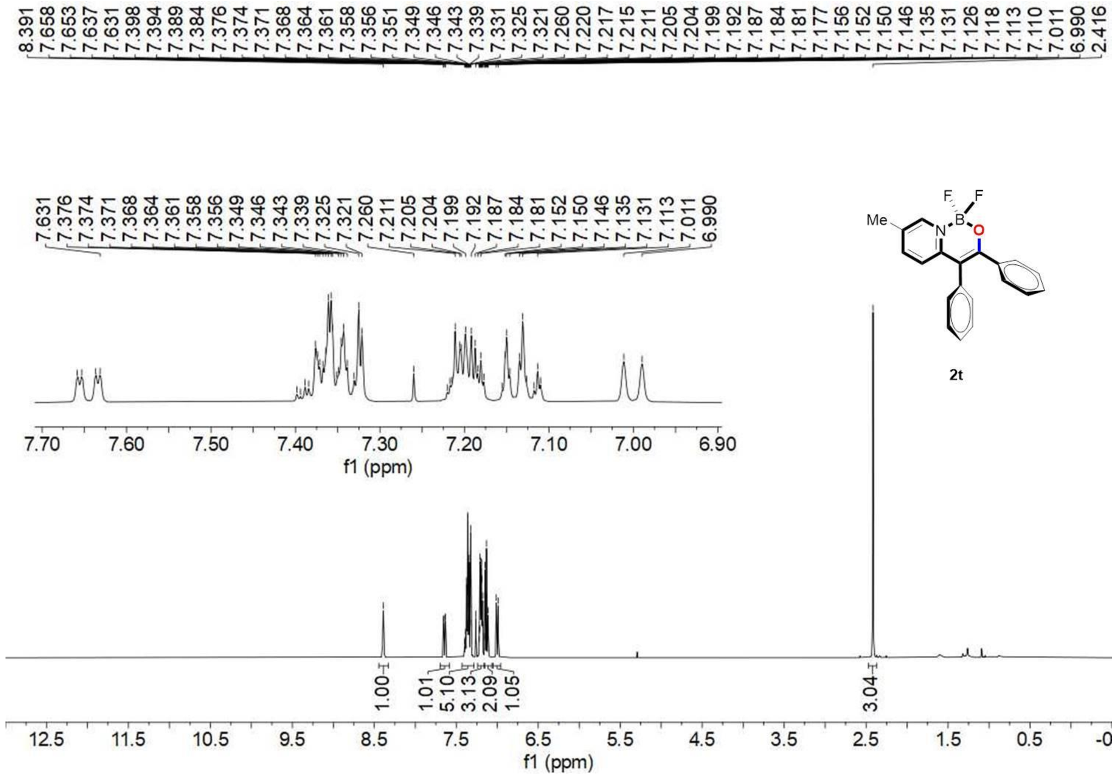
8.391 7.658 7.653 7.637 7.631 7.398 7.394 7.389 7.384 7.376 7.374 7.371 7.368 7.364 7.361 7.358 7.356 7.351 7.349 7.346 7.343 7.339 7.331 7.325 7.321 7.260 7.220 7.217 7.215 7.211 7.205 7.204 7.199 7.192 7.187 7.184 7.181 7.177 7.156 7.152 7.150 7.146 7.135 7.131 7.126 7.118 7.113 7.110 7.011 6.990 2.416
7.631 7.376 7.374 7.371 7.368 7.364 7.361 7.358 7.356 7.349 7.346 7.343 7.339 7.325 7.321 7.260 7.211 7.205 7.204 7.199 7.192 7.187 7.184 7.181 7.152 7.150 7.146 7.135 7.131 7.113 7.011 6.990
7.70 7.60 7.50 7.40 7.30 7.20 7.10 7.00 6.90
f1 (ppm)
Me
F F
B
N
O
2t
1.00 1.01 5.10 3.13 2.09 1.05 3.04
12.5 11.5 10.5 9.5 8.5 7.5 6.5 5.5 4.5 3.5 2.5 1.5 0.5 -0
f1 (ppm)

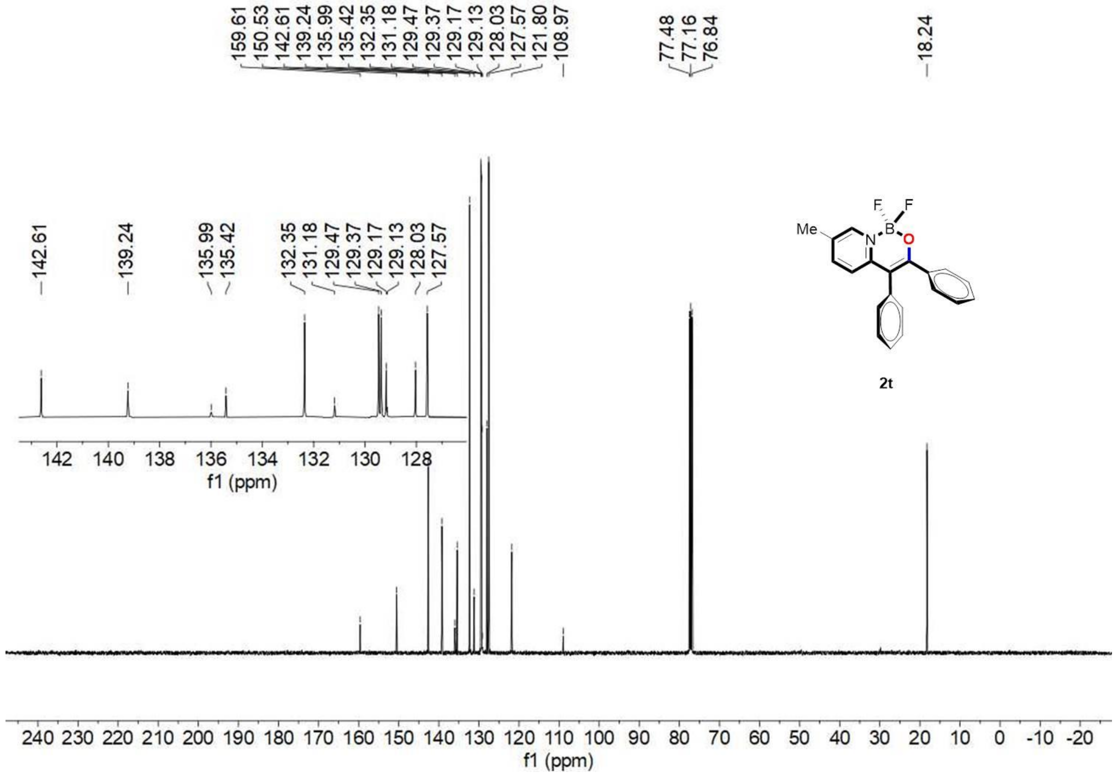
159.61 150.53 142.61 139.24 135.99 135.42 132.35 131.18 129.47 129.37 129.17 129.13 128.03 127.57 121.80 108.97
77.48 77.16 76.84
18.24
142.61 139.24 135.99 135.42 132.35 131.18 129.47 129.37 129.17 129.13 128.03 127.57
142 140 138 136 134 132 130 128
f1 (ppm)
Me
F F
B
N
O
2t
240 230 220 210 200 190 180 170 160 150 140 130 120 110 100 90 80 70 60 50 40 30 20 10 0 -10 -20
f1 (ppm)

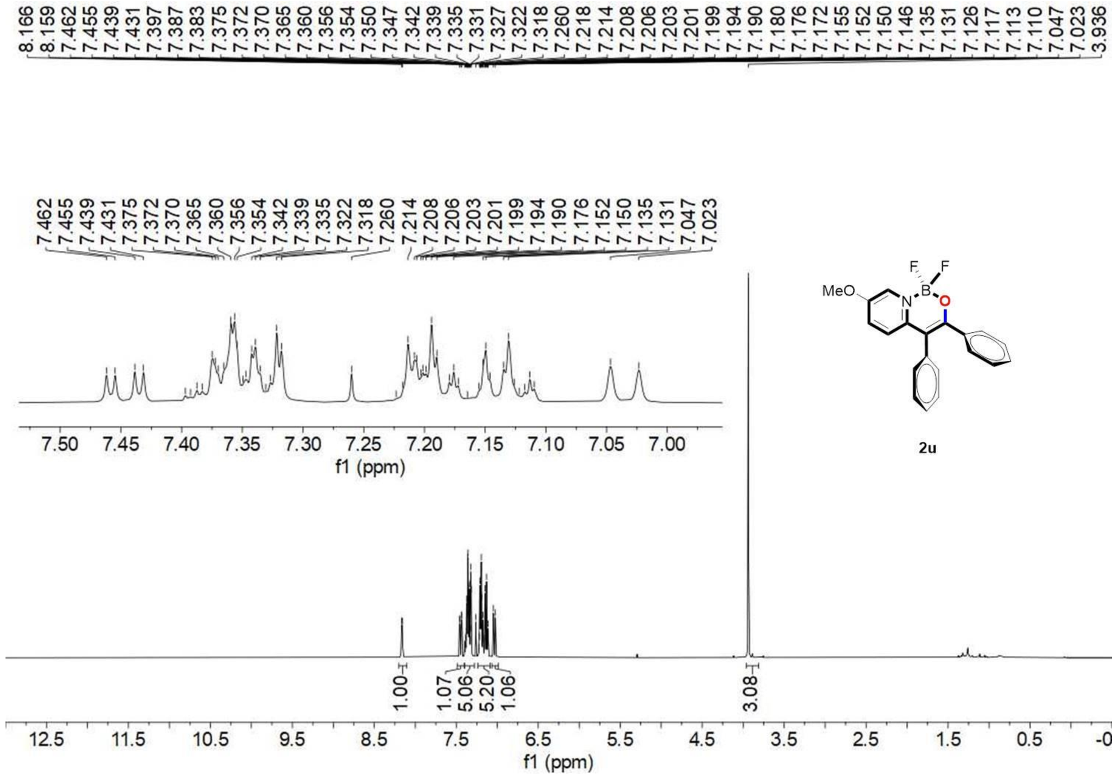
8.166 8.159 7.462 7.455 7.439 7.431 7.397 7.387 7.383 7.375 7.372 7.370 7.365 7.360 7.356 7.354 7.350 7.347 7.342 7.339 7.335 7.331 7.327 7.322 7.318 7.260 7.218 7.214 7.208 7.206 7.203 7.201 7.199 7.194 7.190 7.180 7.176 7.172 7.155 7.152 7.150 7.146 7.135 7.131 7.126 7.117 7.113 7.110 7.047 7.023 3.936
7.462 7.455 7.439 7.431 7.375 7.372 7.370 7.365 7.360 7.356 7.354 7.342 7.339 7.335 7.322 7.318 7.260 7.214 7.208 7.206 7.203 7.201 7.199 7.194 7.190 7.176 7.152 7.150 7.135 7.131 7.047 7.023
f1 (ppm)
MeO
F
F
B
N
O
2u
1.00
1.07
5.06
5.20
1.06
3.08
f1 (ppm)

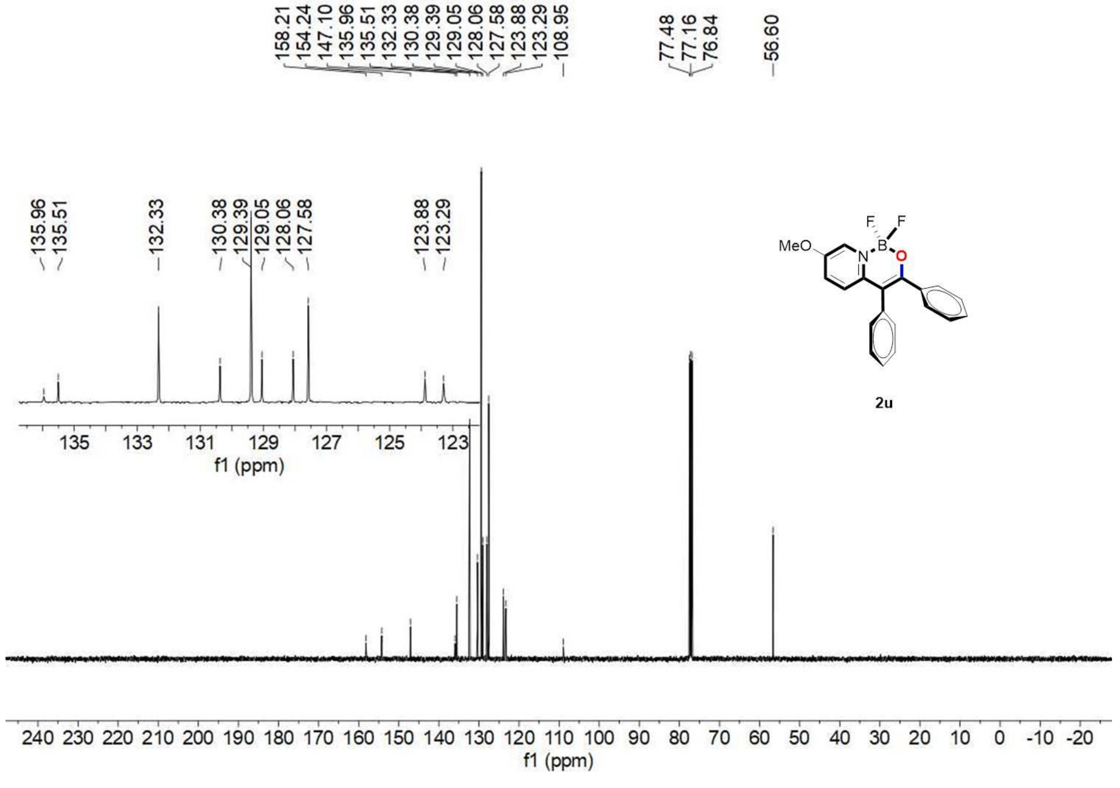
158.21 154.24 147.10 135.96 135.51 132.33 130.38 129.39 129.05 128.06 127.58 123.88 123.29 108.95
77.48 77.16 76.84
56.60
135.96 135.51 132.33 130.38 129.39 129.05 128.06 127.58 123.88 123.29
f1 (ppm)
MeO
F
F
B
N
O
2u
f1 (ppm)

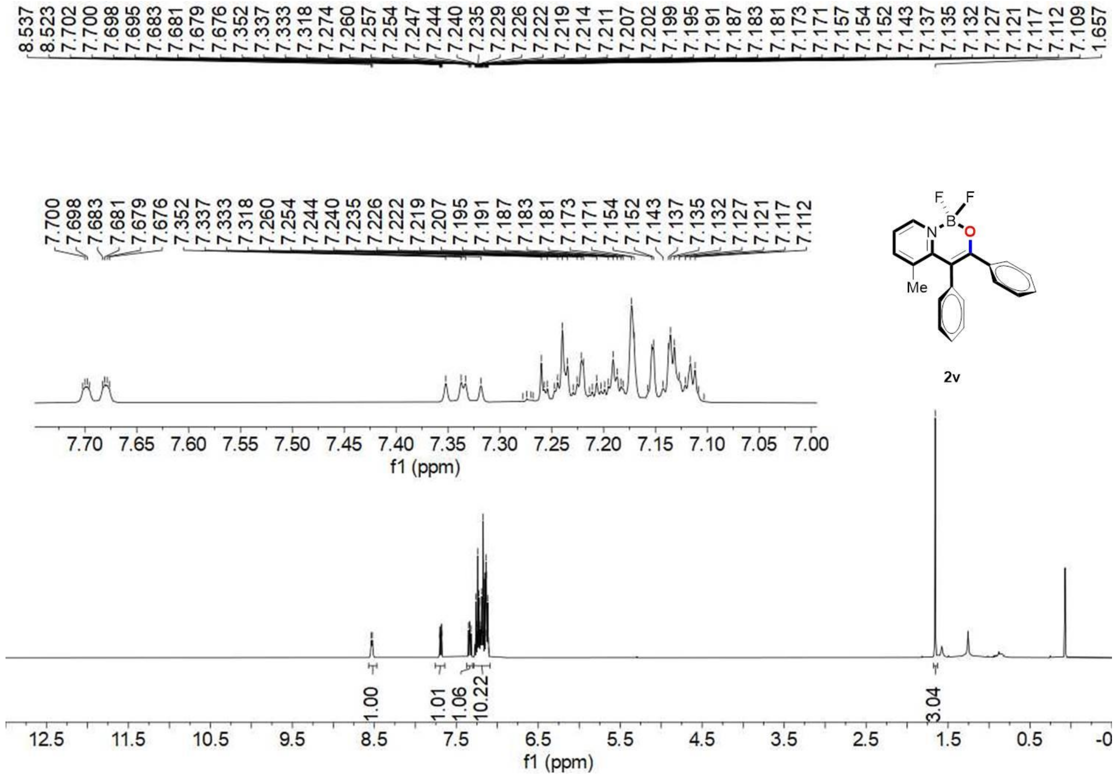
8.537 8.523 7.702 7.700 7.698 7.695 7.683 7.681 7.679 7.676 7.352 7.337 7.333 7.318 7.274 7.260 7.257 7.254 7.247 7.244 7.240 7.235 7.229 7.226 7.222 7.219 7.214 7.211 7.207 7.202 7.199 7.195 7.191 7.187 7.183 7.181 7.173 7.171 7.157 7.154 7.152 7.143 7.137 7.135 7.132 7.127 7.121 7.117 7.112 7.109 1.657
7.700 7.698 7.683 7.681 7.679 7.676 7.352 7.337 7.333 7.318 7.260 7.254 7.244 7.240 7.235 7.226 7.222 7.219 7.207 7.195 7.191 7.187 7.183 7.181 7.173 7.171 7.154 7.152 7.143 7.137 7.135 7.132 7.127 7.121 7.117 7.112
F F B N O Me
2v
7.70 7.65 7.60 7.55 7.50 7.45 7.40 7.35 7.30 7.25 7.20 7.15 7.10 7.05 7.00
f1 (ppm)
1.00 1.01 1.06 10.22 3.04
12.5 11.5 10.5 9.5 8.5 7.5 6.5 5.5 4.5 3.5 2.5 1.5 0.5 -0
f1 (ppm)

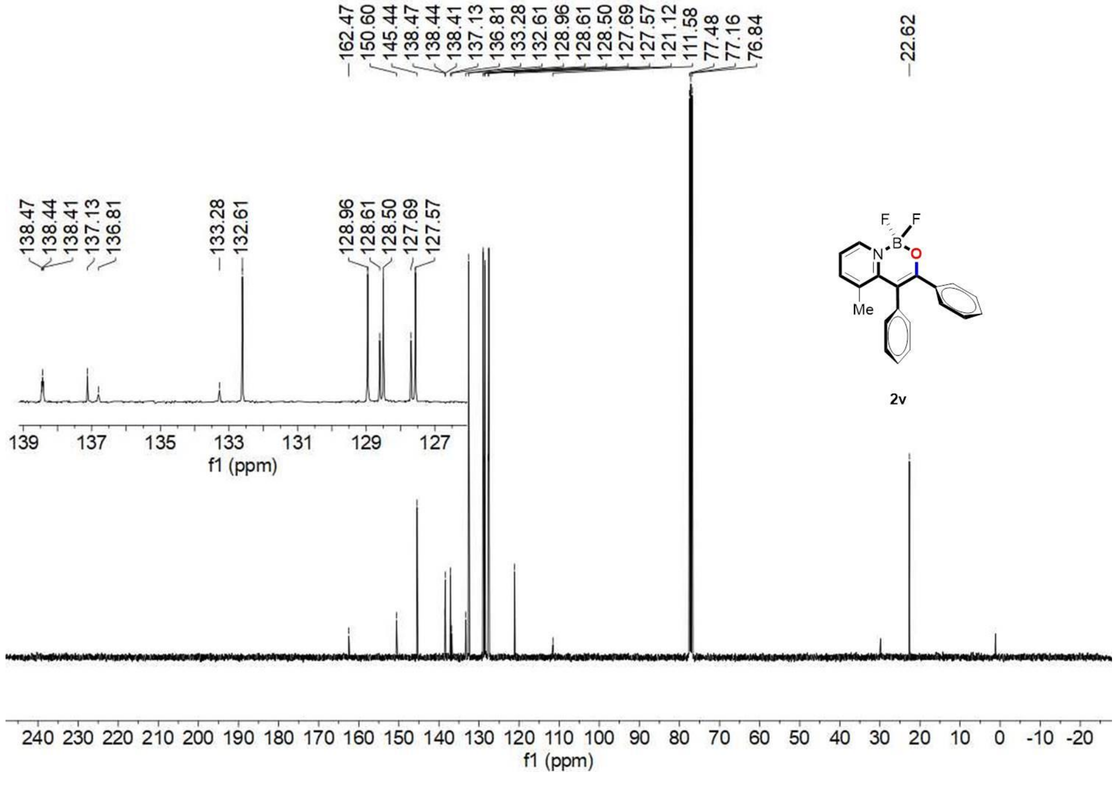
162.47 150.60 145.44 138.47 138.44 138.41 137.13 136.81 133.28 132.61 128.96 128.61 128.50 127.69 127.57 121.12 111.58 77.48 77.16 76.84
22.62
138.47 138.44 138.41 137.13 136.81
133.28 132.61
128.96 128.61 128.50 127.69 127.57
139 137 135 133 131 129 127
f1 (ppm)
F F B N O Me
2v
240 230 220 210 200 190 180 170 160 150 140 130 120 110 100 90 80 70 60 50 40 30 20 10 0 -10 -20
f1 (ppm)

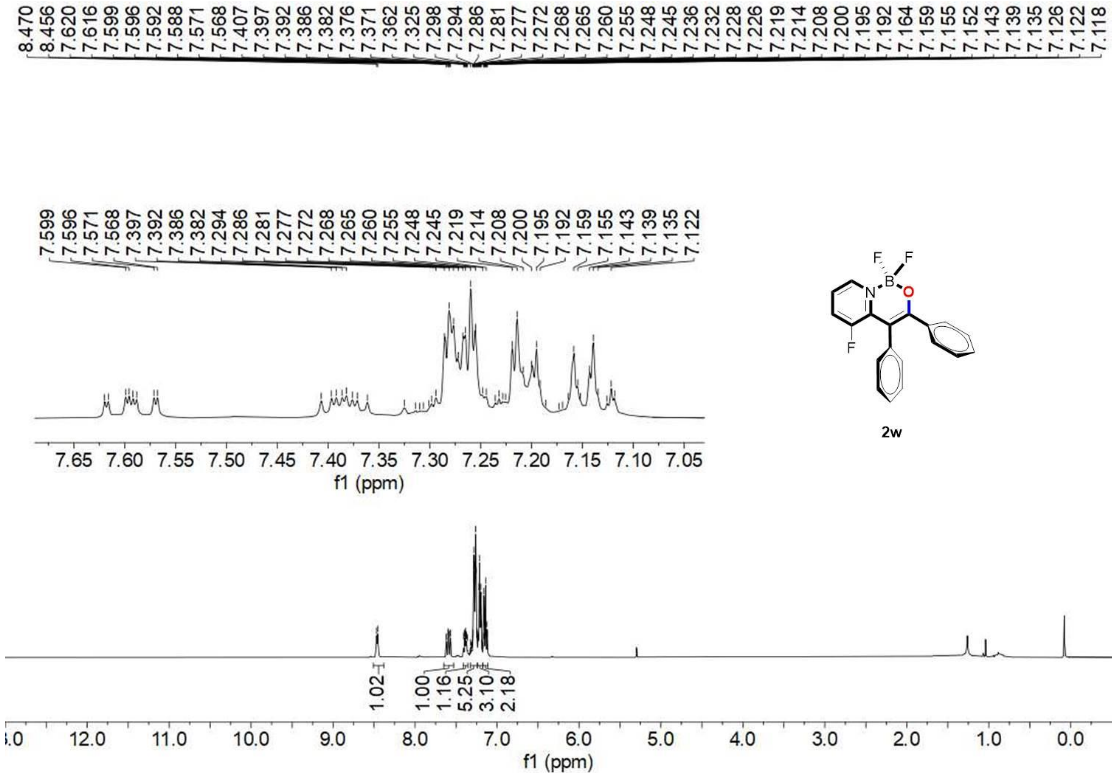
8.470 8.456 7.620 7.616 7.599 7.596 7.592 7.588 7.571 7.568 7.407 7.397 7.392 7.386 7.382 7.376 7.371 7.362 7.325 7.298 7.294 7.286 7.281 7.277 7.272 7.268 7.265 7.260 7.255 7.248 7.245 7.236 7.232 7.228 7.226 7.219 7.214 7.208 7.200 7.195 7.192 7.164 7.159 7.155 7.152 7.143 7.139 7.135 7.126 7.122 7.118
7.599 7.596 7.571 7.568 7.397 7.392 7.386 7.382 7.294 7.286 7.281 7.277 7.272 7.268 7.265 7.260 7.255 7.248 7.245 7.219 7.214 7.208 7.200 7.195 7.192 7.159 7.155 7.143 7.139 7.135 7.122
7.65 7.60 7.55 7.50 7.45 7.40 7.35 7.30 7.25 7.20 7.15 7.10 7.05
f1 (ppm)
1.02 1.00 1.16 5.25 3.10 2.18
12.0 11.0 10.0 9.0 8.0 7.0 6.0 5.0 4.0 3.0 2.0 1.0 0.0
f1 (ppm)
F
F
N
B
O
F

2w

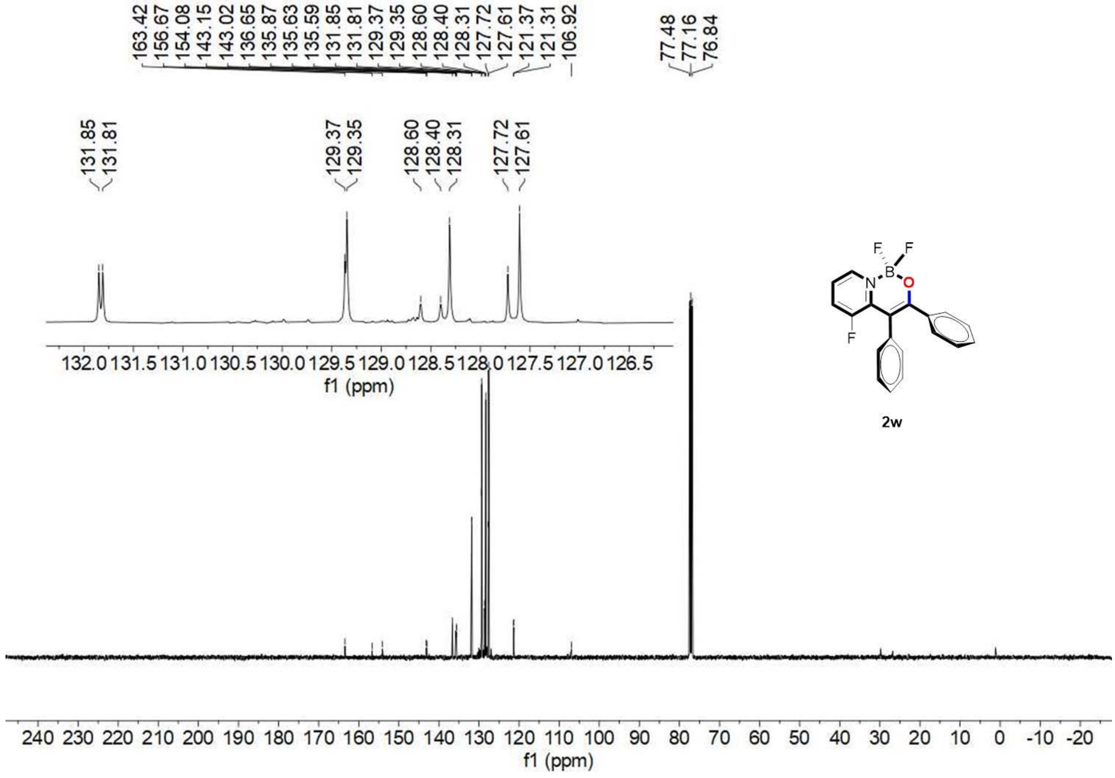
163.42 156.67 154.08 143.15 143.02 136.65 135.87 135.63 135.59 131.85 131.81 129.37 129.35 128.60 128.40 128.31 127.72 127.61 121.37 121.31 106.92
77.48 77.16 76.84
131.85 131.81 129.37 129.35 128.60 128.40 128.31 127.72 127.61
132.0 131.5 131.0 130.5 130.0 129.5 129.0 128.5 128.0 127.5 127.0 126.5
f1 (ppm)
240 230 220 210 200 190 180 170 160 150 140 130 120 110 100 90 80 70 60 50 40 30 20 10 0 -10 -20
f1 (ppm)
F
F
N
B
O
F

2w

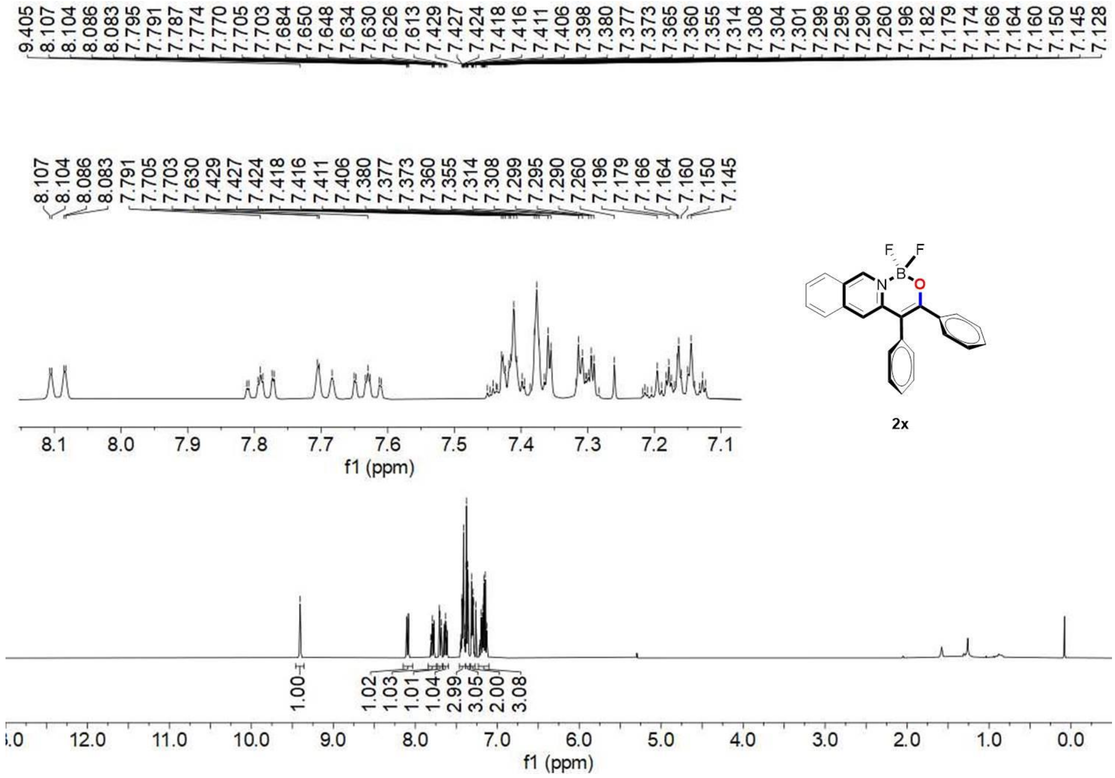
9.405 8.107 8.104 8.086 8.083 7.795 7.791 7.787 7.774 7.770 7.705 7.703 7.684 7.650 7.648 7.634 7.630 7.626 7.613 7.429 7.427 7.424 7.418 7.416 7.411 7.406 7.398 7.380 7.377 7.373 7.365 7.360 7.355 7.314 7.308 7.304 7.301 7.299 7.295 7.290 7.260 7.196 7.182 7.179 7.174 7.166 7.164 7.160 7.150 7.145 7.128
8.107 8.104 8.086 8.083 7.791 7.705 7.703 7.630 7.429 7.427 7.424 7.418 7.416 7.411 7.406 7.380 7.377 7.373 7.360 7.355 7.314 7.308 7.299 7.295 7.290 7.260 7.196 7.179 7.166 7.164 7.160 7.150 7.145
F
F
B
N
O
f1 (ppm)
1.00
1.02
1.03
1.01
1.04
2.99
3.05
2.00
3.08
f1 (ppm)

2x

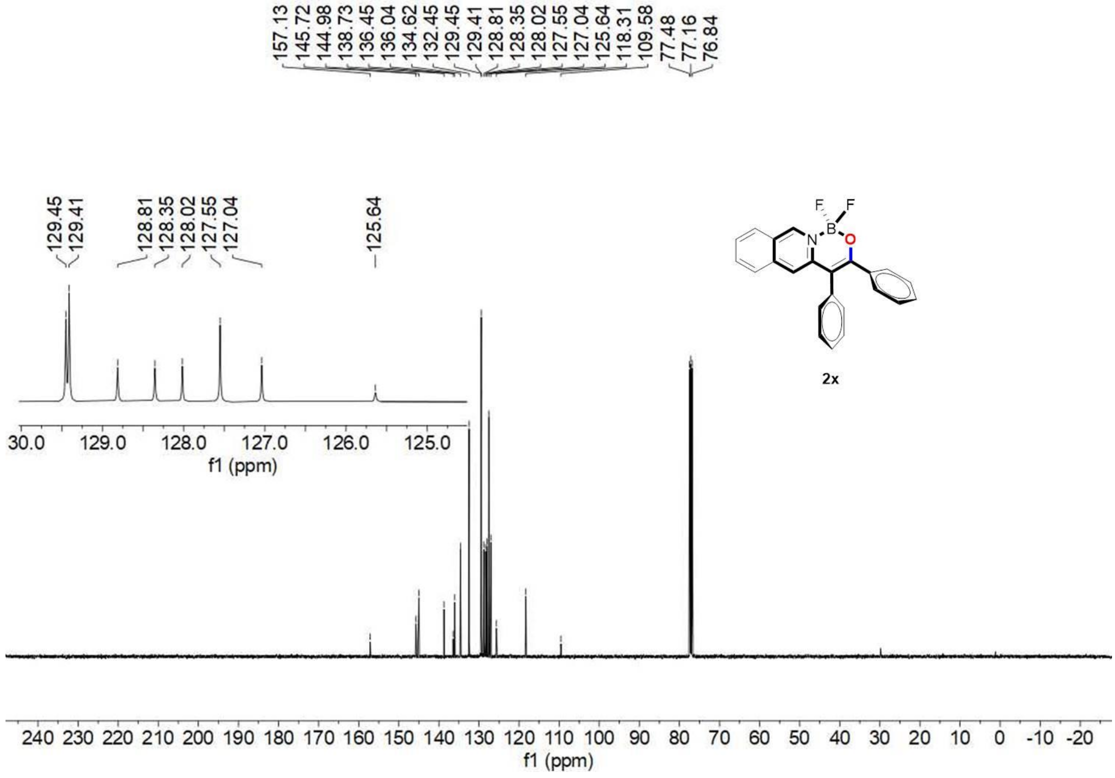
157.13 145.72 144.98 138.73 136.45 136.04 134.62 132.45 129.45 129.41 128.81 128.35 128.02 127.55 127.04 125.64 118.31 109.58 77.48 77.16 76.84
129.45 129.41 128.81 128.35 128.02 127.55 127.04 125.64
F
F
B
N
O
f1 (ppm)
f1 (ppm)

2x

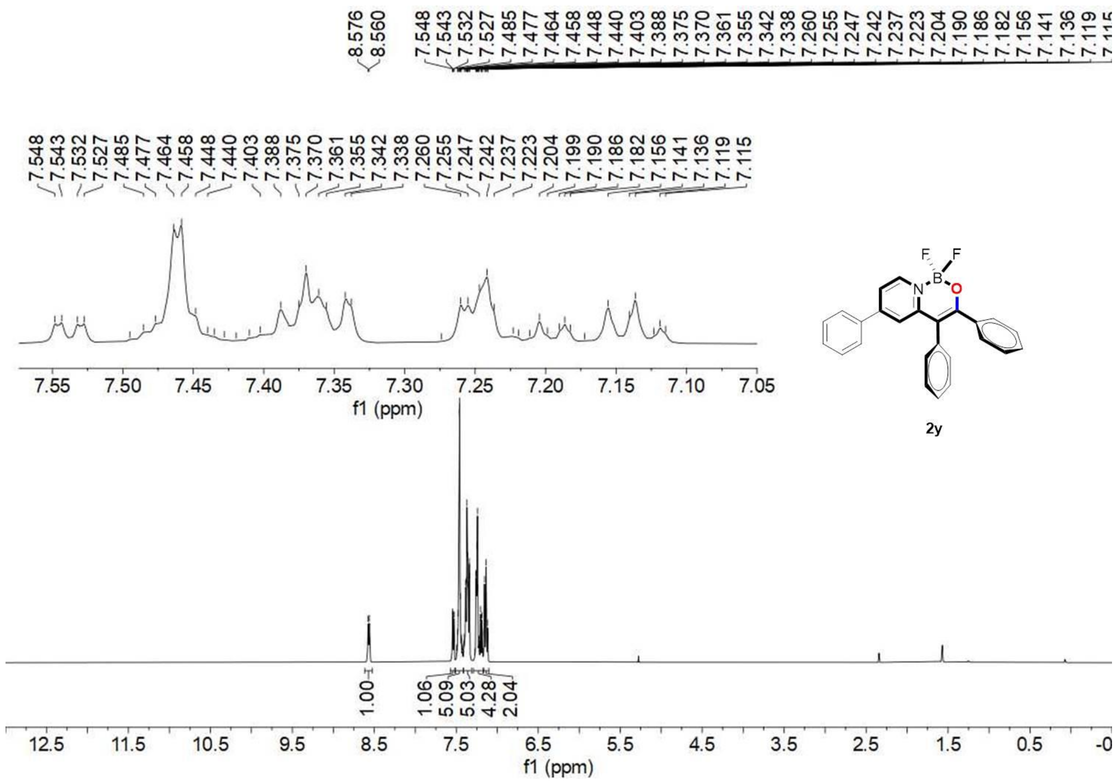
8.576
8.560
7.548
7.543
7.532
7.527
7.485
7.477
7.464
7.458
7.448
7.440
7.403
7.388
7.375
7.370
7.361
7.355
7.342
7.338
7.260
7.255
7.247
7.242
7.237
7.223
7.204
7.199
7.190
7.186
7.182
7.156
7.141
7.136
7.119
7.115
F
F
B
N
O
1.00
1.06
5.09
5.03
4.28
2.04
f1 (ppm)

2y

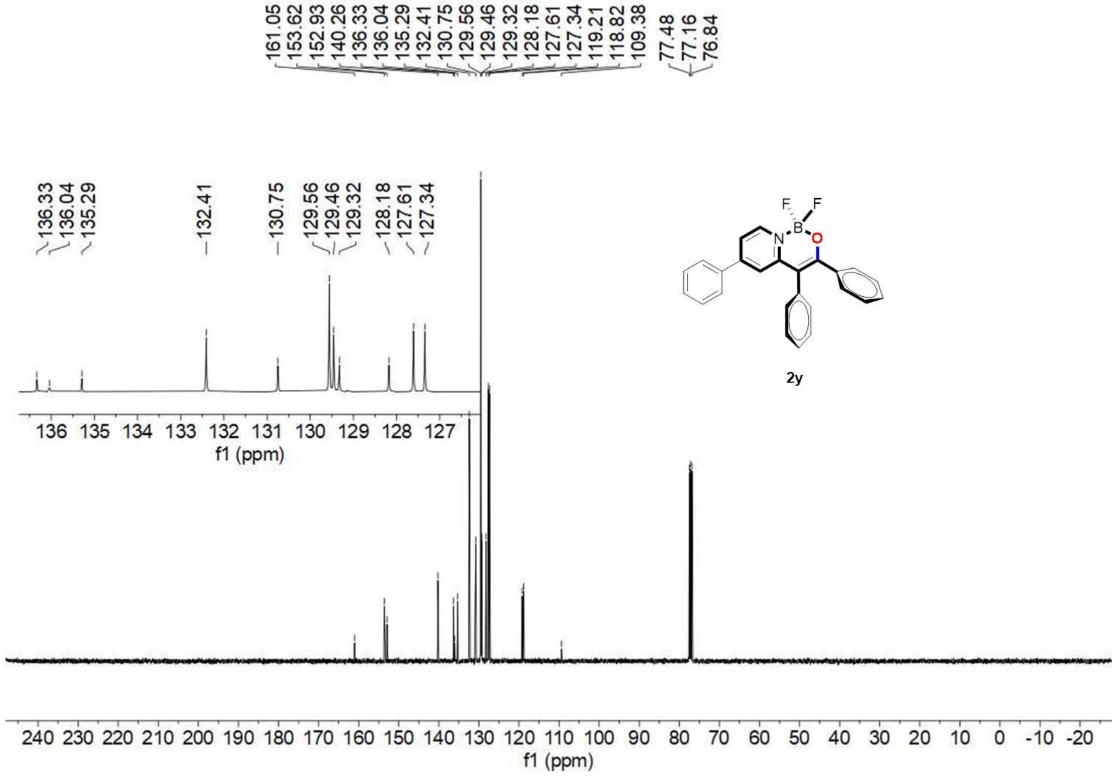
161.05
153.62
152.93
140.26
136.33
136.04
135.29
132.41
130.75
129.56
129.46
129.32
128.18
127.61
127.34
119.21
118.82
109.38
77.48
77.16
76.84
F
F
B
N
O
f1 (ppm)

2y

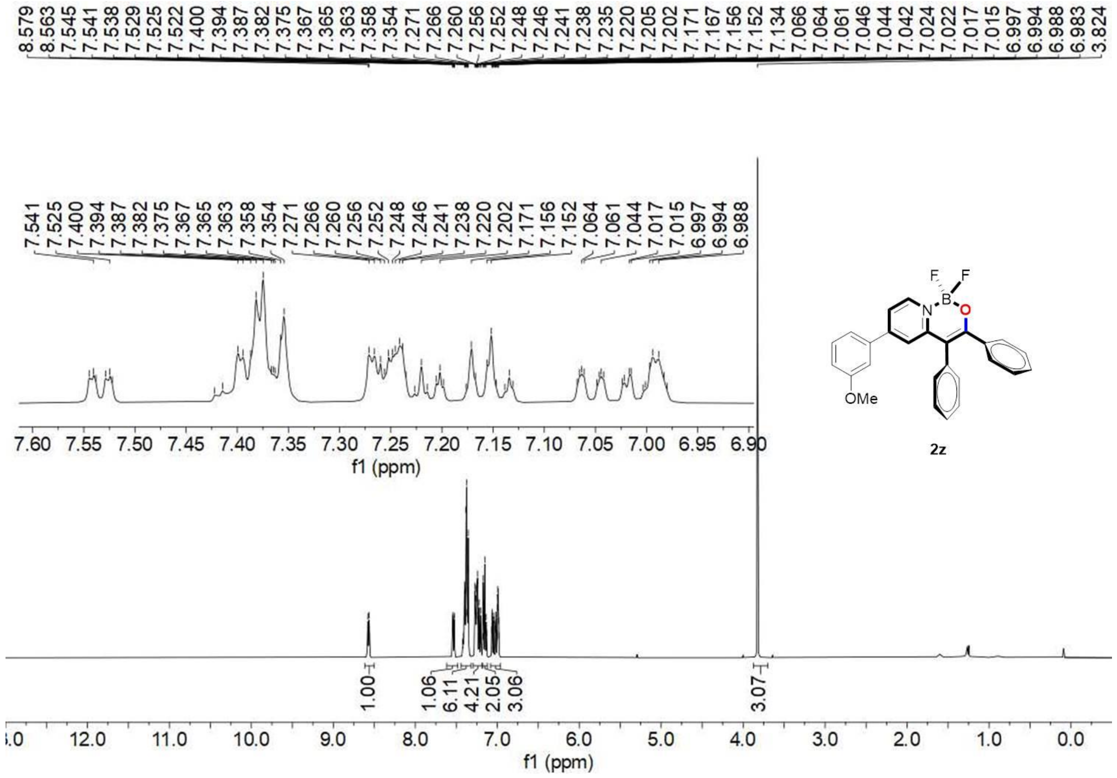
8.579 8.563 7.545 7.541 7.538 7.529 7.525 7.522 7.400 7.394 7.387 7.382 7.375 7.367 7.365 7.363 7.358 7.354 7.271 7.266 7.260 7.256 7.252 7.248 7.246 7.241 7.238 7.235 7.220 7.205 7.202 7.171 7.167 7.156 7.152 7.134 7.066 7.064 7.061 7.046 7.044 7.042 7.024 7.022 7.017 7.015 6.997 6.994 6.988 6.983 3.824
7.541 7.525 7.400 7.394 7.387 7.382 7.375 7.367 7.365 7.363 7.358 7.354 7.271 7.266 7.260 7.256 7.252 7.248 7.246 7.241 7.238 7.220 7.202 7.171 7.156 7.152 7.064 7.061 7.044 7.017 7.015 6.997 6.994 6.988
f1 (ppm)
F F B N O OMe
2z
1.00 1.06 6.11 4.21 2.05 3.06 3.07
f1 (ppm)

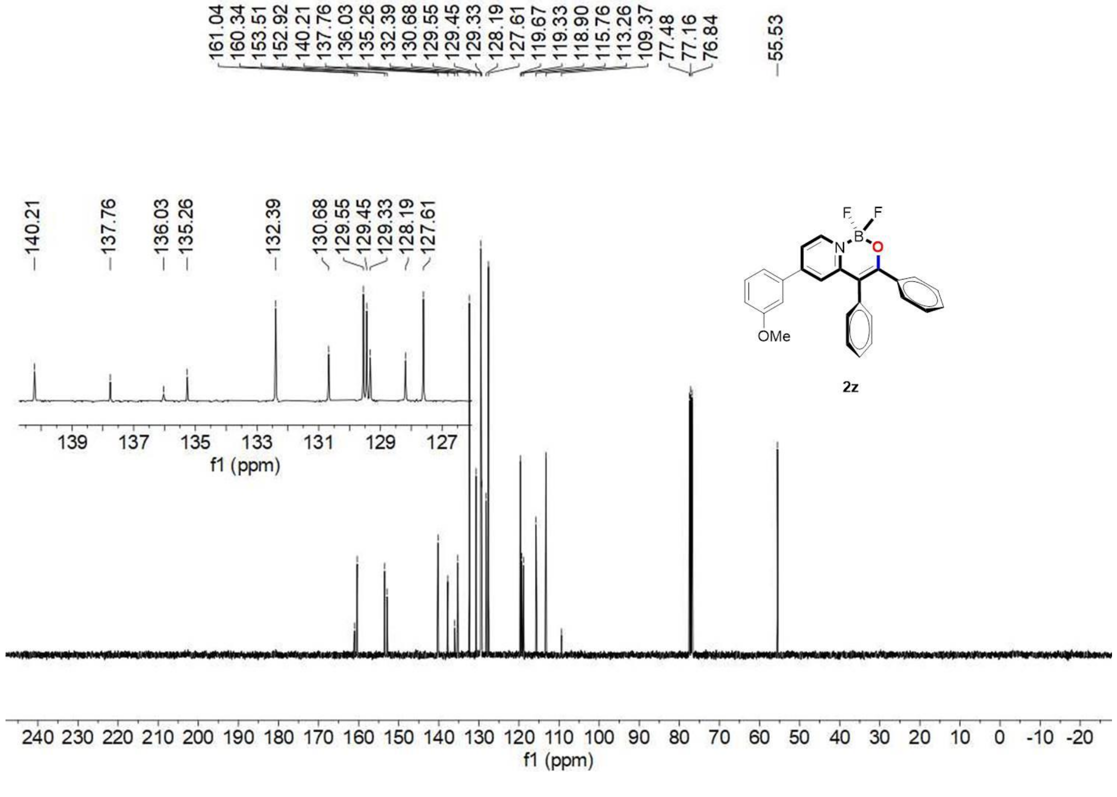
161.04 160.34 153.51 152.92 140.21 137.76 136.03 135.26 132.39 130.68 129.55 129.45 129.33 128.19 127.61 119.67 119.33 118.90 115.76 113.26 109.37 77.48 77.16 76.84 55.53
140.21 137.76 136.03 135.26 132.39 130.68 129.55 129.45 129.33 128.19 127.61
f1 (ppm)
F F B N O OMe
2z
f1 (ppm)

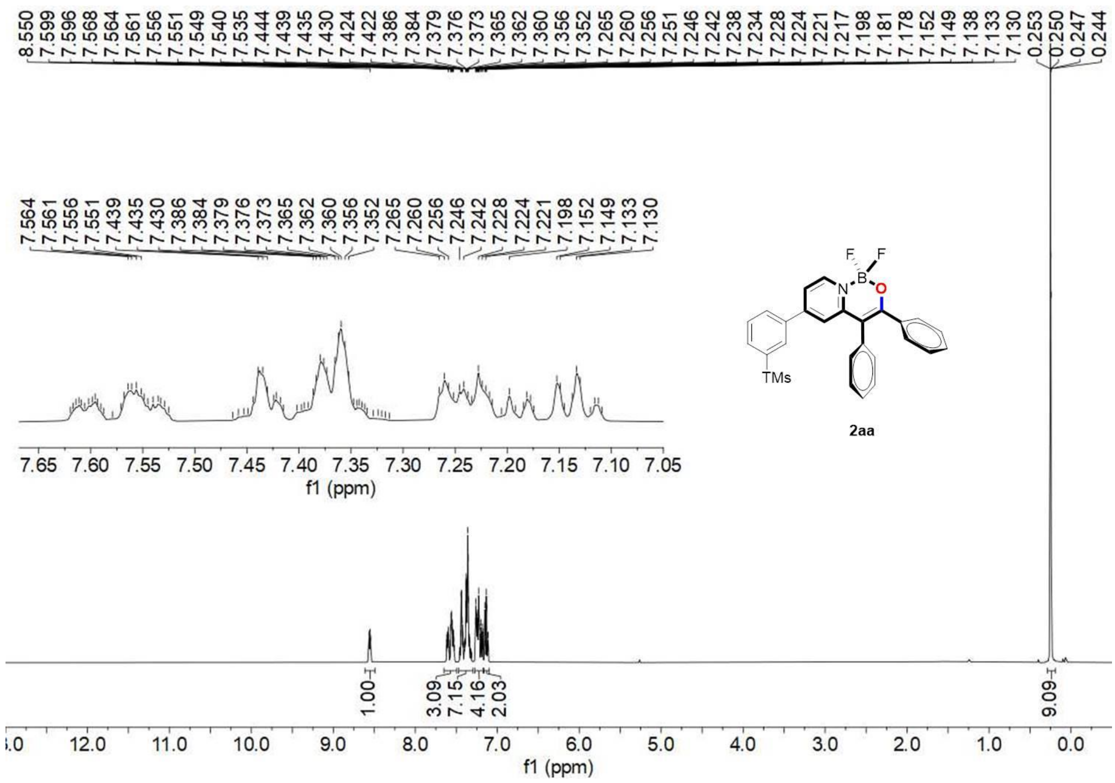
8.550
7.599
7.596
7.568
7.564
7.561
7.556
7.551
7.549
7.540
7.535
7.444
7.439
7.435
7.430
7.424
7.422
7.386
7.384
7.379
7.376
7.373
7.365
7.362
7.360
7.356
7.352
7.265
7.260
7.256
7.251
7.246
7.242
7.238
7.234
7.228
7.224
7.221
7.217
7.198
7.181
7.178
7.152
7.149
7.138
7.133
7.130
0.253
0.250
0.247
0.244
f1 (ppm)
1.00
3.09
7.15
4.16
2.03
9.09
TMs
2aa

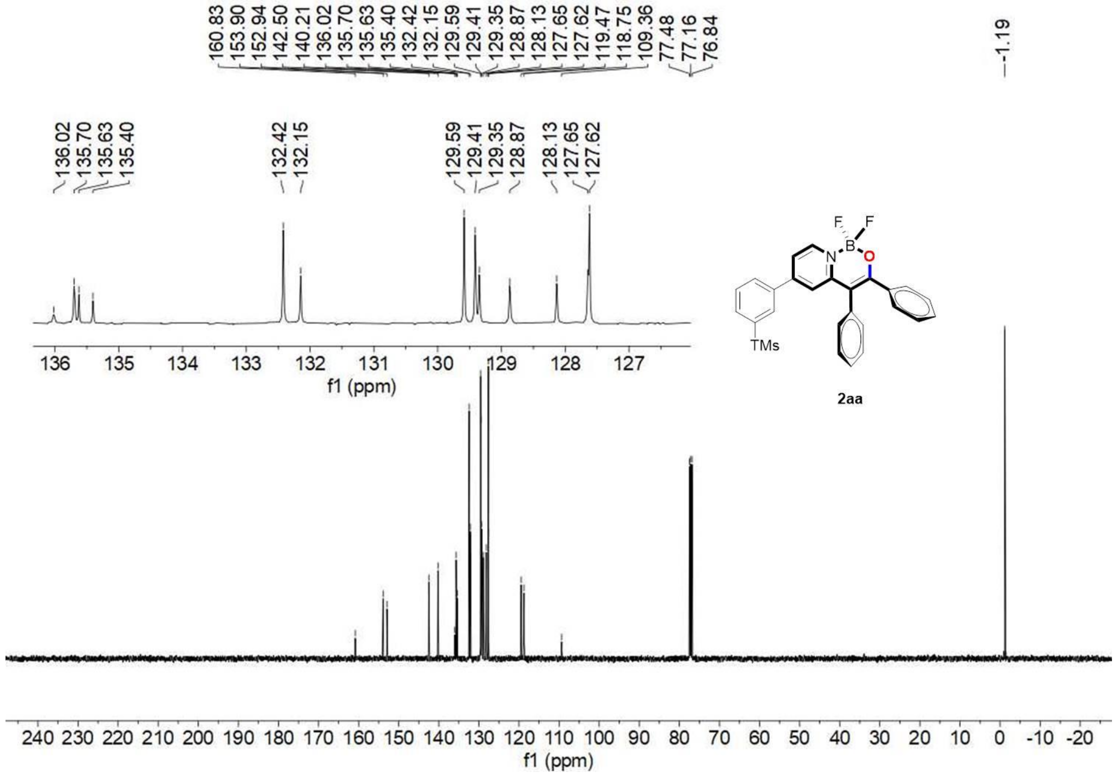
160.83
153.90
152.94
142.50
140.21
136.02
135.70
135.63
135.40
132.42
132.15
129.59
129.41
129.35
128.87
128.13
127.65
127.62
119.47
118.75
109.36
77.48
77.16
76.84
-1.19
f1 (ppm)
TMs
2aa

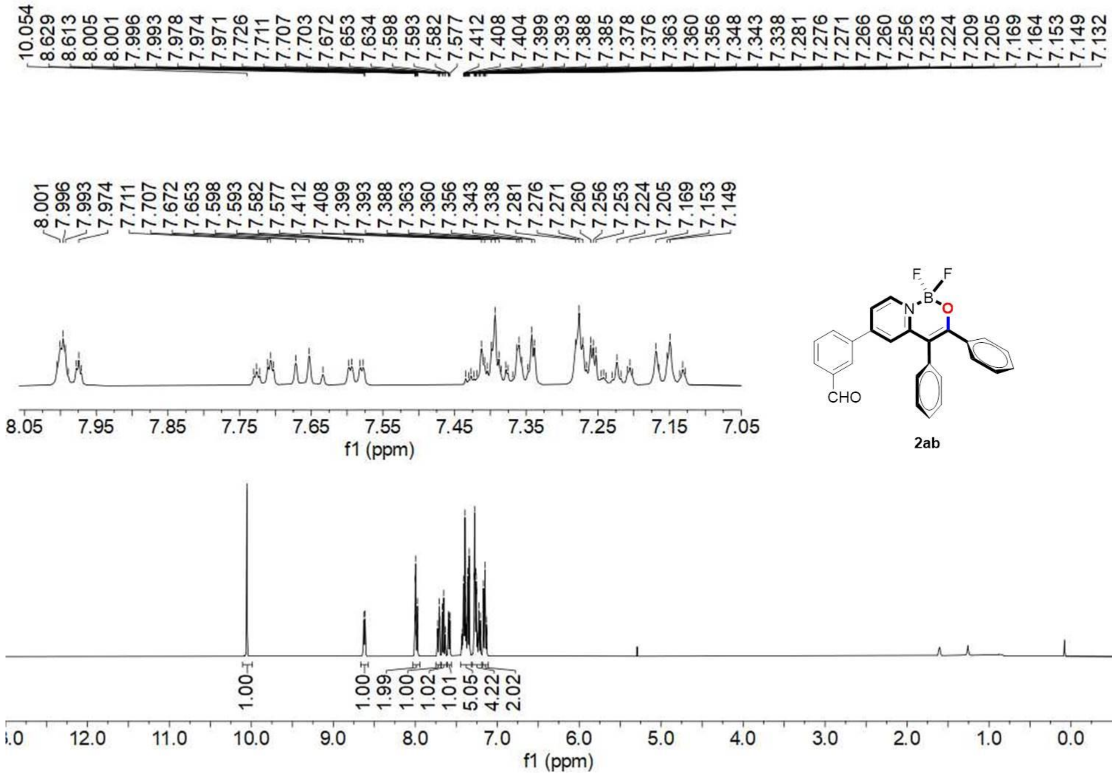
10.054
8.629
8.613
8.005
8.001
7.996
7.993
7.978
7.974
7.971
7.726
7.711
7.707
7.703
7.672
7.653
7.634
7.598
7.593
7.582
7.577
7.412
7.408
7.404
7.399
7.393
7.388
7.385
7.378
7.376
7.363
7.360
7.356
7.348
7.343
7.338
7.281
7.276
7.271
7.266
7.260
7.256
7.253
7.224
7.209
7.205
7.169
7.164
7.153
7.149
7.132
F
F
B
N
O
CHO
2ab
8.05 7.95 7.85 7.75 7.65 7.55 7.45 7.35 7.25 7.15 7.05
f1 (ppm)
1.00
1.00
1.99
1.00
1.02
1.01
5.05
4.22
2.02
12.0 11.0 10.0 9.0 8.0 7.0 6.0 5.0 4.0 3.0 2.0 1.0 0.0
f1 (ppm)

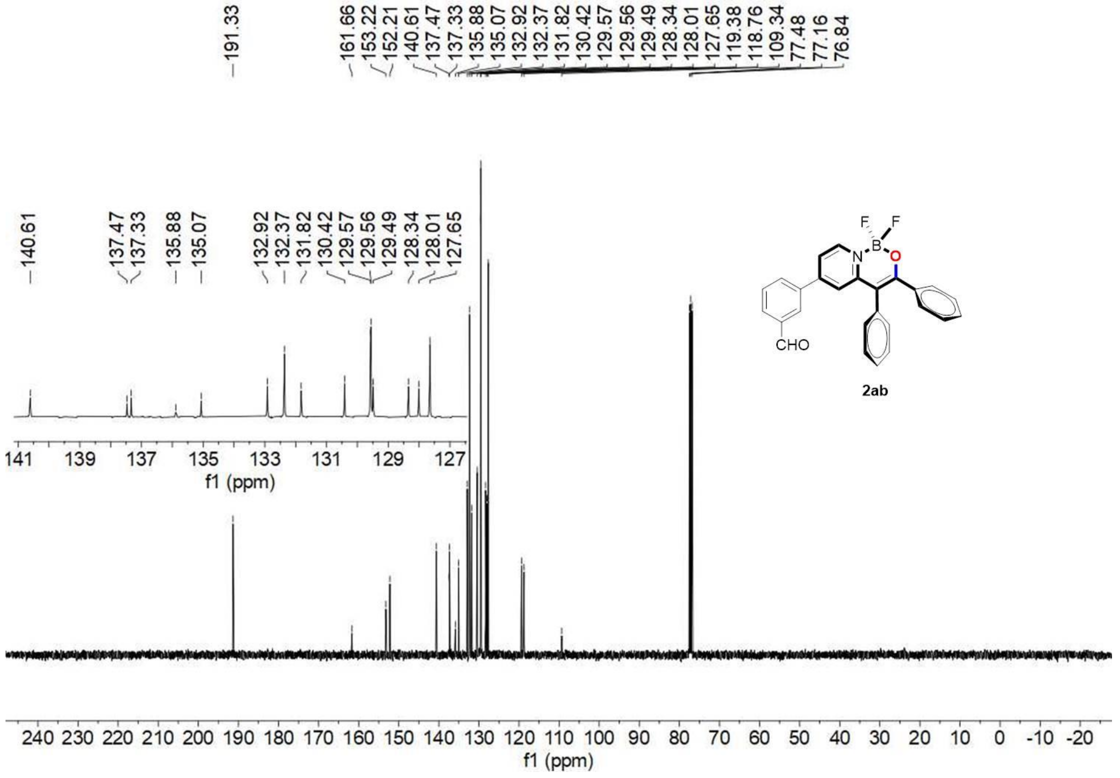
191.33
161.66
153.22
152.21
140.61
137.47
137.33
135.88
135.07
132.92
132.37
131.82
130.42
129.57
129.56
129.49
128.34
128.01
127.65
119.38
118.76
109.34
77.48
77.16
76.84
F
F
B
N
O
CHO
2ab
141 139 137 135 133 131 129 127
f1 (ppm)
240 230 220 210 200 190 180 170 160 150 140 130 120 110 100 90 80 70 60 50 40 30 20 10 0 -10 -20
f1 (ppm)

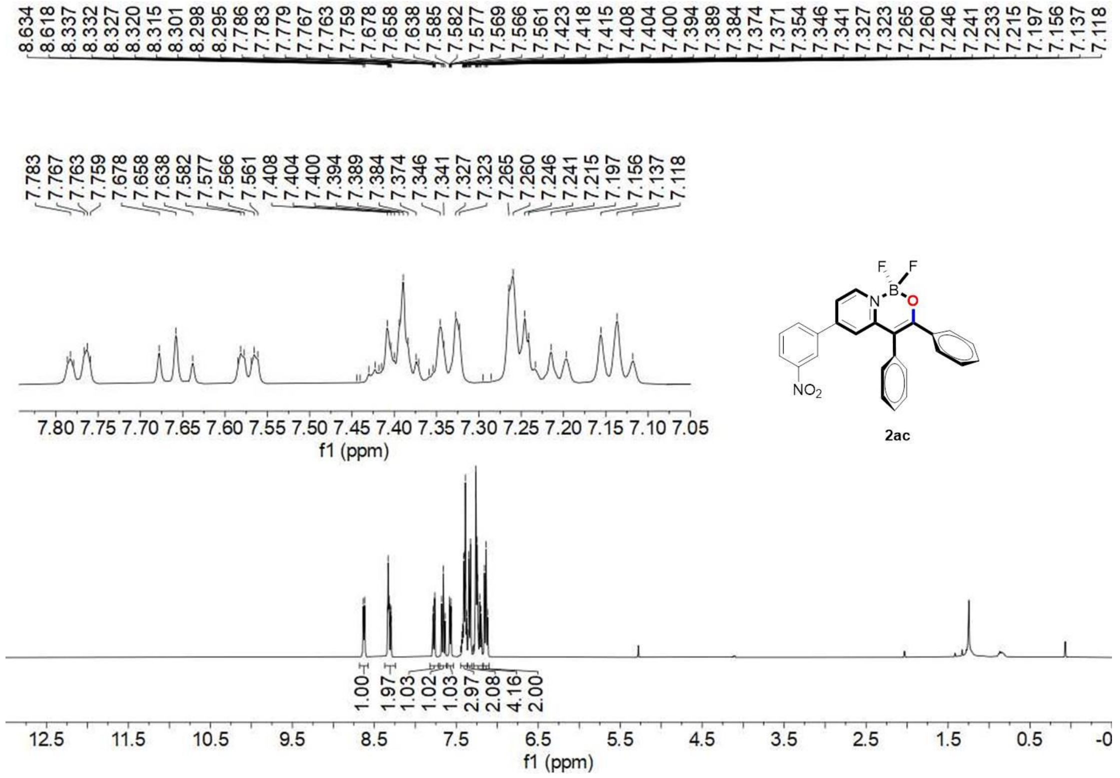
8.634 8.618 8.337 8.332 8.327 8.320 8.315 8.301 8.298 8.295 7.786 7.783 7.779 7.767 7.763 7.759 7.678 7.658 7.638 7.585 7.582 7.577 7.569 7.566 7.561 7.423 7.418 7.415 7.408 7.404 7.400 7.394 7.389 7.384 7.374 7.371 7.354 7.346 7.341 7.327 7.323 7.265 7.260 7.246 7.241 7.233 7.215 7.197 7.156 7.137 7.118
7.783 7.767 7.763 7.759 7.678 7.658 7.638 7.582 7.577 7.566 7.561 7.408 7.404 7.400 7.394 7.389 7.384 7.374 7.346 7.341 7.327 7.323 7.265 7.260 7.246 7.241 7.215 7.197 7.156 7.137 7.118
7.80 7.75 7.70 7.65 7.60 7.55 7.50 7.45 7.40 7.35 7.30 7.25 7.20 7.15 7.10 7.05
f1 (ppm)
F F B N O NO2
2ac
1.00 1.97 1.03 1.02 1.03 1.03 2.97 2.08 4.16 2.00
12.5 11.5 10.5 9.5 8.5 7.5 6.5 5.5 4.5 3.5 2.5 1.5 0.5 -0
f1 (ppm)

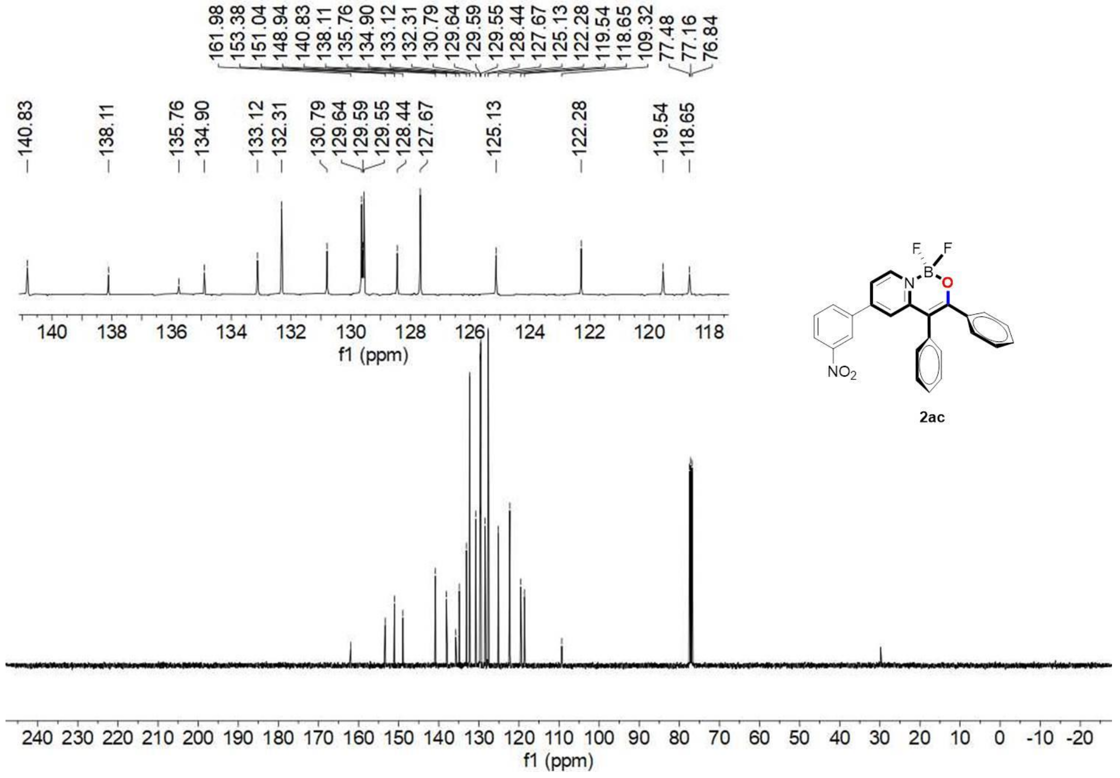
161.98 153.38 151.04 148.94 140.83 138.11 135.76 134.90 133.12 132.31 130.79 129.64 129.59 129.55 128.44 127.67 125.13 122.28 119.54 118.65 109.32 77.48 77.16 76.84
140.83 138.11 135.76 134.90 133.12 132.31 130.79 129.64 129.59 129.55 128.44 127.67 125.13 122.28 119.54 118.65
140 138 136 134 132 130 128 126 124 122 120 118
f1 (ppm)
F F B N O NO2
2ac
240 230 220 210 200 190 180 170 160 150 140 130 120 110 100 90 80 70 60 50 40 30 20 10 0 -10 -20
f1 (ppm)

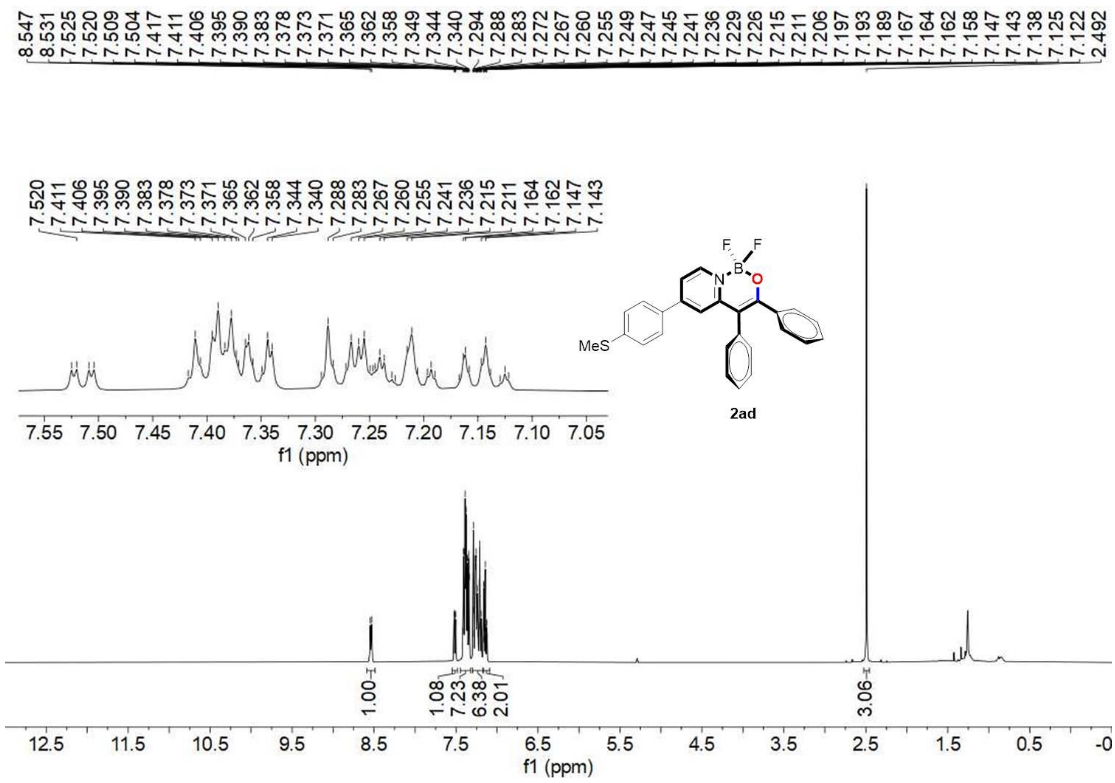
8.547 8.531 7.525 7.520 7.509 7.504 7.417 7.411 7.406 7.395 7.390 7.383 7.378 7.373 7.371 7.365 7.362 7.358 7.349 7.344 7.340 7.294 7.288 7.283 7.272 7.267 7.260 7.255 7.249 7.247 7.245 7.241 7.236 7.229 7.226 7.215 7.211 7.206 7.197 7.193 7.189 7.167 7.164 7.162 7.158 7.147 7.143 7.138 7.125 7.122 2.492
MeS
F F B N O
2ad
1.00 1.08 7.23 6.38 2.01 3.06
f1 (ppm)

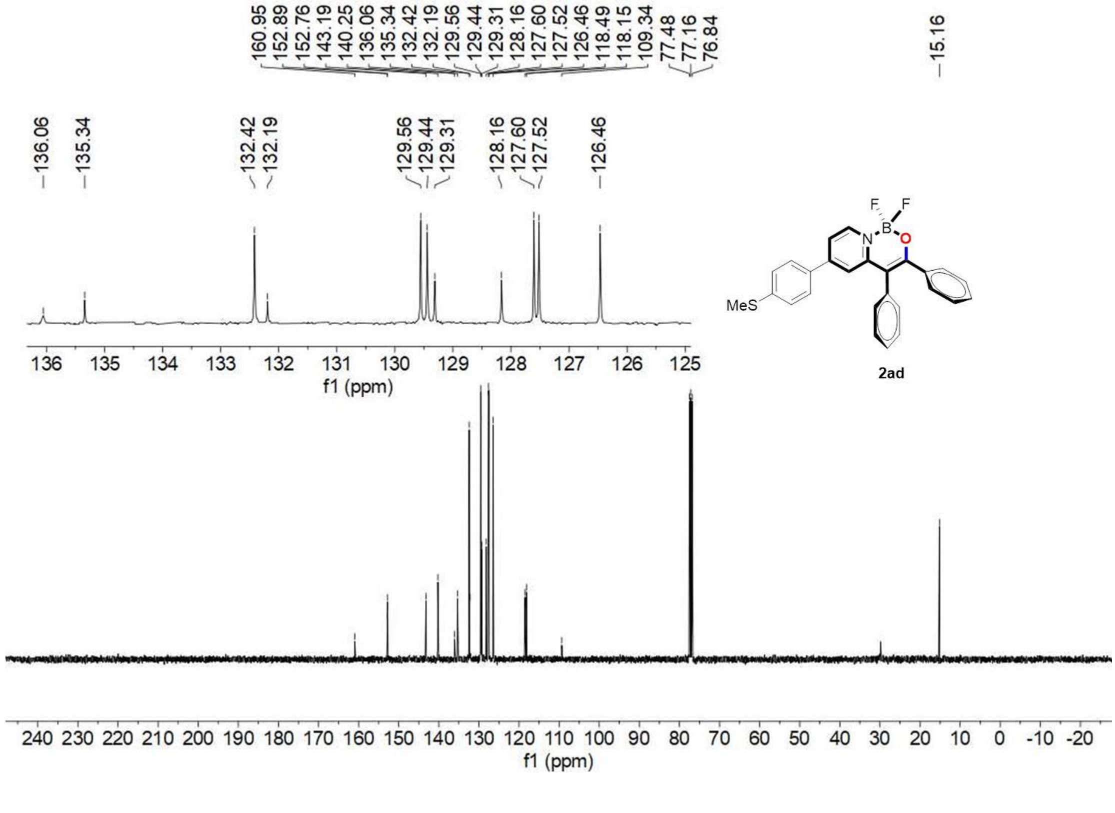
160.95 152.89 152.76 143.19 140.25 136.06 135.34 132.42 132.19 129.56 129.44 129.31 128.16 127.60 127.52 126.46 118.49 118.15 109.34 77.48 77.16 76.84 15.16
MeS
F F B N O
2ad
f1 (ppm)

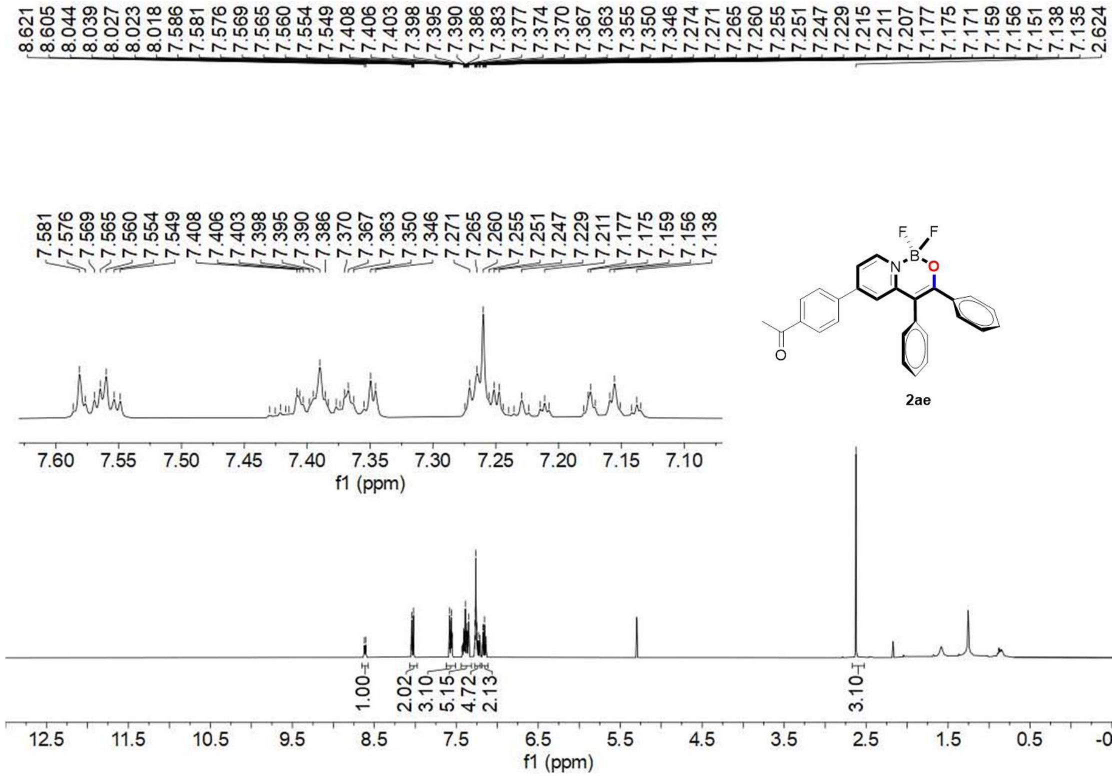
8.621 8.605 8.044 8.039 8.027 8.023 8.018 7.586 7.581 7.576 7.569 7.565 7.560 7.554 7.549 7.408 7.406 7.403 7.398 7.395 7.390 7.386 7.383 7.377 7.374 7.370 7.367 7.363 7.355 7.350 7.346 7.274 7.271 7.265 7.260 7.255 7.251 7.247 7.229 7.215 7.211 7.207 7.177 7.175 7.171 7.159 7.156 7.151 7.138 7.135 2.624
2ae
1.00 2.02 3.10 5.15 4.72 2.13 3.10
f1 (ppm)

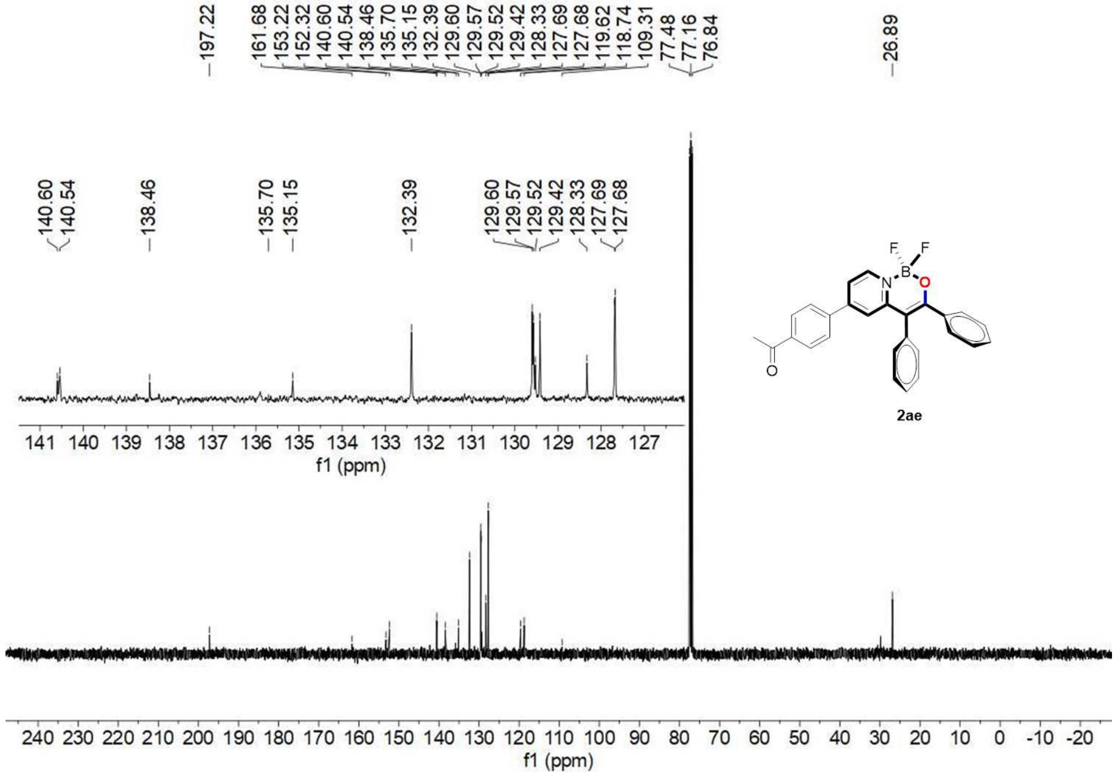
197.22 161.68 153.22 152.32 140.60 140.54 138.46 135.70 135.15 132.39 129.60 129.57 129.52 129.42 128.33 127.69 127.68 119.62 118.74 109.31 77.48 77.16 76.84 26.89
2ae
f1 (ppm)

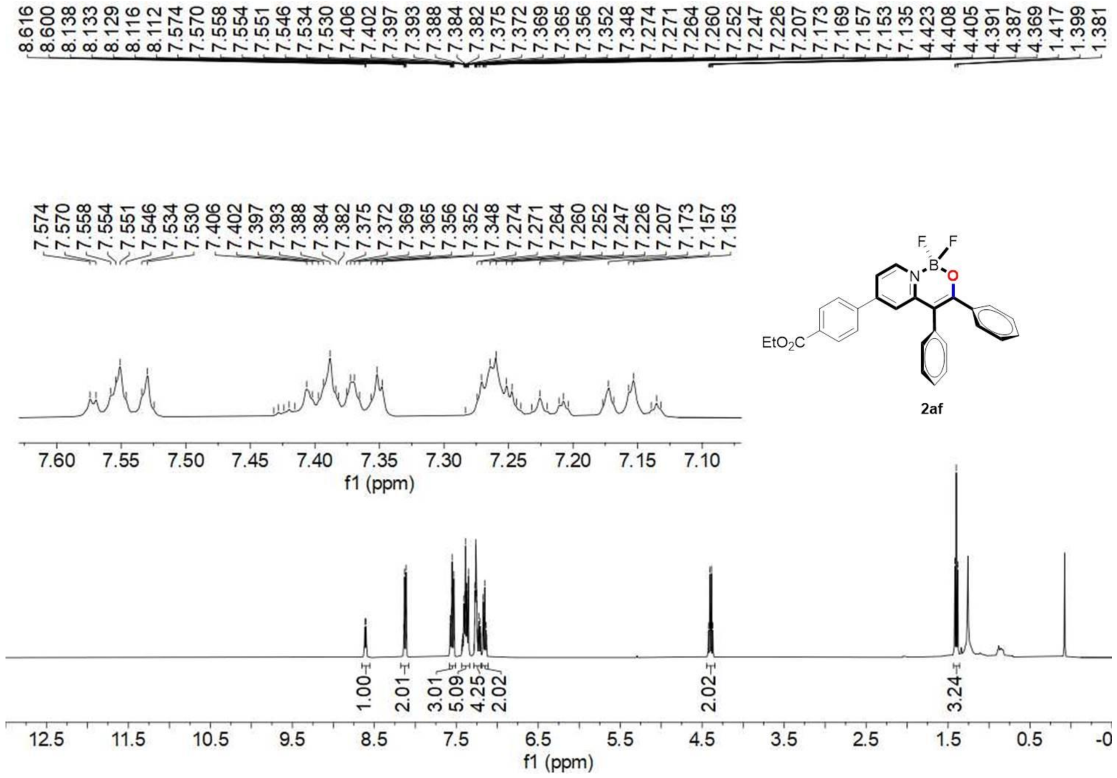
8.616 8.600 8.138 8.133 8.129 8.116 8.112 7.574 7.570 7.558 7.554 7.551 7.546 7.534 7.530 7.406 7.402 7.397 7.393 7.388 7.384 7.382 7.375 7.372 7.369 7.365 7.356 7.352 7.348 7.274 7.271 7.264 7.260 7.252 7.247 7.226 7.207 7.173 7.169 7.157 7.153 7.135 4.423 4.408 4.405 4.391 4.387 4.369 1.417 1.399 1.381
7.574 7.570 7.558 7.554 7.551 7.546 7.534 7.530 7.406 7.402 7.397 7.393 7.388 7.384 7.382 7.375 7.372 7.369 7.365 7.356 7.352 7.348 7.274 7.271 7.264 7.260 7.252 7.247 7.226 7.207 7.173 7.157 7.153
7.60 7.55 7.50 7.45 7.40 7.35 7.30 7.25 7.20 7.15 7.10
f1 (ppm)
F F B N O
EtO2C
2af
1.00 2.01 3.01 5.09 4.25 2.02 2.02 3.24
12.5 11.5 10.5 9.5 8.5 7.5 6.5 5.5 4.5 3.5 2.5 1.5 0.5 -0
f1 (ppm)

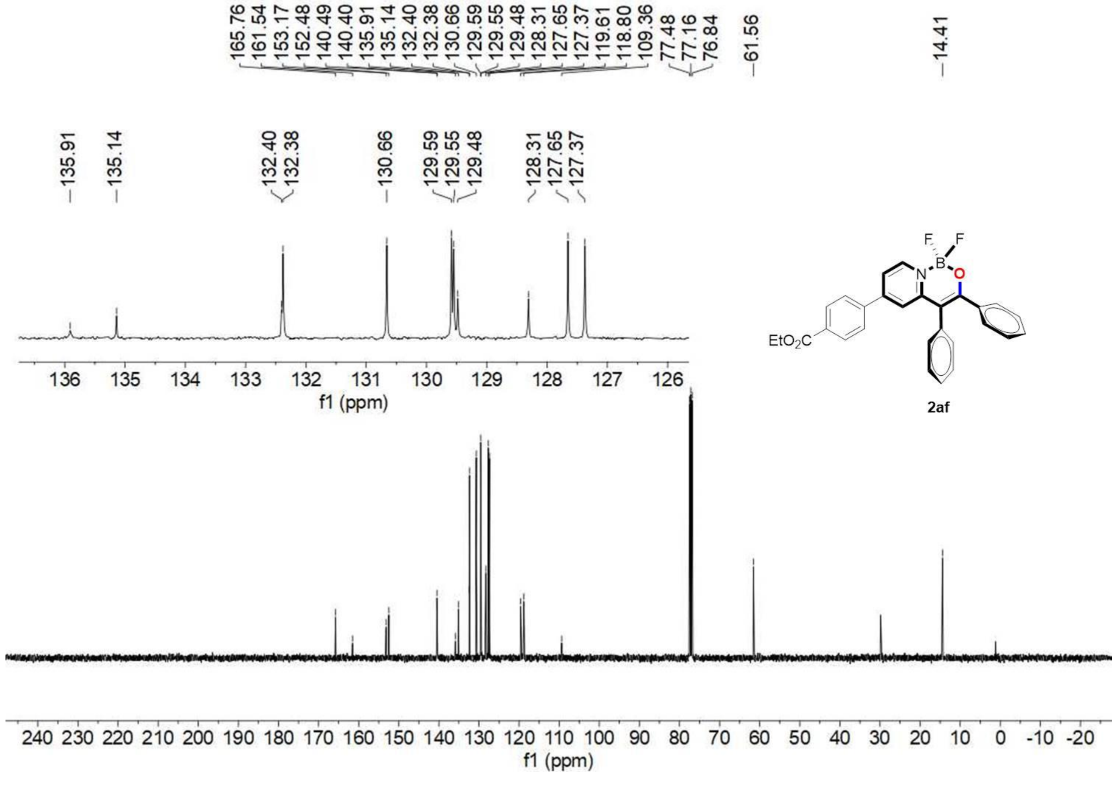
165.76 161.54 153.17 152.48 140.49 140.40 135.91 135.14 132.40 132.38 130.66 129.59 129.55 129.48 128.31 127.65 127.37 119.61 118.80 109.36 77.48 77.16 76.84 61.56 14.41
135.91 135.14 132.40 132.38 130.66 129.59 129.55 129.48 128.31 127.65 127.37
136 135 134 133 132 131 130 129 128 127 126
f1 (ppm)
F F B N O
EtO2C
2af
240 230 220 210 200 190 180 170 160 150 140 130 120 110 100 90 80 70 60 50 40 30 20 10 0 -10 -20
f1 (ppm)

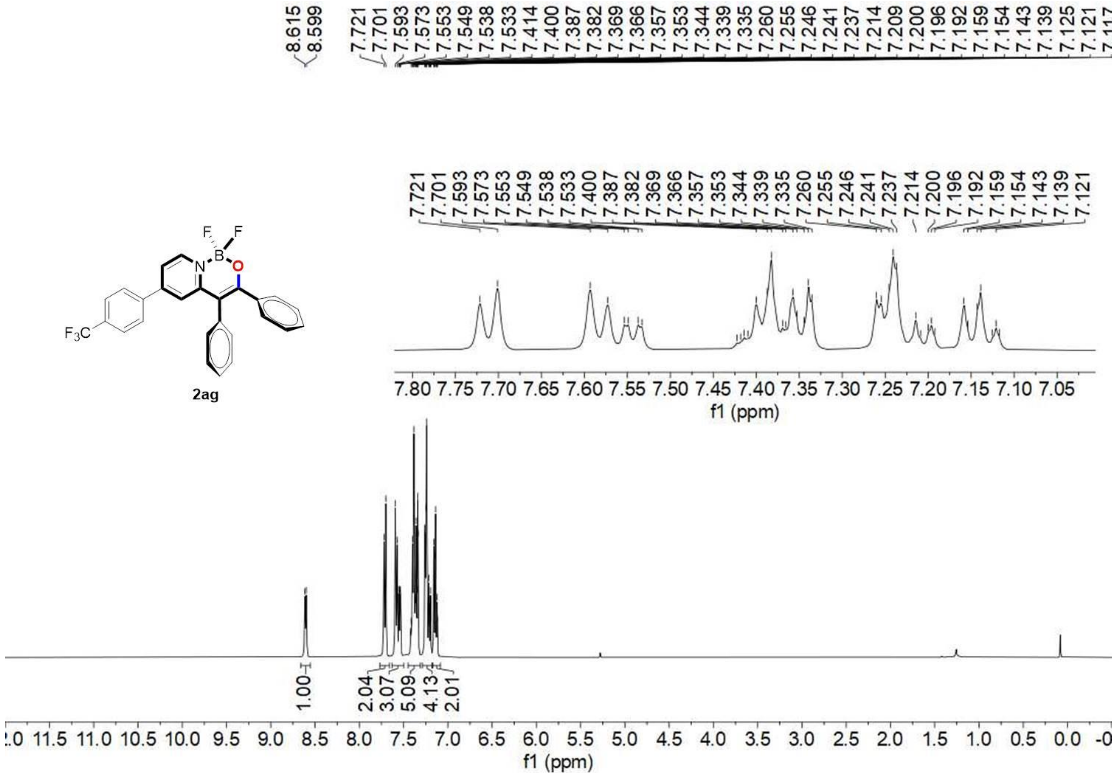
8.615
8.599
7.721
7.701
7.593
7.573
7.553
7.549
7.538
7.533
7.414
7.400
7.387
7.382
7.369
7.366
7.357
7.353
7.344
7.339
7.335
7.260
7.255
7.246
7.241
7.237
7.214
7.209
7.200
7.196
7.192
7.159
7.154
7.143
7.139
7.125
7.121
2ag
7.80 7.75 7.70 7.65 7.60 7.55 7.50 7.45 7.40 7.35 7.30 7.25 7.20 7.15 7.10 7.05
f1 (ppm)
1.00
2.04
3.07
5.09
4.13
2.01
f1 (ppm)

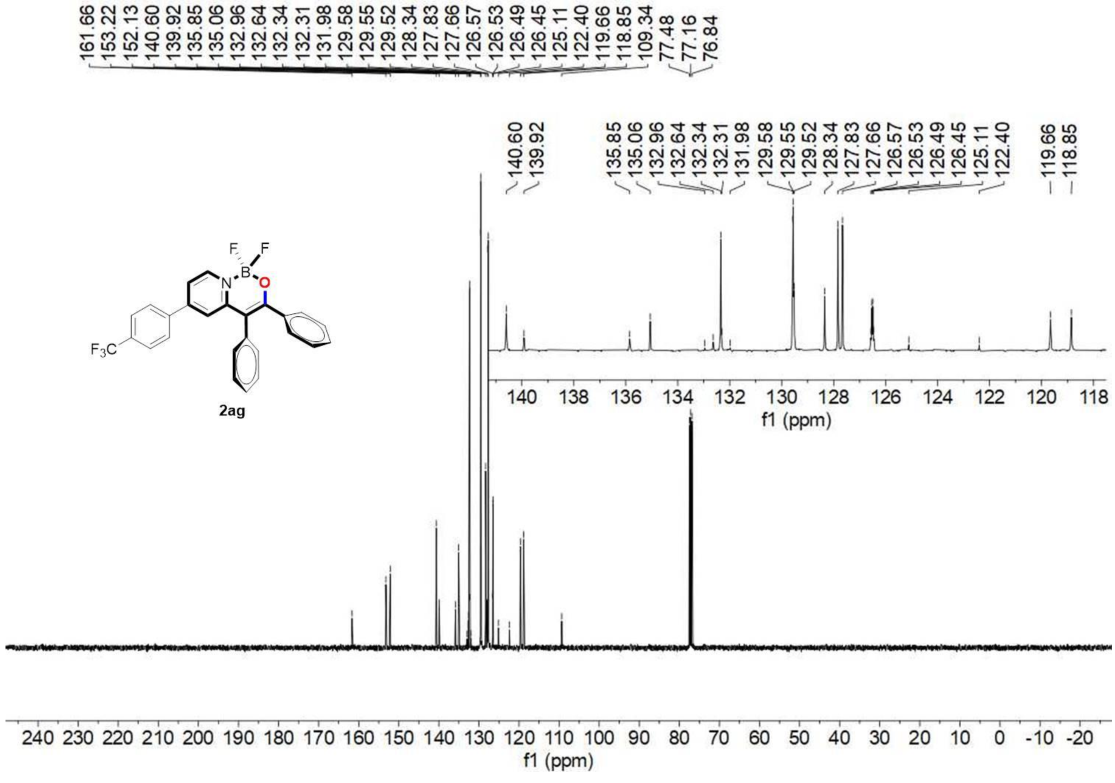
161.66
153.22
152.13
140.60
139.92
135.85
135.06
132.96
132.64
132.34
132.31
131.98
129.58
129.55
129.52
128.34
127.83
127.66
126.57
126.53
126.49
126.45
125.11
122.40
119.66
118.85
109.34
77.48
77.16
76.84
2ag
140 138 136 134 132 130 128 126 124 122 120 118
f1 (ppm)
240 230 220 210 200 190 180 170 160 150 140 130 120 110 100 90 80 70 60 50 40 30 20 10 0 -10 -20
f1 (ppm)

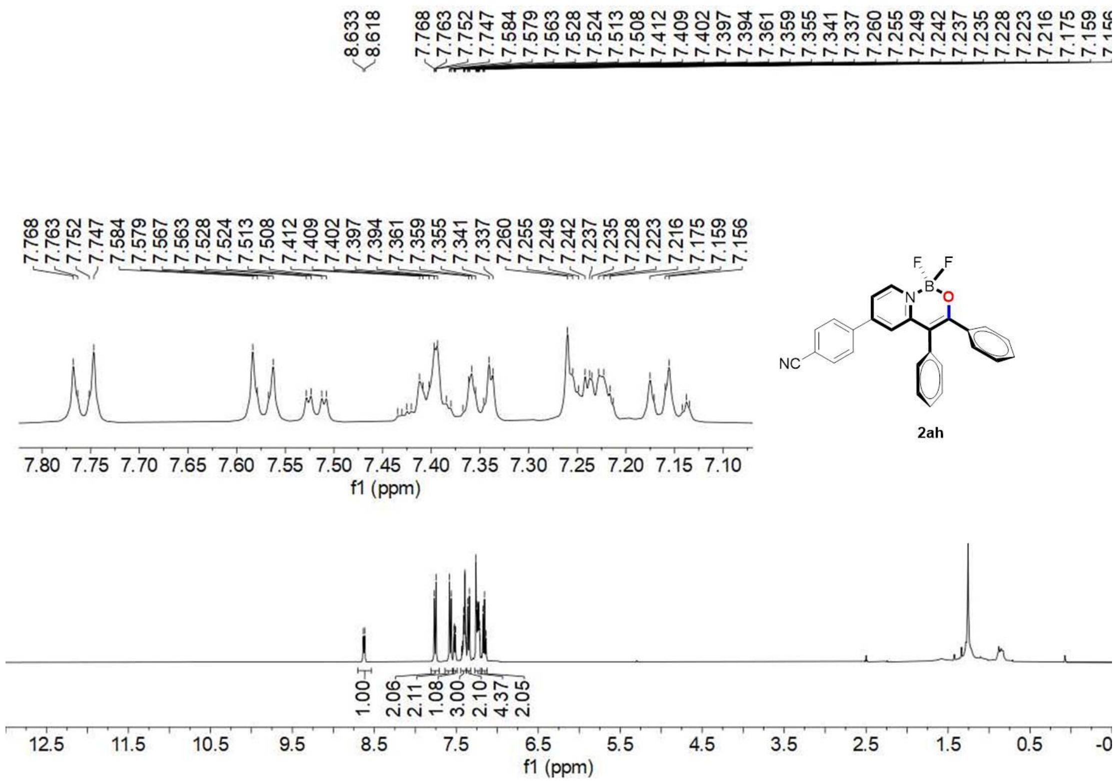

8.633
8.618
7.768
7.763
7.752
7.747
7.584
7.579
7.567
7.563
7.528
7.524
7.513
7.508
7.412
7.409
7.402
7.397
7.394
7.361
7.359
7.355
7.341
7.337
7.260
7.255
7.249
7.242
7.237
7.235
7.228
7.223
7.216
7.175
7.159
7.156
1.00
2.06
2.11
1.08
3.00
2.10
4.37
2.05
f1 (ppm)
NC
F
F
B
N
O

2ah

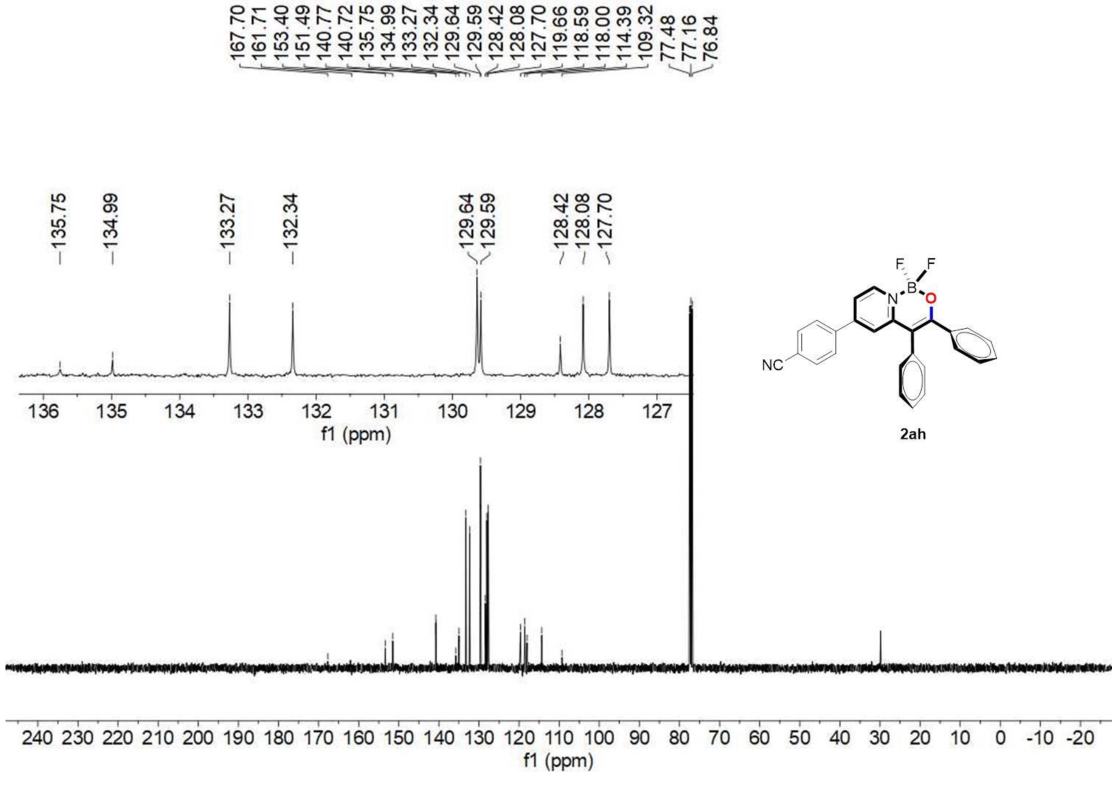

167.70
161.71
153.40
151.49
140.77
140.72
135.75
134.99
133.27
132.34
129.64
129.59
128.42
128.08
127.70
119.66
118.59
118.00
114.39
109.32
77.48
77.16
76.84
f1 (ppm)
NC
F
F
B
N
O

2ah

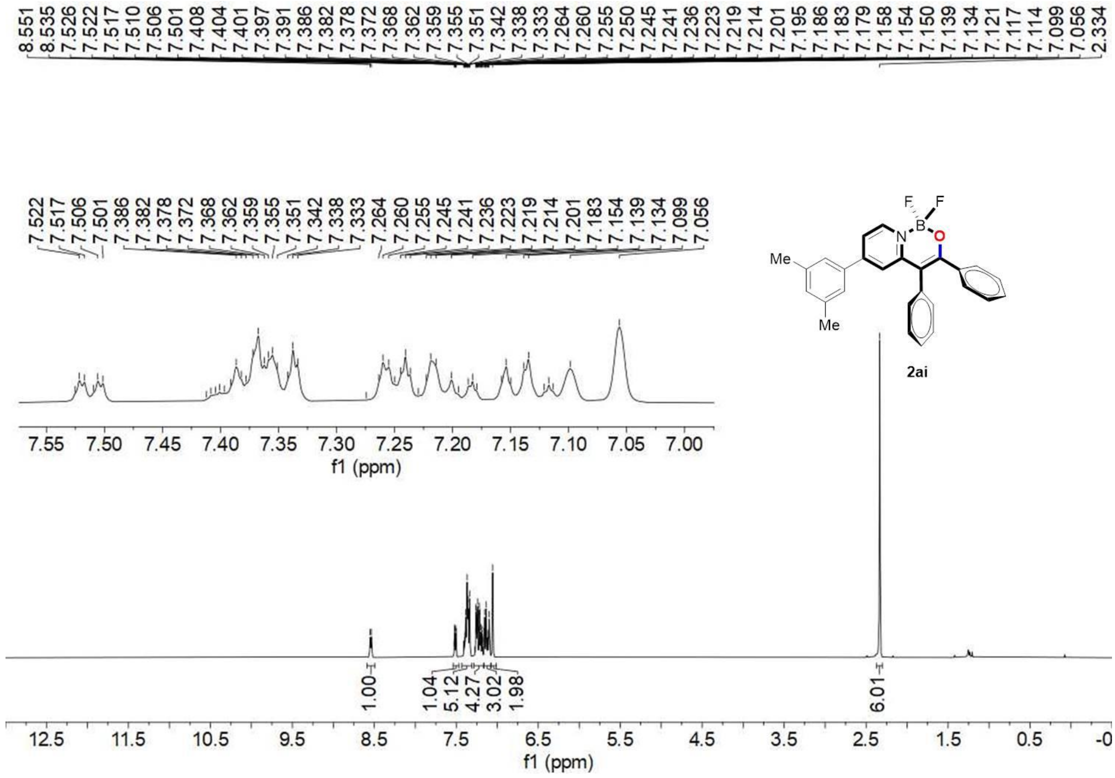
8.551 8.535 7.526 7.522 7.517 7.510 7.506 7.501 7.408 7.404 7.401 7.397 7.391 7.386 7.382 7.378 7.372 7.368 7.362 7.359 7.355 7.351 7.342 7.338 7.333 7.264 7.260 7.255 7.250 7.245 7.241 7.236 7.223 7.219 7.214 7.201 7.195 7.186 7.183 7.179 7.158 7.154 7.150 7.139 7.134 7.121 7.117 7.114 7.099 7.056 2.334
7.522 7.517 7.506 7.501 7.386 7.382 7.378 7.372 7.368 7.362 7.359 7.355 7.351 7.342 7.338 7.333 7.264 7.260 7.255 7.245 7.241 7.236 7.223 7.219 7.214 7.201 7.183 7.154 7.134 7.099 7.056
F F B N O Me Me
2ai
7.55 7.50 7.45 7.40 7.35 7.30 7.25 7.20 7.15 7.10 7.05 7.00
f1 (ppm)
1.00 1.04 5.12 4.27 3.02 1.98 6.01
12.5 11.5 10.5 9.5 8.5 7.5 6.5 5.5 4.5 3.5 2.5 1.5 0.5 -0
f1 (ppm)

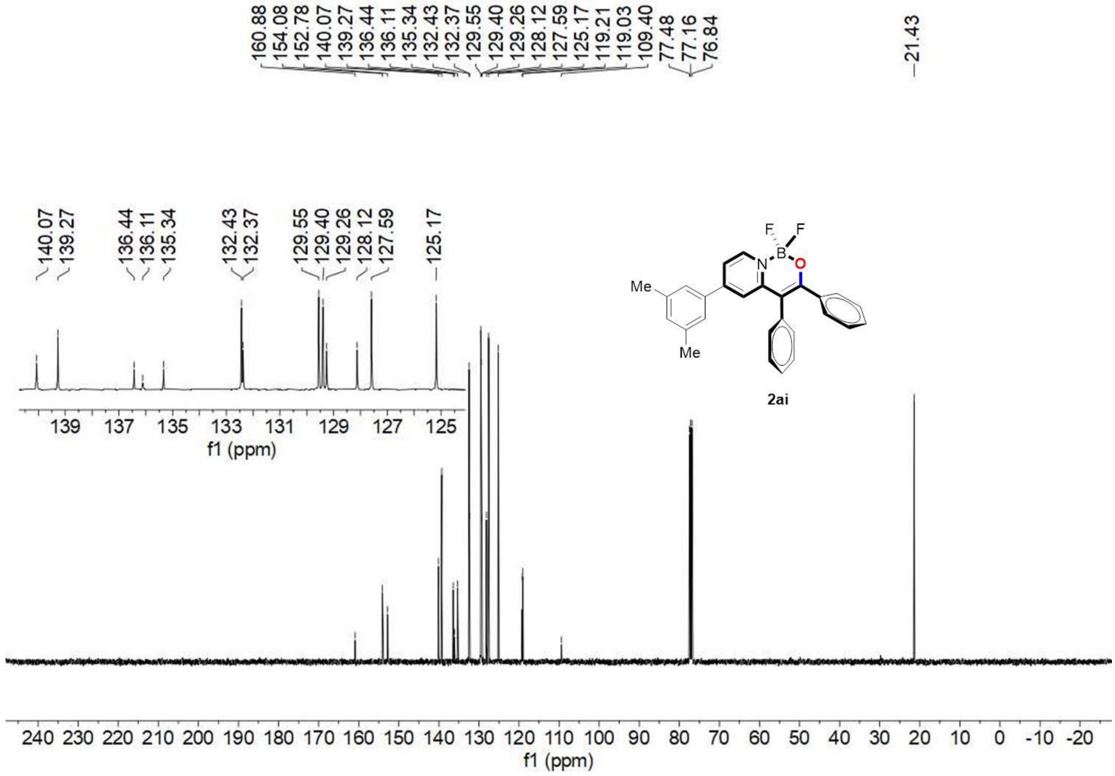
160.88 154.08 152.78 140.07 139.27 136.44 136.11 135.34 132.43 132.37 129.55 129.40 129.26 128.12 127.59 125.17 119.21 119.03 109.40 77.48 77.16 76.84
21.43
140.07 139.27 136.44 136.11 135.34 132.43 132.37 129.55 129.40 129.26 128.12 127.59 125.17
139 137 135 133 131 129 127 125
f1 (ppm)
F F B N O Me Me
2ai
240 230 220 210 200 190 180 170 160 150 140 130 120 110 100 90 80 70 60 50 40 30 20 10 0 -10 -20
f1 (ppm)

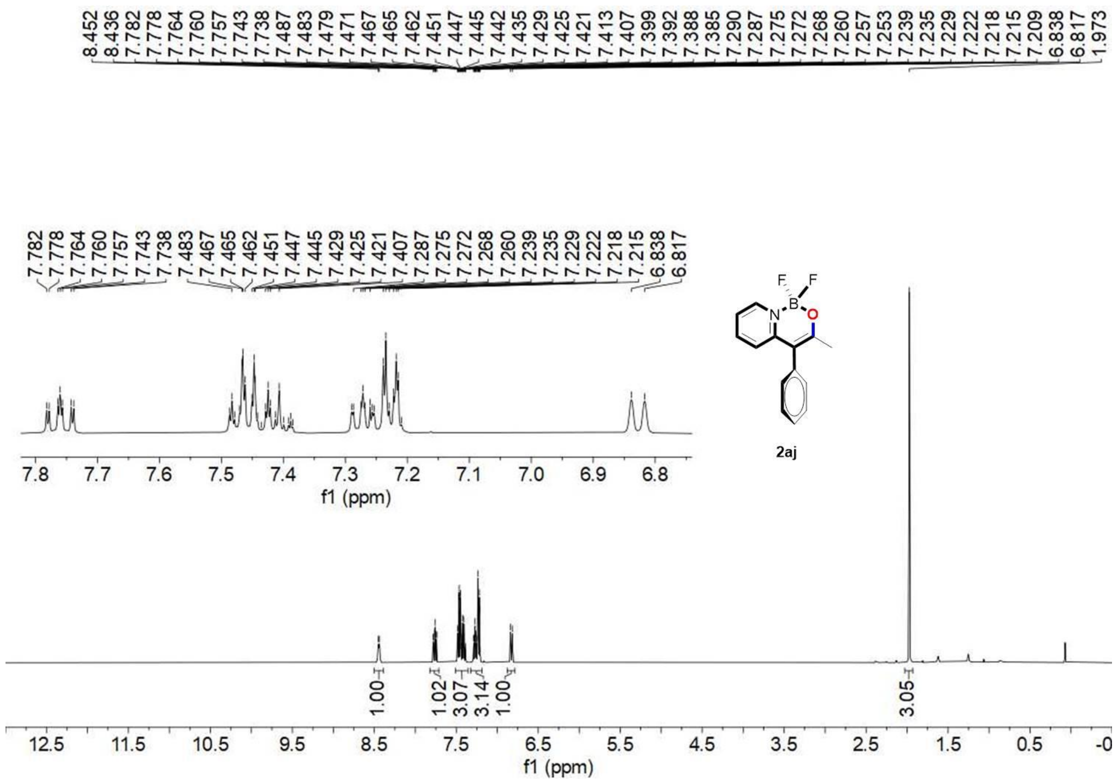
2aj
1.00
1.02
3.07
3.14
1.00
3.05
f1 (ppm)

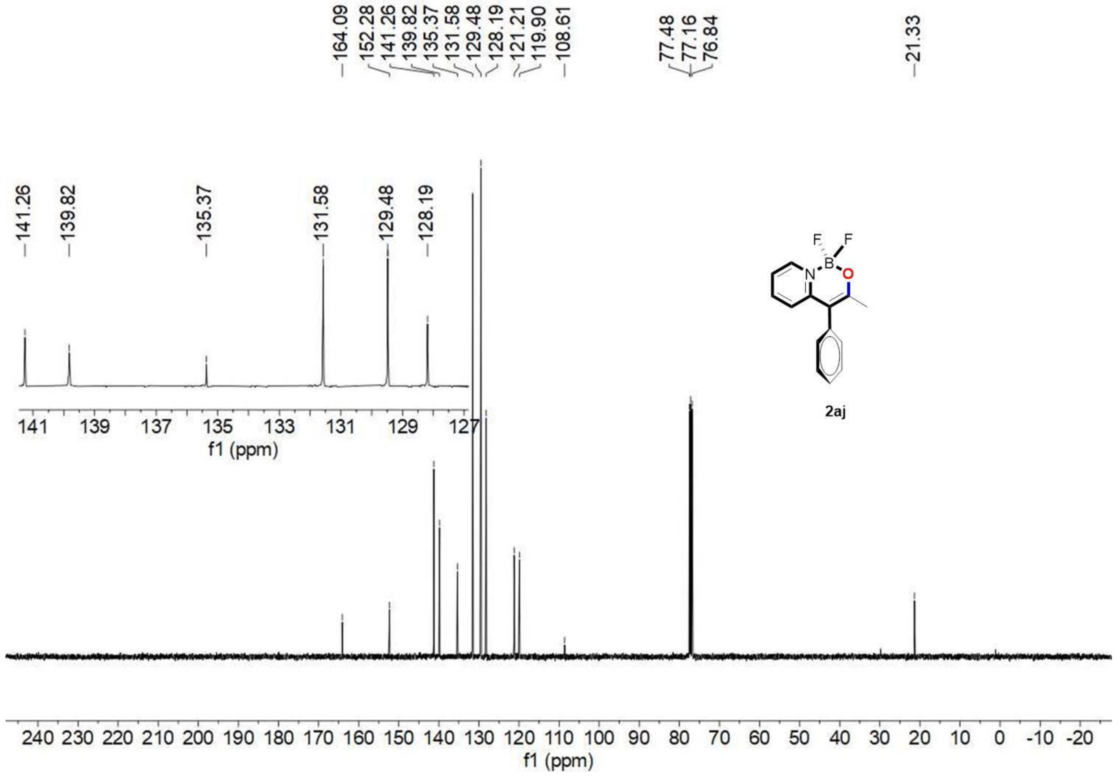
164.09
152.28
141.26
139.82
135.37
131.58
129.48
128.19
121.21
119.90
108.61
77.48
77.16
76.84
21.33
2aj
f1 (ppm)

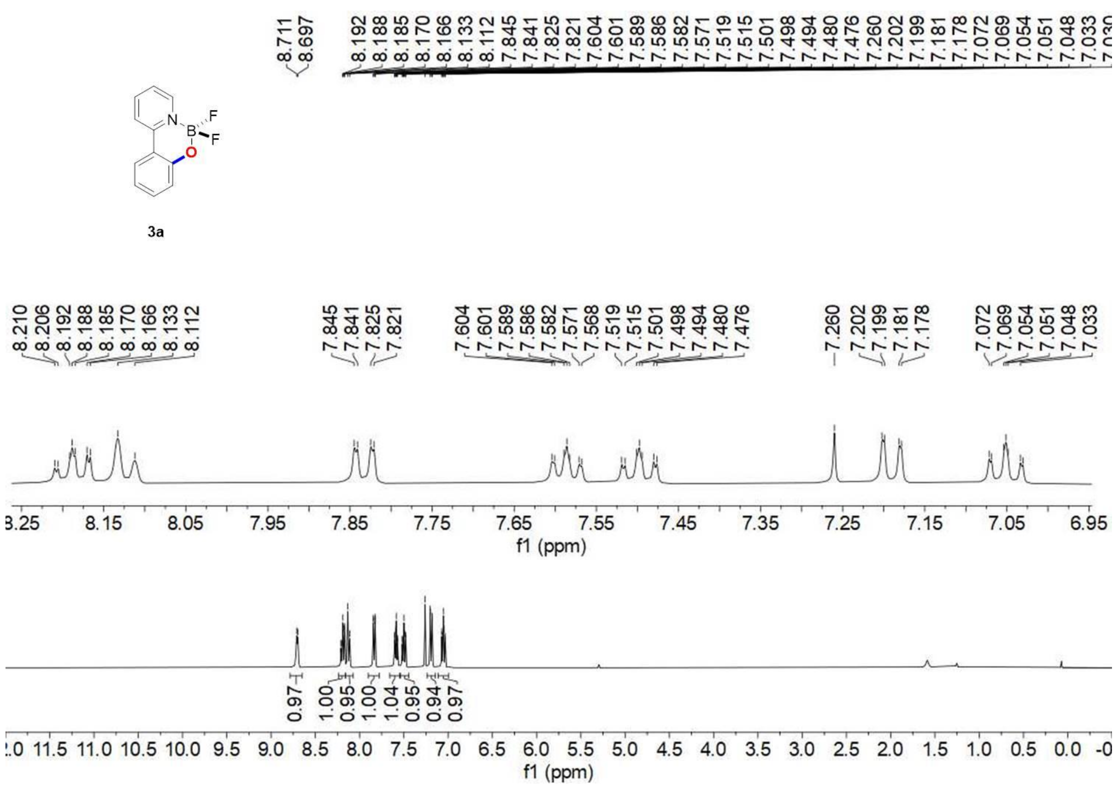

3a

8.711
8.697
8.192 8.188 8.185 8.170 8.166 8.133 8.112 7.845 7.841 7.825 7.821 7.604 7.601 7.589 7.586 7.582 7.571 7.519 7.515 7.501 7.498 7.494 7.480 7.476 7.260 7.202 7.199 7.181 7.178 7.072 7.069 7.054 7.051 7.048 7.033
8.210 8.206 8.192 8.188 8.185 8.170 8.166 8.133 8.112
7.845 7.841 7.825 7.821
7.604 7.601 7.589 7.586 7.582 7.571 7.568 7.519 7.515 7.501 7.498 7.494 7.480 7.476
7.260 7.202 7.199 7.181 7.178
7.072 7.069 7.054 7.051 7.048 7.033
f1 (ppm)
0.97 1.00 0.95 1.00 1.04 0.95 0.94 0.97
f1 (ppm)

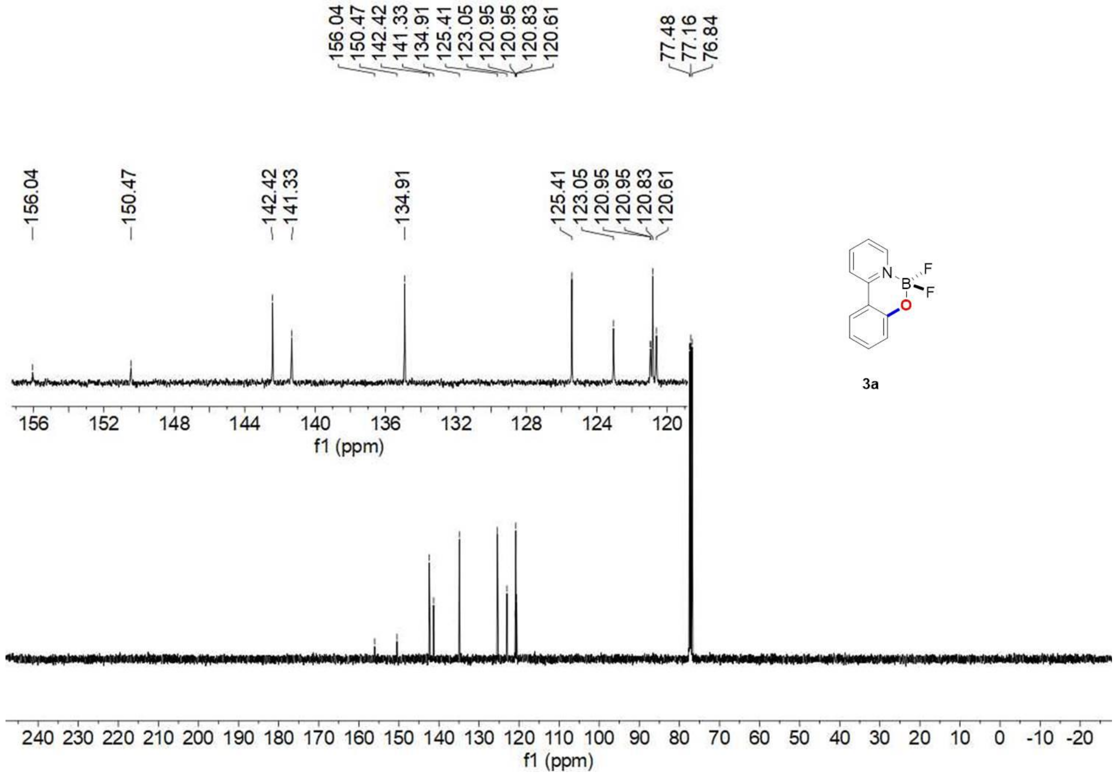

156.04 150.47 142.42 141.33 134.91 125.41 123.05 120.95 120.95 120.83 120.61
77.48 77.16 76.84
156.04
150.47
142.42 141.33
134.91
125.41 123.05 120.95 120.95 120.83 120.61
f1 (ppm)
f1 (ppm)

3a

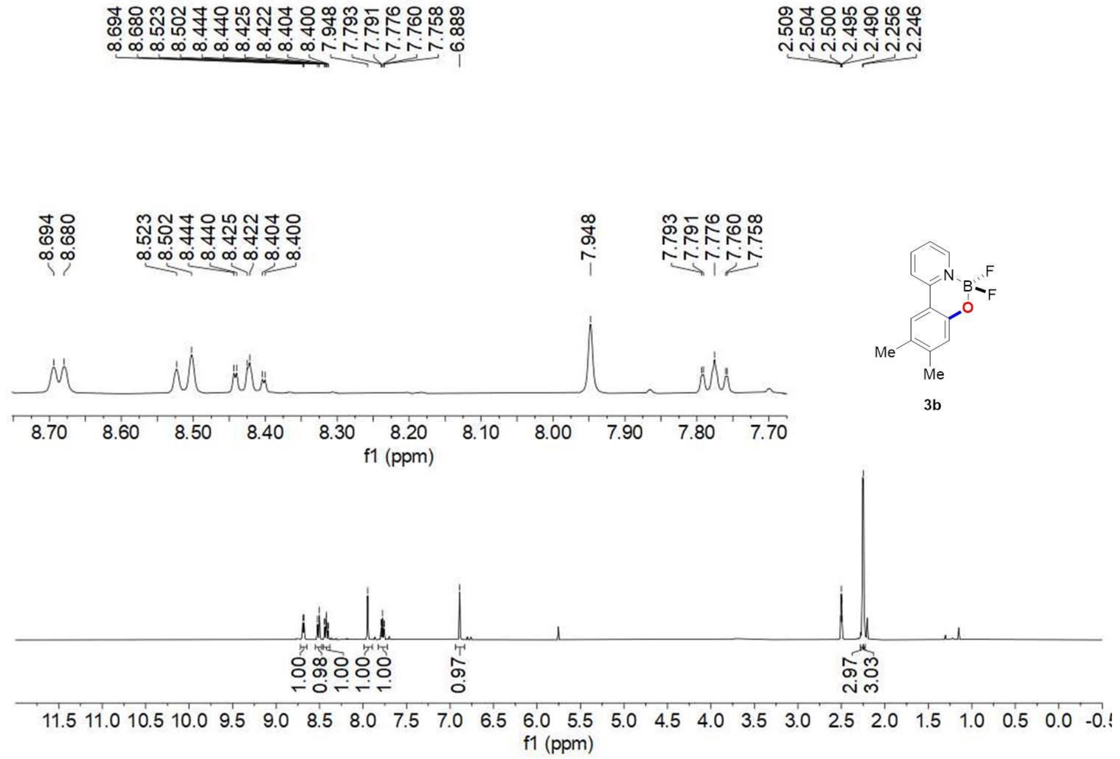
8.694
8.680
8.523
8.502
8.444
8.440
8.425
8.422
8.404
8.400
7.948
7.793
7.791
7.776
7.760
7.758
6.889
2.509
2.504
2.500
2.495
2.490
2.256
2.246
Me
Me
N
B
F
F
O
3b
1.00
0.98
1.00
1.00
1.00
0.97
2.97
3.03
f1 (ppm)

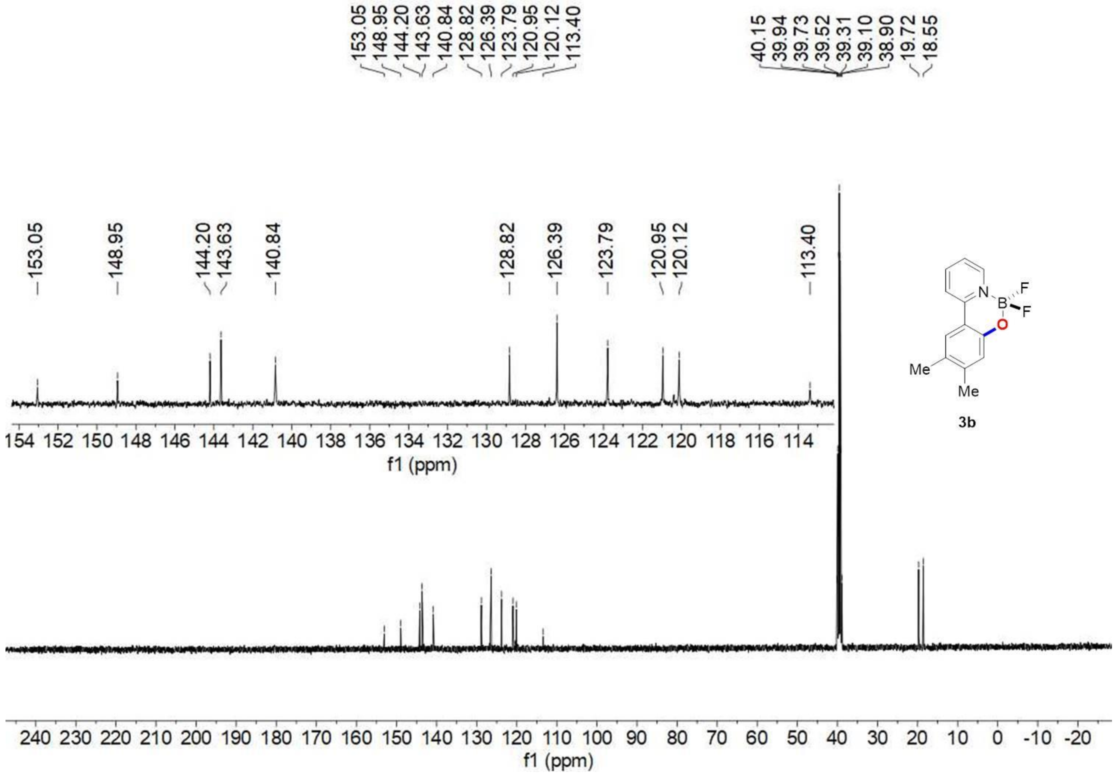
153.05
148.95
144.20
143.63
140.84
128.82
126.39
123.79
120.95
120.12
113.40
40.15
39.94
39.73
39.52
39.31
39.10
38.90
19.72
18.55
Me
Me
N
B
F
F
O
3b
f1 (ppm)

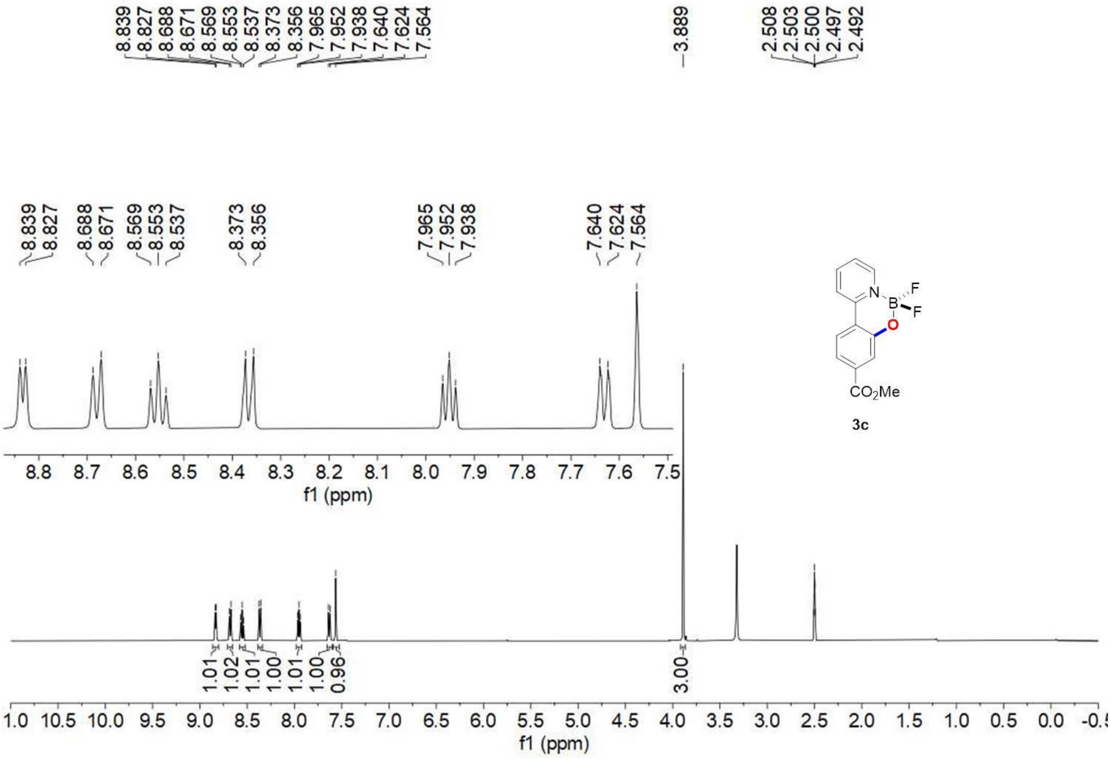
8.839
8.827
8.688
8.671
8.569
8.553
8.537
8.373
8.356
7.965
7.952
7.938
7.640
7.624
7.564
3.889
2.508
2.503
2.500
2.497
2.492
N
B
F
F
O
CO2Me
3c
f1 (ppm)
1.01
1.02
1.01
1.00
1.01
1.00
0.96
3.00
f1 (ppm)

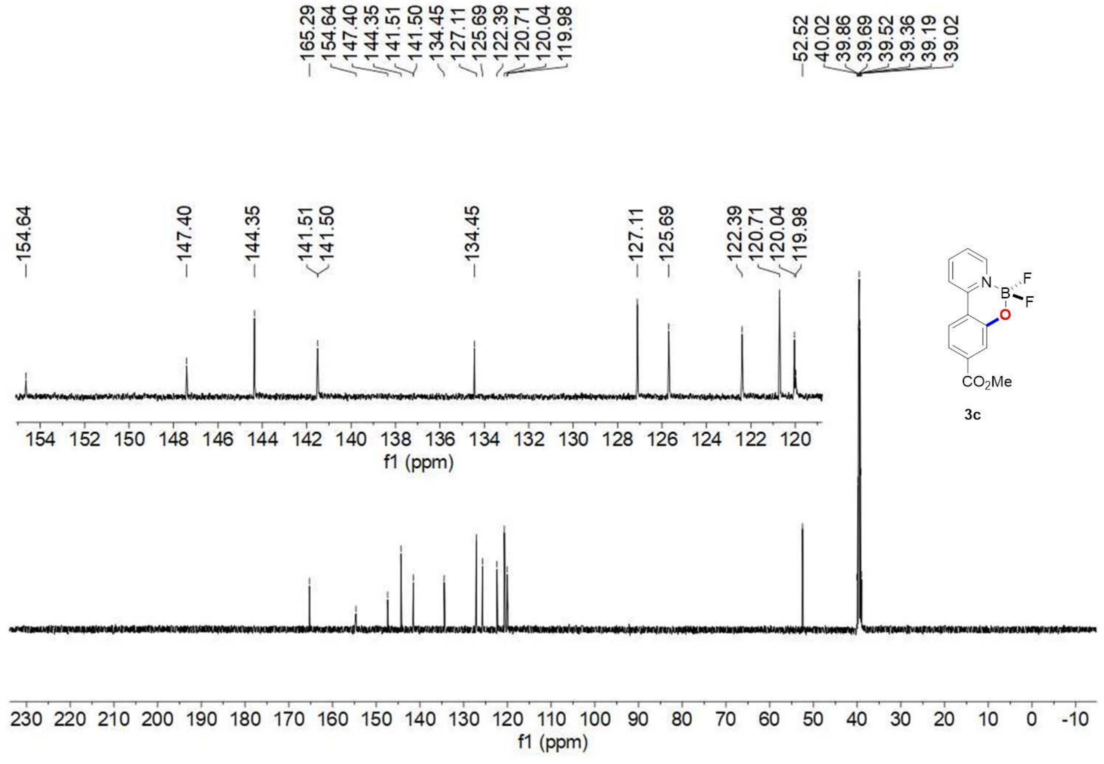
165.29
154.64
147.40
144.35
141.51
141.50
134.45
127.11
125.69
122.39
120.71
120.04
119.98
52.52
40.02
39.86
39.69
39.52
39.36
39.19
39.02
N
B
F
F
O
CO2Me
3c
f1 (ppm)
f1 (ppm)

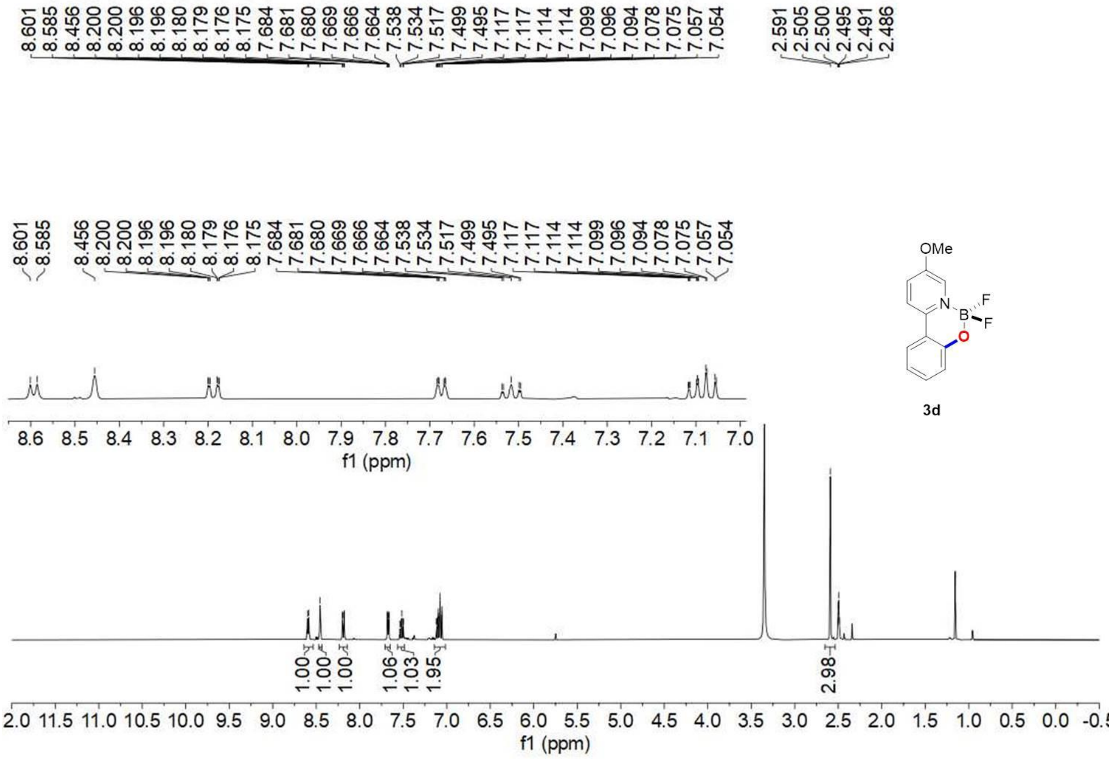
8.601 8.585 8.456 8.200 8.200 8.196 8.196 8.180 8.179 8.176 8.175 7.684 7.681 7.680 7.669 7.666 7.664 7.538 7.534 7.517 7.499 7.495 7.117 7.117 7.114 7.114 7.099 7.096 7.094 7.078 7.075 7.057 7.054
2.591 2.505 2.500 2.495 2.491 2.486
OMe
N
B
F
F
O
3d
1.00
1.00
1.00
1.06
1.03
1.95
2.98
f1 (ppm)

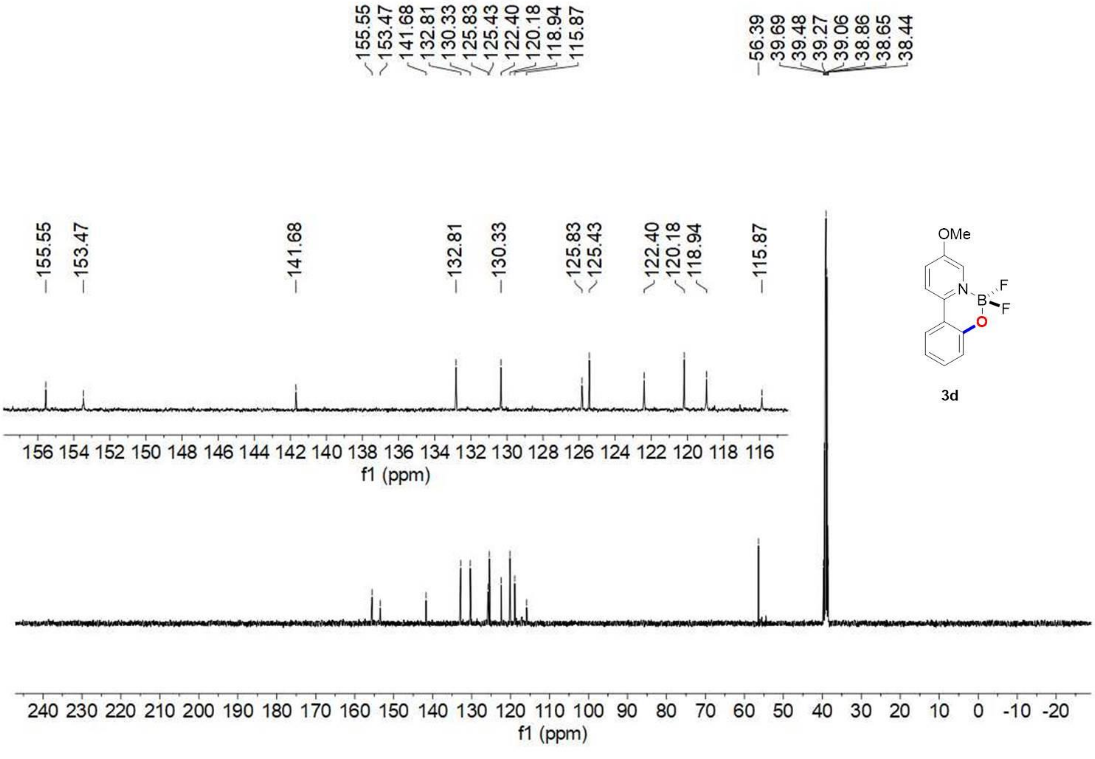
155.55 153.47 141.68 132.81 130.33 125.83 125.43 122.40 120.18 118.94 115.87
56.39 39.69 39.48 39.27 39.06 38.86 38.65 38.44
OMe
N
B
F
F
O
3d
f1 (ppm)

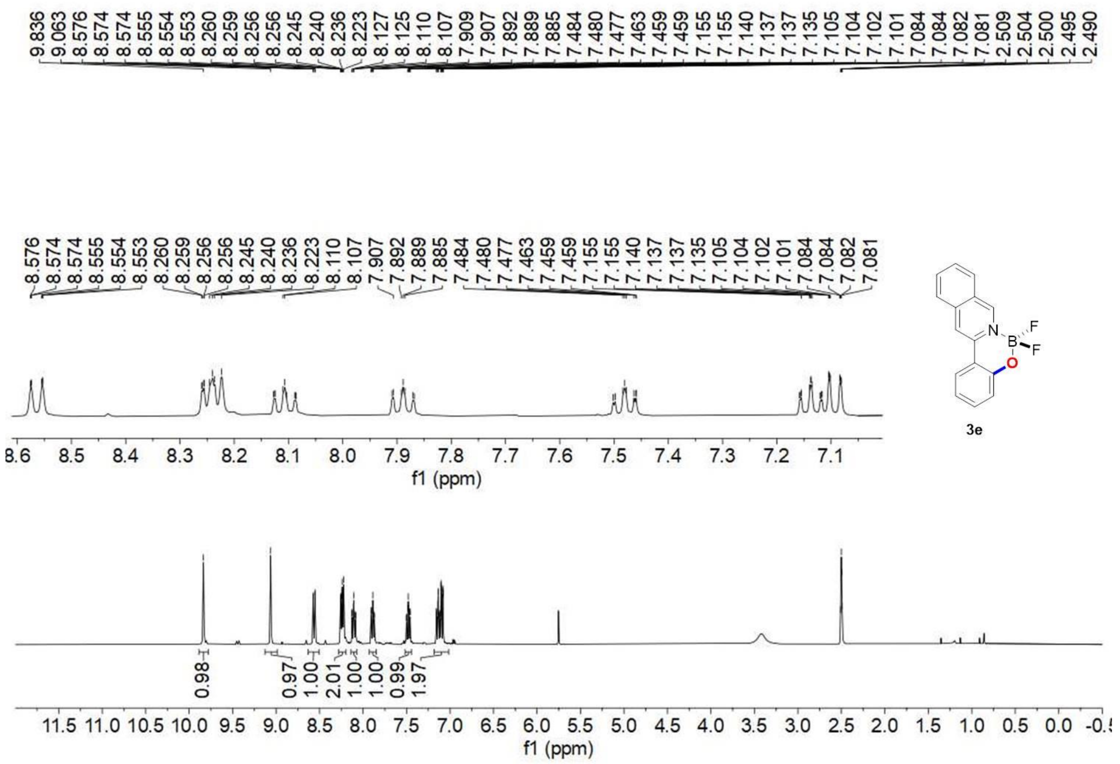
9.836 9.063 8.576 8.574 8.574 8.555 8.554 8.553 8.260 8.259 8.256 8.256 8.245 8.240 8.236 8.223 8.127 8.125 8.110 8.107 7.909 7.907 7.892 7.889 7.885 7.484 7.480 7.477 7.463 7.459 7.459 7.155 7.155 7.140 7.137 7.137 7.135 7.105 7.104 7.102 7.101 7.084 7.084 7.082 7.081 2.509 2.504 2.500 2.495 2.490
3e
f1 (ppm)
0.98 0.97 1.00 2.01 1.00 1.00 0.99 1.97
f1 (ppm)

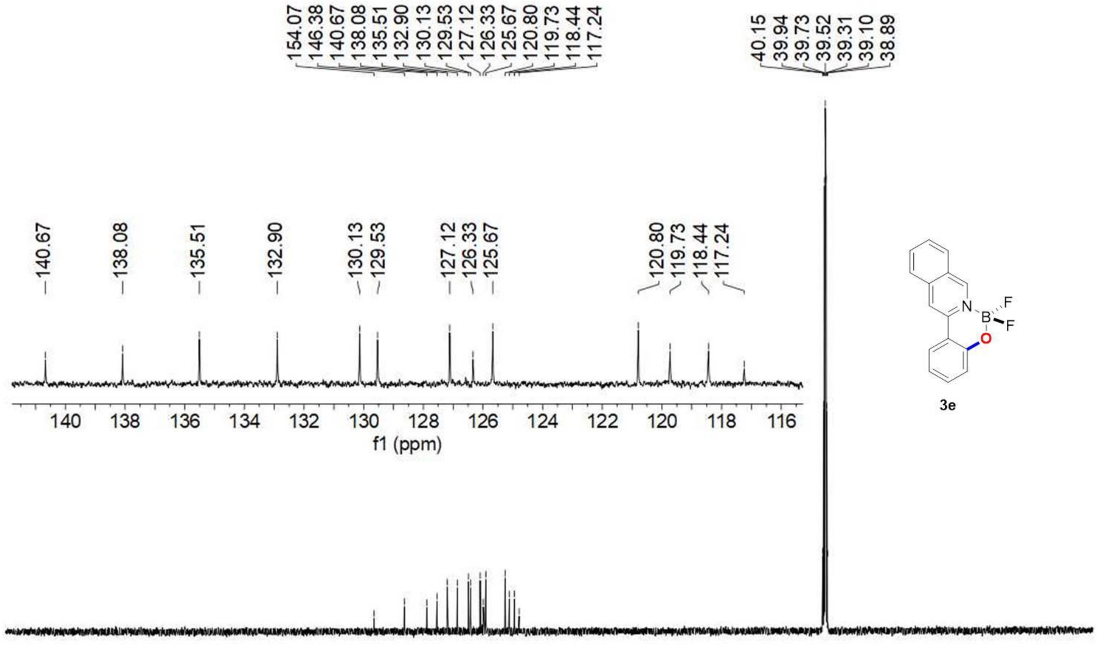
154.07 146.38 140.67 138.08 135.51 132.90 130.13 129.53 127.12 126.33 125.67 120.80 119.73 118.44 117.24
40.15 39.94 39.73 39.52 39.31 39.10 38.89
3e
f1 (ppm)
f1 (ppm)

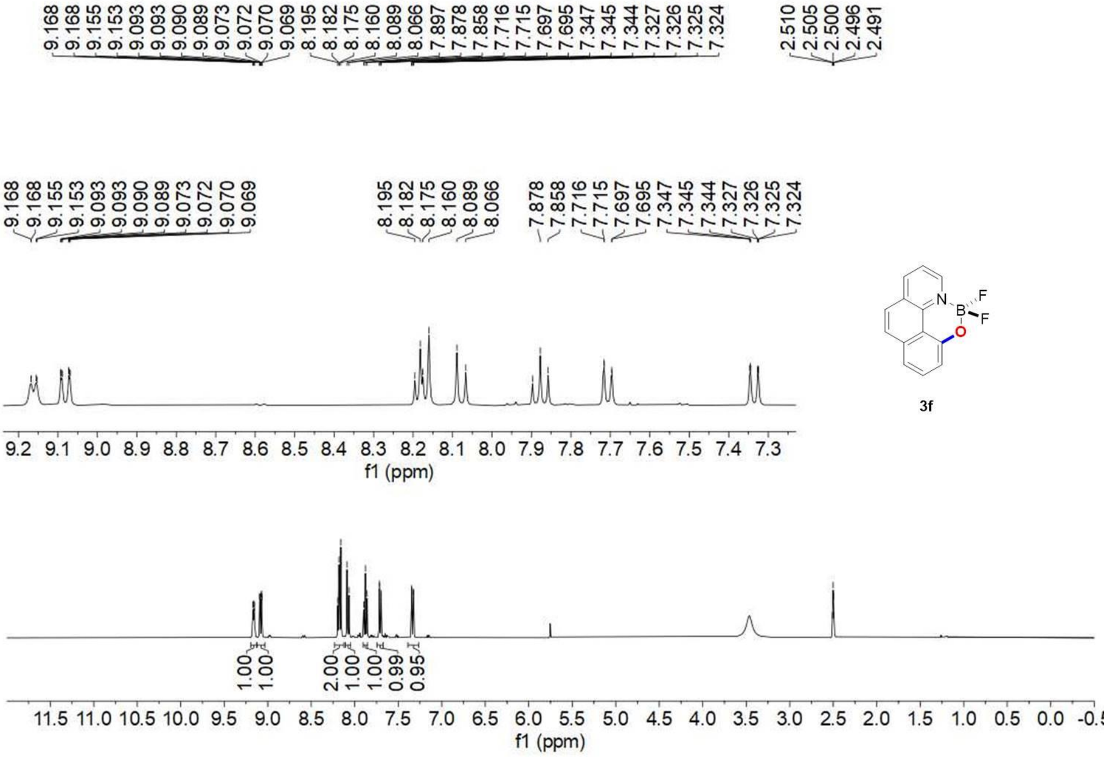

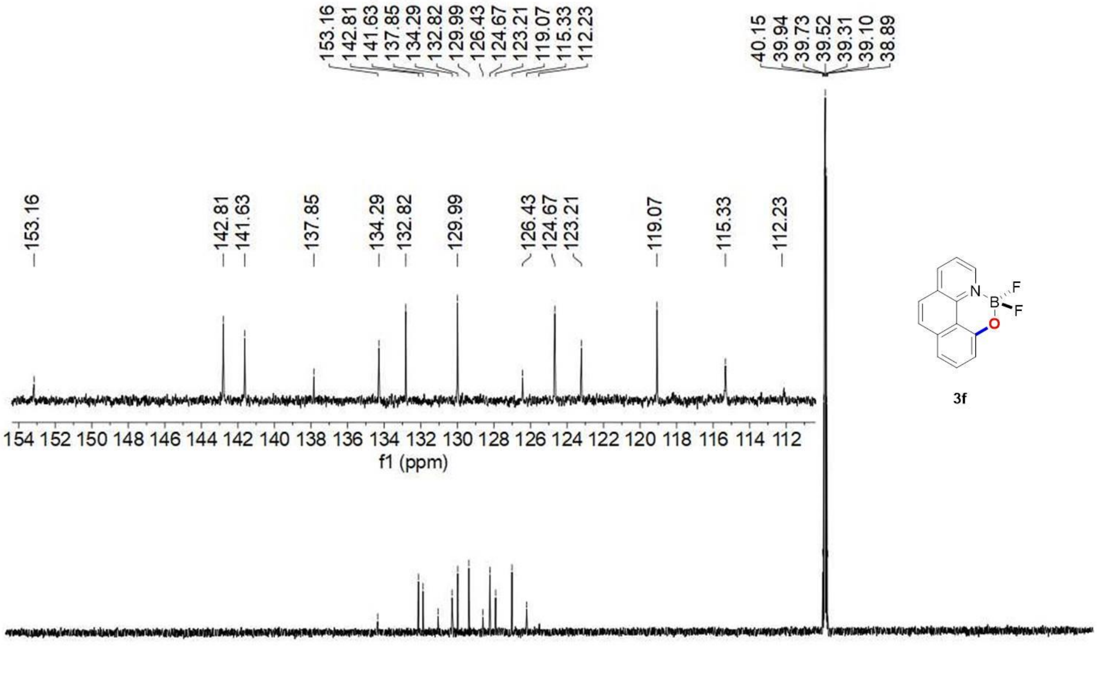

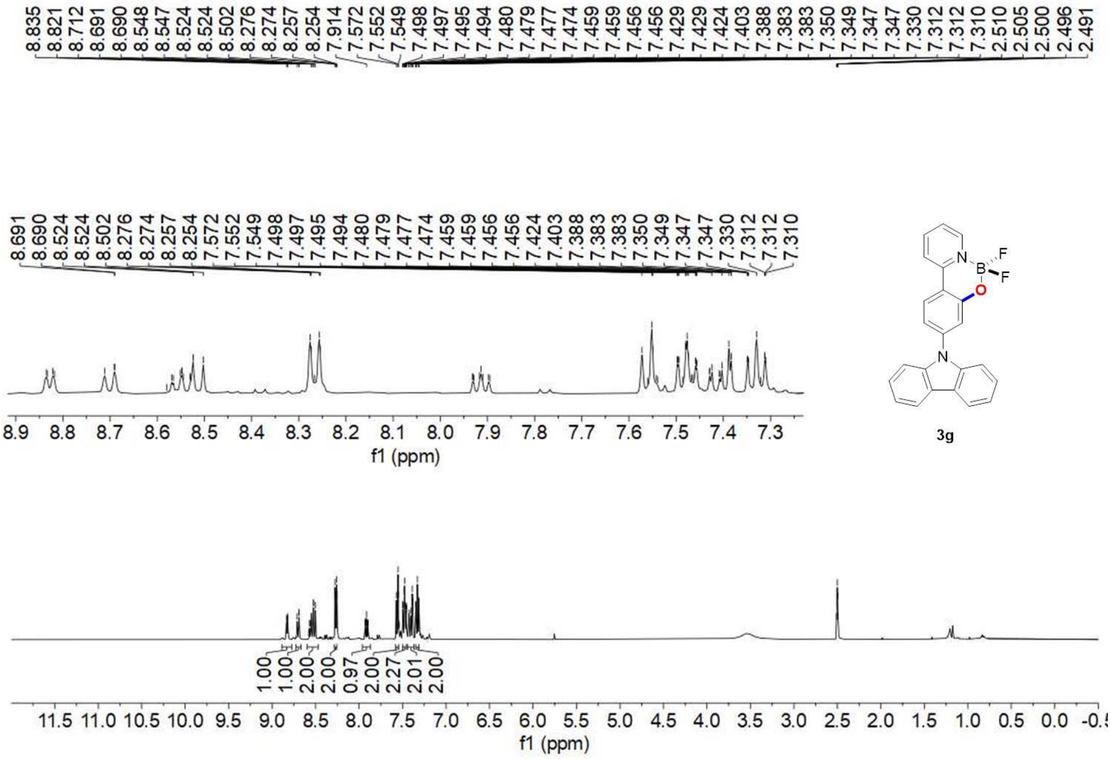
8.835 8.821 8.712 8.691 8.690 8.548 8.547 8.524 8.524 8.502 8.276 8.274 8.257 8.254 7.914 7.572 7.552 7.549 7.498 7.497 7.495 7.494 7.480 7.479 7.477 7.474 7.459 7.459 7.456 7.456 7.429 7.429 7.424 7.403 7.388 7.383 7.383 7.350 7.349 7.347 7.347 7.330 7.312 7.312 7.310 2.510 2.505 2.500 2.496 2.491
8.691 8.690 8.524 8.524 8.502 8.276 8.274 8.257 8.254 7.572 7.552 7.549 7.498 7.497 7.495 7.494 7.480 7.479 7.477 7.474 7.459 7.459 7.456 7.456 7.424 7.403 7.388 7.383 7.383 7.350 7.347 7.347 7.330 7.312 7.312 7.310
f1 (ppm)
1.00 1.00 2.00 2.00 0.97 2.00 2.27 2.01 2.00
f1 (ppm)
N
B
F
F
O
N

3g

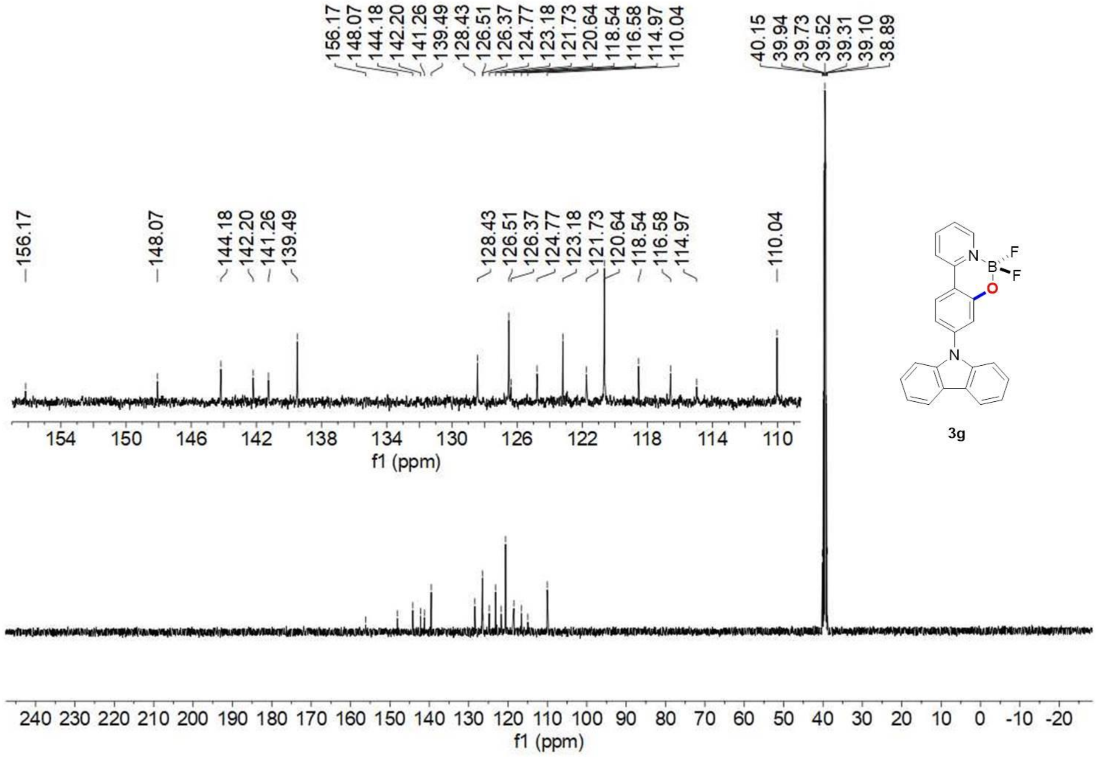
156.17 148.07 144.18 142.20 141.26 139.49 128.43 126.51 126.37 124.77 123.18 121.73 120.64 118.54 116.58 114.97 110.04 40.15 39.94 39.73 39.52 39.31 39.10 38.89
156.17 148.07 144.18 142.20 141.26 139.49 128.43 126.51 126.37 124.77 123.18 121.73 120.64 118.54 116.58 114.97 110.04
f1 (ppm)
f1 (ppm)
N
B
F
F
O
N

3g

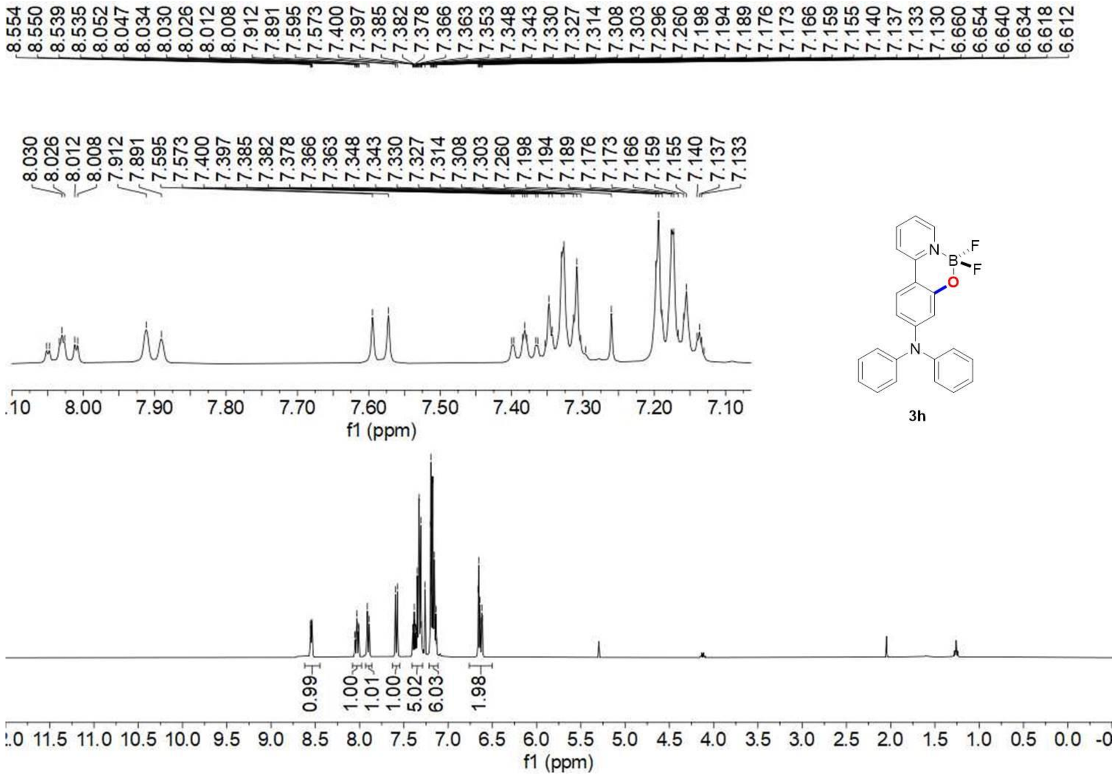
8.554 8.550 8.539 8.535 8.052 8.047 8.034 8.030 8.026 8.012 8.008 7.912 7.891 7.595 7.573 7.400 7.397 7.385 7.382 7.378 7.366 7.363 7.353 7.348 7.343 7.330 7.327 7.314 7.308 7.303 7.296 7.260 7.198 7.194 7.189 7.176 7.173 7.166 7.159 7.155 7.140 7.137 7.133 7.130 6.660 6.654 6.640 6.634 6.618 6.612
0.99 1.00 1.01 1.00 5.02 6.03 1.98
f1 (ppm)

3h

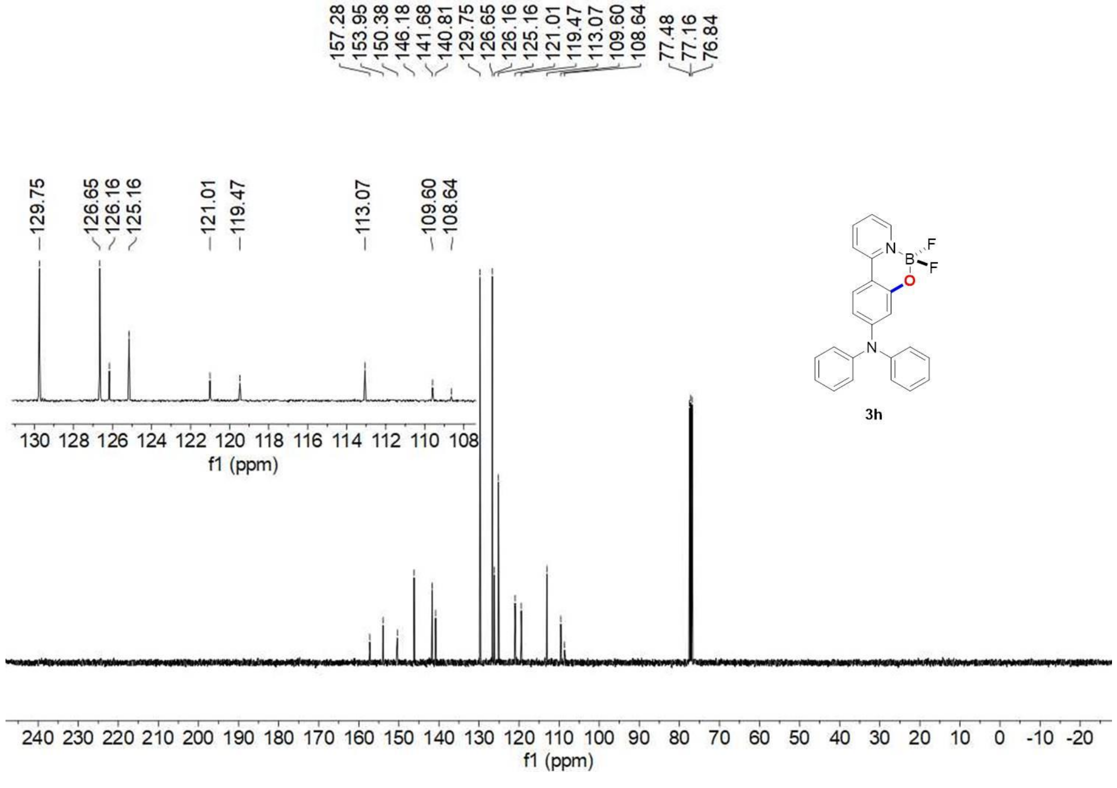
157.28 153.95 150.38 146.18 141.68 140.81 129.75 126.65 126.16 125.16 121.01 119.47 113.07 109.60 108.64 77.48 77.16 76.84
f1 (ppm)

3h
